# Extracellular vesicle proteins monitor and interfere with CLDN18.2-targeted CAR-T cell and antibody therapies against gastrointestinal cancers

**DOI:** 10.1038/s41467-026-77132-9

**Published:** 2026-09-03

**Authors:** Changsong Qi, Mingyang Ma, Kaijie Liang, Chang Liu, Jiarui Li, Yakun Wang, Dan Liu, Miao Zhang, Xiaoyi Chong, Fangli Jiang, Shengde Liu, Zizhen Zhang, Congcong Ji, Yuezong Bai, Meng Fan, Chuanhui Han, Xiaotian Zhang, Jifang Gong, Jian Li, Lin Shen, Cheng Zhang

**Affiliations:** 1 State Key Laboratory of Holistic Integrative Management of Gastrointestinal Cancers, Beijing Key Laboratory of Carcinogenesis and Translational Research, Department of Gastrointestinal Oncology, Peking University Cancer Hospital & Institute, Beijing, China; 2 State Key Laboratory of Holistic Integrative Management of Gastrointestinal Cancers, Beijing Key Laboratory of Carcinogenesis and Translational Research, Department of Early Drug Development Centre, Peking University Cancer Hospital & Institute, Beijing, China; 3 Key Laboratory of Carcinogenesis and Translational Research (Ministry of Education/Beijing), Department of Gastrointestinal Oncology, Peking University Cancer Hospital & Institute, Beijing, China; 4 Shanghai GenProAI Biotech Co., Ltd, Shanghai, China; 5 EVbio Technology Co., Ltd, Beijing, China; 6 State Key Laboratory of Molecular Oncology, Frontiers Science Center for Cancer Integrative Omics, Peking University Cancer Hospital and Institute, Peking University International Cancer Institute, Peking University Health Science Center, Beijing, China

**Keywords:** Translational research, Targeted therapies, Cancer immunotherapy

## Abstract

Claudin 18.2 (CLDN18.2)-targeted CAR-T cell and antibody therapies show promise against gastrointestinal cancers, but biomarkers predictive of long-term benefit and therapeutic sensitization strategies are needed. Here we show that proteins carried by circulating extracellular vesicles (EVs) can reflect and influence the efficacy of CLDN18.2-targeted therapies. By profiling serial plasma samples of patients from the CT041-CG4006 trial (CLDN18.2- CAR-T-therapy) or the GLOW trial (Zolbetuximab), we identified EV-associated CLDN18.2, PD1 and PD-L2 as therapeutic markers and integrated them into a signature (CPP-score) for better predictive robustness. Mechanistically, specific EV-proteins exhaust CAR-T cells, neutralize CLDN18.2-targeted-antibodies, activate immunosuppressive IL-6-expressing cancer-associated fibroblasts, and alter the TH1/TH2 balance, thereby reshaping the tumor microenvironment and interfere with CLDN18.2-targeted therapies. Strategies including combining anti-PD1 antibody or CAF inhibitor, CLDN18.2-CAR-T plus CLDN18.2-antibody, and depleting plasma EVs, can sensitize CLDN18.2-CAR-T/antibody by countermeasuring EV-proteins’ therapeutic interferences. Collectively, our work provides potential options to monitor and improve CLDN18.2-targeted therapies in clinical practice.

## Abstract (web summary)

CLDN18.2-targeted CART and antibody therapies are promising for gastrointestinal cancers, but predictive biomarkers and strategies to enhance efficacy are needed. Here, these authors investigate how extracellular vesicle (EV)- derived proteins influence therapeutic response to CLDN18.2-based CAR-T and targeted therapies.

## Introduction

CLDN18.2 (Claudin18.2) is a transmembrane protein variant encoded by CLDN18^1^, playing a vital role in the formation and integrity of tight junctions^2^. In healthy gastric tissue, CLDN18.2 expression is restricted to differentiated epithelial cells of the mucosa. However, cancer-related disruption of tight junctions and cell polarity exposes CLDN18.2 on tumor tissue surfaces^2,3^. In addition to gastric cancer (GC), CLDN18.2 is also highly expressed in other gastrointestinal (GI) cancers, including pancreatic cancer (PC) and cholangiocarcinoma (CHOL). Due to its tumor-specific expression and prevalence^4^, CLDN18.2 has become an ideal target for CAR-T (Chimeric Antigen Receptor T) cell therapy and antibody therapy in GI cancers^5^.

Our clinical trial (NCT03874897) for a CLDN18.2-targeted-CAR-T therapy, CT041-CG4006, demonstrated encouraging response rates in CLDN18.2-positive GI cancers^6–9^. Meanwhile, CLDN18.2-targeted monoclonal antibody Zolbetuximab showed substantial efficacy in CLDN18.2-positive gastric or gastroesophageal adenocarcinoma and achieved approval as a first-line therapy^10–12^. Ongoing clinical studies exploring other monoclonal/bispecific antibodies/antibody-drug conjugates also exhibited encouraging preliminary results. However, despite these advances, there is an urgent need for biomarkers to predict and monitor the efficacy of CLDN18.2-targeted therapies, while the response rate and survival benefits from current therapies also remain to be improved by additional interventions.

Extracellular vesicles (EVs) are membrane-bound, nanosized particles universally existing in body fluids like plasma and ascites^13^. EVs deliver cell-derived DNA/RNA/protein cargos, transmit signals, and manipulate biological functions^14^. Tumor immune microenvironment (TIME) changes during antitumor therapies are directly recorded and presented by EV-derived proteins, providing a valuable object for therapeutic prediction & monitoring. However, the liquid biopsy of EV-derived proteins instructing CLDN18.2-targeted CAR-T/antibody therapies remains unexplored. Beyond the scope of therapeutic biomarkers, specific EV-proteins’ influence on CLDN18.2-targeted-therapies’ efficacy also deserves to be investigated and intervened for the sake of therapeutic improvement.

In this study, we depicted the dynamic plasma EV-protein profiles for patients from the CT041-CG4006 and GLOW trials. We identified key EV-proteins that reflect the therapeutic efficacy, enabling the generation of an optimized signature to predict and monitor the efficacy of CLDN18.2-targeted CAR-T/antibody therapies. Through comprehensive bioinformatics analyses and experimental validation, we elucidated the roles and mechanisms of key EV proteins in modulating the tumor immune microenvironment (TIME) by influencing CLDN18.2-CAR-T cells, anti-CLDN18.2 antibodies, cancer-associated fibroblasts (CAFs), and the T helper cell balance, thereby undermining the efficacy of CLDN18.2-targeted CAR-T and antibody therapies. Inspired by key EV-proteins’ functions & mechanisms, we proposed strategies to enhance the effectiveness of CLDN18.2-targeted therapies.

## Results

### Overall study workflow and characteristics of patient cohorts

**Fig. 1:**
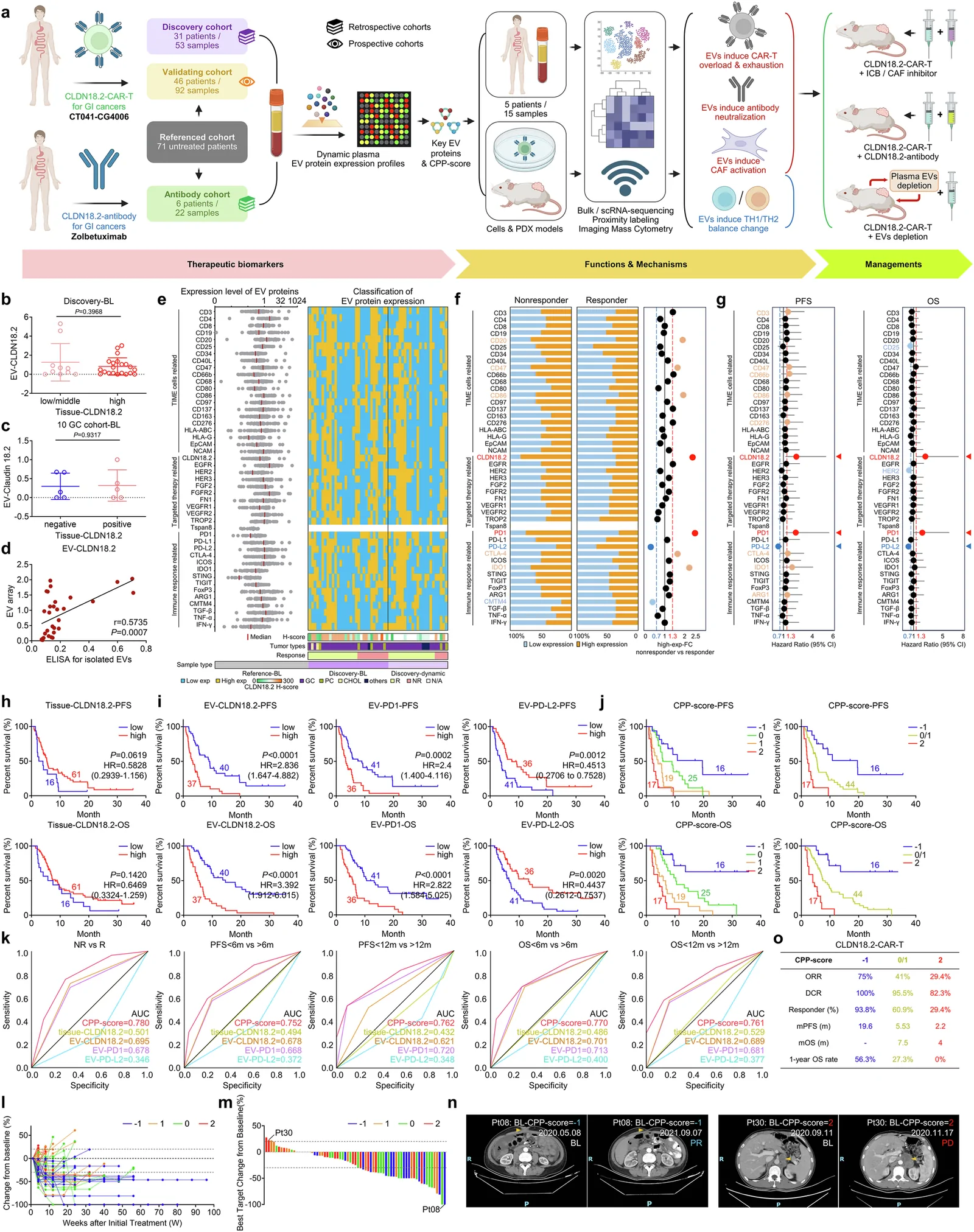
Key EV-proteins and derived CPP-score reflect the efficacy of CLDN18.2-targeted-CAR-T therapy. **a** Overall workflow chart. **b** Comparison of EV-array-based EV-CLDN18.2 expression between tissue CLDN18.2-low/middle (mean ± SD, *n* = 10) and tissue CLDN18.2-high GC patients (mean ± SD, *n* = 21) by two-sided *t*test. **c** Comparison of EV-array-based EV-CLDN18.2 expression between tissue-CLDN18.2-negative/positive GC patients (mean ± SD, neg/pos *n* = 5/5) by two-sided *t*test. **d** Pearson correlation between EV-array-based and ELISA-quantified EV-CLDN18.2 expression in 31 plasma samples obtained from independent patients the discovery cohort. **e** Classifying categorical expressions of each EV-proteins in discovery cohort. Expression status was defined using the median expression level of each EV protein in an independent reference cohort comprising 71 untreated patients with gastrointestinal cancers. **f** Comparing EV-proteins’ proportions of high-expression between responders and nonresponders of CLDN18.2-CAR-T to generated fold change (FC) values. **g** EV-proteins’ prognostic indications for PFS/OS in discovery cohort (Logrank test, dot=Hazard Ratio, band=95% CI of Hazard Ratio, triangle represents *P* < 0.05 for PFS/OS). In 77 patients treated with CLDN18.2-targeted-CAR-T, Kaplan-Meier analysis (Logrank test) for **h** IHC-based-tissue-CLDN18.2-related PFS/OS, **i** EV-CLDN18.2/EV-PD1/EV-PD-L2-related PFS/OS, **j** CPP-score-related PFS/OS were assessed. For 77 patients, **k** tissue-CLDN18.2/EV-proteins/CPP-score’s ROC assessment for efficacy of CLDN18.2-CAR-T, **l** CPP-score-stratified spider plot, and **m** CPP-score-stratified waterfall plot were depicted. **n** Representative CT image-series for representative patients (pt08: baseline-CPP-score = −1/best response=PR; pt30: baseline-CPP-score=2/best response=PD). **o** CPP-score-stratified major clinical outcomes of CLDN18.2-CAR-T therapy. **b–g** were based on the discovery cohort, comprising 31 independent patients. **h–o** were based on the combined CLDN18.2-targeted CAR-T cohort, comprising 77 independent patients, including 31 patients from the discovery cohort and 46 patients from the validating cohort. Group-specific sample sizes are indicated in the corresponding panels. Exact *P* values are provided in the corresponding panels.

We explored biomarkers for CLDN18.2-targeted-therapies in GI cancers (Fig. 1a). Among patients enrolled in CLDN18.2-targeted-CAR-T trial (CT041-CG4006, phase 1, NCT03874897), 77 with applicable plasma samples were included, in which 31 cases from the interim report^6^ (31 baseline/22 dynamic plasma) as a retrospective discovery cohort, another 46 cases from the final report^7^ (46 baseline/46 dynamic plasma) as a prospective validating cohort (Supplementary Fig. 1a–d, Supplementary Dataset 1). For CLDN18.2-targeted-antibody trial (GLOW, Zolbetuximab+CAPOX, phase 3, NCT03653507)^11^, 6 from our center (6 baseline/22 dynamic plasma) were retrospectively included as an antibody cohort (Supplementary Fig. 1e-1f, Supplementary Dataset 2). Plasma samples of another 71 untreated GI cancer patients served as a reference cohort for EV-proteins’ expression thresholds (Supplementary Dataset 3). All plasma samples were administered with an antibody-sandwiched EV expression-array (EV-array)^15^ to quantify EV-proteins. We selected 50 crucial EV-proteins related to TIME cells, immune checkpoint molecules, and therapeutic targets for cancers as the panel for EV-array quantification (Supplementary Dataset 4, 5).

### A plasma EV-protein expression landscape for discovery cohort

We generated an absolute expression spectrum for EV-proteins (Supplementary Fig. 2a). Four EV biomarkers (CD81/CD9/CD63/ALIX) showed stable expression across baseline and treatment timepoints (Supplementary Fig. 2b) with no correlation to response/PFS/OS (Supplementary Fig. 2c, d), indicating plasma EV content is unrelated to therapeutic outcomes. To normalize EV-protein expression, we combined EV-CD81 and EV-CD9, which showed the highest correlation amongst EV biomarkers (Supplementary Fig. 2e), creating a relative expression spectrum for further analysis (Supplementary Fig. 3a).

Relative EV-CLDN18.2 expression showed no correlation with tissue-CLDN18.2 IHC H-score at baseline or during treatment (Fig. 1b and Supplementary Fig. 3b, c). Further assessment in 10 GC patients revealed similar plasma EV-CLDN18.2 expressions between tissue-negative and tissue-positive groups (Fig. 1c and Supplementary Fig. 3d). Plasma EV expressions of T/B cell markers (CD3/CD19) were unrelated to circulating T/B cell proportions (Supplementary Fig. 3e, f), suggesting a different expressional landscape of plasma EV-proteins from tissue or circulating cell proteins. Furthermore, we isolated EVs through size-exclusion chromatography (SEC) method from 10 random plasma samples in the discovery cohort, then quality-controlled their shape, particle size, expression of EV markers and existence of representative EV-proteins in EV-array panel (Supplementary Fig. 3g–j). ELISA validation for CLDN18.2/PD1 expression in EVs isolated from 31 random plasma samples in the discovery cohort confirmed that EV expression array reliably captures a broad dynamic range of expression levels for specific EV-proteins (Fig. 1d and Supplementary Fig. 3k).

### Key EV-Proteins and CPP-Score reflect CLDN18.2-targeted-CAR-T’s therapy efficacy

In discovery cohort, we assessed dynamic correlations between EV-protein expressions (Supplementary Fig. 4) and categorized EV-proteins as “high” or “low” based on the reference cohort’s medians (Fig. 1e). Tissue-CLDN18.2 expression was largely unrelated to PFS/OS of CLDN18.2-CAR-T therapy (Supplementary Fig. 5a). High-expression rates in nonresponders/responders were calculated into fold changes (FC) for all EV-proteins, with FC ≥ 1.3/FC ≤ 0.7 considered as cutoffs for responsive/nonresponsive EV-proteins. For Kaplan-Meier analysis, HR ≥ 1.3/HR ≤ 0.7 were considered as favorable/adverse-prognostic EV-proteins. Only three EV-proteins simultaneously met the thresholds for response/PFS/OS: positively for EV-CLDN18.2/EV-PD1 and negatively for EV-PD-L2 (Fig. 1f, g and Supplementary Fig. 5b). These EV-proteins are mutually uncorrelated prognostic markers (Supplementary Fig. 4).

To improve predictive power, we generated a plasma EV-protein-based signature (CPP-score, ranging from -1 to 2) by counting “high” expressions of EV-CLDN18.2 plus EV-PD1 minus EV-PD-L2. Baseline-CPP-score correlated negatively with PFS/OS (Supplementary Fig. 5c), outperforming tissue-CLDN18.2/EV-CLDN18.2/EV-PD1/EV-PD-L2 in predicting response and outcome (Supplementary Fig. 5d). Also, patients bearing low baseline-CPP-score showed better tumor volume regression (Supplementary Fig. 5e, f). Throughout CLDN18.2-CAR-T treatment, EV-CLDN18.2/EV-PD1/CPP-score were higher while EV-PD-L2 was lower in patients for nonresponders versus responders (Supplementary Fig. 5g), in PFS < 6 m versus PFS > 6 m patients (Supplementary Fig. 5h) and in OS < 12 m versus in OS > 12 m (Supplementary Fig. 5i), proving key EV-proteins/CPP-score can dynamically monitor CLDN18.2-CAR-T therapy. On the other hand, CPP-score remained stable throughout CLDN18.2-CAR-T treatment (Supplementary Fig. 5j), while dynamic CAR-T cell copies (probe & primers shown in Supplementary Table 1) were lower for patients with high baseline-EV-CLDN18.2/EV-PD1/CPP-score and higher for patients with high baseline-EV-PD-L2 (Supplementary Fig. 5k). These data indicated that EV-CLDN18.2/EV-PD1 were related to reduced, while EV-PD-L2 was related with increased CAR-T cell content.

### Prospectively validating EV-proteins and CPP-Score’s predictive power for CLDN18.2-CAR-T therapy

We prospectively recruited an additional 46 CLDN18.2-CAR-T-treated patients (validating cohort) to generate an absolute EV-protein expression spectrum (Supplementary Fig. 6a). Total-plasma EV content remained stable across timepoints (Supplementary Fig. 6b), thus, we combined highly-correlated EV-CD81/EV-CD9 (Supplementary Fig. 6c) to generate a relative expression spectrum (Supplementary Fig. 7a). Relative expressions of EV-CLDN18.2/EV-CD3/EV-CD19 were unrelated with tissue-CLDN18.2 expression or circulating T/B cell proportions (Supplementary Fig. 7b–e). We categorized each EV-protein’s expression as “high”/“low” based on reference cohort (Supplementary Fig. 8a, b) and generated CPP-score. EV-CLDN18.2/EV-PD1 negatively while EV-PD-L2 positively correlated with CLDN18.2-CAR-T’s efficacy (Supplementary Figs. 8c, d, 9b). Baseline-CPP-score more powerfully predicted poor PFS/OS than tissue-CLDN18.2/EV-CLDN18.2/EV-PD1/EV-PD-L2 (Supplementary Fig. 9a–d), as confirmed by tumor volume changes (Supplementary Fig. 9e, f). Dynamically, EV-CLDN18.2/EV-PD1/CPP-score was higher in nonresponders (Supplementary Fig. 9g) and in PFS < 6 m (Supplementary Fig. 9h) or OS < 12 m (Supplementary Fig. 9i) patients. CPP-score remained dynamically stable throughout treatment (Supplementary Fig. 9j), and dynamic CAR copies in peripheral blood were lower for high baseline-CPP-score patients (Supplementary Fig. 9k).

**Fig. 2:**
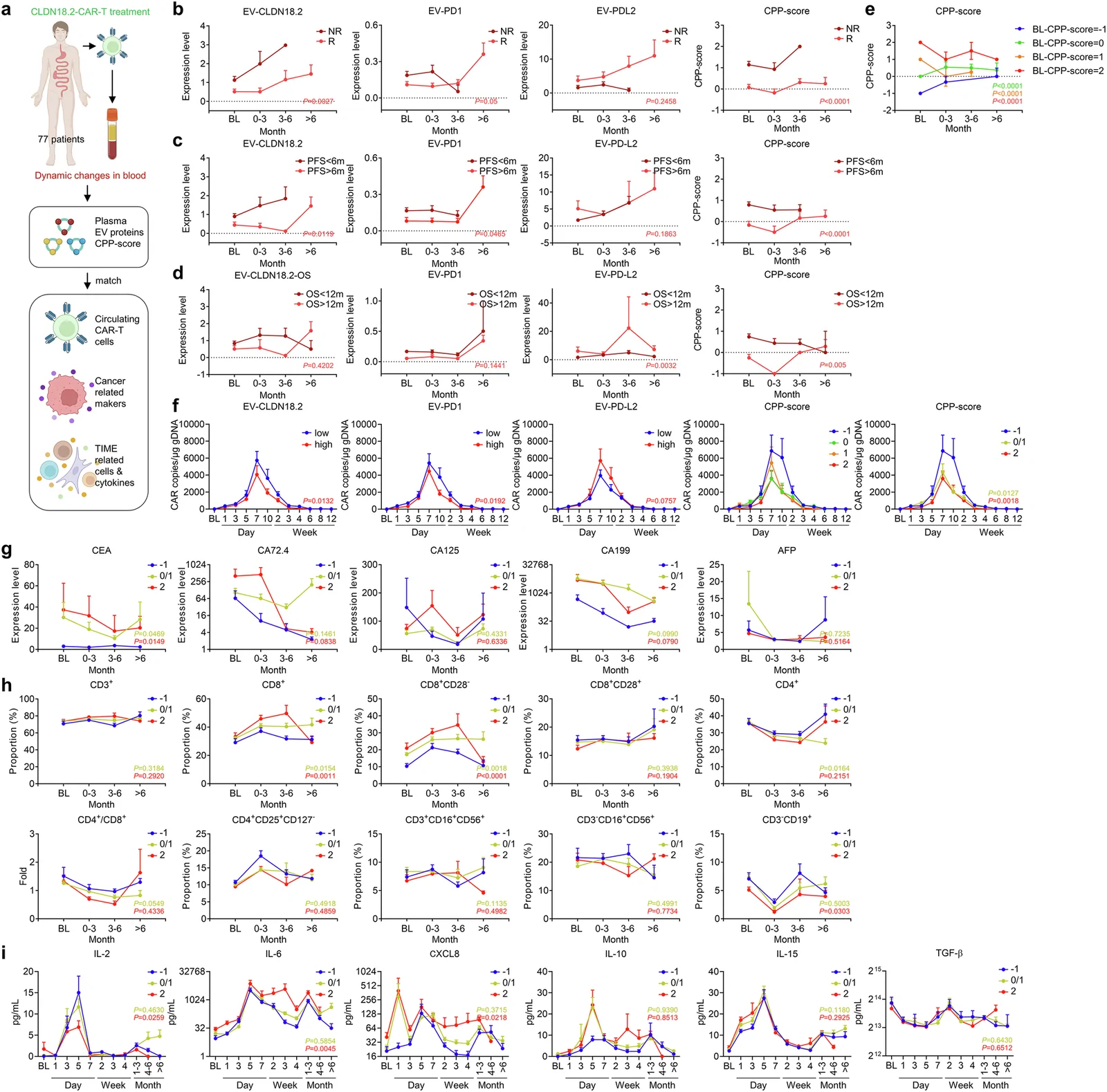
CPP-score reflects immunosuppressive tumor microenvironmental features related with CLDN18.2-targeted-CAR-T therapy. **a** A schematic chart for the assessment of dynamic changes in blood. Dynamic EV-CLDN18.2/EV-PD1/EV-PD-L2/CPP-score between **b** nonresponders (NR, mean ± SD, *n* = 29)/responders (R, mean ± SD, *n* = 45), **c** PFS < 6 m (mean ± SD, *n* = 52)/PFS > 6 m (mean ± SD, *n* = 25) patients, **d** OS < 12 m (mean ± SD, *n* = 57)/OS > 12 m (mean ± SD, *n* = 20) patients. **e** Dynamic EV-CLDN18.2/EV-PD1/EV-PD-L2/CPP-score stratified by baseline-CPP-score (mean ± SD, −1/0/1/2 *n* = 16/25/19/17). **f** Dynamic CAR-T cell content stratified by baseline-EV-CLDN18.2 (mean ± SD, low/high *n* = 32/26)/EV-PD1 (mean ± SD, low/high *n* = 31/27)/EV-PD-L2 (mean ± SD, low/high *n* = 34/24)/CPP-score (mean ± SD, −1/0/1/2 *n* = 12/19/13/14). **g** Dynamic tumor markers stratified by baseline-CPP-score (mean ± SD, −1/0/1/2 *n* = 16/25/19/17). **h** Dynamic immune cell proportions stratified by baseline-CPP-score (mean ± SD, −1/0/1/2 *n* = 16/25/19/17). **i** Dynamic cytokines stratified by baseline-CPP-score (mean ± SD, −1/0/1/2 *n* = 16/25/19/17). For **b–i** were based on the combined CLDN18.2-targeted CAR-T cohort. n denotes independent patients with available serial blood measurements. Each patient contributed repeated measurements at the indicated time points. Statistical differences were assessed using two-way repeated-measures ANOVA. Exact two-sided *P* values are shown in the corresponding panels.

For both discovery and validating cohorts, COX multivariate analysis for the 4 parameters confirmed EV-CLDN18.2/EV-PD1/EV-PD-L2 were more prognostic than CLDN18.2-H-score (Supplementary Table 2). The CPP-score outperformed a logistic regression model (CPP-logistics) for positive control in predicting CLDN18.2-CAR-T efficacy (Supplementary Fig. 10a–d). Therefore, we combined all 77 patients from both cohorts to more effectively illustrate the clinical relevance of key EV-proteins and the CPP-score. For all 77 patients from both cohorts, at baseline, tissue-CLDN18.2 was non-prognostic (Fig. 1h), EV-CLDN18.2/EV-PD1/EV-PD-L2 reflected negative/negative/positive PFS/OS (Fig. 1i), while the CPP-score more robustly reflected negative PFS/OS than single EV-proteins (Fig. 1j, k), with tumor volume changes for all patients (Fig. 1l, m) and CT image-series for representative patients (baseline-CPP-score = -1/2, Fig. 1n) confirmed its predictive strength. Collectively, CPP-score powerfully reflected poor clinical outcomes of CLDN18.2-CAR-T therapy (Fig. 1o and Supplementary Dataset 6). CPP-score’s validity was further supported in both GC (Supplementary Fig. 11a–c) and non-GC cancers (Supplementary Fig. 11d–f). In 77 patients’ blood samples (Fig. 2a), the capability to dynamically monitor CAR-T’s efficacy (Fig. 2b–e) and cell content (Fig. 2f) supports CPP-score as an optimized biomarker for CLDN18.2-CAR-T therapy in GI cancers.

### CPP-score reflects immunosuppressive tumor microenvironmental features related with CLDN18.2-targeted-CAR-T therapy

In addition to biomarkers, EV-proteins/CPP-score’ influence on TIME and CLDN18.2-CAR-T’s efficacy should be addressed. Combining 77 CLDN18.2-CAR-T-treated patients, we found among major tumor markers (CEA/CA72.4/CA125/CA199/AFP) that dynamic CEA level elevated from low to high-baseline-CPP-score cases (Fig. 2g). Among TIME cell features in circulation (total-T lymphocytes (CD3^+^), T helper (CD3^+^CD4^+^), cytotoxic T (CD3^+^CD8^+^), CD4^+^/CD8^+^ ratio, Tregs (CD4^+^CD25^+^CD127^-^), suppressed cytotoxic T (CD8^+^CD28^-^), activated cytotoxic T (CD8^+^CD28^+^), total-B lymphocytes (CD19^+^), NK (CD3^-^CD16^+^56^+^) and NK-T (CD3^+^CD16^+^CD56^+^)), dynamic proportions of activated cytotoxic T cells remained stable, while suppressed cytotoxic T cells increased from low to high baseline-CPP-score patients (Fig. 2h), suggesting links between EV-proteins and T-cell exhaustion. We also assessed the plasma cytokine dynamics (IL-2/IL-6/CXCL8/IL−10/IL-15/TGF-β) throughout CLDN18.2-CAR-T therapy. Fibroblast-related cytokines IL-6/CXCL8 dynamically elevated along with the CPP-score (Fig. 2i), suggesting a relationship between EV-proteins and cancer-associated fibroblasts. Furthermore, the duration of CLDN18.2-CAR-T’s adverse events (AEs) was reduced in high baseline-CPP-score cases (Supplementary Fig. 12a, b), aligning with the consensus that AEs correlate with immune activity and therapeutic efficacy. By comparing the dynamic plasma EV-proteins/CPP-score profiles and ctDNA-based genetic aberrations at baseline/day 7/day 28 of CLDN18.2-CAR-T treatment, we found high CPP-score was paired with several copy number variations (CNVs), especially MYC amplification (Supplementary Fig. 13a), yet was largely unrelated with single nucleotide variations (SNVs, Supplementary Fig. 13b). These data indicate that CPP-score reflects more tumor-associated genetic changes and more immunosuppressive TIME.

### EV-CLDN18.2 overloads and exhausts CLDN18.2-targeted-CAR-T cells

**Fig. 3:**
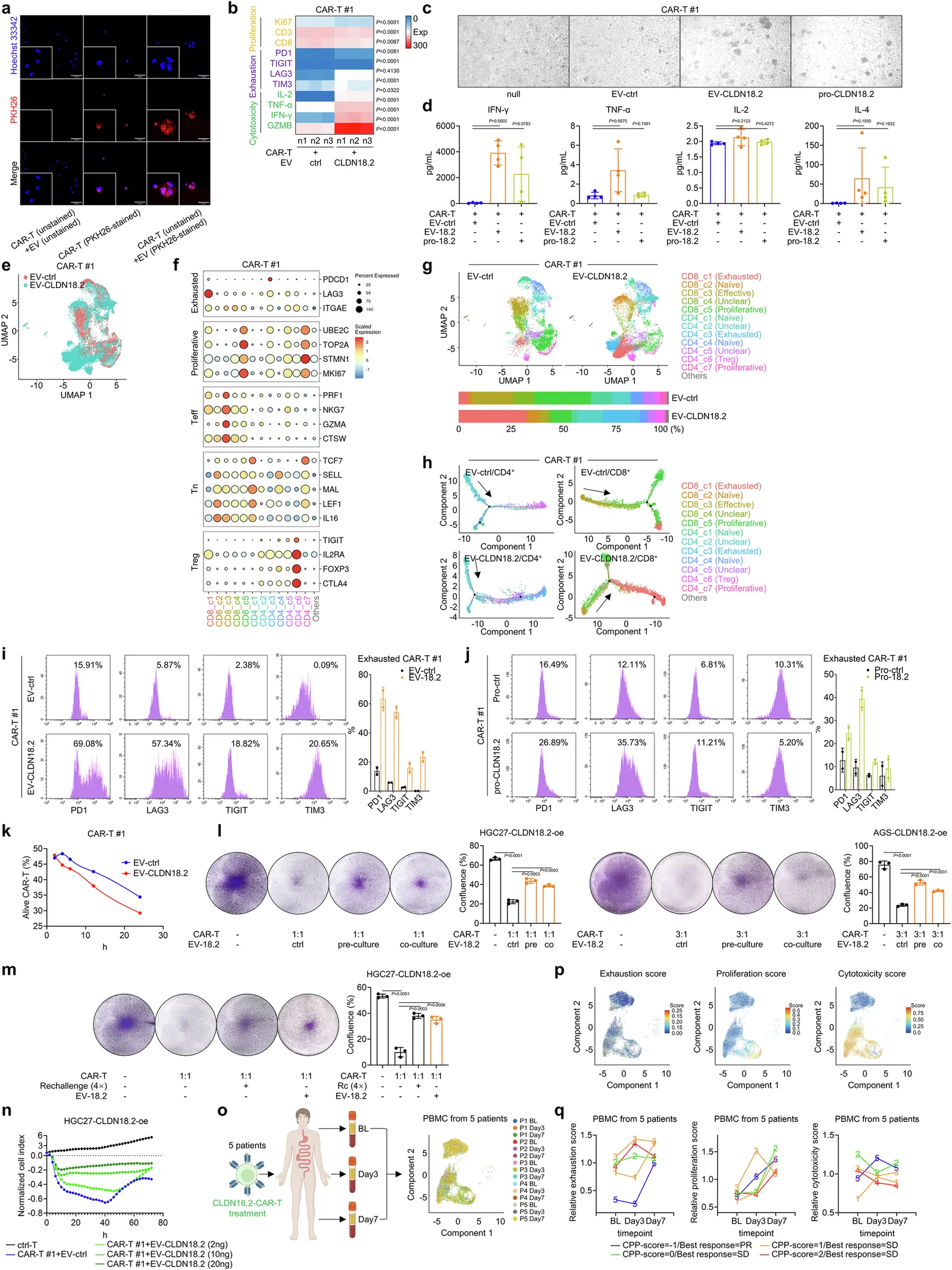
EV-CLDN18.2 overloads and exhausts CLDN18.2-targeted-CAR-T cells. **a** Fluorescent imaging for the uptake of PKH26-stained EVs by Hoechest 33342-stained CLDN18.2-CAR-T cells. Images show one typical field from two representative experiments. **b** Bulk RNA-sequencing of feature genes for proliferation/exhaustion/cytotoxicity in CLDN18.2-targed-CAR-T treated with EV-control or EV-CLDN18.2 (calculated from *n* = 3 independent CAR-T cell culture experiments, two-sided t-test). After EV-CLND18.2/pro-CLDN18.2 treatment, CLDN18.2-CAR-T cells’ **c** Representative morphology of CLDN18.2-targeted CAR-T cells following treatment with EV-CLDN18.2 or purified CLDN18.2 protein (pro-CLDN18.2). **d** expression of cytotoxic/suppressive cytokines were assessed (mean ± SD, calculated from *n* = 4 independent CAR-T cell preparations generated from four independent healthy donors, two-sided t-test). **e** UMAP for CLDN18.2-CAR-T-single cells from EV-ctrl/EV-CLDN18.2 treated groups. **f** Annotations for CAR-T’s clustering. **g** Featured clusters of CAR-T cell linages and their distributions before/after EV-CLDN18.2 treatment. **h** Trajectory analysis for CAR-T cell linages. **i** Effect of EV-CLDN18.2 and (**j**) effect of pro-CLDN18.2 on the exhaustion phenotype of CLDN18.2-targeted CAR-T cells. Data are presented as mean ± SD, from *n* = 2 independent cell-culture experiments using CAR-T cells generated from healthy donor #1. Flow-cytometry plots show one representative independent experiment, and bar graphs summarize both independent experiments. **k** EV-CLDN18.2’s influence on CLDN18.2-targeted-CAR-T’s viability. **l** CLDN18.2-CAR-T’s cytotoxic efficacy against CLDN18.2-overexpressing-HGC27/AGS cancer cells at different E:T ratios (mean ± SD, calculated from *n* = 3 independent cell-culture experiments, two-sided *t*test). **m** CAR-T cells were subjected to four consecutive rounds of rechallenge with CLDN18.2-overexpressing HGC27 cells (mean ± SD, calculated from *n* = 3 independent cell-culture experiments, two-sided t-test). **n** Dose-dependent impact of EV-CLDN18.2 on CLDN18.2-CAR-T’s cytotoxic efficacy. **o** UMAP for PBMC-single cells from five patients in CT041-CG4006 trial. **p** Exhaustion/proliferation/cytotoxicity scores for PBMC-single cells from five patients. **q** Joint analysis for exhaustion/proliferation/cytotoxicity scores, plasma CPP-score and patient response information from baseline to Day 7 of CLDN18.2-CAR-T treatment. **o–q** are based on longitudinal PBMC samples from *n* = 5 independent patients in the validating cohort of the CT041-CG4006 trial.Exact two-sided *P* values for the statistical comparisons in (**b**,**d**,**l**,**m**) are shown in the corresponding panels.

Consisting of EV-CLDN18.2, EV-PD1, and EV-PD-L2, the CPP-score powerfully predicts and monitors the therapeutic efficacy of separate CLDN18.2-CAR-T cohorts. Since CLDN18.2 is the target of CLDN18.2-CAR-T cells, we focused on EV-CLDN18.2’s effect on CLDN18.2-CAR-T efficacy. We isolated EVs from a HEK293F cell line overexpressing human CLDN18.2, and ensured the expression of EV-CLDN18.2 on isolated EVs through multiple quality control steps (Supplementary Fig. 14a–d). On the other hand, we transfected human T cells from four healthy donors to establish CLDN18.2-targeted-CAR-T strains (#1-#4) with >50% CAR expression (Supplementary Fig. 14e). Fluorescent staining proved EVs could be absorbed by CAR-T cells (Supplementary Fig. 14f and Fig. 3a), while bulk RNA-sequencing showed EV-CLDN18.2 treatment for 24 h dramatically upregulated cytotoxic effectors (TNF-α/IFN-γ/Granzyme B) while slightly enhance expression of proliferation (Ki67/CD8) and exhaustion markers (PD1/LAG3) in CLDN18.2-CAR-T cells (Fig. 3b), exhibiting both hyperactivation and exhaustion features. Culturing with EV-CLND18.2 or purified human-CLDN18.2 protein (pro-CLDN18.2) enhanced CLDN18.2-CAR-T’s aggregation (Fig. 3c) while stimulating both cytotoxic (IFN-γ/TNF-α) and suppressive (IL-4) cytokine expressions (Fig. 3d), also representing simultaneous activation and exhaustion of CAR-T cells.

CLDN18.2-CAR-T cells were then treated by EV-CLDN18.2 for 48 h, proceed for single-cell transcriptomic sequencing and clustered into linages (Fig. 3e) by referring to canonical annotations (Fig. 3f). EV-CLDN18.2 prominently increased the proportions of exhausted CD4^+^/CD8^+^ CAR-T cells and decreased proliferative and effective CAR-T cells (Fig. 3g and Supplementary Fig. 14g–i). Trajectory analysis confirmed that EV-CLDN18.2 promoted the evolution of CAR-T cells toward exhaustion and away from proliferation/effectiveness (Fig. 3h). Flow cytometry verified that EV-CLDN18.2/pro-CLDN18.2 exhausted all CAR-T cell strains (Fig. 3i, j and Supplementary Fig. 14j, k), correlating with reduced alive CAR-T cell proportions (Fig. 3k). Using an in vitro cytotoxicity assay, we verified that E:T ratios of 1:1 and 3:1 were sufficient to support CLDN18.2-CAR-T-mediated cytotoxicity against CLDN18.2-overexpressing HGC27 and AGS cells (Supplementary Fig. 14l–m). Furthermore, both pre-culturing and co-culturing CAR-T cells with EV-CLDN18.2 significantly impaired their cytotoxic activity (Fig. 3l). This impairment was comparable to that observed when CAR-T cells were subjected to four consecutive rounds of rechallenge with CLDN18.2-overexpressing cancer cells (Fig. 3m). Additionally, we proved through a real-time cytotoxic killing assay that EV-CLDN18.2 interferes with CAR-T’s cytotoxicity in a dose-dependent manner (Fig. 3n).

From validating cohort, we collected PBMC samples from five patients at baseline/day 3/day 7 of CLDN18.2-CAR-T infusion for single-cell transcriptomic sequencing (Fig. 3o). Applying the same annotation criteria as CLDN18.2-CAR-T cells (Supplementary Fig. 14n–p), we calculated exhaustion, proliferation, cytotoxicity scores for each sample (Fig. 3p) and assessed paired plasma CPP-score with EV expression-array. Among the five patients, only patient No.5 (possessing the lowest baseline-CPP-score) displayed low exhaustion score, relatively high proliferation/cytotoxicity score dynamically across baseline/day 3/day 7, and finally reached PR (Fig. 3q). These data corroborated our previous findings that high-EV-CLDN18.2 patients have fewer CAR-T cells and receive poorer benefits from CLDN18.2-CAR-T therapy, proving that EV-CLDN18.2 overwhelms and exhausts CLDN18.2-CAR-T cells, finally impairs CAR-T cells’ proliferation & cytotoxicity.

### EV-CLDN18.2-stimulated activation of an IL-6-expressing CAF linage compromises CLDN18.2-targeted-CAR-T’s efficacy

**Fig. 4:**
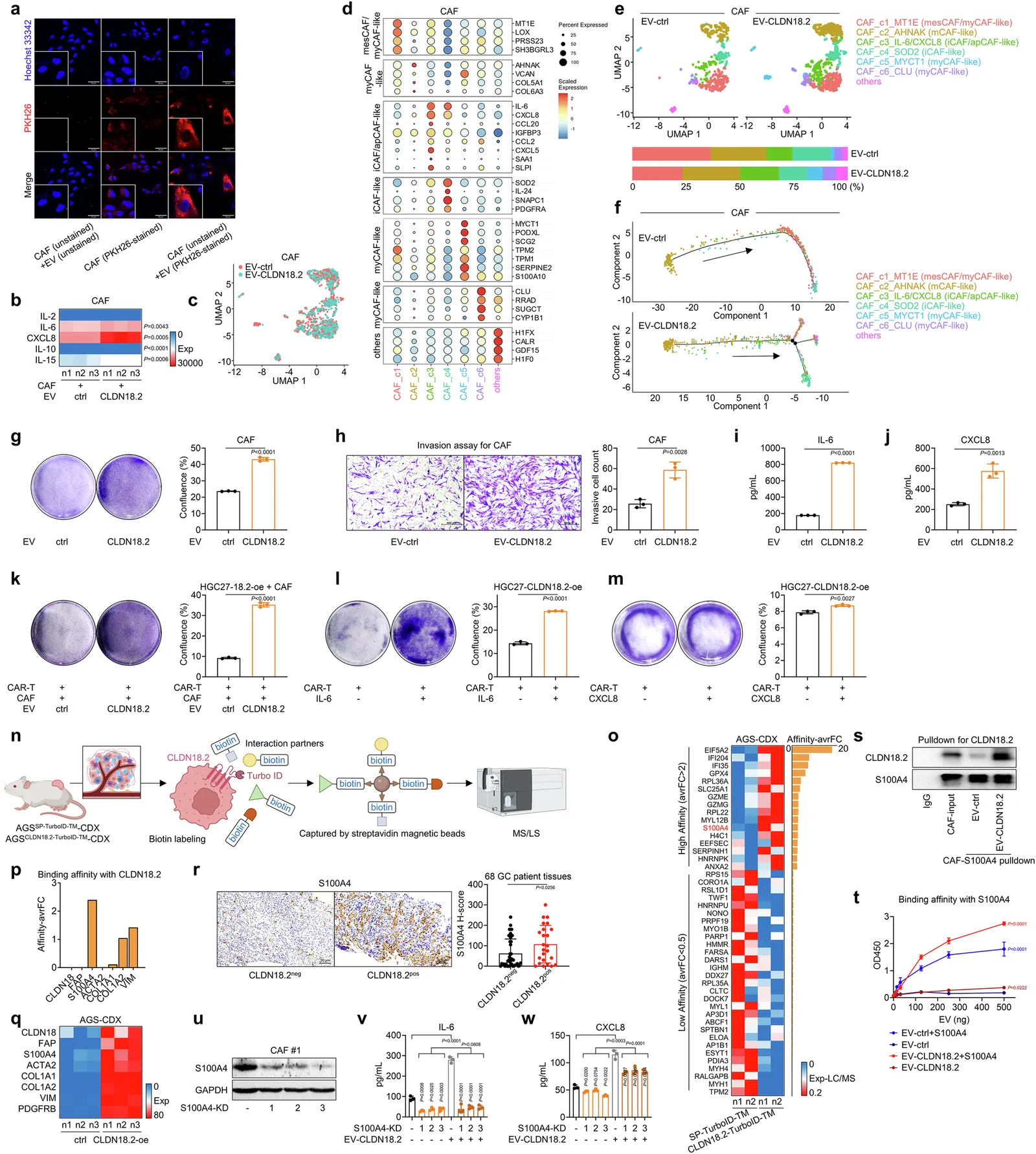
EV-CLDN18.2-induced activation of IL-6-expressing CAFs compromises the efficacy of CLDN18.2-targeted-CAR-T cells. **a** Fluorescent imaging for the uptake of PKH26-stained EVs by Hoechest 33342-stained CAFs. Images show one typical field from two representative experiments. **b** Bulk RNA-sequencing for CAF-related cytokines following treatment with EV-control or EV-CLDN18.2 (calculated from *n* = 3 independent cell-culture experiments using patient-derived CAFs, two-sided *t*test). **c** UMAP for CAF-single cells from EV-ctrl/EV-CLDN18.2 treated groups. **d** Annotations for CAFs’ clustering. **e** Featured clusters of CAF linages and their distribution before/after EV-CLDN18.2 treatment. **f** Trajectory analysis for CAF linages. EV-CLDN18.2’s influence on **g** CAF proliferation, **h** CAF invasiveness, **i** CAF-secreted IL-6 and **j** CAF-secreted CXCL8 were assessed (mean ± SD, calculated from *n* = 3 cell-culture experiments using patient-derived CAFs, two-sided *t*test). **k** EV-CLDN18.2 influenced CLDN18.2-targeted-CAR-T’s cytotoxic efficacy against CLDN18.2-overexpressing-HGC27 cancer cells mixed with CAFs (mean ± SD, calculated from *n* = 3 independent co-culture experiments, two-sided *t*test). Influence of **l** IL-6 and **m** CXCL8 on CLDN18.2-targeted-CAR-T’s efficacy against CLDN18.2-overexpressing-HGC27 were assessed (mean ± SD, calculated from *n* = 3 independent co-culture experiments, two-sided *t*test). **n** An in vivo proximity labeling assay for AGS xenografts overexpressing CLDN18.2-TurboID-TM/SP-TurboID-TM, which identifies CLDN18.2’s interacting proteins. **o** LC-MS identified protein’s expressional fold changes between CLDN18.2-TurboID-TM and SP-TurboID-TM groups were calculated to represent their interaction affinity with CLDN18.2. Proteins harboring >2/ < 0.5 foldchanges of CLDN18.2-TurboID-TM vs SP-TurboID-TM were listed. **p** Key CAF markers’ interaction affinity with CLDN18.2. **o**,**p** were based on *n* = 2 independent CDX tumors per group. **q** Bulk RNA-sequencing for CAF markers in CLDN18.2-overexpressing-AGS-CDX tissue. Data were obtained from *n* = 3 independent CDX tumors per group. **r** S100A4’s IHC staining in tissue of 68 CLDN18.2-negative/positive GC patients (mean ± SD, neg/pos *n* = 44/24, two-sided *t*test). **s** An in vitro assay pulling-down EV-ctrl/EV-CLDN18.2 with CAF-derived S100A4. **t** An in vitro assay measuring the binding affinity between EV-CLDN18.2 and purified S100A4 protein (mean ± SD, calculated from *n* = 3 independent plate-based binding-affinity experiments, two-way repeated-measures ANOVA). **u** Western blot for S100A4 knockdown in isolated CAFs. Measurement for **v** IL-6 and **w** CXCL8 secretion in CAFs after S100A4 knockdown, with or without EV-CLDN18.2 treatment (mean ± SD, calculated from *n* = 3 cell-culture experiments using patient-derived CAFs, two-sided t-test). Exact two-sided *P* values for all statistical comparisons are shown in the corresponding panels.

Given the elevated IL-6/CXCL8 levels in high CPP-score patients (Fig. 2i), we hypothesized EV-CLDN18.2 activates CAFs. We isolated CAFs from GC patients’ ascites (Supplementary Fig. 15a-15b) and verified CAFs uptake EVs (Fig. 4a). Bulk RNA-sequencing revealed that IL-6/CXCL8 were upregulated in CAFs treated with EV-CLDN18.2 for 48 h (Fig. 4b). Using single-cell transcriptomic sequencing, we clustered CAFs into linages (Fig. 4c) following representative annotations (Fig. 4d). EV-CLDN18.2 increased the proportion of an IL-6/CXCL8 expressing CAF linage (Fig. 4e and Supplementary Fig. 15c), which was also confirmed by trajectory analysis (Fig. 4f). EV-CLDN18.2 treatment enhanced CAF’s in vitro proliferation (Fig. 4g), invasiveness (Fig. 4h), IL-6 (Fig. 4i) and CXCL8 (Fig. 4j) secretion. These changes of IL-6/CXCL8 aligned with our findings in CLDN18.2-CAR-T patients (Fig. 2i). In a system where CLDN18.2-overexpressing HGC27 cells were mixed with CAFs, EV-CLDN18.2 reduced CLDN18.2-CAR-T cytotoxicity (Fig. 4k), emphasizing EV-CLDN18.2 amplified CAF’s interference on CAR-T’s efficacy. To investigate the function of the IL-6/CXCL8-expressing CAF linage, we evaluated IL-6/CXCL8’s impact on CAR-T’s cytotoxicity and found pretreatment of IL-6 (Fig. 4l), rather than CXCL8 (Fig. 4m), repressed CLDN18.2-CAR-T’s inhibition on CLDN18.2-overexpressing HGC27, highlighting a more important role of IL-6 in compromising CAR-T’s therapeutic activity.

We then explored the association between CLDN18.2 and CAFs. After establishing subcutaneous CDX models with AGS cells overexpressing CLDN18.2-TurboID-TM/SP-TurboID-TM, we performed in vivo proximity labeling assay to enrich biotinylated xenograft proteins (Fig. 4n). Through liquid chromatography-tandem mass spectrometry (LC-MS) assay, identified protein’s expressional fold changes between CLDN18.2-TurboID-TM and SP-TurboID-TM groups were calculated to represent their interaction affinity with CLDN18.2 (Supplementary Dataset 7). By listing proteins harboring >2/ < 0.5 fold changes of CLDN18.2-TurboID-TM vs SP-TurboID-TM, we noticed that S100A4 has a high interaction affinity with CLDN18.2 (Fig. 4o), while immune precipitation by our recent study verified a tangible interaction between CLDN18.2 and S100A4^16^. Among key CAF markers, S100A4 displayed the highest interaction affinity with CLDN18.2 (Fig. 4p). Subsequent bulk RNA-sequencing for AGS xenograft displayed enriched key CAF marker genes after CLDN18.2 overexpression (Fig. 4q), while IHC-staining in 68 patients’ tissues verified S100A4^+^ CAFs’ accumulation in CLDN18.2-positive GC’s TIME (Fig. 4r). Additionally, we verified a strong interaction between EV-CLDN18.2 and S100A4 through a pulldown assay (Fig. 4s) and a binding affinity assay (Fig. 4t), suggesting EV-CLDN18.2 facilitates CAFs recruitment through interacting with S100A4. Moreover, S100A4 knockdown (Fig. 4u) reduced IL-6 level but showed less pronounced impact on CXCL8 level. Notably, EV-CLDN18.2 failed to rescue the reduction of IL-6 caused by S100A4 knockdown, but retained its function to restore CXCL8 secretion (Fig. 4v, w), suggesting that EV-CLDN18.2 compromises the efficacy of CLDN18.2-targeted CAR-T cells by stimulating an IL-6–expressing CAF lineage in a S100A4-dependent manner, whereas its effect on CXCL8 expression appears to be less dependent on S100A4. Additionally, EV-CLDN18.2 also increased the proportion of two myCAF-like linages (Fig. 4e and Supplementary Fig. 15d, e), but their specific impacts on CLDN18.2-CAR-T require further investigation.

### EV-CLDN18.2 impairs the in vivo efficacy of CLDN18.2-targeted-CAR-T therapy through exhausting CAR-T cells and activating CAFs

**Fig. 5:**
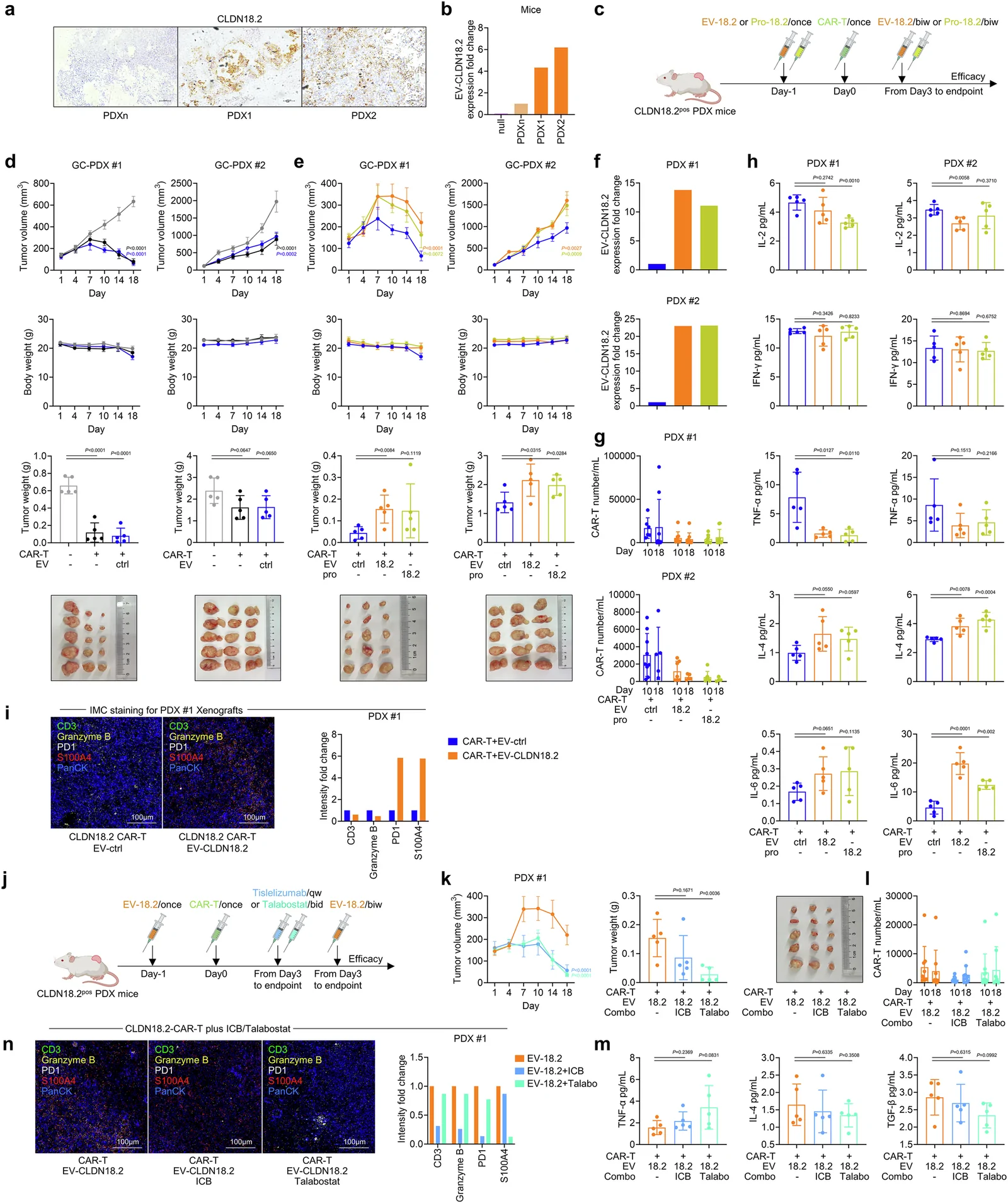
EV-CLDN18.2 impairs the efficacy of CLDN18.2-targeted-CAR-T therapy through inducing CAR-T exhaustion and CAF activation. **a** IHC-assessed CLDN18.2 expression in 3 GC PDXs’ tissue. Images show one typical field from one representative experiment. **b** EV expression-array-assessed EV-CLDN18.2 expression in plasma from xenograft-free mice, CLDN18.2-negative mice, CLDN18.2-positive PDX mice. **c** A schematic model for EV-CLDN18.2/pro-CLDN18.2’s influence on CLDN18.2-targeted-CAR-T cells’ efficacy in PDXs. **d** CLDN18.2-targeted-CAR-T’s efficacy against two CLDN18.2-positive GC PDXs (mean ± SD, calculated from *n* = 5 independent mice per treatment group for each PDX model.). **(e)** EV-CLDN18.2/pro-CLDN18.2’s impact on CLDN18.2-CAR-T’s efficacy against PDXs (mean ± SD, calculated from *n* = 5 independent mice per treatment group for each PDX model.). **f** EV-CLDN18.2/pro-CLDN18.2’s impact on EV-CLDN18.2 expression in PDX mice plasma on Day 18. **g** EV-CLDN18.2/pro-CLDN18.2’s impact on CLDN18.2-CAR-T cells’ content in PDX mice blood on Day 10/18 (mean ± SD, calculated from *n* = 5 independent mice per treatment group, with repeated blood sampling on days 10 and 18). **h** EV-CLDN18.2/pro-CLDN18.2’s impact on cytotoxic effector/immunosuppressive cytokine expressions in PDX mice plasma on Day 18 (mean ± SD, calculated from *n* = 5 independent mice per treatment group for each PDX model.). **i** IMC labeling for EV-CLDN18.2/pro-CLDN18.2’s impact on microenvironment of PDX tissue administrated with CLDN18.2-CAR-T. **j** A schematic model for Tislelizumab/Talabostat rescuing EV-CLDN18.2’s interference with CLDN18.2-targeted-CAR-T’s efficacy in PDXs. The impact of Tislelizumab/Talabostat on EV-CLDN18.2’s interference with **k** CLDN18.2-targeted-CAR-T’s efficacy (mean ± SD, calculated from *n* = 5 independent mice per treatment group), **l** CLDN18.2-targeted-CAR-T cell content in PDX plasma (Day 10/18, mean ± SD, calculated from *n* = 5 independent mice per treatment group, with repeated blood sampling from the same mice on days 10 and 18), and **m** cytotoxic effector/immunosuppressive cytokine expressions in PDX plasma (Day 18) were assessed (mean ± SD, calculated from *n* = 5 independent mice per treatment group). **n** IMC labeling for Tislelizumab/Talabostat’s impact on microenvironment of PDX tissue administrated with CLDN18.2-CAR-T/EV-CLDN18.2. Statistic diversities for **d**,**e**,**g**,**h**,**k**,**l**,**m** were assessed by Two-way repeated-measures ANOVA (curves) or two-sided t-test (bars). Exact *P* values are shown in the corresponding panels.

We evaluated EV-CLDN18.2’s in vivo impact on CLDN18.2-CAR-T therapy. As detected by EV-array in three GC patient-derived xenograft (PDX) models (Fig. 5a), CLDN18.2-positive xenograft mice harbored higher human-EV-CLDN18.2 expression in plasma than CLDN18.2-negative xenograft mice and tumor-free mice (Fig. 5b), proving EV-CLDN18.2 was derived from CLDN18.2-expressing cancer tissue. By administering PDXs with CLDN18.2-CAR-T and isolated-EVs/proteins (Fig. 5c), we found CLDN18.2-CAR-T possess high anti-tumor efficacy (Fig. 5d), while EV-CLDN18.2/pro-CLDN18.2 treatment impaired CLDN18.2-CAR-T’s efficacy (Fig. 5e), confirmed by unchanged total-EV content (Supplementary Fig. 16a) and elevated EV-CLDN18.2 expression in mice plasma (Fig. 5f). These data suggested that circulating pro-CLDN18.2 could be transferred into EV-bounded form. In concert with previous findings about EV-CLDN18.2/pro-CLDN18.2’s influence on CAR-T exhaustion/proliferation/effectiveness, EV-CLDN18.2/pro-CLDN18.2 reduced CLDN18.2-CAR-T cell numbers in PDX mice blood (Fig. 5g), downregulated cytotoxic TNF-α, while elevated immunosuppressive IL-4/IL-6 (Fig. 5h). Consistently, IMC (Imaging Mass Cytometry) staining for xenograft tissue verified that EV-CLDN18.2/pro-CLDN18.2 repelled CLDN18.2-CAR-T cells, promoted CAR-T exhaustion, reduced CAR-T effectiveness and recruited CAFs into TIME (Fig. 5i). These evidences validated that EV-CLDN18.2 impairs CLDN18.2-CAR-T’s in vivo efficacy through exhausting CAR-T cells and activating CAFs.

### Immunotherapy or CAF inhibition reverse EV-CLDN18.2’s interference on CLDN18.2-targeted-CAR-T therapy

We aimed to improve CLDN18.2-targeted-CAR-T therapy through blocking EV-CLDN18.2’s interference on TIME. Since EV-CLDN18.2 induces CAR-T exhaustion/reduction through upregulating PD1/LAG3/TIM3 and stimulates CAFs through interacting with S100A4, combining CAR-T therapy with immune checkpoint blockers (ICBs) or CAF inhibitors may enhance efficacy. Administrating anti-PD1-antibody Tislelizumab or CAF inhibitor Talabostat (Fig. 5j) reversed EV-18.2-induced repression of CLDN18.2-CAR-T efficacy in PDXs (Fig. 5k). Upon EV-CLDN18.2 administration, Tislelizumab reduced circulating CAR-T cell (presumably exhausted) number in mice blood (Fig. 5l), Talabostat reduced TGF-β (CAF stimulator) expression, and both combinations restored TNF-α expression (Fig. 5m). Similar in PDX tissue, Tislelizumab repressed EV-CLDN18.2-induced CAR-T exhaustion (represented by PD1), while Talabostat reduced EV-CLDN18.2-promoted CAF recruitment (represented by S100A4) (Fig. 5n). Mice body weight was unaffected by both administrations (Supplementary Fig. 17a).

Surprisingly, consistent with CT041-CG4006 trial’s findings^7^, combining ICBs did not increase response rate or survival proportions (Supplementary Fig. 18a, b) in CLDN18.2-CAR-T-treated patients, regardless of baseline-EV-CLDN18.2/EV-PD1/EV-PD-L2/CPP-score. Since both EV-CLDN18.2 and EV-PD1 promote CAR-T exhaustion, we inferred that exhausted CAR-T cells highly expressing PD1 are more susceptible to activation-induced cell death (AICD), a form of apoptosis triggered by the exposure of high-level PD1 to anti-PD1 antibodies^17^. Similar to the reduction of circulating CAR-T cells in PDXs (Fig. 5l), we proved that combining ICBs reduced circulating CAR-T cells in patients, regardless of baseline-EV-CLDN18.2/EV-PD1/EV-PD-L2/CPP-score (Supplementary Fig. 18c). Accordingly, scRNA sequencing or western blot confirmed that combining ICBs enhanced apoptosis features in PBMC from 4 patients (Supplementary Fig. 18d) and a GC-PDX (Supplementary Fig. 18e) receiving CLDN18.2-CAR-T therapy. These data revealed why combining anti-PD1 antibodies failed to effectively sensitize CLDN18.2-CAR-T in the CT041-CG4006 trial was due to it reducing CAR-T cells via an AICD effect. Therefore, combining CAF inhibition may be more effective in countering EV-CLDN18.2’s interference with CLDN18.2-CAR-T therapy.

### EV-CLDN18.2 reflects & neutralizes the efficacy of CLDN18.2-targeted Zolbetuximab, offering a synergistic strategy to sensitize CLDN18.2-CAR-T therapy

**Fig. 6:**
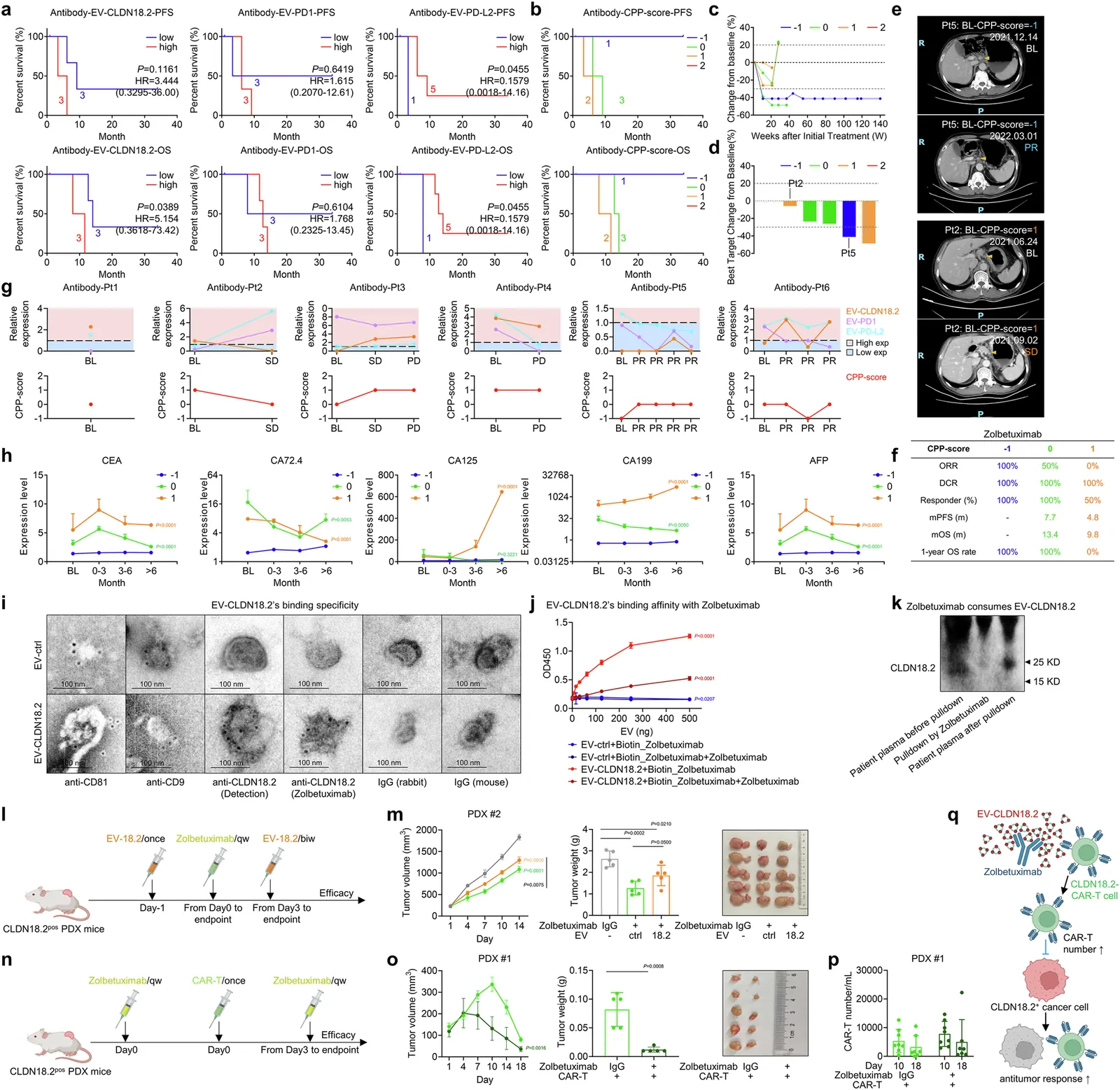
EV-CLDN18.2 reflect and influence the efficacy of Zolbetuximab, offering a synergistic strategy for therapeutic sensitization. In patients treated with CLDN18.2-targeted-antibody Zolbetuximab, the Kaplan-Meier analysis of **a** EV-CLDN18.2/EV-PD1/EV-PD-L2 (Logrank test) and **b** CPP-score for PFS/OS, and CPP-score-stratified **c** spider and **d** waterfall plots. **e** Representative CT image-series for representative patients (pt5: baseline-CPP-score = −1/best response=PR; pt2: baseline-CPP-score=2/best response=SD). **f** Major clinical outcomes of Zolbetuximab classified by baseline-CPP-score. **g** Serial changes of EV-CLDN18.2/EV-PD1/EV-PD-L2/CPP-score and response status for the 6 patients treated with Zolbetuximab+CAPOX. **h** Dynamic tumor markers classified by baseline-CPP-score in Zolbetuximab treatment (mean ± SD, −1/0/1 *n* = 1/3/2, two-way repeated-measures ANOVA). **a–h** were based on the antibody cohort, comprising *n* = 6 independent patients treated with Zolbetuximab plus CAPOX in the GLOW trial. For longitudinal analyses in (**g**) and (**h**), each patient contributed serial plasma or blood measurements at the indicated time points. **i** Immunogold labeling for the interaction of CLDN18.2-detection/treatment antibodies with EV-CLDN18.2. **j** Binding affinity of EV-CLDN18.2 with biotin-labeled Zolbetuximab (Biotin_Zol) or backfilled with intact Zolbetuximab (mean ± SD, calculated from *n* = 3 independent plate-based binding-affinity experiments, two-way repeated-measures ANOVA). **k** Immune-precipitation for EV-CLDN18.2 with Zolbetuximab in a patient plasma sample. Images show one typical blot from one representative experiment. **l** A schematic model for EV-CLDN18.2’s influence on Zolbetuximab’ efficacy in PDXs. **m** EV-CLDN18.2’s impact on Zolbetuximab’s efficacy against PDXs (mean ± SD, calculated from *n* = 5 independent mice per treatment group). **n** A schematic model for combining Zolbetuximab and CLDN18.2-targeted-CAR-T therapies in PDXs. Synergistic improvement on **o** the antitumor efficacy against PDX (mean ± SD, calculated from *n* = 5 independent mice per treatment group.) and **p** CLDN18.2-targeted-CAR-T cell content in PDX plasma on Day 10/18 (mean ± SD, calculated from *n* = 4 independent mice per treatment group, with repeated blood sampling from the same mice at the indicated time points.) were assessed. **q** A proposed mechanistic model illustrating the synergistic antitumor efficacy of combining CLDN18.2-CAR-T with Zolbetuximab. Statistic diversities for (**m,o,p**) were assessed by Two-way repeated-measures ANOVA (curves) or two-sided t-test (bars). Exact two-sided *P* values are shown in the corresponding panels.

In order to explore EV-CLDN18.2’s interference with CLDN18.2-targeted-antibody Zolbetuximab, we included all 6 patients at our center receiving Zolbetuximab+CAPOX (GLOW trial, phase 3) as an antibody cohort (Supplementary Dataset 2). Plasma EV-array analysis generated an absolute expression spectrum (Supplementary Fig. 19a, b), then it was normalized by highly correlated EV-CD81/EV-CD9 into a relative expression spectrum (Supplementary Fig. 20a). Adopting the same criteria as for CLDN18.2-CAR-T cohorts, we found baseline EV-CLDN18.2 had a negative trend, and EV-PD-L2 a positive trend with Zolbetuximab’s efficacy (Supplementary Fig. 20b and Fig. 6a). Baseline CPP-score was a stronger predictor of adverse PFS/OS than single EV proteins (Fig. 6b), indicated unfavorable tumor volume changes (Fig. 6c–e), and reflected poor clinical outcomes (Fig. 6f and Supplementary Dataset 8). EV-proteins/CPP-score dynamically tracked therapeutic efficacy (Fig. 6g), while tumor marker levels were dynamically higher in high baseline-CPP-score patients (Fig. 6h), supporting CPP-score as a potential predictor and monitor of Zolbetuximab’s efficacy in GI cancers.

Immunogold labeling demonstrated EV-CLDN18.2 could be specifically labeled by both CLDN18.2’s detection (Abcam) and treatment antibodies (Zolbetuximab) (Fig. 6i). EV-CLDN18.2 binds to biotin-labeled Zolbetuximab at high affinity, with unlabeled Zolbetuximab backfilled this binding (Fig. 6j), indicating EV-CLDN18.2 can specifically neutralize CLDN18.2-targeted-antibodies. Next, we observed that pulldown with Zolbetuximab could reduce the expression of CLDN18.2 protein in a patient plasma sample (Fig. 6k). Zolbetuximab suppressed growth/xenograft weight of tissue-CLDN18.2-positive GC-PDXs, while EV-CLDN18.2 impaired its effectiveness (Fig. 6l, m and Supplementary Fig. 17b). Moreover, synergistically applying Zolbetuximab and CLDN18.2-CAR-T (Fig. 6n) yielded enhanced therapeutic effectiveness against GC-PDX (Fig. 6o), increased circulating CAR-T cell content (Fig. 6p) and unaffected mice body weight (Supplementary Fig. 17c). These phenomena suggested that Zolbetuximab consumes EV-CLDN18.2, thereby protects CLDN18.2-CAR-T cells from exhaustion and promoting a synergistic antitumor response (Fig. 6q), which supports the potential of combining CLDN18.2-targeted-CAR-T with CLDN18.2-antibody therapies in clinical practice.

### Other CPP-score components (EV-PD1/EV-PD-L2) influence CAR-T’s efficacy by modulating TIME

**Fig. 7:**
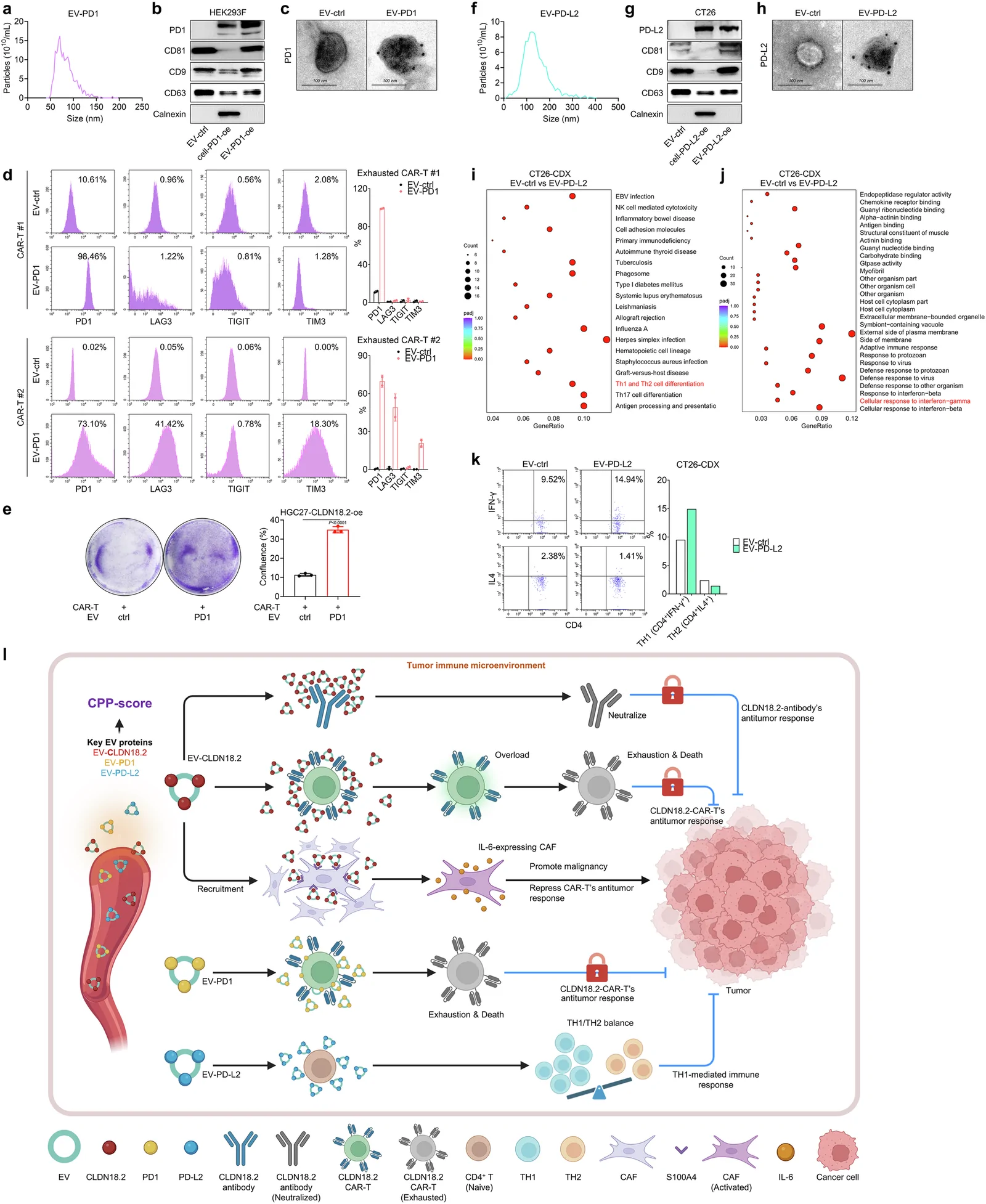
The impact of EV-PD1/EV-PD-L2 on TIME/CLDN18.2-targeted therapies. **a** NTA for size & density of EV-PD1 isolated from HEK293F. **b** Western blot for the EV markers of isolated EV-PD1. **c** Immunogold labeling assay for PD1 expression on EV-PD−1. Images show one typical field from one representative experiment. **d** Effect of EV-PD1 on the exhaustion phenotype of CLDN18.2-targeted CAR-T cells generated from healthy donors #1 and #2. Flow-cytometry plots show one representative experiment, and bar graphs summarize *n* = 2 independent CAR-T-cell culture experiments for each donor-derived CAR-T-cell preparation. Data are presented as mean ± SD. **e** EV-CLDN18.2’s influence on CLDN18.2-targeted-CAR-T’s efficacy against CLDN18.2-overexpressing-HGC27 (mean ± SD, calculated from *n* = 3 independent cytotoxicity experiments, two-sided t-test). **f** NTA for size & density of EV-PD-L2 isolated from CT26. **g** Western blot for the EV markers of isolated EV-PD-L2. **h** Immunogold labeling assay for PD-L2 expression on EV-PD-L2. Images show one typical field from one representative experiment. Bulk RNA-sequencing-based **i** KEGG and **j** GO enrichment for differentially expressed genes in CT26-xenograft treated with CT26-derived EV-PD-L2. **k** CT-26-derived EV-PD-L2’s influence on TH1/TH2 balance in CT26-xenograft tissue. **l** The roles of EV-CLDN18.2/EV-PD1/EV-PD-L2 in TIME and their influences on CLDN18.2-targeted-CAR-T/antibody therapies. Exact adjusted *P* values are provided in the corresponding panels or Source Data file.

Apart from EV-CLDN18.2, EV-PD1 and EV-PD-L2 are additional CPP-score components. We isolated EVs from a HEK293F cell line overexpressing human PD1, and ensured the expression of EV-PD1 on isolated EVs through multiple quality control steps (Fig. 7a–c). EV-PD1 mainly induced PD1-featured exhaustion, rather than LAG3/TIGIT/TIM3-featured exhaustion, in two CLDN18.2-CAR-T cell strings (Fig. 7d), suggesting EV-PD1 absorbed by CAR-T cells exacerbates exhaustion and reduces CAR-T cytotoxicity (Fig. 7e). Meanwhile, we overexpressed mouse PD-L2 in mouse colorectal cancer cell line (CT26) and isolated EVs expressing EV-PD-L2 (Figs. 7f-7h). After intratumorally injecting syngeneic CT26-CDX with mouse-EV-PD-L2, we observed enriched TH1 (T helper 1)/TH2 (T helper 2) cell differentiation (Fig. 7i) and cellular response to IFN-γ (TH1 biomarker, Fig. 7j) through RNA-sequencing in xenograft tissue. Flow cytometry confirmed EV-PD-L2 enhanced TH1 (CD4^+^IFN-γ^+^) and reduced TH2 (CD4^+^IL-4^+^) proportions in CT26 xenografts (Fig. 7k), in accordance to our previous report^18^. Since higher TH1/TH2 ratio reflected more IFN-γ secretion and higher anti-cancer immunity^19^, our work supported that EV-PD-L2 facilitates CLDN18.2-targeted-therapeutic efficacy through activating TH1-related anti-tumor immunity.

Collectively, EV-CLDN18.2 in TIME directly overloads and exhausts CLDN18.2-CAR-T cells while recruiting CAFs and promoting their differentiation into immunosuppressive subtypes, specifically an IL-6-expressing lineage, thereby impairing CAR-T’s efficacy. EV-CLDN18.2 also neutralizes CLDN18.2-targeted antibodies and reduces their anti-tumor responses. Meanwhile, EV-PD1 contributes to therapeutic resistance by exacerbating CAR-T exhaustion, whereas EV-PD-L2 enhances therapeutic efficacy by shifting the TH1/TH2 balance towards TH1, sustaining anti-tumor immunity. These distinct roles of CPP-score-related EV-proteins jointly impact CLDN18.2-targeted therapies (Fig. 7l).

### Plasma EVs depletion sensitizes CLDN18.2-targeted-CAR-T and antibody therapies

**Fig. 8:**
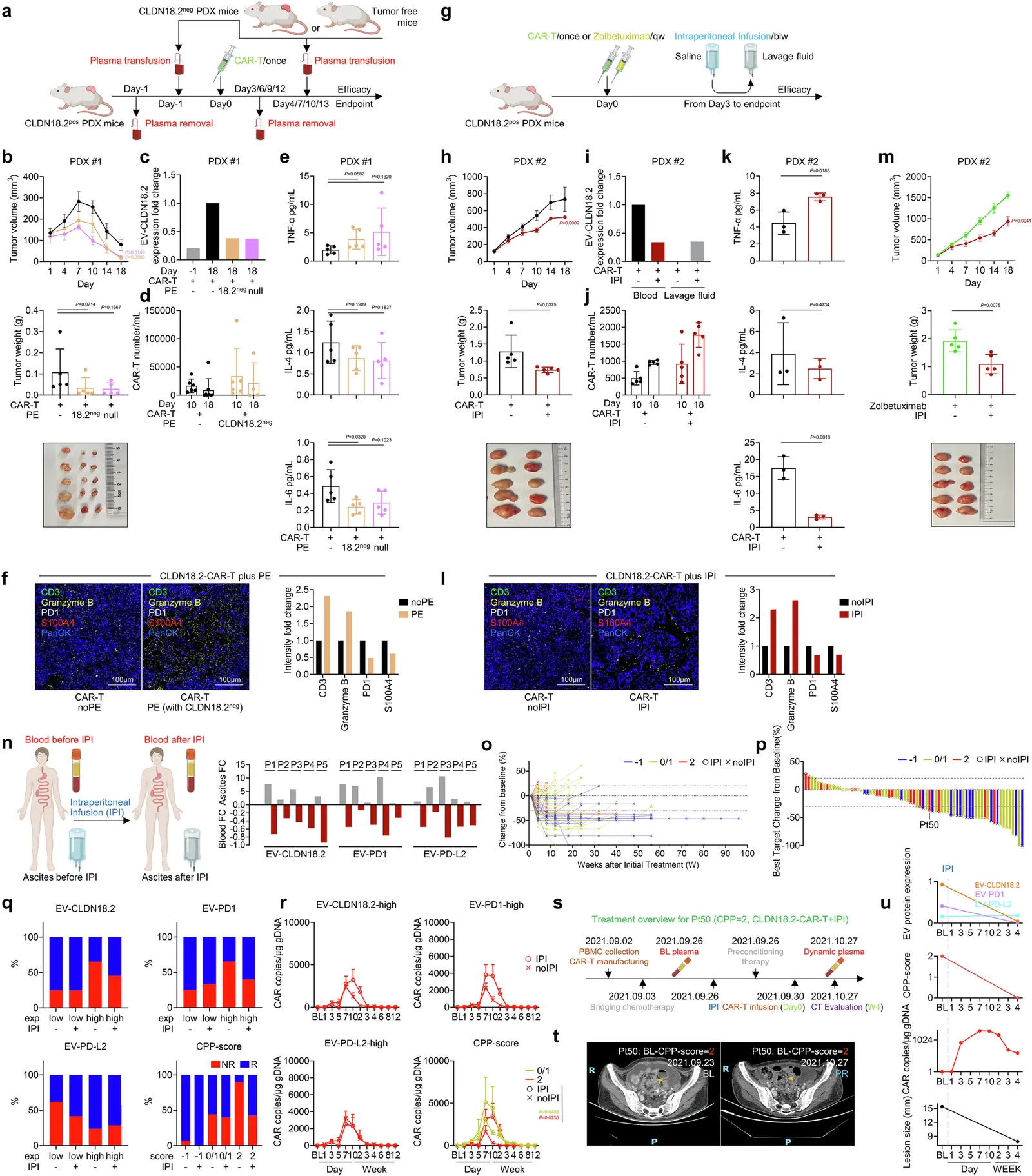
Combining plasma EVs depletion sensitizes CLDN18.2-targeted-CAR-T and antibody therapies. **a** A schematic model of plasma exchange (PE) for CLDN18.2-positive GC PDX. PE’s improvement on **b** CLDN18.2-targeted-CAR-T’s efficacy (mean ± SD, calculated from *n* = 5 independent mice per treatment group), **c** EV-array-quantified EV-CLDN18.2 expression in PDX plasma on Day −1 and Day 18, **d** CLDN18.2-targeted-CAR-T cell content in PDX blood on Day 10 and Day 18 (mean ± SD, n denotes independent mice, and the exact sample sizes at each time point are indicated in the corresponding panel.), and **e** cytotoxic effector/immunosuppressive cytokine expressions in PDX plasma on Day 18 (mean ± SD, calculated from *n* = 5 independent mice per treatment group) were assessed. **f** IMC labeling for PE’s impact on microenvironment of xenograft tissue. Images show one typical field from one representative experiment. **g** A schematic model of intraperitoneal infusion (IPI) for CLDN18.2-positive GC PDX. IPI’s improvement on **h** CLDN18.2-targeted-CAR-T’s efficacy (mean ± SD, calculated from *n* = 5 independent mice per treatment group), **i** EV-CLDN18.2 in PDX plasma on Day 18, **j** CLDN18.2-targeted-CAR-T cell content in PDX plasma on Day 10/18 (mean ± SD, calculated from *n* = 5 independent mice per treatment group, with repeated blood sampling at the indicated time points), and **k** cytotoxic effector/immunosuppressive cytokine expressions in PDX plasma on Day 18 (mean ± SD, calculated from *n* = 3 independent mice per treatment group) were assessed. **l** IMC labeling for IPI’s impact on microenvironment of xenograft tissue. Images show one typical field from one representative experiment. **m** IPI’s improvement on the efficacy of CLDN18.2-targeted-antibody Zolbetuximab (mean ± SD, calculated from *n* = 5 independent mice per treatment group). **n** A schematic chart for IPI in patients harboring ascites, and expression fold changes of key EV-proteins in plasma or ascites of 5 patients received IPI. IPI-stratified **o** spider and **p** waterfall plot for all 77 patients received CLDN18.2-CAR-T treatment. **q** Response rates stratified by EV-proteins/CPP-score and IPI for all patients. **r** Dynamic CAR-T cell content stratified by IPI for patients with high EV-proteins (mean ± SD, low/high *n* = 10/8 for EV-CLDN18.2, 11/7 for EV-PD1, 13/4 for EV-PD-L2)/CPP-score (mean ± SD, 0−1_noIPI/2_noIPI/0−1_IPI/2_IPI *n* = 16/4/7/6). n denotes independent patients with available serial blood measurements. **s** Overview of IPI and CLDN18.2-CAR-T treatment for Pt50 (baseline-CPP-score=2). **t** Representative CT image-series for Pt50. **u** Serial changes of EV-CLDN18.2/EV-PD1/EV-PD-L2/CPP-score/CAR-T cell copy/lesion size for Pt50. Statistic diversities for (**b**,**d**,**e**,**h**,**j**,**k**,**m**,**r)** were assessed by Two-way repeated-measures ANOVA (curves) or two-sided t-test (bars). Exact *P* values are shown in the corresponding panels.

Given EV-CLDN18.2’s interference with CLDN18.2-CAR-T/antibody therapies, depleting it from the blood should enhance therapeutic effectiveness. Considering the cost & difficulty to deplete specific EV-proteins, indiscriminately removing or diluting all plasma EV-proteins is more feasible for clinical practice. Through establishing a syngeneic plasma exchange (PE) model with PDXs (Fig. 8a), we found exchanging the plasma from tissue-CLDN18.2-negative PDX or tumor-free mice improved CLDN18.2-CAR-T’s efficacy (Fig. 8b) by depleting human-EV-CLDN18.2 in tissue-CLDN18.2-positive PDX mice plasma (Fig. 8c) while unaffecting total-EV content (Supplementary Fig. 16b), thereby increased circulating CLDN18.2-CAR-T cell contents (Fig. 8d), upregulated TNF-α and reduced IL-4/IL-6 in mice blood (Fig. 8e). IMC staining confirmed that PE increased CAR-T intensity/effectiveness while reduced CAR-T exhaustion/CAF recruitment in xenograft tissue (Fig. 8f). Additionally, we designed an intraperitoneal infusion (IPI) model based on PDXs (Fig. 8g), which also enhanced CLDN18.2-CAR-T’s efficacy (Fig. 8h) by washing human-EV-CLDN18.2 out from blood into lavage fluid (Fig. 8i) while unaffecting total-EV content (Supplementary Fig. 16c), thereby increased circulating CAR-T cells (Fig. 8j), upregulated TNF-α and reduced IL-4/IL-6 (Fig. 8k) in blood. In xenograft tissue, IPI increased CAR-T intensity/effectiveness and reduced CAR-T exhaustion/CAF recruitment (Fig. 8l). Additionally, IPI also enhanced CLDN18.2-antibody Zolbetuximab’s effectiveness (Fig. 8m). Both PE/IPI displayed minimal influence on mice body weight, suggesting acceptable safety (Supplementary Fig. 17d–f).

Given IPI’s stronger feasibility/eligibility than PE, we evaluated IPI in clinical practices. In 5 peritoneally metastatic GC patients with ascites, IPI depleted blood EV-CLDN18.2/EV-PD1/EV-PD-L2 by washing them into lavage ascites (Fig. 8n) while unaffecting total-plasma EV content (Supplementary Fig. 16d). Reviewing 77 CLDN18.2-CAR-T-treated patients, we found among the patients harboring baseline-CPP-score=2 (low therapeutic expectancy) but reached Partial Response, 80% (4/5) cases received IPI before/during CAR-T treatment (Fig. 8o, p). IPI improved response rate (Fig. 8q) and maintained circulating CLDN18.2-CAR-T cell counts (Fig. 8r), even in high baseline-EV-CLDN18.2/EV-PD1/CPP-score patients. Meanwhile, IPI does not reduce the response rate and circulating CLDN18.2-CAR-T cells in high baseline-EV-PD-L2 patients. Representatively, a patient (baseline: high-EV-CLDN18.2/high-EV-PD1/low-EV-PD-L2/CPP-score=2) received IPI before CLDN18.2-CAR-T infusion (Fig. 8s), who achieved Partial Response at first evaluation (Fig. 8t), along with EV-CLDN18.2/EV-PD1/CPP-score reduction, circulating CLDN18.2-CAR-T maintenance, and tumor shrinkage (Fig. 8u). These PDX and patient data suggested plasma EVs depletion as a promising strategy to sensitize CLDN18.2-CAR-T and antibody therapies.

## Discussion

Although CLDN18.2-targeted CAR-T cell/antibody therapies hold great promise for gastrointestinal cancers, challenges remain in identifying biomarkers for long-term benefit and improving therapeutic efficacy. EVs facilitate the targeted delivery of cargo throughout TIME, regulating intercellular interactions^20^. Tumor-derived EVs promote cancer progression and T cell dysfunction, threatening anti-tumor immunity^21^, while lymphocyte-derived EVs stimulate T cell proliferation, inhibit Treg phenotypes and prime anti-tumor immunity^22,23^. Considering the dynamic nature of EV cargos during cancer treatment, plasma EVs may provide crucial insights for CLDN18.2-targeted therapies.

In this study, we analyzed dynamic plasma samples from GI cancer patients receiving CLDN18.2-targeted-CAR-T (CT041-CG4006 trial) and CLDN18.2-targeted-Zolbetuximab (GLOW trial) therapies. Employing EV expression-array, a highly effective method for quantifying low-abundance proteins in small-volume plasma, we measured the dynamic expression spectrum of crucial EV-proteins, including TIME cell markers, immune checkpoint molecules and druggable cancer targets. EV-CLDN18.2/EV-PD1 correlated with poor, while EV-PD-L2 correlated with favorable therapeutic efficacy. These EV-proteins were integrated into the CPP-score, an optimized signature that may predict poor therapeutic efficacy of both CLDN18.2-CAR-T cell and CLDN18.2-antibody therapies in GI cancers. Notably, patients’ plasma EV-protein expressions did not correlate with tissue/cell counterparts, confirming differences between plasma EV-based and tissue-based TIME^18^, but EV-CLDN18.2 was higher in CLDN18.2-positive PDX mice. This discrepancy between patients and mice could be due to the fact that both tumor and normal epithelial cells contribute to the production of human plasma EV-CLDN18.2, whereas in PDXs, only engrafted CLDN18.2+ tumor cells contribute to the mice plasma EV-CLDN18.2. However, the various cell types expressing PD1 and PD-L2 leave the sources of plasma EV-PD1/EV-PD-L2 unclear. Interestingly, we found that plasma EV-protein expression and CPP-score were paired with MYC amplification in ctDNA. As an important oncogene, MYC promoted the production & release of EVs by cancer cells and leads to switched content of EV cargos^24,25^, probably through regulating the ESCRT pathway^26^. Consequently, whether MYC or other upstream regulators governing the production & release of key-EV-proteins remains to be investigated in the future.

Beyond biomarkers, EV-CLDN18.2/EV-PD1/EV-PD-L2 play crucial roles in modulating TIME and influencing CLDN18.2-targeted therapies. CAR-T cell exhaustion, a critical event induced by immune checkpoints (e.g., PD1/LAG3/TIM3/TIGIT), slows proliferation, increases apoptosis, diminishes antigen recognition and impairs tumor-killing effects, eventually limiting long-term therapeutic benefits from CAR-T^27,28^. It has been reported that EVs highly expressing PD-L1 induce CD19-targeted-CAR-T exhaustion^29,30^; mesothelin^+^PD-L1^+^ EVs inhibited the function of cognate CAR-T cells^31^; while CAR-T-derived EVs’ release of tumor antigens or immunostimulatory RNA reduces CAR-T exhaustion^32^. Nonetheless, EV’s influence on CLDN18.2-CAR-T’s exhaustion and efficacy remains unclear. Our work reported that EV-CLDN18.2 directly induces CLDN18.2-CAR-T exhaustion, therefore reducing CAR-T’s content and antitumor effectiveness. EV-PD1 also induces a PD1-related CAR-T exhaustion, strengthening the suppression of cytotoxicity. Regarding CLDN18.2-antibody therapy, EV-CLDN18.2 specifically binds and neutralizes Zolbetuximab’s antitumor effectiveness, while Zolbetuximab in turn consumes CLDN18.2 in plasma. Similar evidences have been reported that exosomal-HER2 interact with and countering HER2-targeted-Trastuzumab’s therapy^33^. In contrast, EV-PD-L2 appears to enhance the efficacy of CLDN18.2-targeted therapies by promoting a shift towards a TH1 immune response, thereby enhancing immune activation and anti-tumor immunity^18,34^.

EV-proteins also affect cancer-associated fibroblasts (CAFs), the most abundant stromal cells in TIME that closely related to malignancy^35^. Through reshaping extracellular matrix and secreting cancer-promoting cytokines/chemokines/growth factors^36^, CAF strongly supports tumor proliferation, migration/invasion, angiogenesis, drug resistance, and immune escape^37^. We found EV-CLDN18.2 interacts with CAF-harbored S100A4, recruiting CAFs into tumors and stimulating their differentiation into an IL-6/CXCL8-expressing lineage. IL-6 plays pro-inflammatory and tumor-promoting roles in TIME^38,39^. CAF-secreted IL-6 reduces CD8^+^ T’s while increases FoxP3^+^ T’s infiltration in TIME^40^, highlighting inhibiting IL-6’s potential to sensitize ICBs and cell therapies^41^. For ICBs, large cohort investigations exhibited plasma/serum IL-6 as an unfavorable therapeutic biomarker in multiple cancer types^42–44^, while preclinical study showed xenografts with IL-6R-deficient CD8^+^ T responded better to ICB^45^. For cell therapy, it was reported that knocking down IL-6R enhanced EGFR- and B7H3-CAR-T’s activity against cholangiocarcinoma^46^. In accordance to these reports, our work unveiled that EV-CLDN18.2 drives CAF’s secretion of IL-6, impairing CLDN18.2-CAR-T’s efficacy in a S100A4-dependent manner. Oppositely, while CXCL8 also implicated poor immunotherapeutic outcomes^47^ and EV-CLDN18.2 also stimulates CAF-secreted CXCL8, its influence on CLDN18.2-CAR-T efficacy was less pronounced, highlighting IL-6 as a more critical factor. We also found EV-CLDN18.2 stimulated the differentiation of myCAF-like linages (c5/c6, Fig. 4E). Specific myCAF subsets in pancreatic cancer impede ICBs by inducing CD8^+^ T cell exhaustion^48^, while in breast cancer caused CD8^+^ T’s marginalization, CD4^+^ T’s exhaustion and Treg’s infiltration^49^. Referring to these reports, we speculate EV-CLDN18.2-stimulated-myCAFs can also suppress CLDN18.2-CAR-T cells. Finally, we observed no hint about EV-PD1/EV-PD-L2’s direct relevance with CAFs or anti-CLDN18.2-antibody. Taken together, the functional involvements of EV-CLDN18.2/EV-PD1/EV-PD-L2 in TIME highlighted the therapeutic indications of the CPP-score. However, at this stage, the CPP-score is presented as a reference and has not yet been validated for clinical diagnostic use. Further rigorous validation—including the incorporation of spike-in controls, assessment of inter-laboratory reproducibility, and evaluation in larger independent cohorts—will be required before considering any potential clinical applications.

We next designed management to sensitize CLDN18.2-targeted therapies. First, although combining anti-PD1-antibody reduced EV-CLDN18.2-induced CAR-T exhaustion and rescued CAR-T’s efficacy in PDXs, it also triggered activation-induced cell death of CAR-T cells, explaining why ICBs failed to further sensitize CT041-CG4006 trial’s patients^7^ and underscores the constraints of this combination in clinical practice. Second, combining CAF inhibitors suppressed EV-CLDN18.2-induced IL-6 secretion and CAF infiltration, restoring CLDN18.2-CAR-T’s effectiveness, emphasizing CAF inhibition as a promising strategy to sensitize CLDN18.2-CAR-T therapy. Third, combining anti-CLDN18.2-Zolbetuximab with CLDN18.2-CAR-T exerts synergistic effectiveness, potentially due to the fact that EV-CLDN18.2 is consumed after neutralizing Zolbetuximab and alleviates CLDN18.2-CAR-T cells’ exhaustion. On the other hand, reducing the harmful EV-proteins in plasma represents an alternative therapeutic strategy. A recent study reported that chemical inhibitors blocking EV secretion increased CAR-T’s infiltration and anti-tumor activity^31^, however, this route presents limited potential for clinical applications, as well as the possibility of off-target effects associated with the inhibitors. In this study, we found that both PE and IPI depleted key EV proteins without affecting total EV content in mice and patient plasma. Regarding PE, this could be due to the plasma of donor/recipient mice possessing similar total-EV contents. Regarding IPI, we inferred that blood cells, especially platelets, dynamically produce total-EVs and replenish the depleted total-EV-content, while EV-CLDN18.2/EV-PD1/EV-PD-L2 require a longer period for recovery. Through depleting key EV-proteins, PE/IPI restored CAR-T cells’ content/effective molecule expression, eliminated CAF’s infiltration/IL-6-expression, and sensitized CLDN18.2-targeted-CAR-T/antibody’s efficacy. Given that EV-CLDN18.2/EV-PD1 exhausts CLDN18.2-CAR-T and naturalizes Zolbetuximab, their depletion is reasonable to restore therapeutic effectiveness. Interestingly, although EV-PD-L2 is an immune activator, its reduction has minimal impact on CLDN18.2-targeted therapies, indicating the advantage of depleting harmful EV-CLDN18.2/EV-PD1 outweighs the drawbacks of depleting beneficial EV-PD-L2.

Clinically, PE reduced immune-related AEs by clearing offending ICBs/cytokines/chemokines^50^. A cohort study suggested PE potentially improved outcomes in liver-transplanted hepatocellular carcinoma^51^, while a case report also indicated PE as a therapeutic option for hepatocellular carcinoma with anti-VEGF-induced thrombotic microangiopathy^52^. Although these descriptive studies didn’t report underlying mechanisms, we speculate PE’s therapeutic relevance may involve the depletion of harmful EV-proteins. Alternatively, with stronger clinical feasibility, IPI improved CLDN18.2-CAR-T’s response rate in patients with high EV-CLDN18.2/EV-PD1/CPP-score, indicating its potential to enhance therapeutic efficacy. Given that intraperitoneal or hyperthermic intraperitoneal chemotherapy (the standard treatments for peritoneally metastatic GC) share similar physical manipulations with IPI^53,54^, we hypothesize their therapeutic effects may also relate with EV-proteins’ depletion. In future explorations, clinical trials with optimized timing and frequency are warranted to fully assess the potential of PE/IPI and other methods for EVs depletion to enhancing long-term survival of CLDN18.2-targeted therapies.

In conclusion, our work shaped a translational chain regarding CLDN18.2-targeted CAR-T and CLDN18.2-targeted antibody therapies in gastrointestinal cancers. In addition to proposing biomarkers, functions, mechanisms, and management strategies, this study also highlighted EV-proteins’ undeniable potential to influence corresponding therapeutic cells and antibodies, providing a cautionary note for the development of precision therapies in the future.

## Methods

### Ethics statement

All patient specimens and relevant clinical information used in this study were approved for research by the Institutional Ethics Committee of Peking University Cancer Hospital & Institute (2021KT79). Written informed consent was obtained from all participants. The study was conducted in accordance with the Declaration of Helsinki. All animal experiments were approved by the Animal Experimentation Ethics Committee of Peking University Cancer Hospital (EAEC 2023-14). The maximal tumor burden permitted by the ethics committee was 2000 mm³, and this limit was not exceeded in any animal during the study.

### Patients and study design

We conducted an interventional Phase 1 clinical trial of CLDN18.2-targeted-CAR-T therapy in GI cancer patients (CT041-CG4006, NCT03874897). In this study, among the 37 patients previously demonstrated by an interim report^6^, 31 cases with available plasma samples (started treatment from Aug 2019 to Jan 2021) were retrospectively recruited as a discovery cohort. Among the other 61 patients demonstrated by a final report^7^, 46 cases with available plasma (started treatment from Jan 2021 to Dec 2021) were prospectively recruited as a validating cohort. On the other hand, we participated in an interventional Phase 3 clinical trial of CLDN18.2-targeted-antibody therapy (Zolbetuximab+CAPOX) in GI cancer patients (GLOW, NCT03653507)^11^. In this study, all 6 patients enrolled by our center were retrospectively recruited as an antibody cohort. Details for CLDN18.2-CAR-T and CLDN18.2-antibody cohorts were summarized in Supplementary Fig. 1a–f and Supplementary Dataset 1-Dataset 2.

All clinical data were acquired by referring to the medical records of patients. The end point of follow-up was Feb 2023. Tissue CLDN18.2 positivity was defined as IHC + + or +++ with a percentage of ≥40%. HER2 positivity was defined as IHC + ++, or IHC + + plus amplification assayed by fluorescence in situ hybridization (FISH). CPS positivity was defined as CPS ≥ 1. MSI/MMR status was defined by DNA sequencing for microsatellite instability or assayed by immunohistochemistry for deficient mismatch repair. EBV status was defined by DNA sequencing or by IHC as EBER positive. The antitumor efficacy was measured according to Response Evaluation Criteria in Solid Tumors (RECIST) version 1.1 as complete response (CR)/partial response (PR)/stable disease (SD)/progressed disease (PD). Objective response rate (ORR), disease control rate (DCR), progression-free survival (PFS), overall survival (OS) and duration of response (DOR) were followed up. Patients who reached CR/PR, or maintained SD≥6 months, were defined as responders; others were defined as nonresponders. Progression of responders was defined as acquired resistance.

### Specimen preparation and EV expression array

The EV expression array assay is designed to detect surface-accessible proteins carried by EVs. For preparation, antibodies for a panel of key proteins were diluted to 200 μg/mL with PBS with 5% glycerol and then printed onto a 3D modified slide surface (Capital Biochip Corp, Beijing, China) in duplicate using an Arrayjet microarrayer (Roslin, UK). The list of 50 panel proteins and paired antibodies for EV expression array were provided in Supplementary Dataset 4. PBS with 5% glycerol and 100 μg/mL of biotinylated BSA were respectively used as negative/positive controls. Microarray slides were blocked with 3% BSA and incubated with 10 μL unpurified plasma sample diluted (1:10) with 90 μL wash buffer (0.05% Tween20 in PBS), then incubated with biotinylated antibodies of EV markers (anti-human-CD81/CD9/CD63, LifeSpan BioSciences, WA, USA). For detection, slides were incubated with Cy3-labeled streptavidin (Life Technologies) diluted 1:1500 in wash buffer and scanned using the GenePix 4000 A microarray scanner (Molecular Devices, CA, USA). Fluorescent signal intensity was quantified using the GenePix Pro image analysis software (Molecular Devices, CA, USA). The sensitivity, dynamic range, controls, precision, and reproducibility of the EV-array assay was validated, as described in the Results section.

### Data processing of EV expression array

The quantification of EV expression signal intensity achieved absolute expression spectrums. Each EV-protein’s absolute expression was normalized into relative expression by dividing the geometric mean of CD81 and CD9. For further analysis, normalized expression of all panel EV proteins in discovery, validating and antibody cohorts was defined as “high” or “low” by comparing with the median of a 71-case reference cohort. EV-proteins expressed higher than the reference cohort’s median were defined as “high”, while EV-proteins expressed lower than the reference cohort’s median were defined as “low”. For CPP-score, the status of “high” expression of each molecule was counted as 1, while “low” expression was counted as 0, then calculated based on the following formula: CPP-score = count (CLDN18.2^high expression^) + count (PD1^high expression^) − count (PD-L2^high expression^)]. Thus, CPP-score is an integer ranged between -1 and 2, providing a simple manner for therapeutic evaluation. The EV-array dataset for all cohorts was provided in Supplementary Dataset 5.

### cfDNA extraction and next-generation sequencing (NGS)

Plasma cell-free DNA (cfDNA) was extracted using QIAamp Circulating Nucleic Acid Kit (Qiagen, Germany). gDNA from white blood cells was extracted using DNeasy Blood & Tissue Kit (Qiagen, Germany). DNA was qualified with Nanodrop 2000 (Thermo Fisher Scientific) and quantified by Qubit 2.0 using the dsDNA HS Assay Kit (Life Technologies, USA) according to the manufacturer’s recommendations. gDNA from white blood cells were sheared into 200–400 bp fragments using Covaris S2 Sonolab (Covaris, USA), then processed along with cfDNA (30–60 ng) samples using the KAPA Hyper Prep kit (KAPA Biosystems) for library construction. An NGS panel for 30 genes’ CNVs and 100 genes’ SNVs related to solid tumors was used for hybridization enrichment. cfDNA was labeled with unique molecular identifiers (UMI), enriched using targeted hybridization capture, and sequenced on the NovaSeq 6000 platform (Illumina) for 100 bp paired-end sequencing with a mean depth of 35,000.

Quality control was performed with Trimmomatic. Raw data (fastq files) were mapped to the reference human genome hg19 using Burrows-Wheeler Aligner (BWA-mem, v0.7.12). The variant allele frequency (VAF), which reflects the altered ctDNA fraction among all cfDNA in plasma. SNVs were annotated by ANNOVAR against the following databases: dbSNP (v138), 1000 Genome, and ESP6500 (population frequency > 0.015). CNVs (gain/loss) were assessed using FACETS V0.5.6 (https://github.com/mskcc/facets).

### Cell lines & culture and gene overexpression/knockdown

Human GC cell lines AGS/HGC27, human cell line HEK293F and mouse colorectal cancer cell line CT26 were from ATCC (Manassas, VA) or National Infrastructure of Cell Line Resource (Beijing, China), and were authenticated by short tandem repeat profiling. CAFs were isolated from GC ascites. PBMC were purchased from Milestone Biotechnology (Shanghai, China). Cells were maintained in RPMI1640/DMEM (Invitrogen) medium supplemented with 10% fetal calf serum (Gibco, BRL) and 1% penicillin plus streptomycin (HyClone, Logan, UT). Cells were transfected with a lentiviral expression system (Genechem Biotech, Shanghai, China) for overexpression of human-CLDN18.2/human-PD1/mouse-PD-L2 or knockdown of human-S100A4. The sequences for S100A4 knockdown were 5’-CCCAGATAAGCAGCCCAGGAA-3’, 5’-AGCTGCTTTCCAGAAGCTGAT-3’, and 5’-GCTCAACAAGTCAGAACTAAA-3’. Lentivirus’ volume for transfection was calculated based on the formulate: volume (mL) = MOI × number inoculated of cells/viral titer (TU mL^−1^). Polybrene was used to facilitate transfection. Puromycin (InvivoGen, San Diego, CA, USA) was added to facilitate the selection of stably infected cell clones.

### Preparation of CLDN18.2-targeted-CAR-T cells

Peripheral blood mononuclear cells (PBMCs) were isolated from fresh peripheral blood samples using Ficoll density gradient centrifugation. Isolated PBMCs were enriched with CD3 magnetic beads and added to X-Vivo medium containing IL-2. The virus for CLDN18.2-targeted CAR-T was produced by referring to a published patent from CARsgen Therapeutics Co., Ltd. (WO2022/268262) and was transfected with MOI = 3. After 24 h, cells were stimulated with CD3/CD28 activators to continue cultivation and counting. The medium was changed every 48 hs to maintain a T cell concentration of 1 × 10^6^ cells/mL. CAR expression rate was detected on the 8th day for subsequent experiments.

### Quantification for the persistence of CLDN18.2-targeted-CAR-T cells

For CT041-CG4006-treated patients, 10 mL peripheral blood samples were collected for each evaluation. The persistence of CLDN18.2-targeted-CAR-T cells was evaluated by quantifying the woodchuck hepatitis post-transcriptional regulatory element region of the lentiviral transgene with qPCR (Supplementary Table 1). gDNA was extracted using the QIAamp DNA Midi Kit (Qiagen). The standard curve for transcripts’ copy number was established by the amplification of a ten-fold serially diluted linearized plasmid PSD001 between 2×10^2^ and 2×10^6^ copies. The number of transgene copies per microgram of gDNA was determined on a 7500 Fast (Thermo Fisher Scientific), and triplicated for each sample. For CT041-CG4006-treated xenograft mice, 50 μL peripheral blood samples were retro-orbitally collected for each evaluation, incubated with flow cytometry antibodies for human CD3 (300439, Biolegend) and human F(ab) (109-095-006, Jackson), then treated with 400 μL red blood cell lysis buffer (555899, BD Pharmingen) and mixed with DPBS to stop lysis. CAR-T cells were precipitated, washed and resuspended for flow cytometry counting.

### Production, isolation, and validation of specific EV-proteins

To produce EVs expressing specific EV-proteins, a human-CLDN18.2-overexpressing HEK293F cell subline, a human-PD1-overexpressing HEK293F cell subline and a mouse-PD-L2-overexpressing CT26 cell subline were cultured in medium containing exosome-free serum and proceeded for the collection of culture medium supernatant when cell density reached 80%. Culture medium was centrifuged at 300 ×g for 10 min to collect the supernatant. Supernatant was centrifugated at 2000 × g for 15 min to remove cell debris, then diluted by seven-fold volume of PBS and processed through a 0.22 μm vacuum filter to remove large particles. Supernatant was diafiltrated and concentrated using Hydrosart® TFF (tangential flow filtration) device with 300 kDa cut-off filters (item number: 3M51467901E-SW, Sartorius, NY, USA), then ultracentrifuged using a P50A72-986 rotor (CP100NX; Hitachi, Brea, CA, USA) at 100,000 ×g, 4 °C for 2 h to pellet EVs. Pelleted EVs was resuspended in PBS and centrifuged again at 100,000 ×g, 4 °C for 2 h. After PBS wash, isolated EVs was re-suspended in 100 µL PBS.

Isolated EVs were quantified with BCA method, then validated with transmission electron microscopy (TEM), nanoparticle tracking analysis (NTA) and western blot methods. For TEM assay, isolated EVs were incubated on the mesh, stained with uranyl acetate, then observed using a transmission electron microscope (H-7650, Hitachi). For NTA assay, isolated EVs were observed using the ZetaView PMX 110 (Particle Metrix, Meerbusch, Germany) powered by a 405 nm laser to measure particle size and quality. Of note, while NTA is widely used for estimating EV size distribution and concentration, its results are known to be sensitive to sample preparation and should not be interpreted as an indicator of absolute EV purity. For WB assay, appropriate primary antibodies were used to detect MISEV-aligned positive (CD81 (ab79559, Abcam; GTX101766, Genetex)/CD9 (ab223052, ab307085, Abcam)/CD63(ab216130, Abcam; GTX28219, Genetex)) and negative (Calnexin (ab22595, Abcam)) EV markers. For immunogold labeling assay, concentrated EVs were resuspended with 4% paraformaldehyde and absorbed by a 400-mesh carbon/formvar coated grids. The grids were blocked with 5% BSA (w/v) and incubated with primary antibodies (CD81 (sc−166029, Santa)/CD9 (sc−13118, Santa)/CLDN18.2 (NC004, Novoprotein)/Zolbetuximab (HY-P99058, MCE)/PD1 (ab52587, Abcam)/PD-L2 (14-5888-82, Invitrogen)/IgG (rabbit), IgG (mouse)) at room temperature, then incubated with secondary antibodies (anti-rabbit IgG (G7402, Sigma-Aldrich), anti-mouse IgG (G7652, Sigma-Aldrich) conjugated to 10-nm gold particles (Merck). The grids were stained with uranyl acetate and observed using TEM assay.

To isolate EVs from patient plasma, we used a kit combining chromatography and SEC-based purification (EVTMC, EVbio, China) following the manufacturer’s instructions. The kit utilizes a two-step process: Step 1 (Size Exclusion): 100 μL of plasma was loaded onto a column pre-packed with Sepharose CL-6B resin (Cytiva). After the flow-through was discarded by gravity, the EV-enriched fraction was eluted with 200 μL of PBS (Fraction 1). Step 2 (Mixed-Mode Chromatography): The eluate (Fraction 1) was then applied to a second column containing a proprietary mixture of Fractogel EMD SO3-(M) (MilliporeSigma) and Capto Core 700 (Cytiva) resins. Following a brief incubation, the purified EVs were collected in the flow-through by centrifugation at 500 × g for 30 s (Final EV Fraction).

For ELISA assay of isolated EVs, EVs were diluted to a final volume of 100 µL with coating buffer (0.1 M carbonate, pH=9.6) and incubated on ELISA plates overnight at 4 °C. Following incubation, the plates were washed with PBST and blocked with 100 µL of 5% BSA for 60 min at room temperature. The samples were then incubated with primary antibodies for CLDN18.2 (ab241330, Abcam)/PD1 (ab52587, Abcam) for 2 h at room temperature. After another round of PBST wash, the plates were incubated with a goat-anti-mouse IgG secondary antibody for 1 h at room temperature. Subsequently, 100 µL of TMB substrate solution was added and allowed to react for 30 min. The reaction was stopped by adding 100 µL of stop solution, and the optical density (OD) was immediately measured at 450 nm.

### Fluorescence uptake experiment for extracellular vesicles

After isolation by ultracentrifugation, EVs produced by HEK293F cells were stained with PKH26 (D0030, Solarbio) and re-purified using an EV purification kit (ES9016, Echobiotech), which operates based on molecular sieving and size-exclusion chromatography (SEC). A volume of 50 μL of stained EVs was re-purified according to the manufacturer’s instructions. Re-purified EVs (10 μg/mL) were then co-cultured with Hoechest 33342 (C1027, Beyotime) pre-stained CAR-T cells or CAFs (1 × 10^6^ cells/mL) for 12 h. Co-cultured cells were fixed with 4% paraformaldehyde, rinsed with PBS, detected with fluorescence labeled antibodies and observed under a fluorescence microscope.

### Western blot, immunohistochemistry (IHC), flow cytometry, and ELISA for cytokines

For western blot, freshly extracted EVs/cells were resuspended with 100 μL of RIPA lysis solution. After BCA quantification, protein samples were denaturalized with boiling loading buffer, proceeded for electrophoresis, transferred onto polyvinylidene fluoride (PVDF) membranes (Millipore, USA), probed with appropriate primary antibodies (CLDN18 (ab203563)/CD81 (ab79559)/CD9 (ab223052, ab307085)/CD63(ab216130)/Calnexin (ab22595) from Abcam, CD63 (GTX28219)/CD81 (GTX101766) from Genetex, GAPDH (#2118)/α-SMA (#19245)/FAP (#52818)/cleaved Caspase 3 (#9664)/Bcl-2 (#15071) from Cell Signaling Technology) and secondary antibodies, illuminated with Clarity Western ECL substrate (Bio-Rad, Hercules, CA) and visualized with Amersham Imager 600 (GE Healthcare, Chicago, IL).

For IHC, formalin-fixed and paraffin-embedded (FFPE) tissue slides were deparaffinized with dimethylbenzene, rehydrated with ethanol, treated with 3% H_2_O_2_ to quench endogenous peroxidase activity, processed for antigen retrieval in boiling EDTA solution (pH=9.0, ZSGB-BIO), blocked from nonspecific bindings by 5% goat serum, stained with hematoxylin-eosin (Solarbio) or CLDN18.2/S100A4 antibody-DAB combinations, and scored by separate pathologists to calculate H-score.

For flow cytometry, fresh cells were collected and washed with DPBS (Dulbecco’s Phosphate-Buffered Saline), then resuspend with DPBS into 1×10^6^ cells/mL. Ensuring a viability greater than 95%, cells were identified by CD3-FITC staining (317305, Biolegend), followed by surface staining of PD−1-PE (367404, Biolegend), TIM-3-APC (345012, Biolegend), LAG-3-PE/Cy7 (369310, Biolegend), and TIGIT-BV605 (372710, Biolegend) for the CD3^+^ population. 5 μL antibodies were diluted into 100 μL for each sample. Cells were stained at room temperature in the dark, washed with DPBS, filtered and proceeded for flow cytometry detection (CytoFlex, Beckman). The gating strategy was as follows: First gate (FSC/SSC): Forward and side scatter were adjusted to ensure that all cell populations of interest were within the view. A gate was drawn to include all viable cells. Second gate (CD3^+^ cells): Based on the negative control (unstained T cells), the CD3^+^ population was gated. Third gate (positive populations for checkpoint markers): Based on the CD3^+^ gate, voltages were adjusted to resolve positive and negative populations for each marker. Populations with fluorescence intensity greater than 10⁴ were considered positive. Flow cytometry results were analyzed using the CytoExpert software. CLDN18.2-CAR-T cells from 4 donors’ PBMC (CAR-T #1-#4) were used to assess cell exhaustion, in which 2 biological replicates were provided for the assessment of statistical significance in CAR-T #1 (two-sided t-test).

For ELISA, plasma samples or cell culture medium supernatant were diluted and measured for the expression of IFN-γ/TNF-α/IL-2/IL-4/IL-6/CXCL8/TGF-β (RK04339, Abclonal) according to the kit manufacturer’s instructions. Targeted concentrations were calculated based on the linear range of the manufacturer’s standard control. In each group, 3-4 biological replicates were provided for the assessment of statistical significance (two-sided t-test).

### Biotin-labeled antibody backfill assay

EVs were diluted with 50 mM sodium bicarbonate/carbonate buffer (pH=9.6) and incubated overnight at 4 °C on 96-well plates (Corning), then blocked with 1% BSA at room temperature. Subsequently, plate was incubated with biotin-labeled or intact CLDN18.2-antibody Zolbetuximab (HY-P99058, MCE) for 1 h, following horseradish peroxidase-conjugated streptavidin (Thermo) for another 1 h under room temperature. Finally, color development was achieved using tetramethylbenzidine (Pierce) and terminated with H_2_SO4. The absorbance value at 370 nm was measured with a BioTek plate reader. In each group, 2 biological replicates were provided for the assessment.

### Cell morphology, cytotoxic killing, real-time cytotoxic killing, and invasion assay

For the morphology and cytotoxic killing assay, CLDN18.2-overexpressing HGC27, CAFs or a mixture of equal proportions of HGC27 and CAFs were pre-cultured into 6-well plates as triplicates. CAR-T cells were added at different effector-to-target (E:T) ratios in 1 mL culturing system for 24 hours-cytotoxic killing of 3 × 10^5^ cancer cells. Specified E:T ratios of 1:1 for 3 × 10^5^ HGC27 cells, or 3:1 for 3 × 10^5^ AGS cells, were adopted for subsequent analysis with EV-proteins/purified proteins. EV-CLDN18.2 (10 μg/mL), pro-CLDN18.2 (5 μg/mL, CL2-H52P7, ACROBiosystems), EV-PD1 (10 μg/mL), IL-6 (3 μg/mL (HY-P7044AF, MCE)) and CXCL8 (2 μg/mL (HY-P700133AF, MCE)) were co-cultured or pre-cultured with cells for 24 h-cytotoxic killing.

For rechallenging of cytotoxic killing, CAR-T cells were co-cultured with CLDN18.2-overpressing HGC27 for four consecutive 24-hour rounds, then subjected to for 24 h-cytotoxic killing. Cell morphology was assessed by light microscope. The crystal blue staining for cell confluence was used to identify cell viability after cytotoxic killing.

For real-time analysis of cytotoxic killing, 1 × 10^4^ tumor cells per well were spread onto a Real-Time Cell Analysis (RTCA) plate for 24 h, then treated with different content of EVs (2/10/20 ng per well) and measured with a xCELLigence RTCA DPlus device. Normalized cell index was measured every 2 h for 72 h to draw a dynamic killing curve.

For invasion assay, cells were counted & resuspended with serum-free medium (2 × 10^5^/mL, 150 μL), inoculated in the upper chamber of each transwell (pre-coated with matrigel) (Corning, New York, NY) and cultured in complete medium for 48 h, then fixed with methanol and dyed with 0.1% crystal violet. After removing cells in the upper chamber with cotton wool, the penetrated cells were counted under the microscope.

For both cytotoxic killing and invasion assay, 3 biological replicates were provided in each group for the assessment of statistical significance (two-sided t-test).

### Imaging Mass Cytometry (IMC) staining

Tissue was cut into 4 μm thick paraffin sections and baked at 65°C before dewaxing and hydration treatment. Endogenous peroxidase was then removed, and antigen repair was performed using EDTA (pH=9.0) at room temperature. Tissues were incubated with IMC-specialized antibodies (CD3 (ab251607)/GZMB (ab255868)/PD−1 (ab201811)/S100A4 (ab220213)/PANCK (ab308263) from Abcam) overnight at 4 °C with a concentration of 1:200, washed and incubated with secondary antibodies, then stained with DAB and hematoxylin. After 1% hydrochloric acid alcohol separation, slides were subjected to flowing water reverse blue, and finally dehydrated and transparent before sealing.

### RNA sequencing

Total RNA was extracted from cells/tissues using Trizol method. Quality control was performed using an Agilent 2100 Bioanalyzer (Agilent Technologies) to ensure RNA integrity. Next generation RNA-sequencing was performed using an Illumina HiSeq instrument (Illumina, San Diego, CA).

### 10x Genomics-based single-cell RNA sequencing

CAR-T cells/PBMCs/CAFs were isolated by density gradient centrifugation using Ficoll-Paque Plus medium (GE Healthcare) and washed with Ca/Mg-free PBS, subsequently passed through a 100 µm SmartStrainer and centrifuged at 400 g, suspended in red blood cell lysis buffer (Miltenyi) to lyse red blood cells, and re-suspended in sorting buffer (PBS supplemented with 2% fetal bovine serum (FBS, Gibco)). Single cell suspensions were stained with 7AAD antibodies, sorted by FACS with a BD Melody instrument, and cell viability and concentration were determined using a Countstar Automated Cell Counter. Cells were captured in droplets to generate nanoliter-scale Gel bead in EMulsion (GEMs) and then reversely transcribed in a C1000 Touch Thermal Cycler (Bio-Rad) programmed at 53 °C for 45 min, 85 °C for 5 min, and held at 4 °C.

After reverse transcription and cell barcoding, emulsions were broken and cDNA was isolated and purified with Cleanup Mix containing DynaBeads and SPRIselect reagent (Thermo Fisher Scientific), followed by PCR amplification. For RNA-seq library construction, amplified cDNA was fragmented and end-repaired, double-sided size-selected, PCR-amplified with sample indexing primers, and double-sided size-selected. Enriched PCR product was fragmented and end-repaired, size-selected, PCR-amplified with sample-indexing primers, and size-selected. Libraries prepared according to the manufacturer’s user guide were then purified and profiled for quality assessment. Single-cell RNA libraries were sequenced by an Illumina NovaSeq 6000 sequencer with 150 bp paired-end reads.

### Processing of single-cell sequencing data

Raw gene expression matrices were generated for each sample by the Cell Ranger (Version 6.1.2) pipeline coupled with human reference version refdata-gex-GRCh38-2020-A. Filtered gene expression matrices were analyzed by R software (Version 4.1.2) with the Seurat package (Version 4.3.0.1). The percentage of mitochondrial transcripts for each cell (percent.mito) was calculated and added as metadata to the Seurat object. All samples were merged into one Seurat object using the merge function in Seurat. Data were filtered for cells (nfeatures>200) and mitochondrial genes (percent_mt<10%). After the filtering, the number of cells proceeded for analysis was 7991/10379 for EV-ctrl-CAR-T/EV-CLDN18.2-CAR-T and 470/1035 for EV-ctrl-CAF/EV-CLDN18.2-CAF groups.

Dimension reduction and unsupervised clustering were performed according to the standard workflow in Seurat. NormalizeData function was applied to normalize the single nucleus gene expression data, and we used 2000 genes with high cell-to-cell variation, which were calculated using the FindVariableFeatures function for further dimensionality reduction. Then, the effect of the percentage of mitochondrial gene count was regressed out by using the ScaleData function. A principal component analysis (PCA) matrix was calculated to reduce noise by using RunPCA, and thirty principal components were selected for downstream analysis. We performed batch correction using the Harmony R package (Version 0.1.1). Then Uniform Manifold Approximation and Projection (UMAP) and clustering were performed to identify clusters. Finally, we clustered cells using the FindNeighbors and FindClusters functions and performed non-linear dimensional reduction.

We used the FindAllMarkers function with the Wilcoxon rank-sum test to identify and annotate differentially expressed genes (DEGs). DEGs were filtered using an average log2 (fold change) of 0.25. The main cell types were annotated based on the expression pattern of DEGs and the well-known cellular markers from the literature. For trajectory inference analysis, monocle (Version 2.22.0) in R was used. Raw count data from the Seurat object was converted to a CellDataSet object using the newCellDataSet function in Monocle. DEGs between cell types (calculated using the Findallmarkers function in Seurat, sorted according to their avg_log2FC, and the top 1000 were extracted as significantly different genes) were set as the ordering genes. The minimum spanning tree was constructed using the reduceDimensions function (reduction_method = “DDRTree”).

### Proximity‑dependent biotin labeling assay

We fused and expressed biotin ligated TurboID in the extracellular segment of CLDN18.2 as the experiment group (CLDN18.2-TurboID-TM), and a plasmid with only biotin ligated TurboID as the negative control group (SP-TurboID-TM). We constructed AGS cells stably express SP-TurboID-TM or CLDN18.2-TurboID-TM with lentivirus, then subcutaneously inoculated 5×10^6^ cells into 5-week-old female BALB-C nude mice (Vital River Laboratories, Beijing, China) for xenograft formation. After two weeks, mice were intraperitoneally treated with 500 μM biotin for 60 minutes, and harvested tumor cells were lysed in RIPA buffer. Streptavidin magnetic beads were mixed with the supernatant and rotated overnight at 4 °C to attach biotinylated proteins. Biotinylated proteins were eluted in 150 mL of boiling elution buffer (55 mM pH=8.0 Tris-HCl, 0.1% SDS, 5 mM DTT and 0.5 M biotin), then assessed through liquid chromatography-tandem mass spectrometry (LC-MS/MS) analysis, with two biological replicates of CDXs assessed for each group. LC-MS data for proximity labeling assay was listed in Supplementary Dataset 7. Each protein’s expressional fold changes between CLDN18.2-TurboID-TM and SP-TurboID-TM groups were calculated to represent their interaction affinity with CLDN18.2.

Proteins harboring >2/ < 0.5 foldchanges of CLDN18.2-TurboID-TM vs SP-TurboID-TM were demonstrated for subsequent analysis. For proteins harboring >2 foldchanges of CLDN18.2-TurboID-TM vs SP-TurboID-TM, the expression value should be larger than zero and rank within the upper three quartiles (highest 75%) for at least one of CLDN18.2-TurboID-TM’s biological replicate; For proteins harboring <0.5 foldchanges of CLDN18.2-TurboID-TM vs SP-TurboID-TM, the expression value should be larger than zero and rank within the upper three quartiles (highest 75%) for at least one of SP-TurboID-TM’s biological replicate.

### Assessment of S100A4’s interaction with EV-CLDN18.2

For the in vitro pulldown assay, beads for protein G (10003D, Invitrogen) pre-bounded with anti-S100A4 antibody (16105−1-AP, Proteintech) were used to combine S100A4 protein in cell lysate of CAFs isolated from a GC patient, then we used the system to pulldown EV-ctrl or EV-CLDN18.2 isolated from HEK293F cells. Pulled-down proteins were denatured from beads with boiling loading buffer, then measured by Western Blot for CLDN18.2 and S100A4.

For the binding affinity assay, EV-ctrl/EV-CLDN8.2 isolated from HEK293F cells were immobilized overnight at 4 °C in a 96-well microplate coated with 50 mM sodium bicarbonate/carbonate buffer (pH=9.6). Following three washes with PBS containing 0.05% Tween-20 (PBST), the wells were blocked with 1% BSA for 1 h at room temperature. After additional PBST washes, immobilized EVs were incubated for 1 h at room temperature with recombinant human S100A4 protein (Ag9019, Proteintech). After PBST washing, plates were probed with anti-S100A4 primary antibody (16105-1-AP, Proteintech) and HRP-conjugated goat anti-rabbit IgG secondary antibody (32460, Thermo Fisher Scientific). After final washes, 100 μL of TMB substrate solution was added per well and incubated for 30 minutes. The reaction was stopped by adding 100 μL of stop solution per well, and absorbance was measured at 450 nm. In each group, two biological replicates were provided for the assessment of statistical significance (two-sided t-test).

### Assessment of EV-PD-L2 related impacts

CT26 cells were inoculated into 5-week-old female BALB-C mice (Vital River Laboratories) for xenograft formation. When tumor volume reaches 150 mm^3^, mice were randomly grouped and treated with CT26-derived EV-PD-L2 (200 μg) for twice per week (biw). Mice were sacrificed when tumor volume over 2000 mm^3^ or after 21 days dosing, then proceeded for the flow cytometry assessment of EV-PD-L2 related TIME changes.

### Establishment of patient-derived xenograft (PDX) models

Surgically-resected fresh tumor tissue from CLDN18.2-positive GC patients were washed with PBS and inoculated into 5-week-old female NCG mice (HFK Biotechnology, Beijing, China) to construct PDXs. Xenograft tumors were transferred to the next generation of mice when its volume reached 500 mm^3^. After 3 passages, PDXs were adopted to test tissue-CLDN18.2 positivity in xenograft tissue and EV-CLDN18.2 expression in plasma, then subjected to evaluate the effectiveness of reagents. Mice were weighed twice a week, with tumor volume calculated by the formula V = L×W^2^×0.5 (V, volume; L, length; W, width of tumor). Mice were sacrificed when tumor volume over 2000 mm^3^ or after 21 days dosing. 5 mice were ensured in each group for the assessment of statistical significance (Two-way repeated-measures ANOVA for growth curve, two-sided t-test for tumor weight, cytokine concentration and CAR-T concentration).

### Administration of CAR-T, EV-proteins and EVs depletion in PDX models

For CLDN18.2-targeted CAR-T/antibody and EV-CLDN18.2/pro-CLDN18.2 administration, xenograft mice were grouped when tumor volume reached 150 mm³. Each mouse intravenously received 5 × 10⁶ CAR-T cells for once (Day 0), or 2 mpk Zolbetuximab/IMAB362 for once per week (qw) since Day 0. EV-CLDN18.2/pro-CLDN18.2 were dissolved in PBS and administered intravenously at 200 μg/100 μg per treatment, respectively. EV-CLDN18.2/pro-CLDN18.2 were administered once at Day -1, and twice a day since Day 3.

For plasma exchange (PE) groups, tissue CLDN18.2-positive/negative xenografts were prepared. Xenograft mice were grouped when tumor volume reached 150 mm³. Each mouse intravenously received 5 × 10⁶ CLDN18.2-targeted CAR-T cells for once (Day 0). 300 μL blood extracted from a CLDN18.2-positive xenograft mouse with the retro-orbital blood collection method was centrifuged to remove plasma, then replenished with an equal volume of plasma isolated from the blood of CLDN18.2-negative xenograft mice or tumor-free mice, and transfused back into the same CLDN18.2-positive xenograft mouse through tail vein injection. PE was administered once on Day -1, and twice since Day 3.

For intraperitoneal infusion (IPI) groups, xenograft mice were grouped when tumor volume reached 150 mm³. Each mouse intravenously received 5×10⁶ CAR-T cells for once (Day 0). Intraperitoneal infusion was performed by abdominally injecting 3 mL physiological saline and removing it after 30 min. Intraperitoneal infusion was administered once on Day -1, and twice since Day 3.

For combination groups, xenograft mice were grouped when the tumor volume reached 150 mm³. Each mouse intravenously received 5 × 10⁶ CAR-T cells for once (Day 0). EV-CLDN18.2 (200μg)/pro-CLDN18.2 (10μg) were administered intravenously once on Day -1, and twice since Day 3. Tislelizumab (10 mpk, HY-P99052, MCE) was intravenously administrated qw. Talabostat (10 μg, HY-13233A, MCE) was orally administrated twice a day (bid).

5 mice were ensured in all groups mentioned above, for the assessment of statistical significance (Two-way repeated-measures ANOVA for growth curve, two-sided t-test for tumor weight, cytokine concentration and CAR-T concentration).

### Statistics, reproducibility, and formatting

Subgroup diversities was assessed by chi-square test/unpaired two-tailed two-sided t-test/two-way repeated-measures ANOVA. Consistency between indexes was assessed by Pearson correlation analysis. Survival proportions were assessed by Kaplan–Meier analysis paired with Log-rank test. A two-sided *P* < 0.05 was considered statistically significant. Error bars were exhibited in form of mean ± standard deviation (SD). Analysis and graphing were powered by R 3.5.1/SPSS 21.0/GraphPad Prism 8/BioRender programs.

### Reporting summary

Further information on research design is available in the Nature Portfolio Reporting Summary linked to this article.

## Supplementary information

Supplementary Information

Description of Additional Supplementary Files

Supplementary Dataset

Reporting Summary

Transparent Peer Review file

## Data Availability

The single-cell RNA-sequencing data generated in this study have been deposited in the National Genomics Data Center, China National Center for Bioinformation, under BioProject accession PRJCA024021 and Genome Sequence Archive for Human accession HRA006971. All other data generated or analyzed during this study are included in this article and its Supplementary Information. Source data are provided with this paper.
