# Supplementary material for: Extracellular vesicle proteins monitor and interfere with CLDN18.2-targeted CAR-T cell and antibody therapies against gastrointestinal cancers
Source: Nat Commun. 2026 Sep 3;17:10502. doi: 10.1038/s41467-026-77132-9 (PMC13638995; doi:10.1038/s41467-026-77132-9)
Supplement: Supplementary file 1 [file 41467_2026_77132_MOESM1_ESM.pdf]

Supplementary Figures

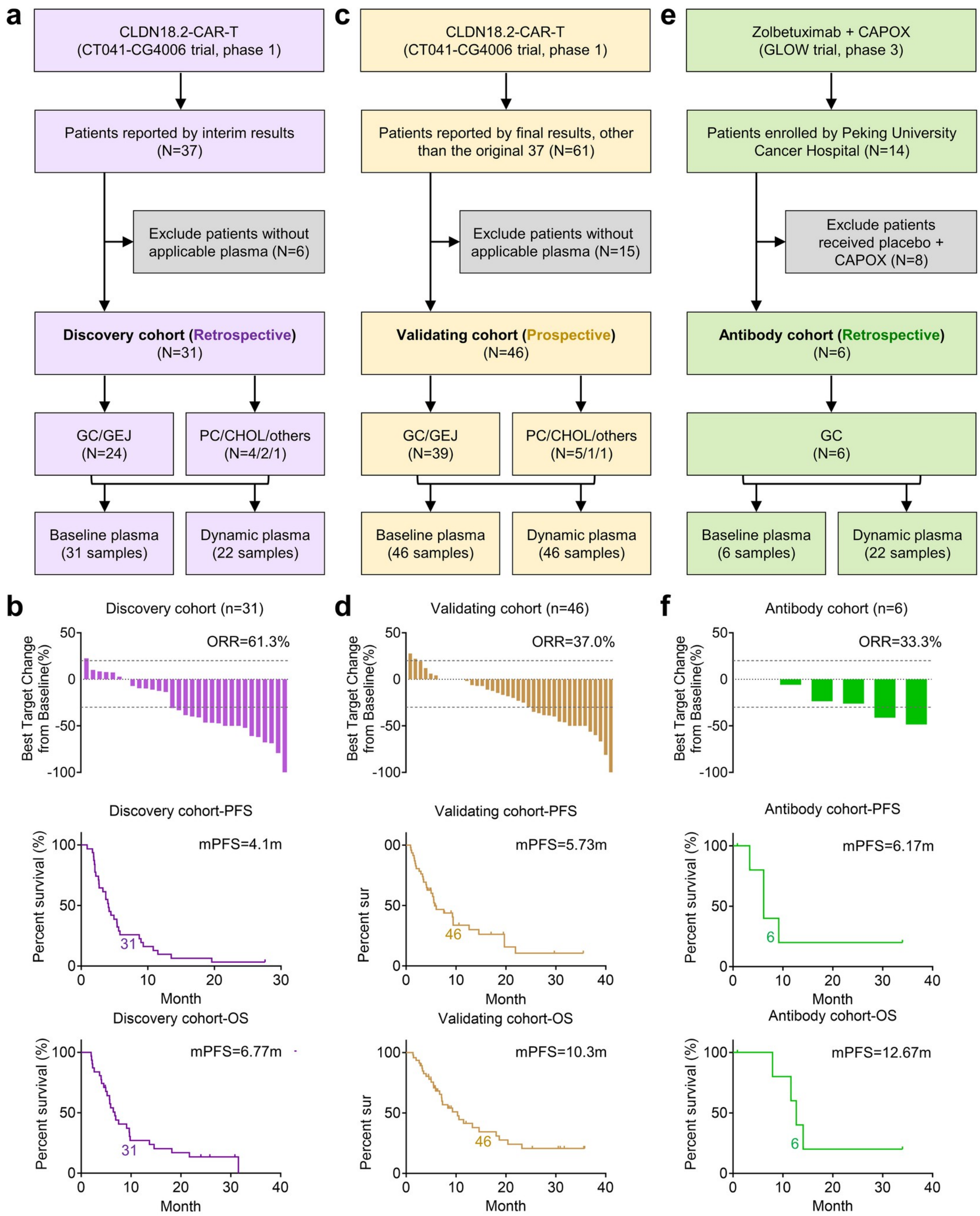

Supplementary Fig. 1. An overview for patient cohorts of CLDN18.2-targeted-CAR-T/antibody therapies used in this study. (a) Recruitment of CLDN18.2-CAR-T's discovery cohort. **(b)** Clinical features of discovery cohort. **(c)** Recruitment of CLDN18.2-CAR-T's validating cohort. **(d)** Clinical features of validating cohort. **(e)** Recruitment of CLDN18.2-antibody cohort. **(f)** Clinical features of antibody cohort. n denotes biologically independent patients. The discovery, validating and antibody

cohorts comprised n = 31, n = 46 and n = 6 independent patients, respectively.

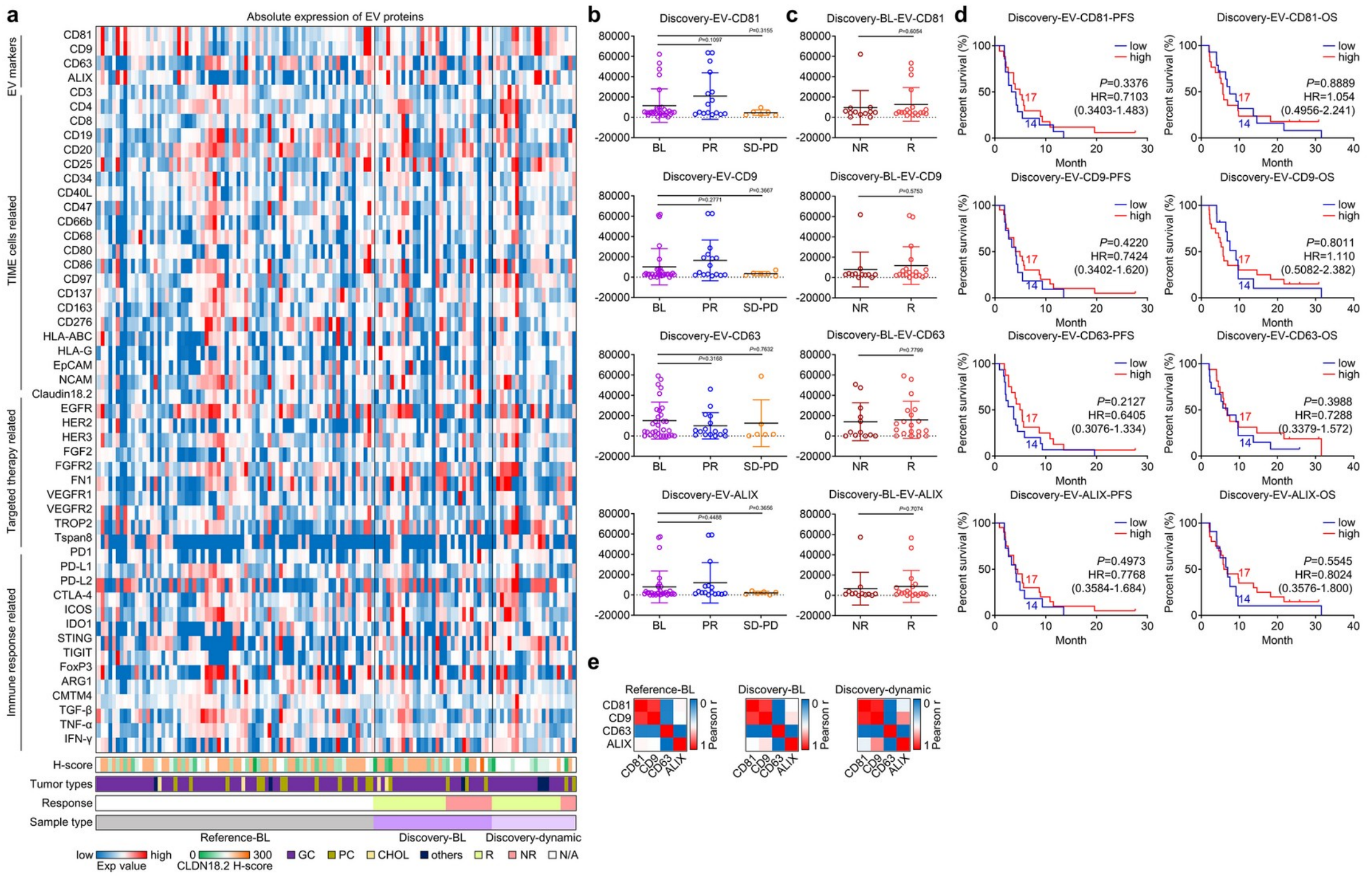

Supplementary Fig. 2. An absolute EV-protein expression spectrum for discovery cohort. (a) Heatmap for absolute expressions of 50 panel EV-proteins at all time points in reference and discovery cohorts. **(b)** Absolute expressions of EV-specific markers at BL, PR or SD/PD points. **(c)** Absolute expressions of EV-specific markers at BL between responders (R) and nonresponders (NR). **(d)** EV-specific markers' Kaplan-Meier analysis for PFS/OS. **(e)** The mutual expressional correlations amongst EV-specific markers for BL/dynamic samples in reference or discovery cohorts. For baseline analyses, n denotes independent patients. For longitudinal analyses, n denotes available plasma samples obtained from patients with serial sampling.~~Baseline analyses include biologically independent patients; longitudinal analyses include available plasma samples from patients with serial sampling.~~ Exact two-sided P values are shown in the corresponding panels.

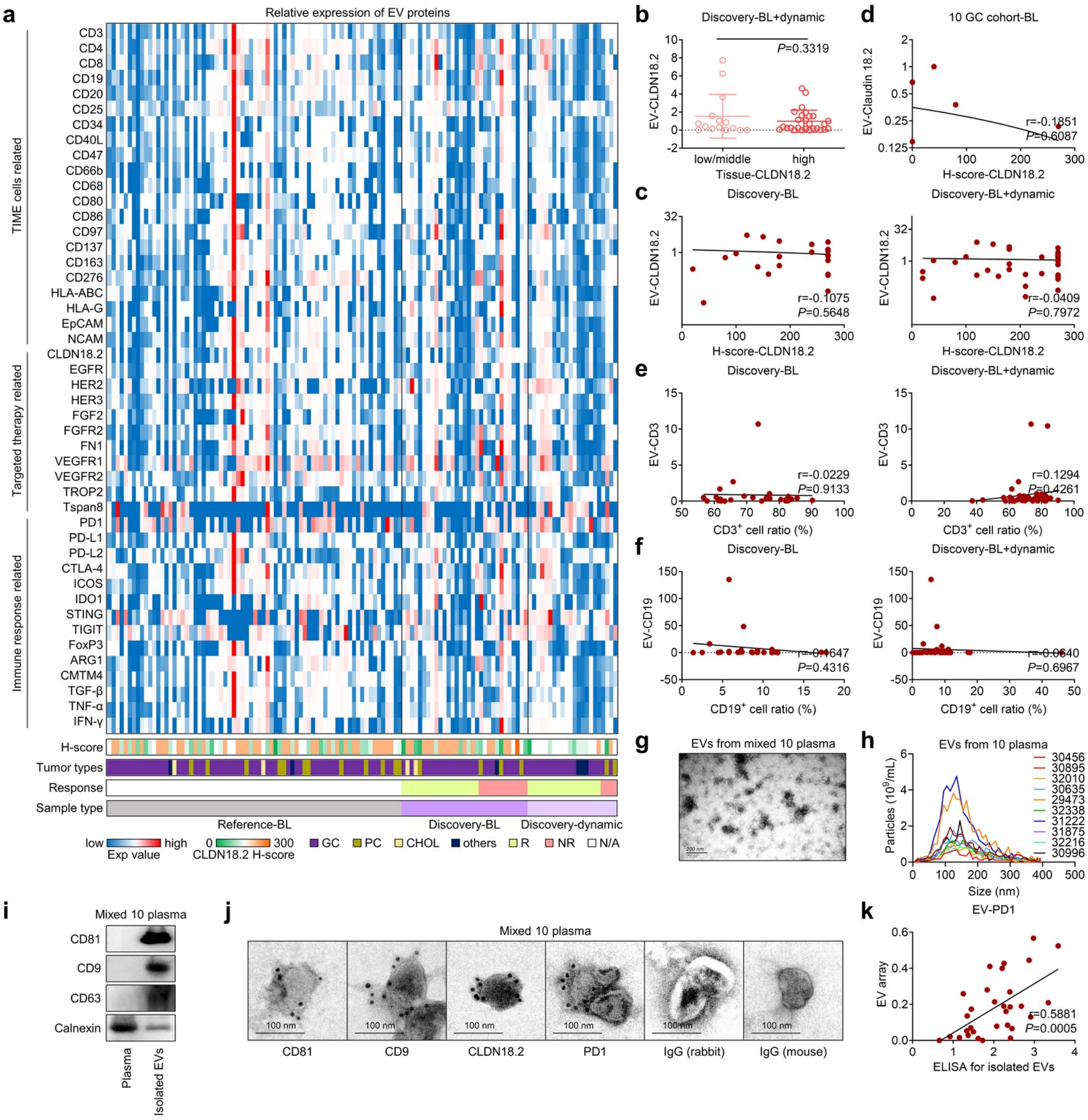

Supplementary Fig. 3. Verification of relative EV-protein expression spectrum for discovery cohort. (a) Heatmap for relative expressions of 50 panel EV-proteins at all time points in reference and discovery cohort. **(b)** Comparing EV-array-based-EV-CLDN18.2 expression between patients with low/intermediate tissue CLDN18.2 expression (n = 10 independent patients) and patients with high tissue CLDN18.2 expression (n = 21 independent patients) in the discovery cohort.~~with IHC-based-tissue-CLDN18.2 expression in discovery cohort.~~ **(c)** Linear correlations between EV-array-based-EV-CLDN18.2 expression and tissue-CLDN18.2 H-score in discovery cohort. **(d)** Linear correlation between EV-array-based-EV-CLDN18.2 expression and tissue-CLDN18.2 H-score for 5 tissue-CLDN18.2-negative and 5 tissue-CLDN18.2-positive GC patients. Linear correlations between **(e)** EV-array-based-EV-CD3 expression and flow-based-CD3$^+$ T cell ratio, or between **(f)** EV-array-based-EV-CD19

expression and flow-based-CD19$^+$ B cell ratio in discovery cohort. **(g)** TEM-visualized shape for the mix of 10 isolated plasma EV samples from 10 independent patients. **(h)** NTA-assessed size and density of the same pooled plasma EV preparation.for 10 plasma EV samples from 10 independent patients. **(i)** Western Blot-assessed EV markers in the same pooled plasma EV preparation. of the mix of 10 plasma EV samples from 10 independent patients. **(j)** Immunogold labelling assay for CD81/CD9/CLDN18.2/PD1 in the same pooled plasma EV preparation.mix of 10 plasma EV samples from 10 independent patients. Panels g–j were performed using a pooled EV preparation generated from plasma samples obtained from 10 independent patients in the discovery cohort. **(k)** Correlations between EV-array-quantified and ELISA-quantified expressions of EV-PD1 for 31 plasma samples. Baseline analyses include biologically independent patients; longitudinal analyses include available plasma samples from patients with serial sampling. Exact two-sided *P* values are shown in the corresponding panels.

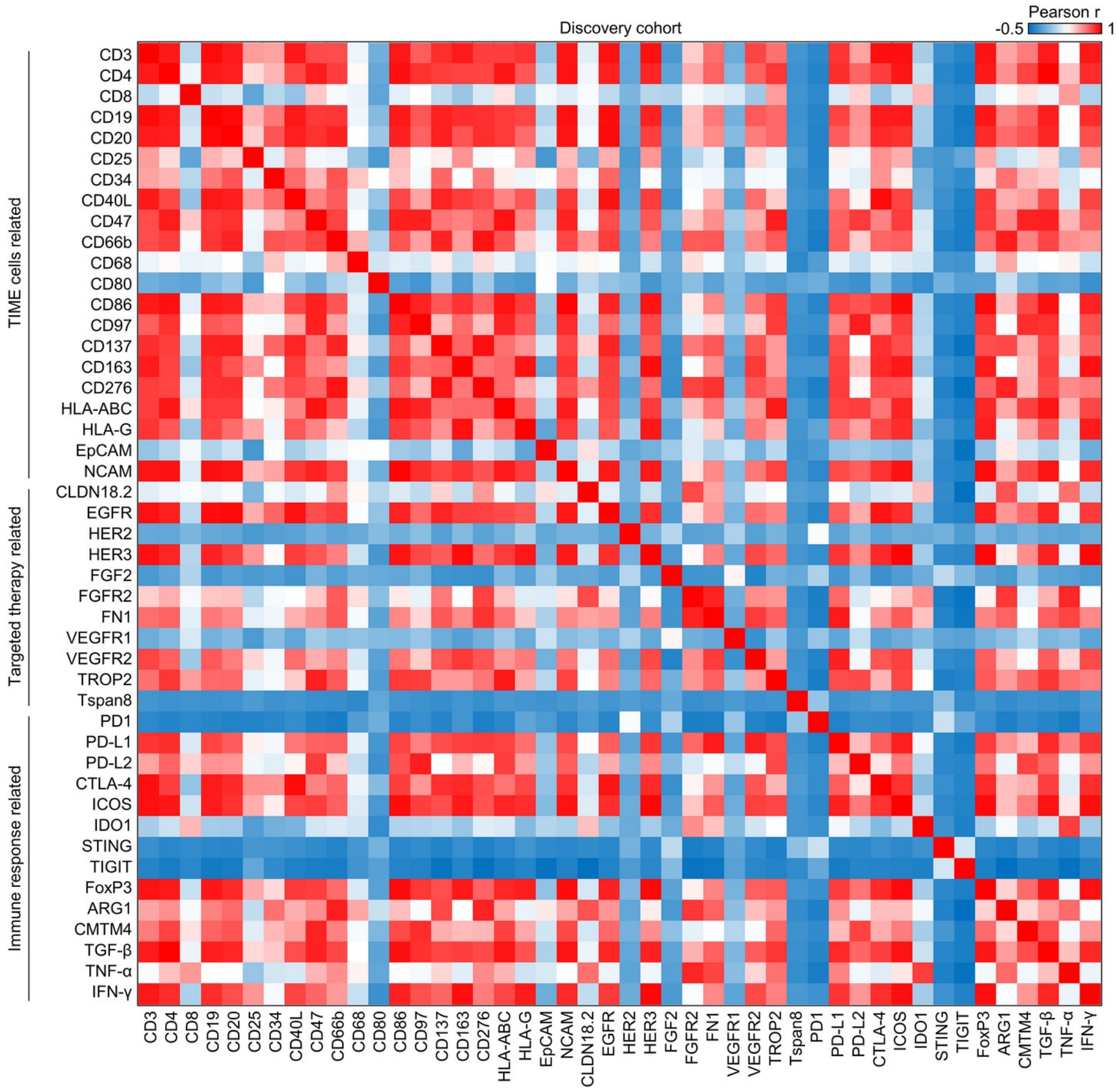

Supplementary Fig. 4. Mutual Pearson correlations amongst the expressions of 50 EV-proteins for baseline plasma samples of discovery cohort.

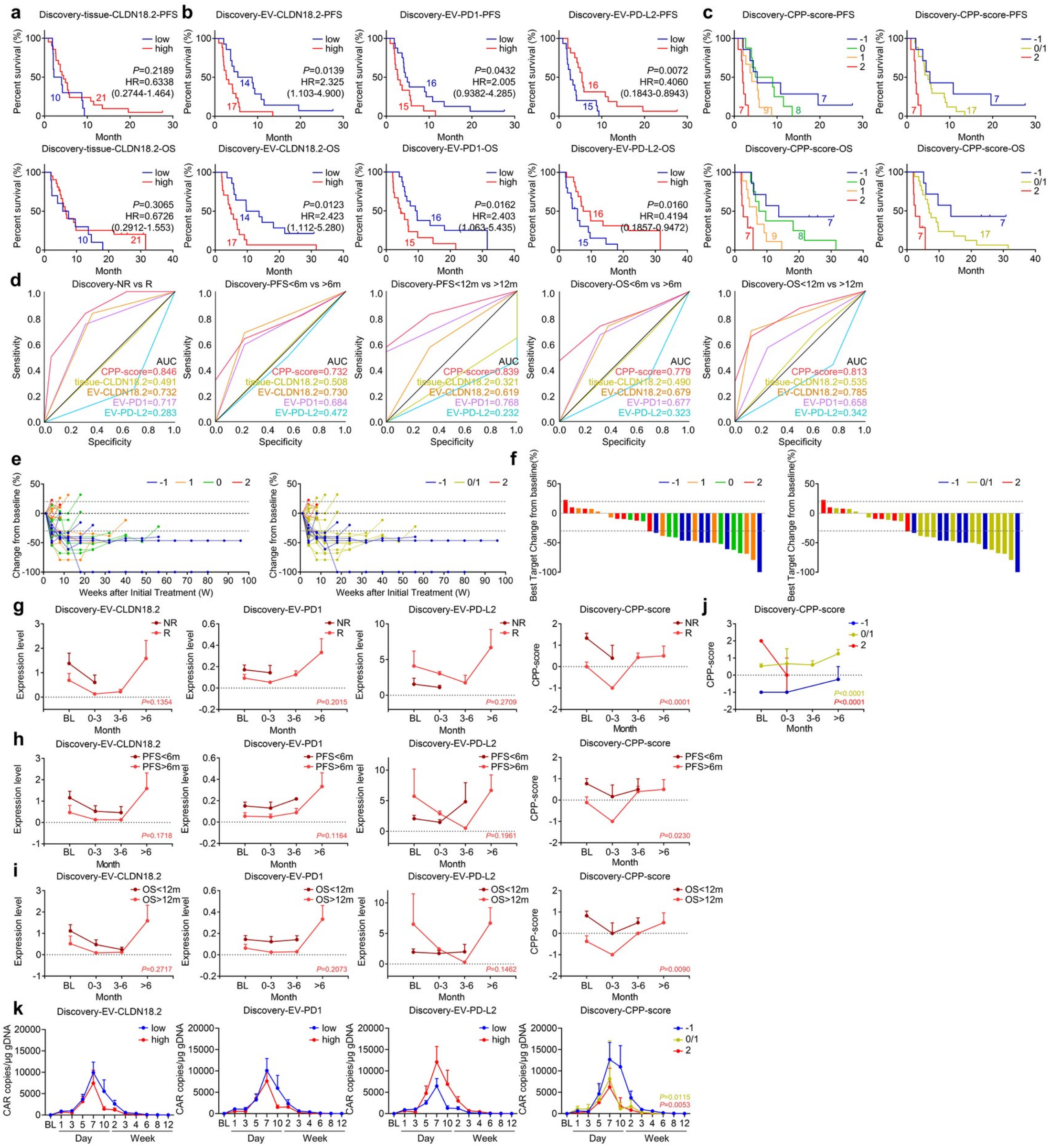

Supplementary Fig. 5. Three key EV-proteins and derived CPP-score reflect the efficacy of CLDN18.2-targeted-CAR-T therapy in discovery cohort. In CLDN18.2-targeted-CAR-T discovery cohort, Kaplan-Meier analysis for **(a)** tissue-CLDN18.2-related PFS/OS, **(b)** EV-CLDN18.2/EV-PD1/EV-PD-L2-related PFS/OS, and **(c)** CPP-score-related PFS/OS were calculated. For discovery cohort, **(d)** tissue-CLDN18.2/EV-proteins/CPP-score's ROC assessment for efficacy of CLDN18.2-CAR-T, **(e)** CPP-score-stratified spider plot, **(f)** CPP-score-stratified waterfall plot were depicted. Dynamic EV-CLDN18.2/EV-PD1/EV-PD-L2/CPP-score between **(g)** nonresponders (NR)/responders (R), **(h)** PFS<6m/PFS>6m patients, **(i)** OS<12m/OS>12m patients. **(j)** Dynamic EV-CLDN18.2/EV-PD1/EV-PD-L2/CPP-score stratified by baseline-CPP-score. **(k)** Dynamic CAR-T cell

content stratified by baseline-EV-CLDN18.2/EV-PD1/EV-PD-L2/baseline-CPP-score. Panels a–f were based on n = 31 independent patients in the discovery cohort. Panels g–j were based on available serial plasma samples obtained from 22 independent patients in the discovery cohort. For longitudinal analyses, each patient contributed repeated plasma measurements at the indicated time points, and the number of available samples may vary between time points. Panel k included independent patients with available serial peripheral-blood CAR-T-cell measurements. Survival differences were assessed using two-sided log-rank tests, and longitudinal data were analysed using two-way repeated-measures ANOVA. Statistical differences were assessed using Logrank test or two-way repeated-measures ANOVA. Exact two-sided *P* values are shown in the corresponding panels.

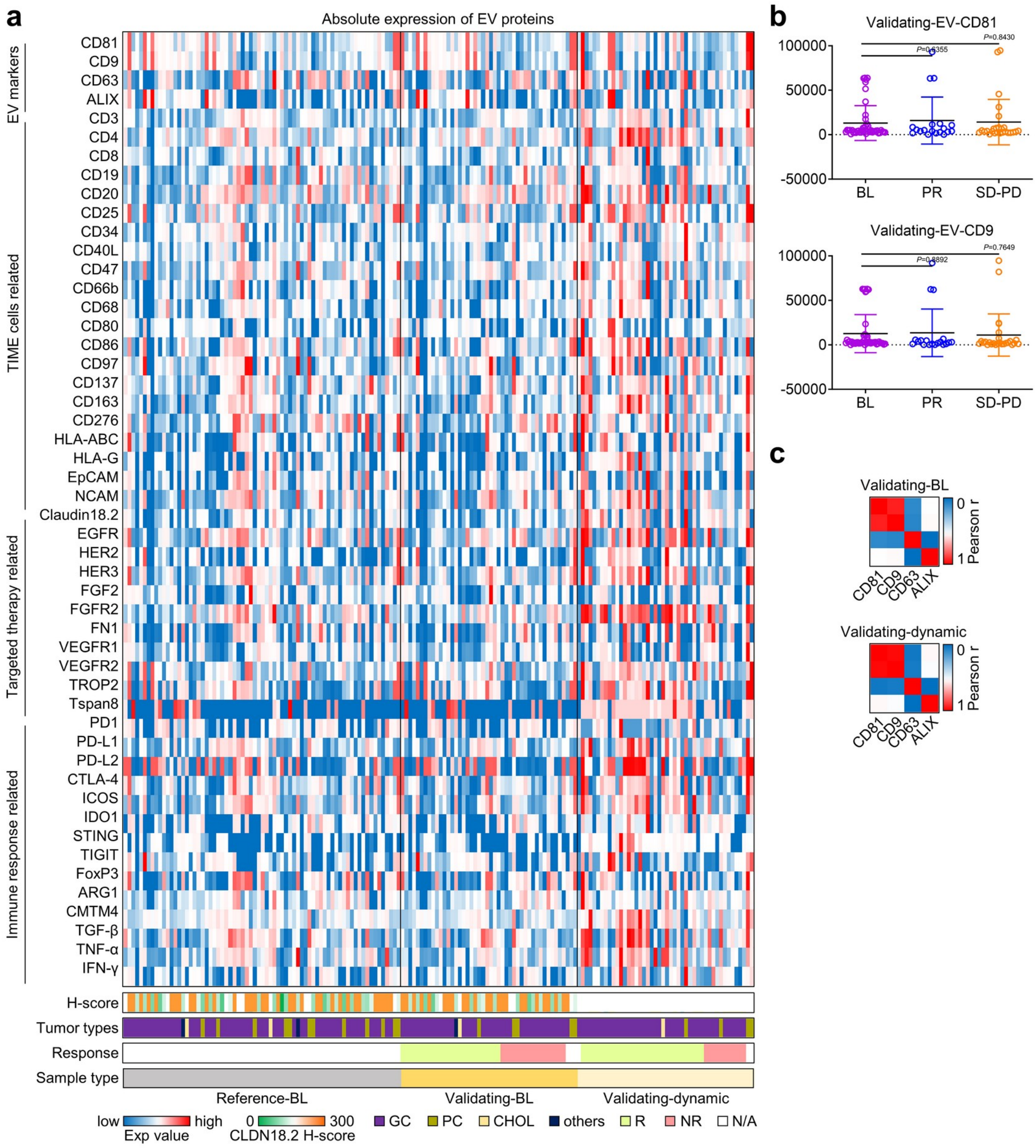

Supplementary Fig. 6. An absolute EV-protein expression spectrum for validating cohort. (a) heatmap for absolute expressions of 50 panel EV-proteins at all time points in reference and validating cohorts. **(b)** Absolute expressions of EV-specific markers at BL, PR or SD/PD points for validating cohort. **(c)** The mutual expressional correlations amongst EV-specific markers at BL and all time points. The reference and validating cohorts comprised n = 71 and n = 46 biologically independent patients, respectively. For baseline analyses, n denotes biologically independent patients. For longitudinal analyses, n denotes available plasma samples obtained from patients with serial sampling. Exact two-sided *P* values are shown in the corresponding panels..

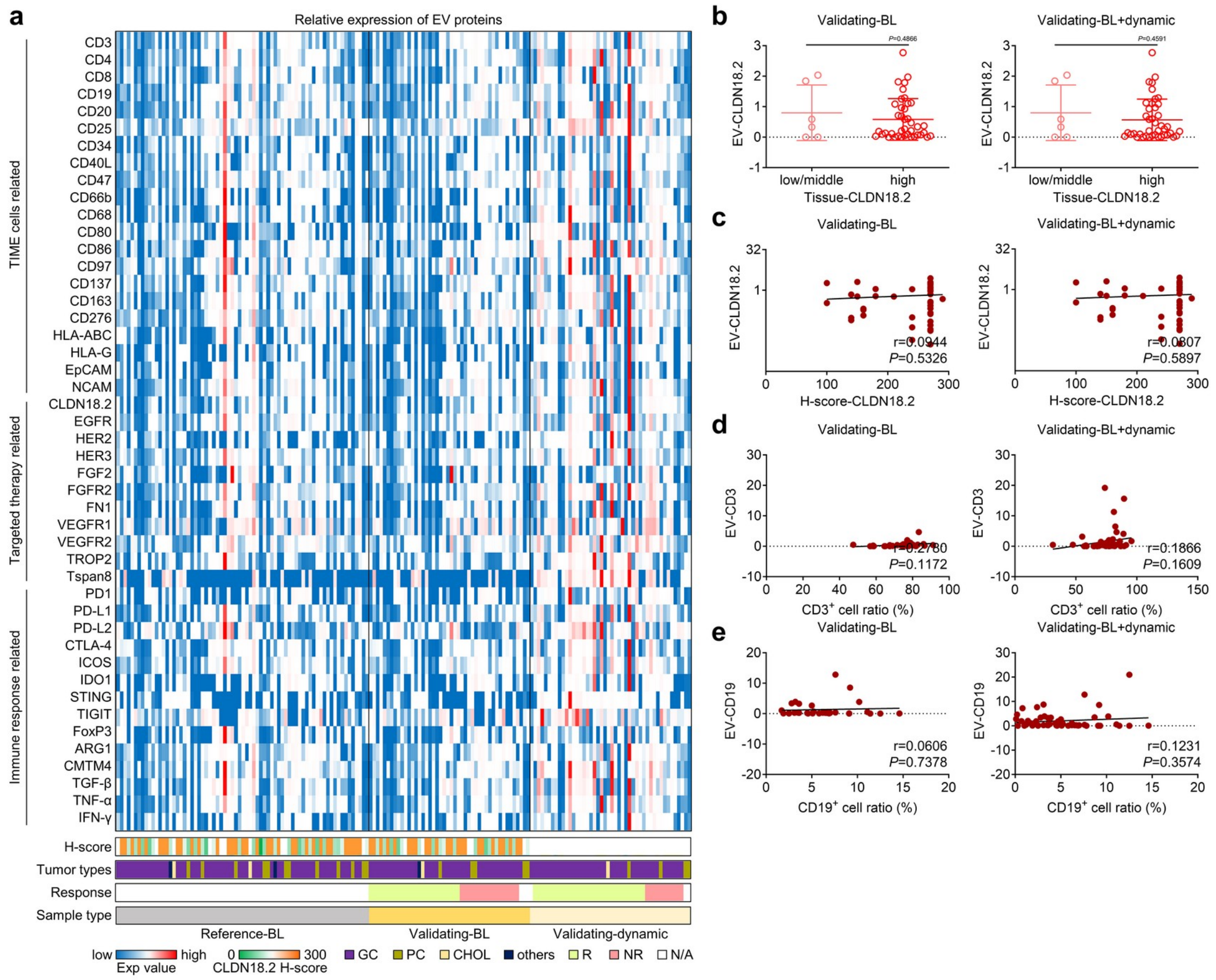

Supplementary Fig. 7. A relative EV-protein expression spectrum for validating cohort. (a) Heatmap for relative expressions of 50 panel EV-proteins at all time points in reference and validating cohort. **(b)** Comparing EV-array-based-EV-CLDN18.2 expression with IHC-based-tissue-CLDN18.2 expression in validating cohort. **(c)** Linear correlation between EV-array-based-EV-CLDN18.2 expression and IHC-based-tissue-CLDN18.2 H-score in validating cohort. Linear correlations between **(d)** EV-array-based-EV-CD3 expression and flow-based-$CD3^+$ T cell ratio in validating cohort, or between **(e)** EV-array-based-EV-CD19 expression and flow-based-$CD19^+$ B cell ratio in validating cohort. The reference and validating cohorts comprised n = 71 and n = 46 independent patients, respectively. For baseline analyses, n denotes independent patients, each contributing one baseline plasma sample; for longitudinal analyses, n denotes available follow-up plasma samples obtained from patients with serial sampling. ~~Baseline analyses include biologically independent patients; longitudinal analyses include available plasma samples from patients with serial sampling.~~ Exact two-sided *P* values are shown in the corresponding panels.

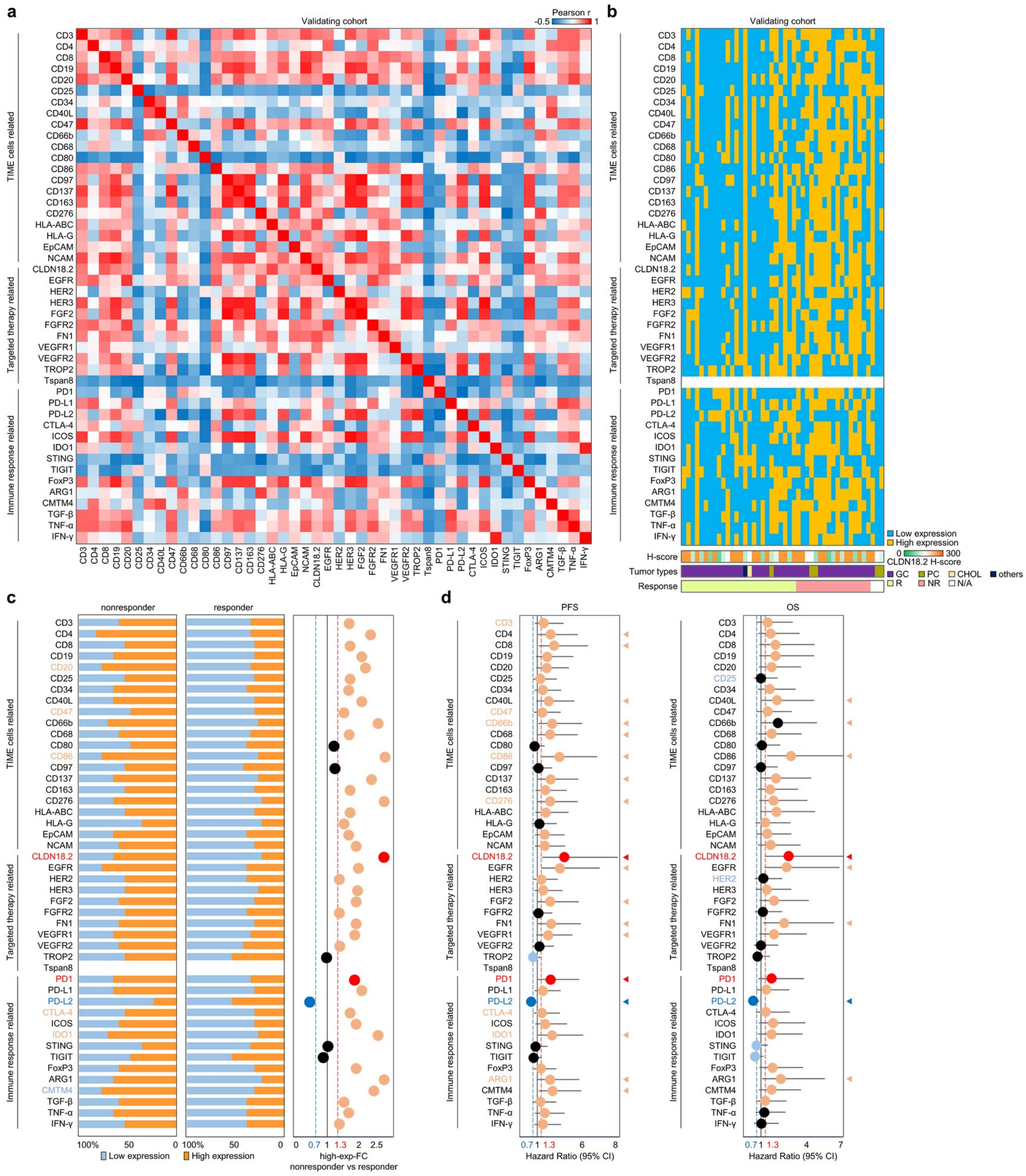

Supplementary Fig. 8. Screening plasma EV-proteins related with efficacy of CLDN18.2-targeted-CAR-T in validating cohort. (a) Mutual correlations amongst the expressions of 50 EV-proteins in validating cohort of CLDN18.2-CAR-T therapy. **(b)** Classifying categorical expressions of each EV-proteins in validating cohort by referring to the median of reference cohort. **(c)** Comparing each EV-proteins' proportions of high-expression between responders and nonresponders to generated fold change values. **(d)** EV-proteins' prognostic indications for PFS/OS (Logrank test, dot=Hazard Ratio, band=95% CI of Hazard Ratio, triangle represents *P*<0.05 for PFS/OS). Panels a–d included n = 46 independent patients from the validating cohort. High and low EV-protein expression was defined using median

values derived from an independent reference cohort of 71 untreated patients with gastrointestinal cancers.

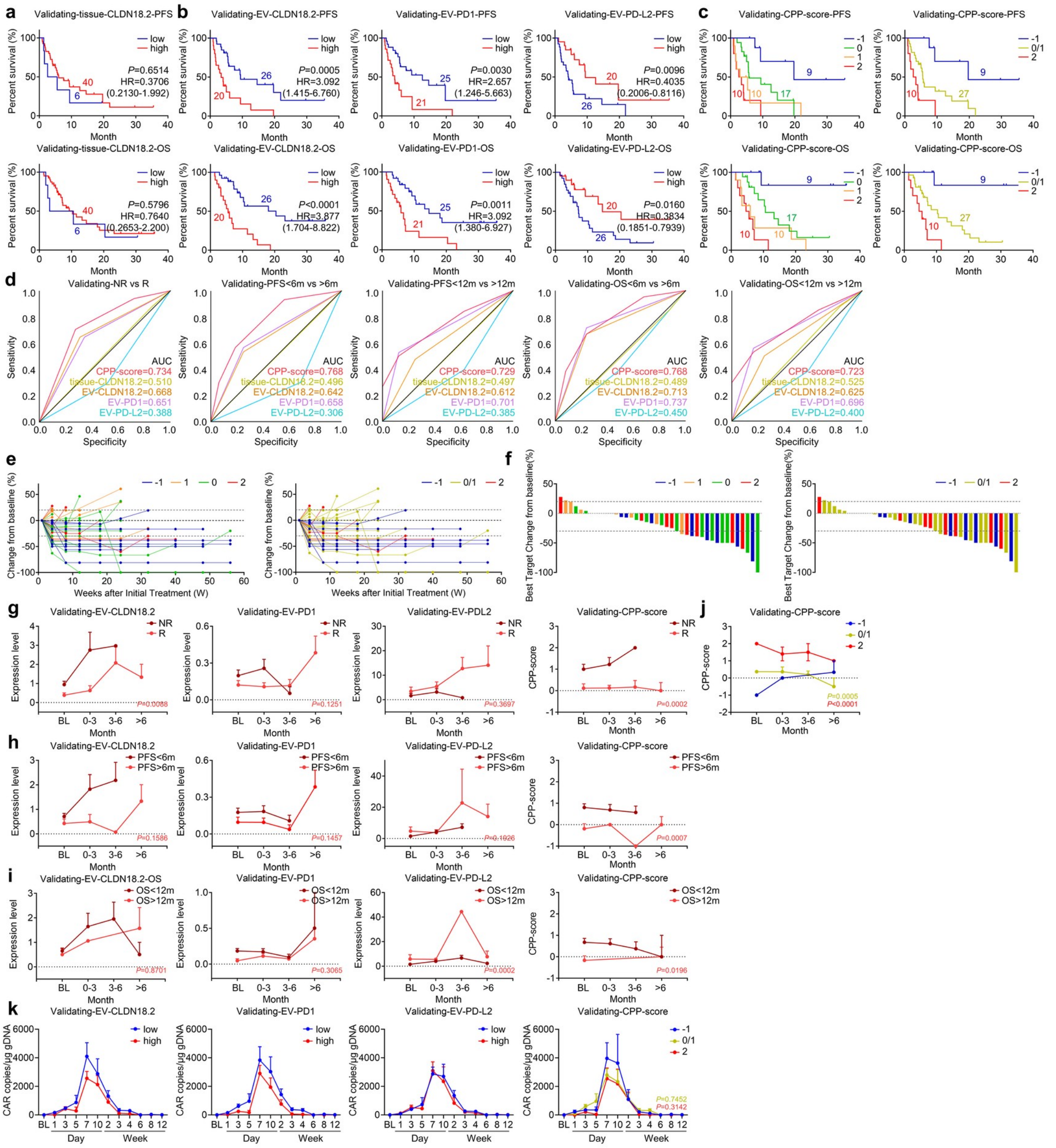

Supplementary Fig. 9. Three key EV-proteins and derived CPP-score reflect the efficacy of CLDN18.2-targeted-CAR-T therapy in validating cohort. In CLDN18.2-targeted-CAR-T validating cohort, Kaplan-Meier analysis for **(a)** tissue-CLDN18.2-related PFS/OS, **(b)** EV-CLDN18.2/EV-PD1/EV-PD-L2-related PFS/OS, and **(c)** CPP-score-related PFS/OS were calculated. For validating cohort, **(d)** tissue-CLDN18.2/EV-proteins/CPP-score's ROC assessment for efficacy of CLDN18.2-CAR-T, **(e)** CPP-score-stratified spider plot, **(f)** CPP-score-stratified waterfall plot were depicted. Dynamic EV-CLDN18.2/EV-PD1/EV-PD-L2/CPP-score between **(g)** nonresponders (NR)/responders (R), **(h)** PFS<6m/PFS>6m patients, **(i)** OS<12m/OS>12m patients. **(j)** Dynamic EV-CLDN18.2/EV-PD1/EV-PD-L2/CPP-score stratified by baseline-CPP-score. **(k)** Dynamic CAR-T cell

content stratified by baseline-EV-CLDN18.2/EV-PD1/EV-PD-L2/baseline-CPP-score. Panels a–f were based on baseline data from n = 46 independent patients in the validating cohort. For panels g–j, n denotes independent patients with available serial plasma measurements at the indicated time points. Panel k included independent patients with available serial peripheral-blood CAR-T-cell measurements. Group-specific sample sizes are indicated in the corresponding panels. Survival differences were assessed using two-sided log-rank tests, and longitudinal data were analysed using two-way repeated-measures ANOVA. Statistical differences were assessed using Logrank test or two-way repeated-measures ANOVA. Exact two-sided *P* values are shown in the corresponding panels.

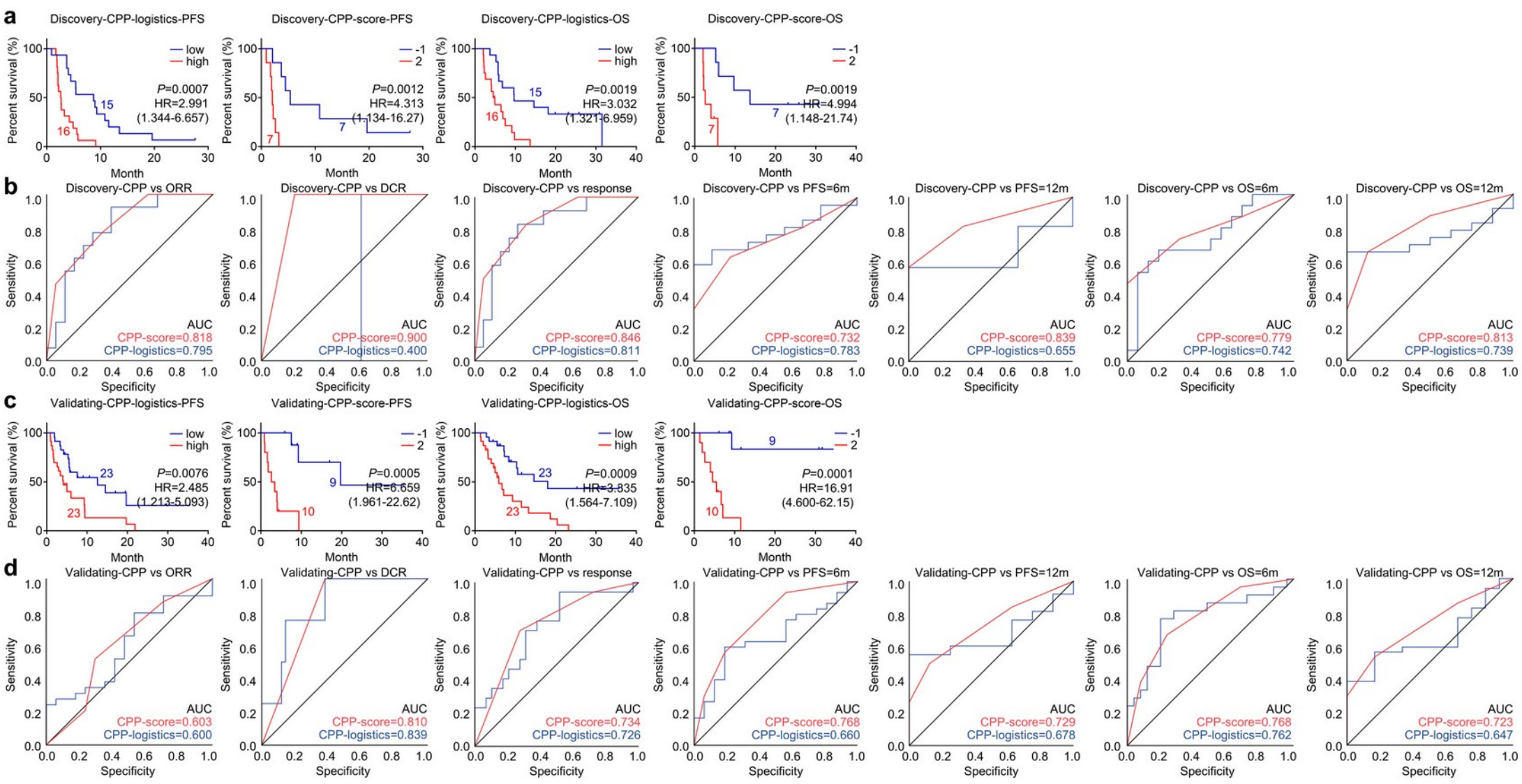

Supplementary Fig. 10. Comparing the robustness to predict efficacy of CLDN18.2-CAR-T between a logistics regression-weighted model (CPP-logistics) and CPP-score. (a) The robustness to assess PFS/OS for CPP-logistics and CPP-score in discovery cohort. **(b)** The robustness of ROC-assessed characterization on ORR/DCR/responses/prognosis for CPP-logistics and CPP-score in discovery cohort. **(c)** The robustness to assess PFS/OS for CPP-logistics and CPP-score in validating cohort. **(d)** The robustness of ROC-assessed characterization on ORR/DCR/responses/prognosis for CPP-logistics and CPP-score in validating cohort. Panels a and b were based on n = 31 independent patients in the discovery cohort. Panels c and d were based on n = 46 independent patients in the validating cohort. Exact P values are shown in the corresponding panels.~~n denotes biologically independent patients.~~

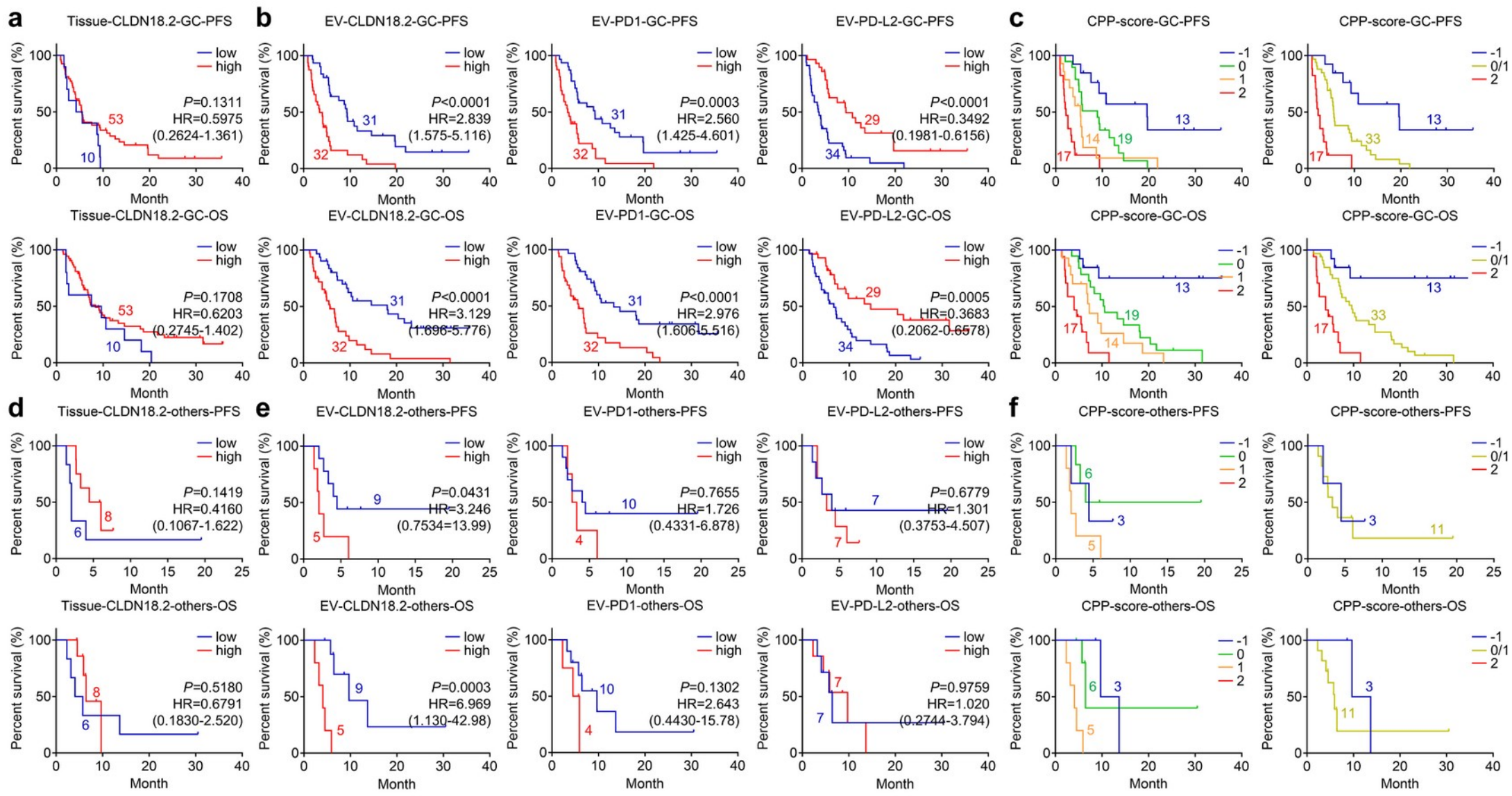

Supplementary Fig. 11. CPP-score predicts the efficacy of CLDN18.2-targeted-CAR-T therapy in GC and other cancers. (a) IHC-based-tissue-CLDN18.2's prognostic indication for PFS/OS in 63 GC patients treated with CLDN18.2-targeted-CAR-T therapy. **(b)** EV-CLDN18.2/EV-PD1/EV-PD-L2's prognostic indications for PFS/OS in GC patients. **(c)** CPP-score's prognostic indications for PFS/OS in GC patients. **(d)** IHC-based-tissue-CLDN18.2's prognostic indication for PFS/OS in 14 non-GC patients treated with CLDN18.2-targeted-CAR-T therapy. **(e)** EV-CLDN18.2/EV-PD1/EV-PD-L2's prognostic indications for PFS/OS in non-GC patients. **(f)** CPP-score's prognostic indications for PFS/OS in non-GC patients. Panels a–c included n = 63 independent patients with gastric cancer, and panels d–f included n = 14 independent patients with non-gastric gastrointestinal cancers. Survival differences were assessed using two-sided log-rank tests. Exact P values are shown in the corresponding panels.

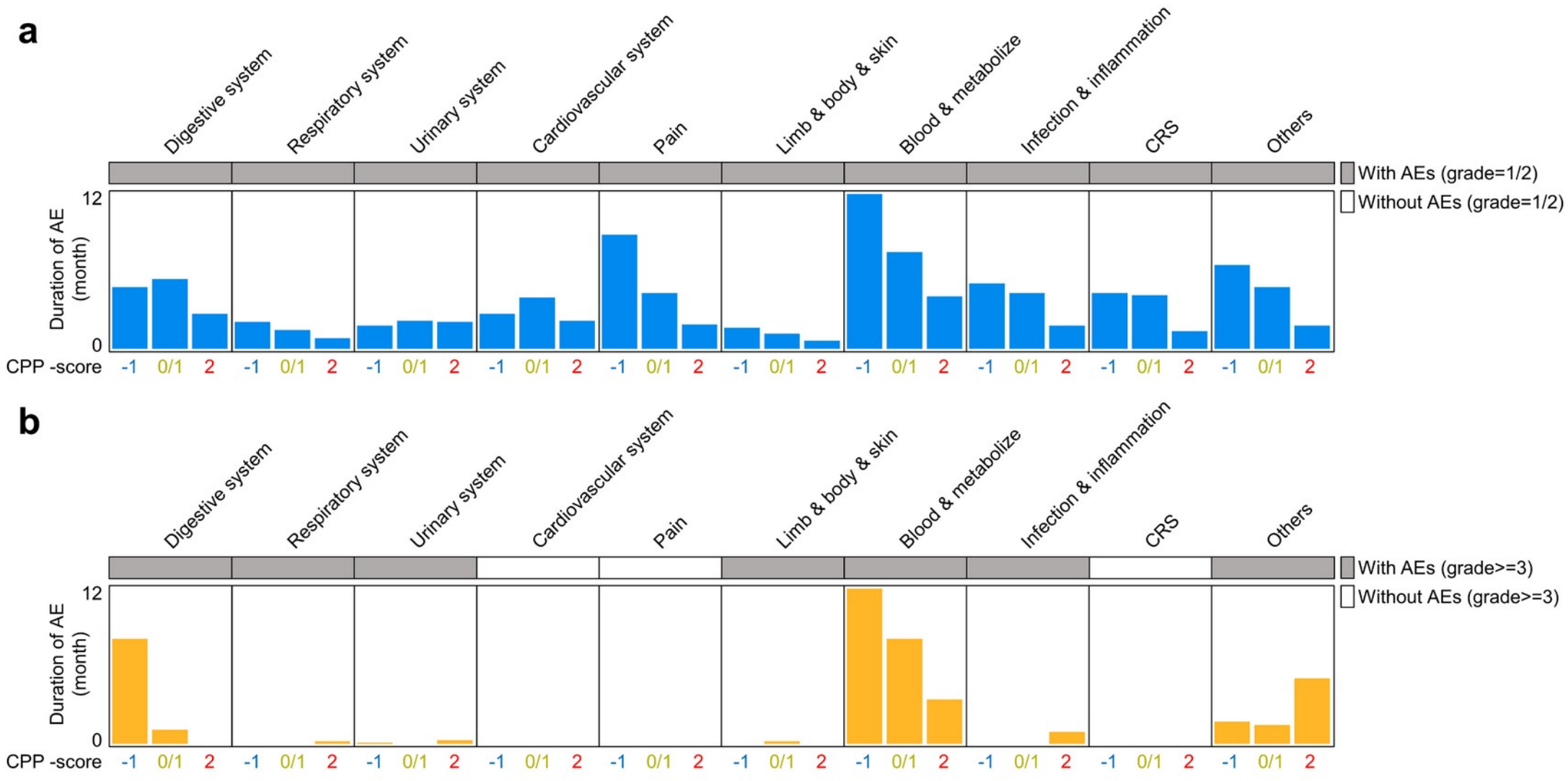

Supplementary Fig. 12. CPP-score's indication on adverse events (AEs) related with CLDN18.2-targeted-CAR-T therapy. (a) CPP-score's indication on sites/durations of grade 1/2 AEs in all CLDN18.2-CAR-T-treated patients. **(b)** CPP-score's indication on sites/durations of grade 3 AEs in all CLDN18.2-CAR-T-treated patients. The analysis was based on the combined cohort of 77 independent patients. For each panel, n denotes patients who experienced the indicated grade of adverse events.

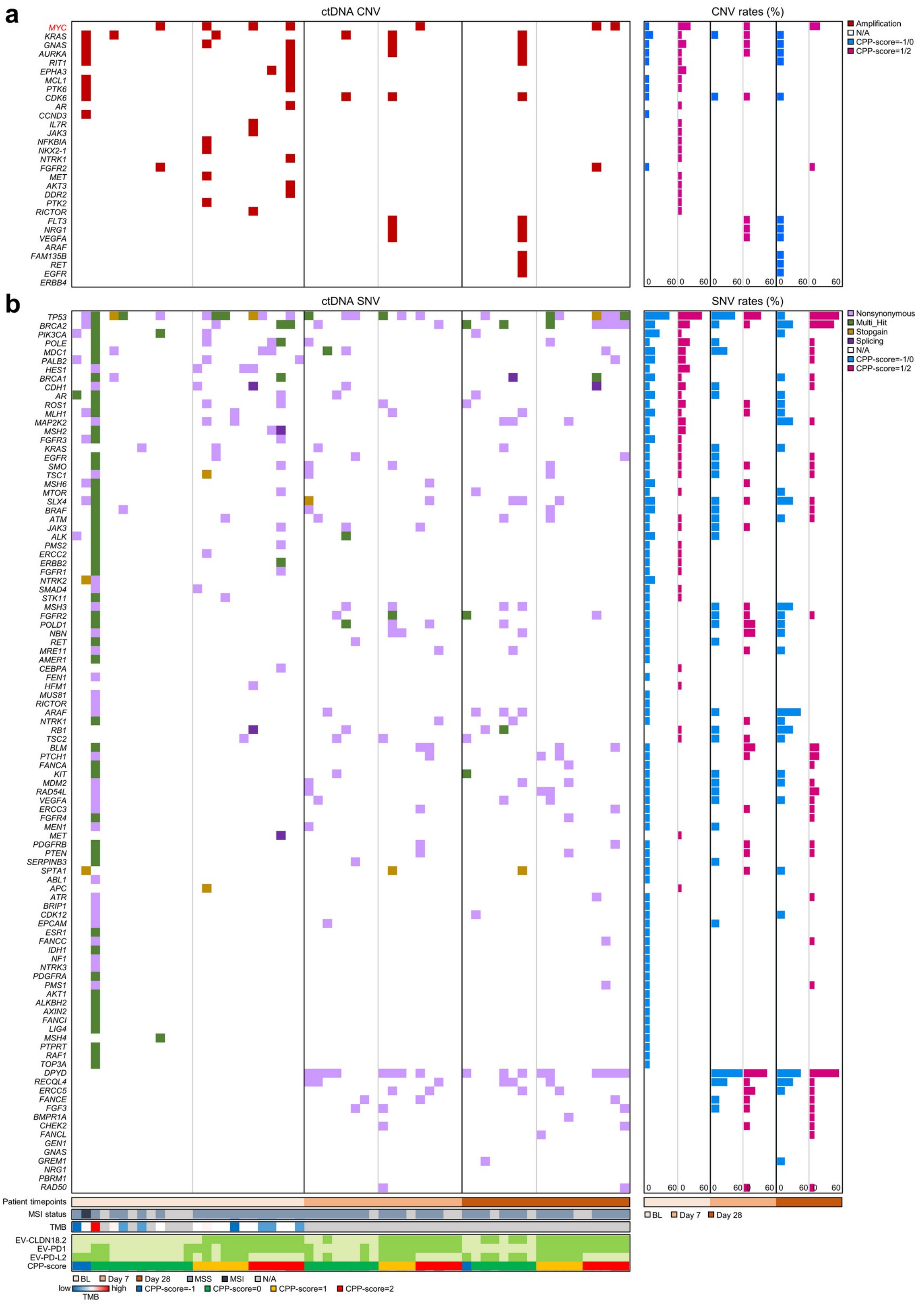

Supplementary Fig. 13. Dynamic plasma EV-proteins/CPP-score paired with ctDNA profiles for CLDN18.2-CAR-T patients. (a) Dynamic plasma EV-proteins/CPP-score paired with CNVs. **(b)** Dynamic plasma EV-proteins/CPP-score paired with SNVs. Panels a and b included independent patients

with matched EV-array and ctDNA measurements. Each patient contributed up to three serial plasma samples collected at baseline, day 7 and day 28. n denotes independent patients.

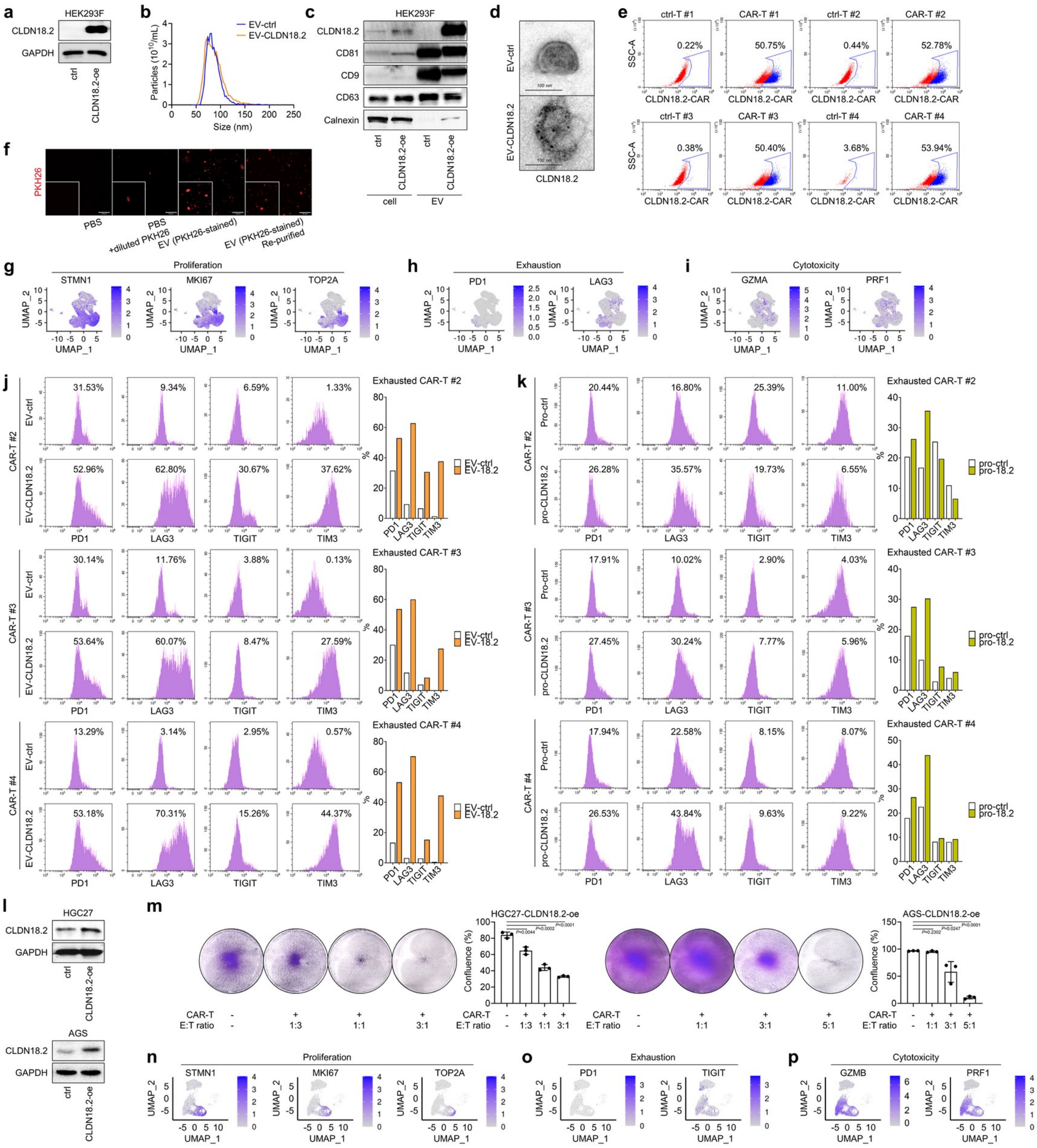

Supplementary Fig. 14. Extended evidences supporting EV-CLDN18.2 overloads and exhausts CLDN18.2-targeted-CAR-T cells. (a) Western blot for stable overexpression of CLDN18.2 in HEK293F cell. **(b)** NTA-assessed size and density for isolated EV-CLDN18.2. **(c)** Western Blot-assessed EV markers for isolated EV-CLDN18.2. **(d)** Immunogold labelling assay for the expression of CLDN18.2 on EV-CLDN18.2. **(e)** Flow-based positive expression rate of CAR in constructed CAR-T cells from donor #1-#4. **(f)** Fluorescent imaging for PKH26-stained EVs before and after re-purification, with PBS/PKH26-stained PBS as blank and negative control. ~~**(g)**~~ **(g)** Proliferation-related, **(h)** exhaustion-related and **(i)** cytotoxicity-related feature plots derived from single-cell RNA-sequencing of EV-control- and EV-CLDN18.2-treated CLDN18.2-targeted CAR-T cells. ~~Proliferative-related feature~~

plot for EV-treated CAR-T cells. **(h)** Exhaustion-related feature plot for EV-treated CAR-T cells. **(i)** Cytotoxicity-related feature plot for EV-treated CAR-T cells. **(j)** EV-CLDN18.2 and **(k)** pro-CLDN18.2's influence on the exhaustion of CLDN18.2-targeted-CAR-T cells from donor #2-#4. **(l)** Western blot-assessed CLDN18.2 overexpression in GC cells HGC27/AGS. **(m)** CLDN18.2-CAR-T-mediated cytotoxicity against CLDN18.2-overexpressing HGC27 and AGS cells at different E:T ratios (mean±SD, calculated from n=3 independent cytotoxicity experimentsbiological replicates, two-sided t-test). **(n)** Proliferative-related feature plot for CT041-CG4006-treated patient PBMC samples. **(o)** Exhaustion-related feature plot for CT041-CG4006-treated patient PBMC samples. **(p)** Cytotoxicity-related feature plot for CT041-CG4006-treated patient PBMC samples. Panels n–p were based on PBMC samples collected at baseline, day 3 and day 7 from n = 5 independent patients in the validating cohort of the CT041-CG4006 trial. Data are presented as individual donor values and mean ± s.d. Statistical comparisons were performed using two-sided paired t-tests, and eExact *P* values are shown in the corresponding panels.

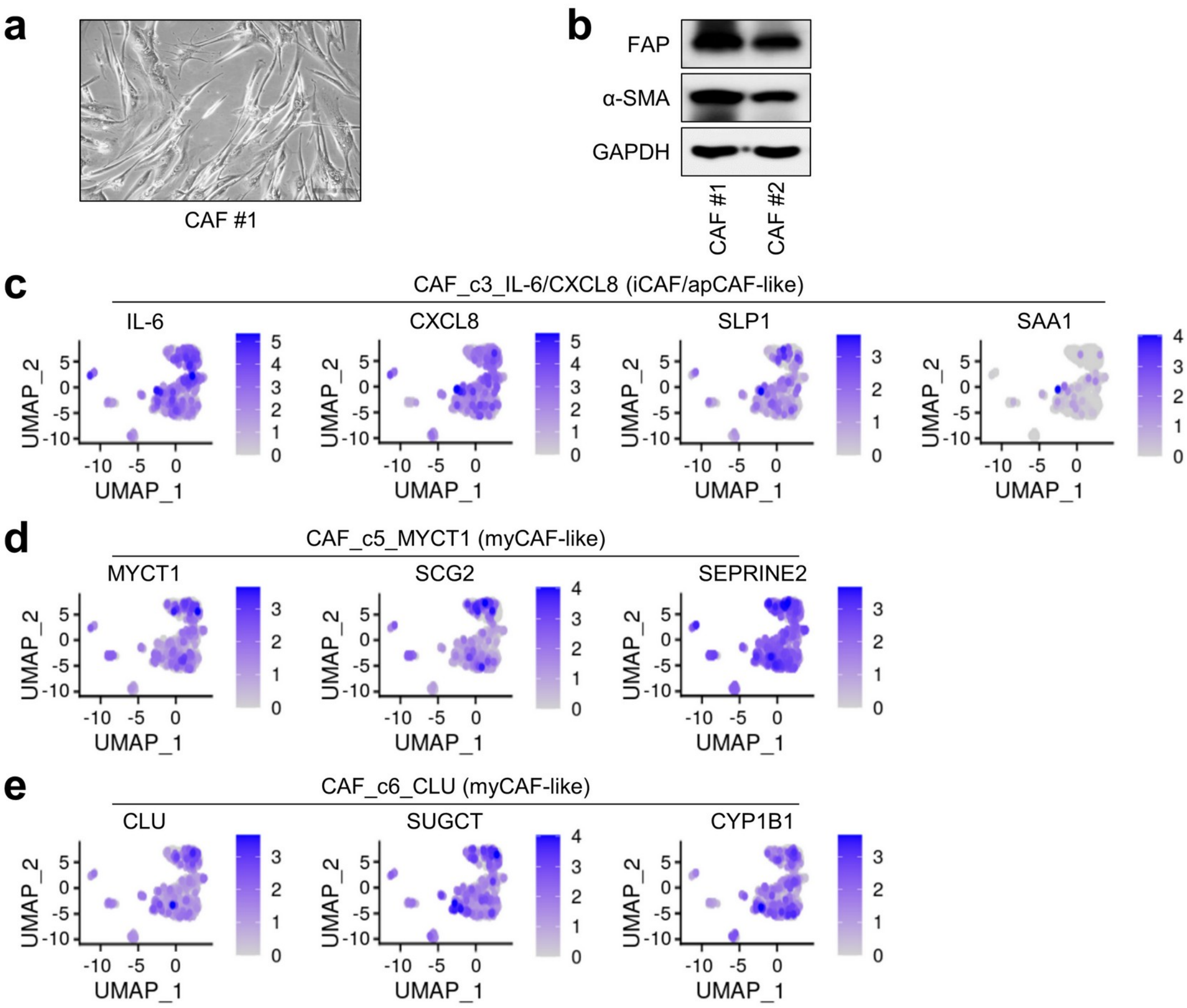

Supplementary Fig. 15. Extended evidences supporting EV-CLDN18.2 induces CAF activation. (a) Morphology of CAFs isolated from GC ascites. **(b)** Western blot-assessed expressions of canonical CAF markers in isolated CAFs. **(c)** Representative feature plots for CAF_c3_IL-6/CXCL8 linage. **(d)** Representative feature plots for CAF_c5_MYCT1 linage. **(e)** Representative feature plots for CAF_c6_CLU linage. Panels c–e were derived from single-cell RNA-sequencing analysis of EV-control- and EV-CLDN18.2-treated patient-derived CAFs.

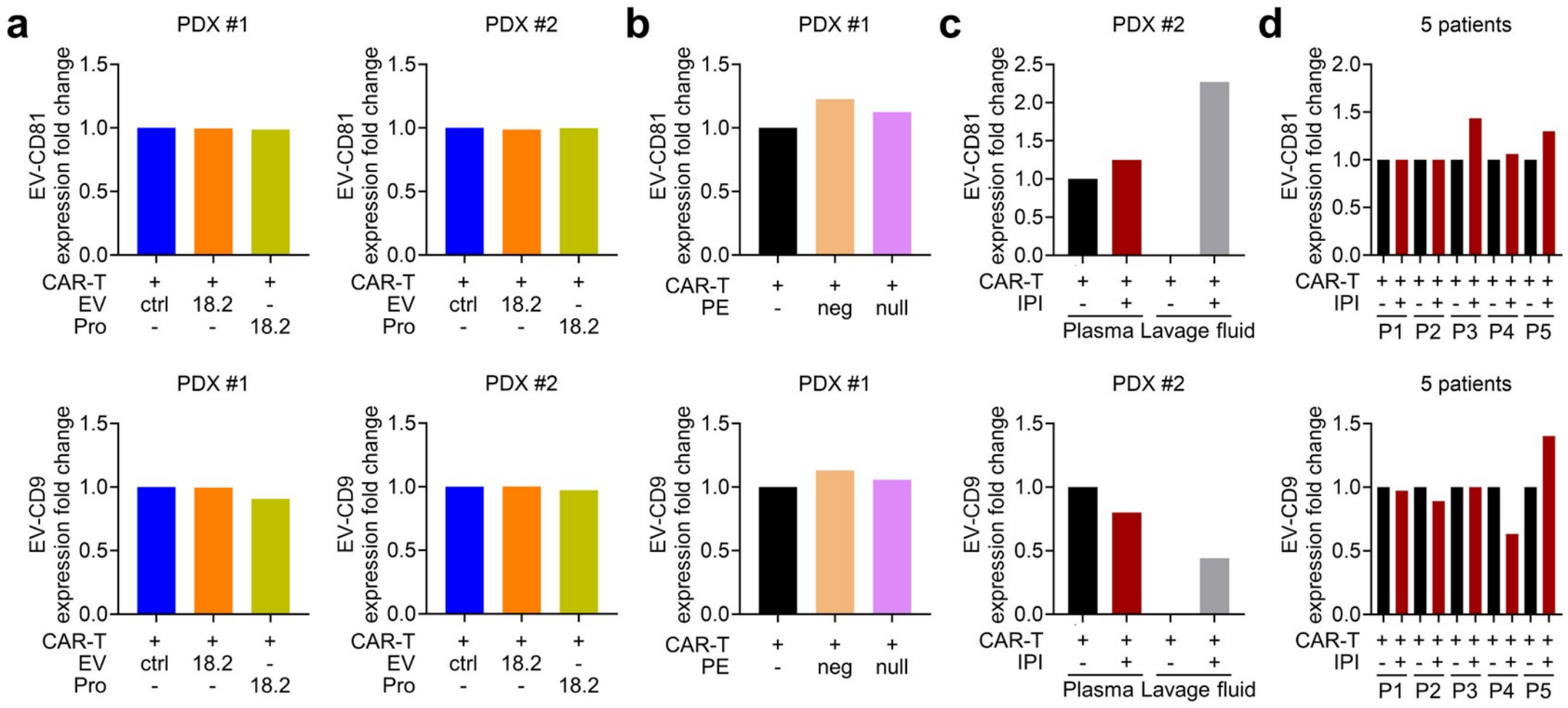

Supplementary Fig. 16. Changes of total EV content in plasma from PDX mice and patients. (a) CD81/CD9-characterized changes of total EV content for two PDX models received CLDN18.2-targeted-CAR-T and EV-CLDN18.2/pro-CLDN18.2. **(b)** CD81/CD9-characterized changes of total EV content for a PDX model received CLDN18.2-targeted-CAR-T and plasma exchange (PE). **(c)** CD81/CD9-characterized changes of total EV content for a PDX model received CLDN18.2-targeted-CAR-T and intraperitoneal infusion (IPI). **(d)** CD81/CD9-characterized changes of total EV content for five gastric cancer patients received intraperitoneal infusion (IPI).

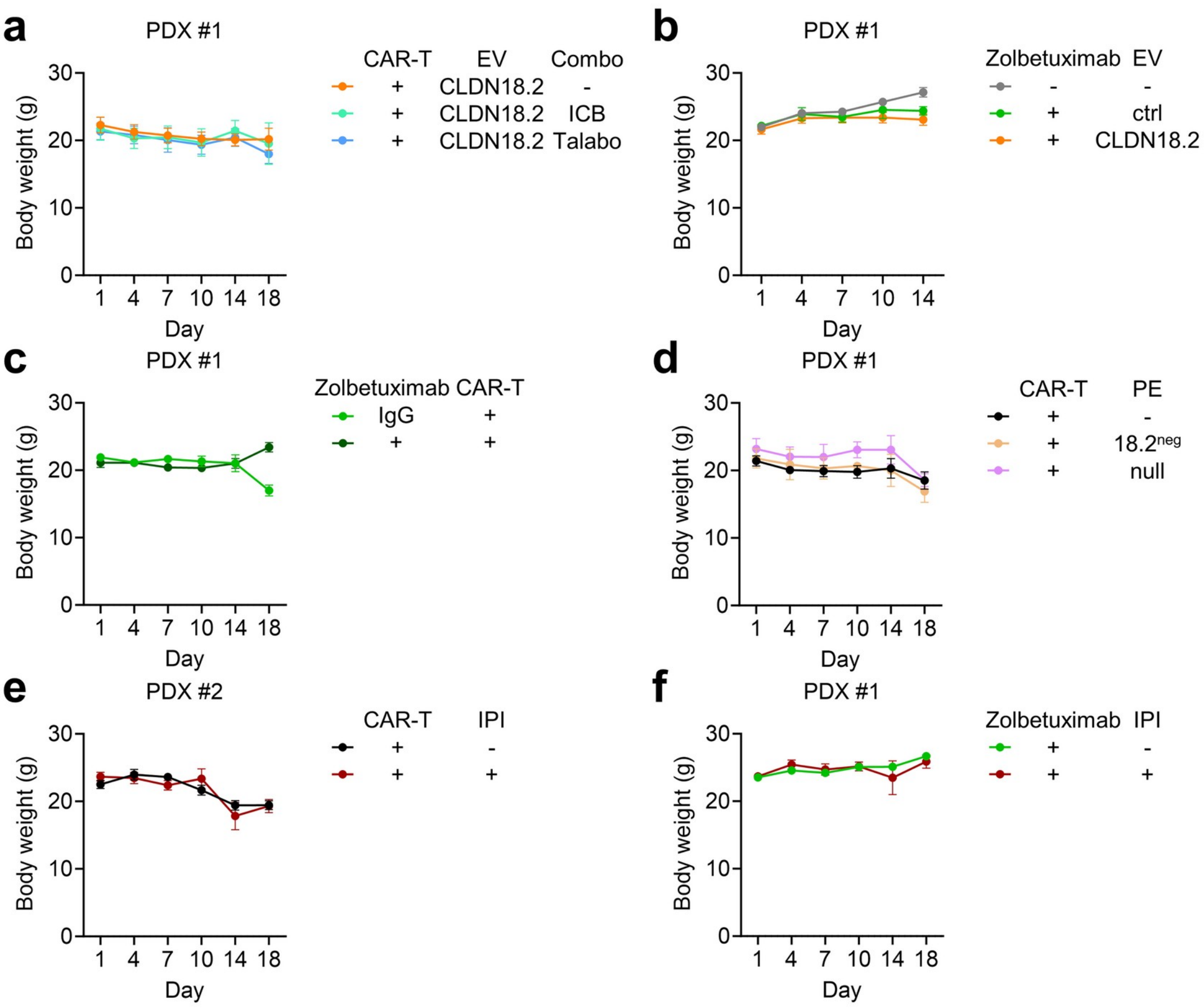

Supplementary Fig. 17. Changes of body weight for xenograft mice. (a) The influence of combining ICB (Tislelizumab) or Talabostat with CLDN18.2-CAR-T and EV-CLDN18.2 on body weight of xenograft mice. **(b)** The influence of combining EV-CLDN18.2 with anti-CLDN18.2-antibody on body weight of xenograft mice. **(c)** The influence of combining anti-CLDN18.2-antibody with CLDN18.2-CAR-T on body weight of xenograft mice. **(d)** The influence of combining PE with CLDN18.2-CAR-T on body weight of xenograft mice. **(e)** The influence of combining IPI with CLDN18.2-CAR-T on body weight of xenograft mice. **(f)** The influence of combining IPI with anti-CLDN18.2-antibody on body weight of xenograft mice. For all panels, data are presented as mean ± SD from n = 5 independent mice per treatment group. Each mouse contributed repeated body-weight measurements at the indicated time points. Data are presented as mean ± s.d. from n = 5 biologically independent mice per group.

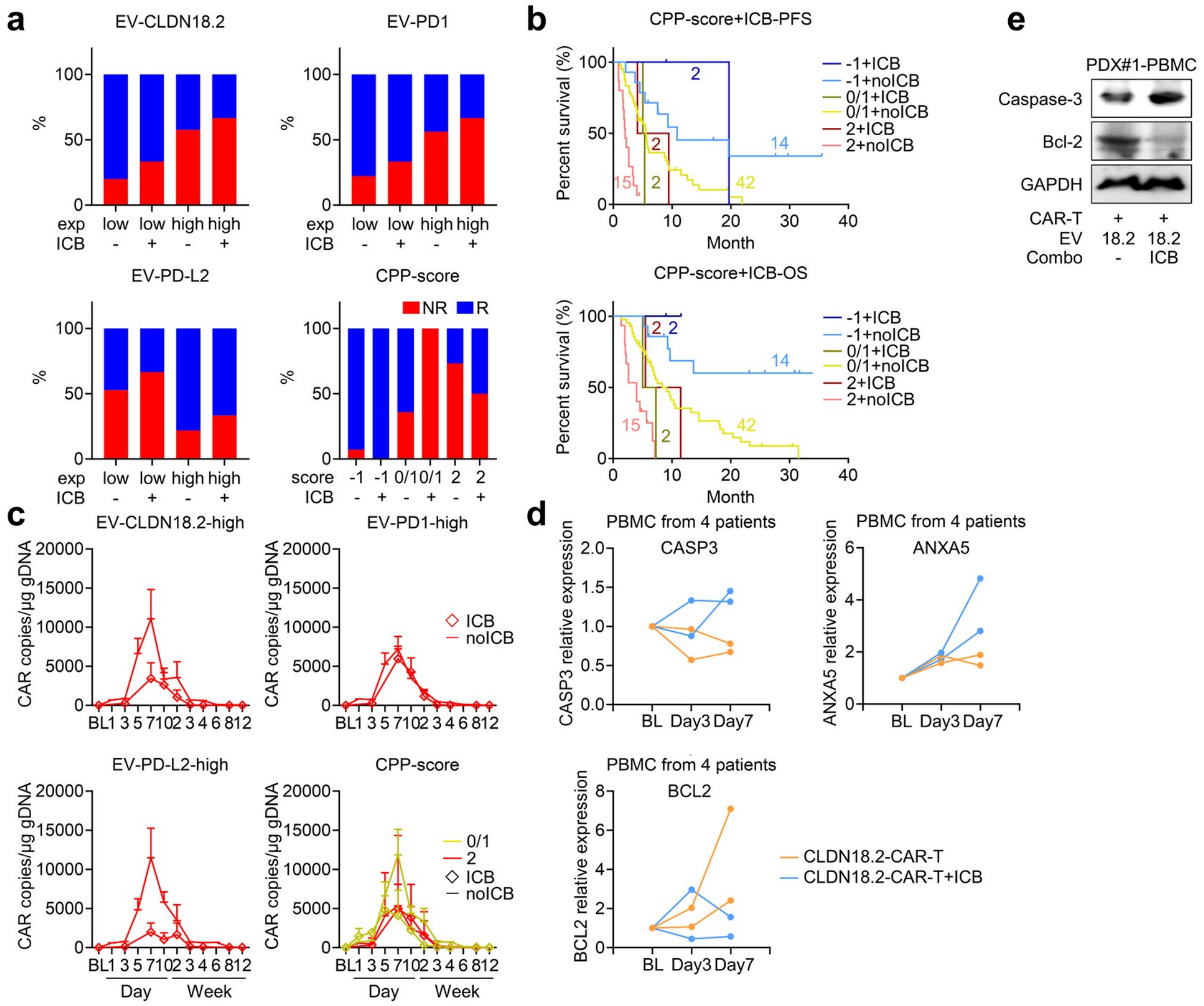

Supplementary Fig. 18. Combining ICB treatment induced AICD effect of CLDN18.2-targeted-CAR-T cells. (a) Response rates for all 77 patients stratified by integrating CPP-score and ICB. **(b)** PFS/OS proportions for all 77 patients stratified by integrating CPP-score and ICB. **(c)** Dynamic changes of plasma CAR-T cell content for patients harboring high EV-protein expression/CPP-score were stratified by ICB. n denotes independent patients with available serial peripheral-blood CAR-T-cell measurements. **(d)** Transcript levels of CASP3/ANXA5/BCL2 at baseline/Day 3/Day 7 of treatment, derived from PBMC of two patients received CLDN18.2-CAR-T and two patients received CLDN18.2-CAR-T combined with ICB. **(e)** Western blot analysis of Caspase 3 expression in PBMC from GC PDX#1 after received CLDN18.2-CAR-T or CLDN18.2-CAR-T combined with ICB.

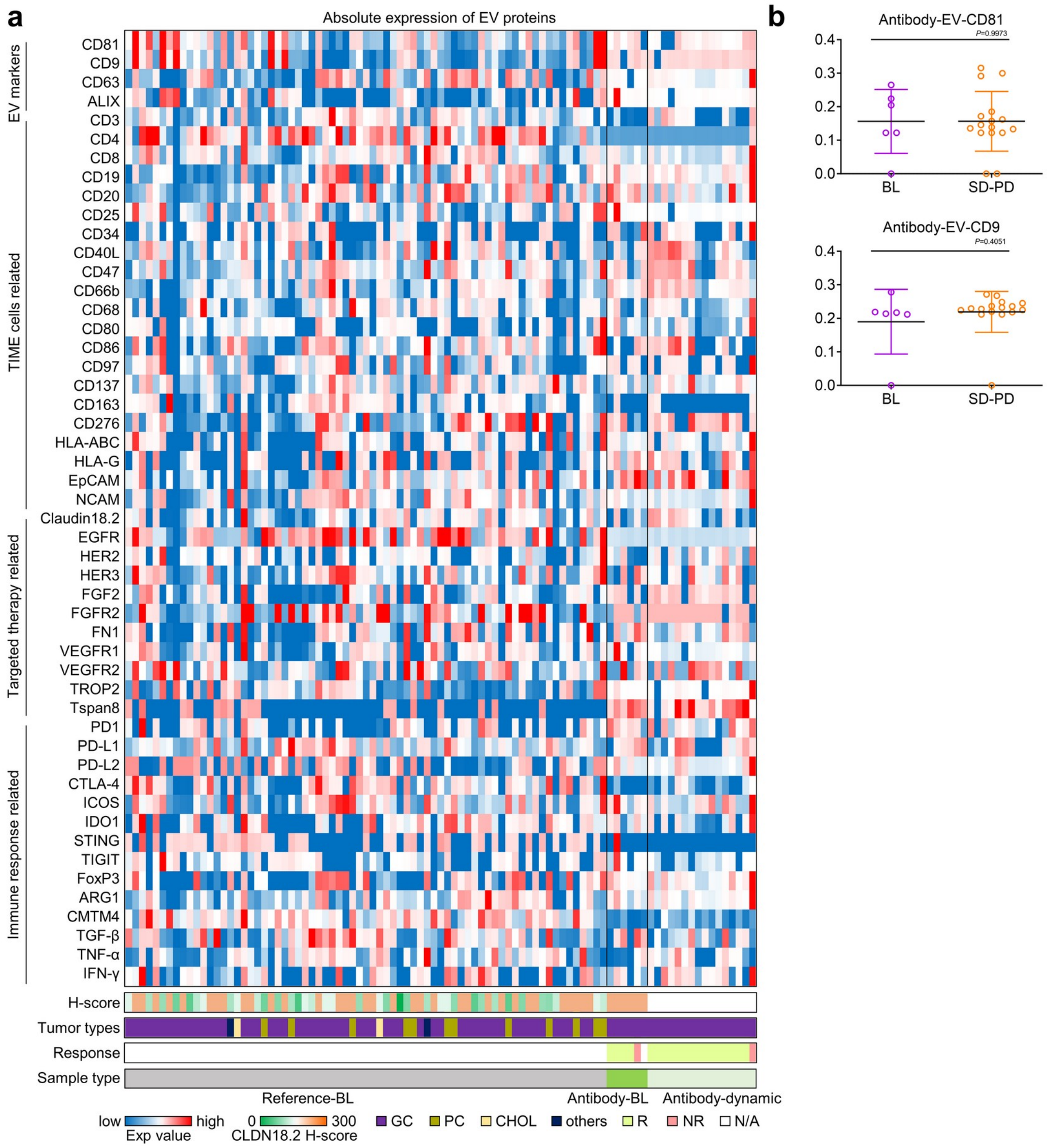

Supplementary Fig. 19. An absolute EV-protein expression spectrum for antibody cohort. (a) heatmap for absolute expressions of 50 panel EV-proteins for all time points in reference and antibody cohorts. **(b)** Absolute expressions of EV-specific markers at BL, PR or SD/PD points for antibody cohort. For baseline analyses, n denotes independent patients, each contributing one baseline plasma sample. For longitudinal analyses, n denotes available plasma samples obtained from patients with serial sampling. Statistical comparisons were performed using two-sided paired t-tests, and exact *P* values are shown in the corresponding panels.

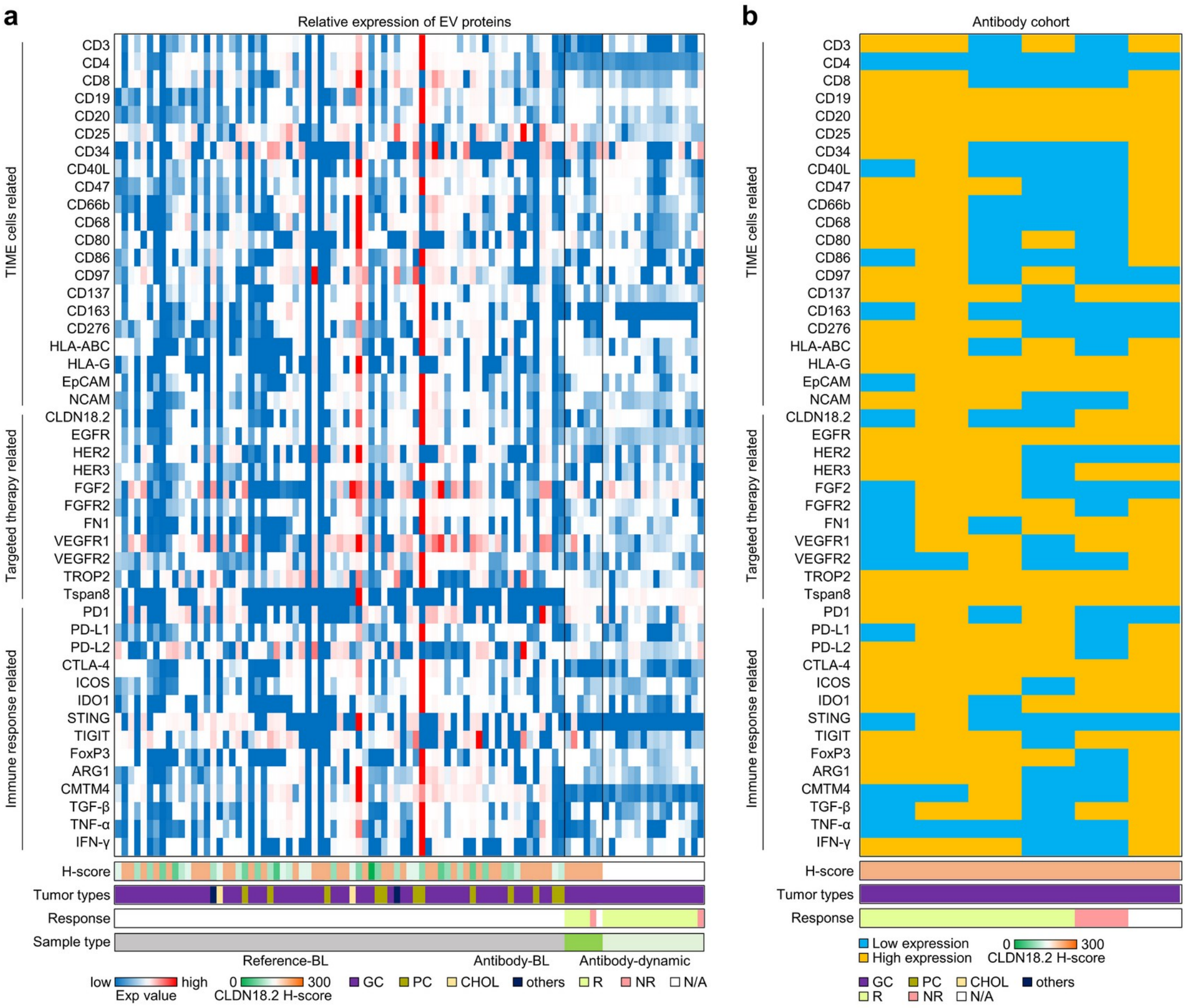

Supplementary Fig. 20. A relative EV-protein expression spectrum for antibody cohort and subsequent analysis. (a) Heatmap for relative expressions of 50 panel EV-proteins at all time points in reference and antibody cohort. **(b)** Each EV-proteins' expressions at BL were defined as "high" or "low" based on the median of reference cohort. The reference cohort comprised n = 71 independent untreated patients with gastrointestinal cancers, and the antibody cohort comprised n = 6 independent patients treated with Zolbetuximab plus CAPOX. For longitudinal analyses, n denotes available serial plasma samples collected from these patients; for baseline classification in panel b, n denotes independent patients.

Supplementary Tables

Supplementary Table 1. Reagent and sequence for qPCR analysis of CLDN18.2-targeted CAR-T cell expansion and persistence

qPCR Reagent	Sequence
CAR-CLDN18.2 FAM Probe	5’-CTGAGCAGCGTGACCGCCGC-3’
Forward Primer CAR-CLDN18.2	5’-TGGAGTGGATCGGCTACATC-3’
Reverse Primer CAR-CLDN18.2	5’-AGTAGTAGATGGCGGTGTCG-3’

Supplementary Table 2. COX multivariate analysis for the involvement of CLDN18.2 H-score, EV-CLDN18.2, EV-PD1 and EV-PD-L2 with CLDN18.2-CAR-T's PFS and OS in both discovery and validating cohorts.

	**Discovery cohort**				**Validating cohort**			
	**PFS**		**OS**		**PFS**		**OS**	
**Variates**	***P* value**	**Hazard Ratio (95% CI)**	***P* value**	**Hazard Ratio (95% CI)**	***P* value**	**Hazard Ratio (95% CI)**	***P* value**	**Hazard Ratio (95% CI)**
CLDN18.2 H-score	0.3385	1 (0.9934-1.002)	0.8127	1 (0.9949-1.004)	0.6271	1 (0.9916-1.0051)	0.3751	1 (0.9890-1.004)
EV-CLDN18.2	0.014	1.45 (1.0784-1.952)	0.0648	1.32 (0.9831-1.774)	0.001	4.78 (2.1418-10.6620)	0.001	3.44 (1.6434-7.209)
EV-PD1	0.0647	7.62 (0.8832-65.764)	0.0486	10.28 (1.0143-104.135)	0.4533	0.38 (0.0294-4.8316)	0.524	2.3 (0.1773-29.862)
EV-PD-L2	0.0882	0.96 (0.9225-1.006)	0.1778	0.96 (0.9119-1.017)	0.0062	0.92 (0.8624-0.9757)	0.0779	0.95 (0.8973-1.006)
