# Supplementary material for: Extracellular vesicle proteins monitor and interfere with CLDN18.2-targeted CAR-T cell and antibody therapies against gastrointestinal cancers
Source: Nat Commun. 2026 Sep 3;17:10502. doi: 10.1038/s41467-026-77132-9 (PMC13638995; doi:10.1038/s41467-026-77132-9)
Supplement: Supplementary file 2 [file 41467_2026_77132_MOESM2_ESM.pdf]

Description of Additional Supplementary Files

Supplementary Dataset 1: Demographic and clinicopathological characteristics, molecular biomarker status, treatment response, survival outcomes, metastasis status, and treatment information of patients in the discovery and validation cohorts receiving CLDN18.2-targeted CAR-Tcell therapy.

Supplementary Dataset 2: Demographic and clinicopathological characteristics, molecular biomarker status, treatment response, survival outcomes, and metastasis status of patients in the antibody cohort receiving zolbetuximab plus CAPOX.

Supplementary Dataset 3: Demographic and clinicopathological characteristics and molecular biomarker status of patients in the reference cohort.

Supplementary Dataset 4: List of antibodies used for the extracellular vesicle expression array, including the target molecules, suppliers, and catalogue numbers.

Supplementary Dataset 5: Extracellular vesicle protein expression profiles and clinical characteristics of patients in the discovery, validation, and antibody cohorts.

Supplementary Dataset 6: Demographic and clinicopathological characteristics, molecular biomarker status, treatment response, and survival outcomes of patients in the discovery and validation cohorts stratified by CPP score.

Supplementary Dataset 7: LC–MS-based protein abundance profiles from control and CLDN18.2-targeted proximity-labeling assays.

Supplementary Dataset 8: Demographic and clinicopathological characteristics, molecular biomarker status, treatment response, and survival outcomes of patients in the antibody cohort stratified by CPP score.
