# Supplementary material for: Extracellular vesicle proteins monitor and interfere with CLDN18.2-targeted CAR-T cell and antibody therapies against gastrointestinal cancers
Source: Nat Commun. 2026 Sep 3;17:10502. doi: 10.1038/s41467-026-77132-9 (PMC13638995; doi:10.1038/s41467-026-77132-9)
Supplement: Supplementary file 3 [file 41467_2026_77132_MOESM4_ESM.pdf]

nature portfolio

Corresponding author(s): Changsong Qi, Jian Li, Lin Shen, Cheng Zhang

Last updated by author(s): Jun 22, 2026

Reporting Summary

Nature Portfolio wishes to improve the reproducibility of the work that we publish. This form provides structure for consistency and transparency in reporting. For further information on Nature Portfolio policies, see our Editorial Policies and the Editorial Policy Checklist.

Statistics

For all statistical analyses, confirm that the following items are present in the figure legend, table legend, main text, or Methods section.

n/a	Confirmed	
☐	☒	The exact sample size (*n*) for each experimental group/condition, given as a discrete number and unit of measurement
☐	☒	A statement on whether measurements were taken from distinct samples or whether the same sample was measured repeatedly
☐	☒	The statistical test(s) used AND whether they are one- or two-sided<br>*Only common tests should be described solely by name; describe more complex techniques in the Methods section.*
☐	☒	A description of all covariates tested
☒	☐	A description of any assumptions or corrections, such as tests of normality and adjustment for multiple comparisons
☐	☒	A full description of the statistical parameters including central tendency (e.g. means) or other basic estimates (e.g. regression coefficient) AND variation (e.g. standard deviation) or associated estimates of uncertainty (e.g. confidence intervals)
☐	☒	For null hypothesis testing, the test statistic (e.g. *F*, *t*, *r*) with confidence intervals, effect sizes, degrees of freedom and *P* value noted<br>*Give P values as exact values whenever suitable.*
☒	☐	For Bayesian analysis, information on the choice of priors and Markov chain Monte Carlo settings
☒	☐	For hierarchical and complex designs, identification of the appropriate level for tests and full reporting of outcomes
☒	☐	Estimates of effect sizes (e.g. Cohen's *d*, Pearson's *r*), indicating how they were calculated

Our web collection on statistics for biologists contains articles on many of the points above.

Software and code

Policy information about availability of computer code

Data collection	Single-cell RNA libraries were sequenced by an Illumina NovaSeq 6000 sequencer with 150 bp paired-end reads. Raw gene expression matrices were generated for each sample by the Cell Ranger (Version 6.1.2) pipeline coupled with human reference version refdata-gex-GRCh38-2020-A. Filtered gene expression matrices were analyzed by R software (Version 4.1.2) with the Seurat package (Version 4.3.0.1). The percentage of mitochondrial transcripts for each cell (percent.mito) was calculated and added as metadata to the Seurat object. All samples were merged into one Seurat object using the merge function in Seurat. Data were filtered for cells (nfeatures>200) and mitochondrial genes (percent_mt<10%)..<br>Dimension reduction and unsupervised clustering were performed according to the standard workflow in Seurat. NormalizeData function was applied to normalize the single nucleus gene expression data, and we used 2000 genes with high cell-to-cell variation, which were calculated using the FindVariableFeatures function for further dimensionality reduction. Then, the effect of the percentage of mitochondrial gene count was regressed out by using the ScaleData function. A principal component analysis (PCA) matrix was calculated to reduce noise by using RunPCA, and thirty principal components were selected for downstream analysis. We performed batch correction by harmony R package (Version 0.1.1). Then Uniform Manifold Approximation and Projection (UMAP) and clustering were performed to identify clusters. Finally, we clustered cells using the FindNeighbors and FindClusters functions and performed non-linear dimensional reduction.<br>We used the FindAllMarkers function with the Wilcoxon rank-sum test to identify and annotate differentially expressed genes (DEGs). DEGs were filtered using an average log2 (fold change) of 0.25. The main cell types were annotated based on the expression pattern of DEGs and the well-known cellular markers from the literature. For trajectory inference analysis, monocle (Version 2.22.0) in R was used. Raw count data from the Seurat object was converted to a CellDataSet object using the newCellDataSet function in monocle. DEGs between celltypes (calculated using the Findallmarkers function in Seurat, sorted according to their avg_log2FC, and top 1000 were extracted as significantly different genes) were set as the ordering genes. The minimum spanning tree was constructed using the reduceDimensions function (reduction_method = "DDRTree").

Data analysis	Subgroup diversities was assessed by chi-square test/unpaired two-tailed two-sided t-test/two-way repeated-measures ANOVA. Consistency between indexes was assessed by Pearson correlation analysis. Survival proportions were assessed by Kaplan–Meier analysis paired with Log-rank test. A two-sided P<0.05 was considered statistically significant. Error bars were exhibited in form of mean ± standard deviation (SD). Analysis and graphing were powered by R 3.5.1/SPSS 21.0/GraphPad Prism 8/BioRender programs.

For manuscripts utilizing custom algorithms or software that are central to the research but not yet described in published literature, software must be made available to editors and reviewers. We strongly encourage code deposition in a community repository (e.g. GitHub). See the Nature Portfolio guidelines for submitting code & software for further information.

Data

Policy information about availability of data

All manuscripts must include a data availability statement. This statement should provide the following information, where applicable:

- Accession codes, unique identifiers, or web links for publicly available datasets
- A description of any restrictions on data availability
- For clinical datasets or third party data, please ensure that the statement adheres to our policy

All single-cell RNA-sequencing data generated by this study are available at https://www.cncb.ac.cn/ (accession No. PRJCA024021 and No. HRA006971)

Research involving human participants, their data, or biological material

Policy information about studies with human participants or human data. See also policy information about sex, gender (identity/presentation), and sexual orientation and race, ethnicity and racism.

Reporting on sex and gender	The human participants in this study were gastrointestinal cancer patients receiving CLDN18.2-CAR-T or CLDN18.2-antibody therapies. Sex and gender information were not considered by this study.
Reporting on race, ethnicity, or other socially relevant groupings	The human participants in this study were gastrointestinal cancer patients receiving CLDN18.2-CAR-T or CLDN18.2-antibody therapies. All patients are of Chinese ethnicity, and treated in Peking University Cancer Hospital & Institute.
Population characteristics	The human participants in this study were gastrointestinal cancer patients receiving CLDN18.2-CAR-T or CLDN18.2-antibody therapies. Clinicopathological information (primary location, lauren, stage, CLDN18.2-status, HER2-status, PD-L1-status, MSI/MMR-status, EBV-status, metastasis-status) and treatment information (regimens, responses, OS, PFS) of patients were summarized in supplementary tables.
Recruitment	GI cancer patients from two clinical trials, including 77 patients from a phase 1 clinical trial of CLDN18.2-targeted-CAR-T therapy (CT041-CG4006, NCT03874897) and 6 patients from an Phase 3 clinical trial of CLDN18.2-targeted-antibody therapy (GLOW, NCT03653507), were recruited in this study as two CAR-T cohorts and one antibody cohort. Another 71 untreated GI cancer patients served as a reference cohort. All patients were treated and recruited by Peking University Cancer Hospital & Institute.
Ethics oversight	All patient specimens and relevant clinical information were approved for research applications by the Institutional Ethics Committee of Peking University Cancer Hospital & Institute (2021KT79). Written informed consents were obtained from all donors.

Note that full information on the approval of the study protocol must also be provided in the manuscript.

Field-specific reporting

Please select the one below that is the best fit for your research. If you are not sure, read the appropriate sections before making your selection.

☒ Life sciences ☐ Behavioural & social sciences ☐ Ecological, evolutionary & environmental sciences

For a reference copy of the document with all sections, see nature.com/documents/nr-reporting-summary-flat.pdf

Life sciences study design

All studies must disclose on these points even when the disclosure is negative.

Sample size	Among patients enrolled in CLDN18.2-targeted-CAR-T trial (CT041-CG4006, NCT03874897), 77 with applicable plasma samples were included, in which 31 cases from the interim report as a retrospective discovery cohort, another 46 cases from the final report as a prospective validating cohort. For CLDN18.2-targeted-antibody trial (GLOW, NCT03653507), 6 patients from our center were retrospectively included as an antibody cohort. Plasma samples of another 71 untreated GI cancer patients served as a reference cohort.
Data exclusions	For all patients receiving CLDN18.2-CAR-T (CT041-CG4006 trial) or CLDN18.2-antibody Zolbetuximab (GLOW trial) treatment in Peking University Cancer Hospital & Institute, those without applicable plasma samples or those receiving placebo were excluded.
Replication	The CAR-T cells used for experiments were generated from replicate human donors. All animal experiments were performed with replicate mice for each groups.

Randomization	Participates were directly allocated into cohorts by their treatment regimens. Randomization is not relevant to this study.
Blinding	Participates were directly allocated into cohorts by their treatment regimens. Blinding is not relevant to this study.

Reporting for specific materials, systems and methods

We require information from authors about some types of materials, experimental systems and methods used in many studies. Here, indicate whether each material, system or method listed is relevant to your study. If you are not sure if a list item applies to your research, read the appropriate section before selecting a response.

Materials & experimental systems

n/a	Involved in the study
☐	☒ Antibodies
☐	☒ Eukaryotic cell lines
☒	☐ Palaeontology and archaeology
☐	☒ Animals and other organisms
☐	☒ Clinical data
☒	☐ Dual use research of concern
☒	☐ Plants

Methods

n/a	Involved in the study
☒	☐ ChIP-seq
☐	☒ Flow cytometry
☒	☐ MRI-based neuroimaging

Antibodies

Antibodies used

Antibodies for EV expression array
Alix, Santa, sc-53538; ARG1, Genetex, GTX634218; CD137, Sinobiological, 10041-RRP02; CD163, Genetex, GTX42365; CD19, Genetex, GTX42325; CD20, Sinobiological, 11007-MM01; CD25, Genetex, GTX18115; CD276, Sinobiological, 11188-MM06; CD3, Genetex, GTX628462; CD34, Sinobiological, 10103-MM13; CD4, Genetex, GTX17283; CD40L, Sinobiological, 10239-R301; CD47, Sinobiological, 12283-R006; CD63, Genetex, GTX28219; CD66b, Sinobiological, 11729-R013; CD68, Sinobiological, 11192-R039; CD8, Genetex, GTX83296; CD80, Sinobiological, 10698-MM01; CD81, Santa, sc-166029; CD86, Sinobiological, 10699-MM02; CD9, Santa, sc-13118; CD97, Genetex, GTX637674; Claudin 18.2, Novoprotein, NC004; CMTM4, Genetex, GTX34320; CTLA-4, Invitrogen, MA5-30308; EGFR, Sinobiological, 10001-MM01; EpCAM, Sinobiological, 10694-T52; FGF-2, Genetex, GTX83243; FGFR-2, Invitrogen, MA5-30433; FN1, Genetex, GTX60571; FoxP3, Abcam, ab36607; HER2, Biolegend, 324402; HER3, Sinobiological, 10201-MM01; HLA-ABC, Genetex, GTX42432; HLA-G, Genetex, GTX24570; ICOS, Sinobiological, 13338-1-AP; IDO1, Genetex, GTX634652; IFN-γ, Genetex, GTX15624; NCAM, Sinobiological, 10673-MM05; PD1, Abcam, ab52587; PDL1, Proteintech, 66248-1-Ig; PD-L2, Invitrogen, 14-5888-82; STING (TMEM173), Abways, CY7204; TGF-beta 1, Sinobiological, 10804-R016; TIGIT, Abcam, ab243903; TNF-α, Genetex, GTX26671; TROP2, Sinobiological, 10428-MM02; Tspan8, Santa, sc-293317; VEGFR1, Invitrogen, BMS196; VEGFR2, Invitrogen, 14-5821-82

Immunogold labelling assay
CD81, Santa, sc-166029; CD9, Santa, sc-13118; CLDN18.2, Novoprotein, NC004; Zolbetuximab, MCE, HY-P99058; PD1, Abcam, ab52587; PD-L2, Invitrogen, 14-5888-82; rabbit IgG, Sigma-Aldrich, G7402; mouse IgG, Sigma-Aldrich, G7652

WB and flow cytometry
CLDN18, Abcam, ab203563; CD81, Abcam, ab79559; CD81, Genetex, GTX101766; CD9, Abcam, ab223052; CD9, Abcam, ab307085; CD63, Abcam, ab216130; CD63, Genetex, GTX28219; Calnexin, Abcam, ab22595; GAPDH, Cell Signaling Technology, #2118; a-SMA, Cell Signaling Technology, #19245; FAP, Cell Signaling Technology, #52818; Cleaved Caspase 3, Cell Signaling Technology, #9664; Bcl-2, Cell Signaling Technology, #15071; PD-1, Biolegend, 367404; TIM3, Biolegend, 345012; LAG3, Biolegend, 369310; TIGIT, biolenged, 372710

Biotin-labeled antibody backfill assay
Zolbetuximab, MCE, HY-P99058

IMC labeling
CD3, Abcam, ab251607; GZMB, Abcam, ab255868; PD-1, Abcam, ab201811; S100A4, Abcam, ab220213; PANCK, Abcam, ab308263

Validation

Antibodies for EV expression array
Alix, Santa, sc-53538
https://www.scbt.com/zh/p/alix-antibody-3a9
ARG1, Genetex, GTX634218
https://www.genetex.cn/Product/Detail/Arginase-1-antibody-GT5811/GTX634218
CD137, Sinobiological, 10041-RP02
https://cn.sinobiological.com/antibodies/human-cd137-10041-rp02
CD163, Genetex, GTX42365
https://www.genetex.cn/Product/Detail/CD163-antibody-EDHu-1/GTX42365
CD19, Genetex, GTX42325
https://www.genetex.cn/Product/Detail/CD19-antibody-LE-CD19/GTX42325
CD20, Sinobiological, 11007-MM01:
https://cn.sinobiological.com/antibodies/human-cd20-11007-mm01
CD25, Genetex, GTX18115

https://www.genetex.cn/Product/Detail/IL2-Receptor-alpha-antibody-IL2RA-2395/GTX18115
CD276, Sinobiological, 11188-MM06
https://cn.sinobiological.com/antibodies/human-b7-h3-11188-mm06
CD3, Genetex, GTX628462
https://www.genetex.cn/Product/Detail/CD3-antibody-GT0013/GTX628462
CD34, Sinobiological, 10103-MM13
https://cn.sinobiological.com/antibodies/cd34-10103-mm13
CD4, Genetex, GTX17283
https://www.genetex.cn/Product/Detail/CD4-antibody-8G1B12/GTX17283
CD40L, Sinobiological, 10239-R301
https://cn.sinobiological.com/antibodies/human-cd40-ligand-10239-r301
CD47, Sinobiological, 12283-R006
https://cn.sinobiological.com/antibodies/human-cd47-12283-r006
CD63, Genetex, GTX28219
https://www.genetex.cn/Product/Detail/CD63-antibody-MEM-259/GTX28219
CD66b, Sinobiological, 11729-R013
https://cn.sinobiological.com/antibodies/human-cd66b-11729-r013
CD68, Sinobiological, 11192-R039
https://cn.sinobiological.com/antibodies/human-cd68-11192-r039
CD8, Genetex, GTX83296
https://www.genetex.cn/Product/Detail/CD8-antibody-RAVB3/GTX83296
CD80, Sinobiological, 10698-MM01
https://cn.sinobiological.com/antibodies/human-b7-1-10698-mm01
CD81, Santa, sc-166029
https://www.scbt.com/zh/p/cd81-antibody-b-11
CD86, Sinobiological, 10699-MM02
https://cn.sinobiological.com/antibodies/human-cd86-10699-mm02
CD9, Santa, sc-13118
https://www.scbt.com/zh/p/cd9-antibody-c-4
CD97, Genetex, GTX637674
https://www.genetex.cn/Product/Detail/CD97-antibody-HL1925/GTX637674
Claudin 18.2, Novoprotein, NC004
https://www.novoprotein.com.cn/product-detail?productNumber=NC004
CMTM4, Genetex, GTX34320
https://www.genetex.cn/Product/Detail/CMTM4-antibody/GTX34320
CTLA-4, Invitrogen, MA5-30308
https://www.thermofisher.cn/cn/zh/antibody/product/CTLA-4-Antibody-clone-06-Monoclonal/MA5-30308
EGFR, Sinobiological, 10001-MM01
https://cn.sinobiological.com/antibodies/human-egfr-10001-mm01
EpCAM, Sinobiological, 10694-T52
https://cn.sinobiological.com/antibodies/human-epcam-10694-t52
FGF-2, Genetex, GTX83243
https://www.genetex.cn/Product/Detail/FGF2-antibody-2H5G2C11/GTX83243
FGFR-2, Invitrogen, MA5-30433
https://www.thermofisher.cn/cn/zh/antibody/product/FGFR2-Antibody-clone-016-Recombinant-Monoclonal/MA5-30433
FN1, Genetex, GTX60571
https://www.genetex.cn/Product/Detail/Fibronectin-antibody-2F4G2/GTX60571
FoxP3, Abcam, ab36607
https://www.abcam.cn/products/primary-antibodies/foxp3-antibody-2a11g9-ab36607.html
HER2, Biolegend, 324402
https://www.biolegend.com/en-us/products/purified-anti-human-cd340-erbb2-her-2-antibody-3764
HER3, Sinobiological, 10201-MM01
https://cn.sinobiological.com/antibodies/human-her3-erbb3-10201-mm01
HLA-ABC, Genetex, GTX42432
https://www.genetex.cn/Product/Detail/HLA-Class-I-antibody-W6-32/GTX42432
HLA-G, Genetex, GTX24570
https://www.genetex.cn/Product/Detail/HLA-G-antibody-MEM-G-11/GTX24570
ICOS, Proteintech, 13338-1-AP
https://www.ptgcn.com/products/ICOS-Antibody-13338-1-AP.htm
IDO1, Genetex, GTX634652
https://www.genetex.cn/Product/Detail/IDO1-antibody-GT273/GTX634652
IFN-γ, Genetex, GTX15624
https://www.genetex.cn/Product/Detail/Interferon-gamma-antibody-2G1/GTX15624
NCAM, Sinobiological, 10673-MM05
https://cn.sinobiological.com/antibodies/human-ncam-10673-mm05
PD1, Abcam, ab52587
https://www.abcam.com/en-us/products/primary-antibodies/pd1-antibody-nat105-ab52587
PDL1, Proteintech, 66248-1-Ig
https://www.ptgcn.com/products/PD-L1-CD274-Antibody-66248-1-Ig.htm
PD-L2, Invitrogen, 14-5888-82
https://www.thermofisher.cn/cn/zh/antibody/product/CD273-B7-DC-Antibody-clone-MIH18-Monoclonal/14-5888-82
STING (TMEM173), Abways, CY7204
https://www.abways.com/ProductsStd/CY7204.html
TGF-beta 1, Sinobiological, 10804-R016
https://www.sinobiological.com/category/tgf-beta-1
TIGIT, Abcam, ab243903

https://www.abcam.com/en-us/products/primary-antibodies/tigit-antibody-blr047f-bsa-free-ab243903
TNF-α, Genetex, GTX26671
https://www.genetex.cn/Product/Detail/TNF-alpha-antibody/GTX26671
TROP2, Sinobiological, 10428-MM02
https://www.sinobiological.com/antibodies/human-trop2-10428-mm02
Tspan8, Santa, sc-293317
https://www.scbt.com/zh/p/tspan8-antibody-1e5
VEGFR1, Invitrogen, BMS196
https://www.thermofisher.cn/cn/zh/antibody/product/VEGF-Receptor-1-Flt1-Antibody-clone-Flt-19-Monoclonal/BMS196
VEGFR2, Invitrogen, 14-5821-82
https://www.thermofisher.cn/cn/zh/antibody/product/CD309-FLK1-Antibody-clone-Avas12a1-Monoclonal/14-5821-82

Immunogold labelling assay
CD81, Santa, sc-166029
https://www.scbt.com/zh/p/cd81-antibody-b-11
CD9, Santa, sc-13118
https://www.scbt.com/p/cd9-antibody-c-4
CLDN18.2, Novoprotein, NC004
https://www.novoprotein.com/product-detail?productNumber=NC004
Zolbetuximab, MCE, HY-P99058
https://www.medchemexpress.cn/zolbetuximab.html
PD1, Abcam, ab52587
https://www.abcam.com/en-us/products/primary-antibodies/pd1-antibody-nat105-ab52587
PD-L2, Invitrogen, 14-5888-82
https://www.thermofisher.cn/cn/zh/antibody/product/CD273-B7-DC-Antibody-clone-MIH18-Monoclonal/14-5888-82
rabbit IgG, Sigma-Aldrich, G7402
https://www.sigmaaldrich.cn/CN/zh/search/g7402?
focus=products&page=1&perpage=30&sort=relevance&term=G7402&type=product
mouse IgG, Sigma-Aldrich, G7652
https://www.sigmaaldrich.cn/CN/zh/search/g7652?
focus=products&page=1&perpage=30&sort=relevance&term=G7652&type=product
WB and flow cytometry
CLDN18, Abcam, ab203563
https://www.abcam.com/en-us/products/primary-antibodies/claudin18-antibody-34h14l15-ab203563
CD81, Abcam, ab286173
https://www.abcam.cn/products/primary-antibodies/cd81-antibody-a1504-ab286173.html
CD9, Abcam, ab223052
https://www.abcam.com/en-us/products/primary-antibodies/cd9-antibody-ab223052
Calnexin, Abcam, ab22595
https://www.abcam.com/en-us/products/primary-antibodies/calnexin-antibody-er-marker-ab22595
GAPDH, Cell Signaling Technology, #2118
https://www.cellsignal.com/products/primary-antibodies/gapdh-14c10-rabbit-mab/2118
a-SMA, Cell Signaling Technology, #19245
https://www.cellsignal.com/products/primary-antibodies/a-smooth-muscle-actin-d4k9n-xp-rabbit-mab/19245
FAP, Cell Signaling Technology, #52818
https://www.cellsignal.com/products/primary-antibodies/fap-f1a4g-rabbit-mab/52818
Cleaved Caspase 3, Cell Signaling Technology, #9664
https://www.cellsignal.com/products/primary-antibodies/cleaved-caspase-3-asp175-5a1e-rabbit-mab/9664
Bcl-2, Cell Signaling Technology, #15071
https://www.cellsignal.com/products/primary-antibodies/bcl-2-124-mouse-mab/15071
PD-1, Biolegend, 367404
https://www.biolegend.com/en-gb/products/pe-anti-human-cd279-pd-1-antibody-12183
TIM3, Biolegend, 345012
https://www.biolegend.com/en-gb/products/apc-anti-human-cd366-tim-3-antibody-8302
LAG3, Biolegend, 369310
https://www.biolegend.com/en-gb/products/pe-cyanine7-anti-human-cd223-lag-3-antibody-13551
TIGIT, biolenged, 372710
https://www.biolegend.com/en-gb/products/brilliant-violet-421-anti-human-tigit-vstm3-antibody-13935

Biotin-labeled antibody backfill assay
Zolbetuximab, MCE, HY-P99058
https://www.medchemexpress.cn/zolbetuximab.html

IMC labeling
CD3, Abcam, ab251607
https://www.abcam.cn/products/primary-antibodies/cd3-epsilon-antibody-cal57-bsa-and-azide-free-ab251607.html
GZMB, Abcam, ab255868
https://www.abcam.cn/products/primary-antibodies/granzyme-b-antibody-epr22645-206-bsa-and-azide-free-ab255868.html
PD-1, Abcam, ab201811
https://www.abcam.cn/products/primary-antibodies/pd1-antibody-nat105-bsa-and-azide-free-ab201811.html
S100A4, Abcam, ab220213
https://www.abcam.cn/products/primary-antibodies/s100a4-antibody-epr146392-bsa-and-azide-free-ab220213.html
PANCK, Abcam, ab308263
https://www.abcam.cn/products/primary-antibodies/pan-cytokeratin-antibody-epr28285-45-bsa-and-azide-free-ab308263.html

Eukaryotic cell lines

Policy information about cell lines and Sex and Gender in Research

Cell line source(s)	Human GC cell lines AGS/HGC27, human cell line HEK293F and mouse colorectal cancer cell line CT26 were from ATCC (Manassas, VA) or National Infrastructure of Cell Line Resource (Beijing, China).
Authentication	All cell lines were authenticated by short tandem repeat profiling.
Mycoplasma contamination	All cell lines were tested negative for mycoplasma contamination.
Commonly misidentified lines (See ICLAC register)	Not involved.

Animals and other research organisms

Policy information about studies involving animals; ARRIVE guidelines recommended for reporting animal research, and Sex and Gender in Research

Laboratory animals	NCG mice (HFK Biotechnology, Beijing, China), BALB-C nude mice (Vital River Laboratories, Beijing, China), and BALB-C mice (Vital River Laboratories) were used in this study.
Wild animals	No wild animals were used in this study.
Reporting on sex	Only female mice were used in this study, because female mice are more docile, making them easier to handle and manipulate during experimental procedures.
Field-collected samples	No field-collected samples were used in this study.
Ethics oversight	All animal experiments were performed under the permission of the Animal Experimentation Ethics Committee of Peking University Cancer Hospital (EAEC 2023-14).

Note that full information on the approval of the study protocol must also be provided in the manuscript.

Clinical data

Policy information about clinical studies

All manuscripts should comply with the ICMJE guidelines for publication of clinical research and a completed CONSORT checklist must be included with all submissions.

Clinical trial registration	This study used clinical data from CT041-CG4006 (NCT03874897) and GLOW (NCT03653507) trials.
Study protocol	No clinical interventions were involved in this study.
Data collection	All clinical data used in this study were obtained from the medical records of clinical trials, after being approved for research applications by the Institutional Ethics Committee of Peking University Cancer Hospital & Institute. The end point of follow-up was Feb 2023.
Outcomes	The antitumor efficacy was measured according to Response Evaluation Criteria in Solid Tumors (RECIST) version 1.1 as complete response (CR)/partial response (PR)/stable disease (SD)/progressed disease (PD). Objective response rate (ORR), disease control rate (DCR), progression-free survival (PFS), overall survival (OS) and duration of response (DOR) were followed-up. Patients who reached CR/PR, or maintained SD≥6 months were defined as responders, others were defined as nonresponders. Progression of responders were defined as acquired resistance.

Plants

Seed stocks	Not involved.
Novel plant genotypes	Not involved.
Authentication	Not involved.

Flow Cytometry

Plots

Confirm that:

☒ The axis labels state the marker and fluorochrome used (e.g. CD4-FITC).

☒ The axis scales are clearly visible. Include numbers along axes only for bottom left plot of group (a 'group' is an analysis of identical markers).

☒ All plots are contour plots with outliers or pseudocolor plots.

☒ A numerical value for number of cells or percentage (with statistics) is provided.

Methodology

Sample preparation	Fresh cells were collected and washed with DPBS (Dulbecco's Phosphate-Buffered Saline), resuspend with DPBS into 1×10^6 cells/mL, treated with 5 µL corresponding antibodies for each test, stained at room temperature in dark, administrated with DPBS wash and filtering, and proceeded for detection with a flow cytometry machine (CytoFlex, Beckman).
Instrument	CytoFlex, Beckman
Software	CytExpert
Cell population abundance	The collected cells are directly processed for flow cytometry, with a cell abundance of 100%.
Gating strategy	For collected cells, ensuring a viability higher than 95%. Label T cells for CD3, then stain CD3-positive T cells for PD-1, TIM-3, LAG-3, and TIGIT. The first gate is for FSC/SSC, where the voltage is adjusted to ensure that all target cell populations are within the field of view before gating. The second gate is for CD3-positive cells, with unstained T cells serving as the negative control to determine the position of the CD3-positive gate. Based on the positivity in the second gate, adjust the voltage to separate the target cell population into two groups. The positive group is designated as the third gate, with fluorescence intensity values above 10E4 considered positive.

☒ Tick this box to confirm that a figure exemplifying the gating strategy is provided in the Supplementary Information.
