# Supplementary material for: Extracellular vesicle proteins monitor and interfere with CLDN18.2-targeted CAR-T cell and antibody therapies against gastrointestinal cancers
Source: Nat Commun. 2026 Sep 3;17:10502. doi: 10.1038/s41467-026-77132-9 (PMC13638995; doi:10.1038/s41467-026-77132-9)
Supplement: Supplementary file 4 [file 41467_2026_77132_MOESM5_ESM.pdf]

nature portfolio

Peer Review File

Extracellular Vesicle Proteins Monitor and Interfere with CLDN18.2-Targeted CAR-T Cell and Antibody Therapies Against Gastrointestinal Cancers

Corresponding Author: Professor Cheng Zhang

This file contains all reviewer reports in order by version, followed by all author rebuttals in order by version.

Version 0:

Reviewer comments:

Reviewer #1

(Remarks to the Author)
This manuscript investigates the role of extracellular vesicle in mediating the efficacy of CLDN18.2-targeted CAR-T cell and antibody therapies against gastrointestinal cancers. The study is ambitious in scope and employs sophisticated multi-omics techniques.
Strengths:
• Novelty: The identification of EV-derived proteins as biomarkers predictive of treatment response is a significant contribution to the field. The CPP-score, combining the expression of several key proteins, offers a robust and potentially clinically useful predictive biomarker.
• Comprehensive Methodology: The study uses a robust and comprehensive methodology including multi-omics analyses, functional assays, and in vivo experiments. The use of both CAR-T and antibody therapy arms strengthens the findings.
• Mechanistic Insights: The study makes attempts to elucidate the mechanisms by which EV-proteins interfere with therapy efficacy, implicating CAF activation and T cell exhaustion. This is a valuable step towards therapeutic intervention.
• Clinical Relevance: The authors directly translate their preclinical findings into clinical implications by exploring plasma exchange and intraperitoneal infusion strategies for therapeutic sensitization.

(Abstract):
• "but biomarkers for long-term benefit and therapeutic sensitization strategies are needed" —> "but biomarkers predictive of long-term benefit and therapeutic sensitization strategies are needed."
• "We depicted EV-protein expression profiles" —> "We determined EV-protein expression profiles" or "We characterized EV-protein expression profiles".
• "then performed multi-omics, functional and mechanistic investigations" —> "and then performed multi-omics, functional, and mechanistic investigations". (Improved flow)
(Introduction):
• "In healthy gastric tissues, CLDN18.2 expression is limited to differentiated epithelial cells in the mucosa." —> "In healthy gastric tissue, CLDN18.2 expression is restricted to differentiated epithelial cells of the mucosa." (More precise)
• "Due to its prevalence and tumor-specific presence," —> "Due to its tumor-specific expression and prevalence," (Improved flow)
• "efficacy of CAR-T reported in solid tumors" —> "efficacy reported for CAR-T therapy in solid tumors". (Clarified)
• "However, despite these advances, there is an urgent need for proper biomarkers instructing & monitoring CLDN18.2-targeted therapies' efficacy," —> "However, despite these advances, there is an urgent need for biomarkers to predict and monitor the efficacy of CLDN18.2-targeted therapies," (More concise and grammatically correct)
Page 6 (Introduction):
• "we uncovered the roles and mechanisms of key EV-proteins in modulating TIME through influencing CLDN18.2-CAR-T cells, anti-CLDN18.2-antibody, cancer associated fibroblasts (CAFs) and T helper cell balance, thus interfering with the efficacy of CLDN18.2-targeted-CAR-T/antibody therapies." —> "We elucidated the roles and mechanisms of key EV proteins in modulating the tumor immune microenvironment (TIME) by influencing CLDN18.2-CAR-T cells, anti-CLDN18.2 antibodies, cancer-associated fibroblasts (CAFs), and T helper cell balance, thus interfering with the efficacy of CLDN18.2-targeted CAR-T and antibody therapies." (Improved flow and clarity. Rephrased for conciseness and accuracy)

A thorough grammatical and typographical review of the entire manuscript is recommended before re-submission. This is a potentially impactful study that offers a novel approach to improve the efficacy of CLDN18.2-targeted therapies.

Reviewer #2

(Remarks to the Author)
In their study, Qi et al's investigated the role of CLDN18.2-derived EVs as 1) a blood biomarker of resistance to CAR-T therapy and CLDN18.2-targeting antibodies and 2) a mediator of CAR-T dysfunction by inducing CAR-T exhaustion through binding of EV-bound CLDN18.2 to the T-cell derived CAR and by promoting the release of IL-6 by CAFs.

The manuscript contains an impressive amount of results and discovery+validation patient cohorts. With 8 main figures, 9 extended figures, 10 Supplementary figures and 8 supplementary tables, this paper is difficult to read and at each sentence, we have to navigate through the paper to find the right figure panel. Separating the story in two high-impact papers should be considered. Most figures have 10+ panels which make them difficult to interpret. Some panels are very small and the statistical significance not always readable. Repetitive key panels could be kept in main figures and the remaining in Supplementary Data.

The significance of the results presented is high because it "solves" several concepts in the EV biology that are still uncleared.
The authors could acknowledge and discuss their data with a recent study from the Guo's group showing the biological and clinical role of MSLN+-PD-L1+EVs in promoting resistance to MSLN CAR-T (Zhong W et al, Cancer Research, 2023). The study includes several innovative methods and all the results seem to align well the hypothesis. It is well recognized that the study of EVs not only as blood biomarker and as promoter of therapeutic resistance is challenging due to the difficulty of quantifying EV subsets with high reproducibility and isolating them with high purity. Given the quality of the results obtained in their study, the authors are demanded to provide more details on the procedures and methods used in their study. In addition, some key experiments require technical validation and appropriate controls.

My comments will be divided in three major studies conducted in this manuscript:
Part 1: Identification of CLDN18.2/PD-1/PD-L2 EVs as circulating biomarkers of response to CLDN18.2 CAR-T therapy
Elevated plasma concentrations of CLDN18.2-EVs are associated with shorter clinical outcome in response to CAR-T which validate a prior report with MSLN-EVs and MSLN-CAR-T therapy (Zhong et al, in Cancer Research 2023). To that end, the authors employ a EV microarray similar to what has been originally described by Jorgensen M et al, JEV, 2013. The authors should cite the original paper to support feasibility of the EV array. Most clinical findings in this paper origin from the EV array data. Given the relevance and performance of this technique presented herein, the authors should address the concerns below and provide raw EV array data for all measurements in Supplementary files.
1) The protocol to isolate EVs from conditioned media and plasma is odd. The authors state they centrifuge at 15,000 x g to pellet EVs. At such speed, only low abundant large EVs > 200 nm are likely to pellet and a higher centrifugation speed is needed to isolate smaller EVs. Centrifugation alone has been shown to have low EV purity due to the co-isolation of aggregates, soluble factors and lipoproteins. Use of size-based methods combined with centrifugation has been shown to improve EV purity. The authors should address this as it is unclear whether EV array measured EVs only or EVs and soluble proteins.
There are several methodological details missing in the plasma EV isolation protocol. The authors should use the MIBlood-EV tool to report their technical procedures (Lucien F et al, JEV, 2023).
2) For the EV array, the authors claim they use 10 μL unpurified plasma sample diluted (1:10), in other words 1 microliter of plasma. It seems a very low volume to have enough sensitivity for detecting double-positive EVs (target + CD81/CD9/CD63/ALIX). In their original study, Jorgensen et al, showed that 1 microliters of plasma can be enough to detect signal over noise but there is a lot variability between patients and some may be below the LoD and LoQ. The assays conducted do not seem to include positive and negative controls.
Importantly, LoD for markers tested is not provided. While it could be difficult to demonstrate the linear range of detection and quantification of all markers, it should be presented for the key markers identified such as CLDN18.2, PD-L1 and PD-1. Interestingly, the panel of markers used include non-surface bound proteins and intravesicular proteins such as FOXP3, STING or ARG1. It is unclear how the authors have been able to capture EVs using intravesicular targets.
The authors should also provide how they determine low and high cutoffs for KM curves.
In Fig1d, Extended Figure 2k, the authors claim that the accuracy of the EV array is proven due to the correlation between EV array and ELISA for CLDN18.2-EV quantification. A correlation coefficient of 0.66 and 0.70 is not impressive and does not convince on the accuracy of the EV array vs ELISA. Visually, the coefficient of determination would be very low, thereby not supporting the authors' claim. No detailed methodology for the ELISA is provided.
3) Some figures lack statistical analysis. In Figure 1f and 1g, p value and statistical approach is not provided which precludes the identification of potential biomarker of response to CAR-T.
4) Gating strategy for flow cytometry analysis should be provided. No methodological details on PBMC isolation, staining and flow cytometry acquisition and data analysis are provided. Antibody concentrations and fluorophores used should be provided.

Part 2: CLDN18.2 EVs interacts with CAR and induces CAR-T dysfunction:
1) At the conclusion of their first part (Figure 8), the authors state: "CPP-score remained stable throughout CLDN18.2-CAR-T treatment (Extended Data Fig. 3j), while dynamic CAR-T cell copies (Supplementary Table S5) were lower for patients with high baseline-EV-CLDN18.2/EV-PD1/CPP-score and higher for patients with high baseline-EV-PD-L2 (Extended Data Fig. 3k), indicating EV-CLDN18.2/EV-PD1 reduce CAR-T cell content, while EV-PD-L2 maintains it." With the correlative data presented, the association of EV dynamics with response to CAR-T cells does not mean that EVs are directly impacting on

CAR-T efficacy. The authors should rephrase their conclusion.
Did the authors look at the association of radiographic tumor burden with plasma CLDN18.2 EV concentration and response to CAR-T? It is possible that poor PFS/OS is found in patients high baseline tumor burden. Does the multivariate analysis of CLDN18.2 and outcome included radiographic tumor burden?
In Supplementary Table S6, the authors claim that EV markers are independent prognostic markers at baseline of CAR-T response. However, p values are not showing statistical significance for all three markers, especially with OS as endpoint. CLDN18.2 EV seems more consistent. The authors should revise accordingly.
In the Page 10, the authors claim that: "These data indicate that CPP-score reflects higher tumor burden and more immunosuppressive
TIME.". Is it possible the authors refer to "tumor mutational burden" rather than "tumor burden" since they analyze CNV, Myc amplification...
2) In Figure 3a, the authors show that CAR-T cells are positive for PKH26-labeled EVs. Negative control is CAR-T alone but given that PKH26 form aggregates and false positive signals, PKH26 dye in medium should be used as negative control. Methodology mentions purification of EVs post-PKH26 labeling using an EV purification kit from Echobiotech). More details on the purification kit is needed to evaluate the purity post-PKH26 labeling.
Importantly, there is no mention of the EV-CART ratio used and co-culture duration in uptake and functional assays. Are they representative of what is found in patients?
3) In Figure 3i, 3j, Extended Fig 7, data show 1 replicate from 1 experiment which is underpowered to draw any conclusions.

Part3: CLDN18.2 EVs interacts with CAFs promoting the release of IL-6 blocking CAR-T efficacy:
1) The concerns for the PKH26 experiment described previously apply here. Co-culture details (time and EV-cell ratio) must be provided.
2) The authors had a good idea to use a proximity labeling system (TurboID) to identify interacting partners of CLDN18.2-expressing tumor cells in vivo. However, there is a lack of methodological details on the data acquisition and analysis by mass spectrometry. No validation is provided for the surface expression of engineered proteins in tumor cells. The number of samples included in the experiment is unknown. The authors do not provide any negative control for the proximity labeling in vivo. Mass spectrometry data obtained from proximity labeling with statistical analysis should be provided in Supplementary files.
3) The authors identify S100A4 as a CAF-associated marker highly present in tumors with CLDN18.2 oe. The authors should provide a more detailed analysis of their proximity labeling data such as number of proteins detected in ctrl and oe conditions, differential expression analysis and pathway enrichment analysis. Was S100A4 the only CAF-derived interaction of CLDN18.2 tumor cells? What was the rationale to select S100A4? The authors recently published the same experiment in 2024 https://biosignaling.biomedcentral.com/articles/10.1186/s12964-023-01406-8 and it is unclear what is different between the two proximity labeling experiments. In this submitted manuscript, it seems to be a reproduction of the data previously generated.
4) Did they authors attempt to validate the interaction of CLDN18.2 EVs with S100A4 in vitro?
5) In Figure 5, the authors show that CLDN18.2 EVs prevent CAR-T therapy in vivo. How did the authors measure EV-CLDN18.2 in plasma of mice treated with CLDN18.2 protein? How did they rule out the detection of soluble proteins?
6) In Figure 5b, the authors showed that CLDN18.2-EVs are higher in plasma of mice harboring CLDN18.2 positive xenografts compared to those with negative expression. It is different from patient data showing no correlation between CLDN18.2 EVs and tissue CLDN18.2 expression (Fig.1). The authors should discuss this discrepancy. Is there an association of plasma levels of CLDN18.2-EVs and tumor volume in PDX models?
6) The authors use 200 micrograms of EVs or 10 ug of soluble proteins twice a week in their in vivo experiments. It is a huge amount probably in supra physiological levels compared to circulating CLDN18.2 EV levels in patients. What is the lowest concentration to significantly block CAR-T efficacy? The authors should discuss this.
7) To prevent the activity of EV-CLDN18.2, the authors ingeniously employed a plasma exchange model. Briefly, 300 microliters of blood was collected retro-orbitally from a CLDN18.2-positive xenograft was centrifuged to remove plasma and purified plasma was mixed with plasma from a CLDN18.2 negative xenograft or tumor-free mice and injected tail vein. It was performed prior CAR-T treatment and biweekly after treatment. Each PE cycle corresponds to a change of ±15% of total blood. While the approach is interesting, there is no prior literature or technical validation of this PE model. Changes in circulating levels of CLDN18.2 pre and post-infusion would be important to support the efficacy of PE.
8) Figures 7d, 7k, Suppl Fig8 show 1 replicate from 1 experiment which is underpowered to draw any conclusions.

Reviewer #3

(Remarks to the Author)
In this manuscript, Qi et al. investigate the effect of extracellular vesicle (EV) proteins on the function of CLDN18.2 CAR-T cells. By profiling EV proteins in patients with gastrointestinal cancers receiving CLDN19.2 therapy or Zolbetuximab, they find that protein expression levels of CLDN18.2/PD1/PD-L2 correlate with patient responses to therapy. The authors demonstrate that extracellular vesicles containing CLDN18.2 protein exhaust CAR-T cells in vitro and lead to diminished efficacy in tumor models. Moreover, several different strategies to counteract EV proteins enhance therapeutic efficiency in mouse models.

The article is well presented and contains an impressive amount of data, ranging from patient data to sophisticated biochemical assays. I think the article should be considered for publication at Nature Communications, yet it would first need to address some concerns. Probably owing to the wide range of techniques employed, I think some key findings lack the sufficient depth and replicates. I detail my concerns below:

- The article is novel since this is the first time, as far as I know, where EV proteins are monitored and measured in patients

received CAR T cell therapy. Nonetheless, the detrimental effect of tumor-derived exosomes in CAR T cell functionality has already been described at least in two previous articles: Ukrainskaya et al. Scientific Reports 2023 and Zhong et al. Cancer Research 2023. The authors should cite these two articles, which already showed that tumor antigens in EVs are capable of driving CAR-T cell exhaustion and reduction in cytotoxic capacity.

- Correlation between levels of protein tissue expression and protein in extracellular vesicles. The authors show that EV CD19 and CLDN18.2 protein levels do not correlate with tumor tissue levels. On the other hand, in mice the authors observe higher EV-CLDN18.2 in mice with CLD18.2 + tumors. Altogether, this data is difficult to interpret.

One plausible explanation is that CLDN18.2 from healthy tissue also contribute to EV-CLDN18.2 in patients. The authors should measure levels of CLDN18.2 in healthy patients and compare them to the current data) patients with low or high CLDN18.2 tumors).

- Analysis of cytotoxic killing of HGC27-CDL18.2-oe (Figure 3m). The authors cannot conclude that cytotoxicity was impaired from this experiment, the wording must be changed. There is a lot of killing even when treated with EV-CLDN18.2. The axis used generates confusion, please change to a more standard axis (perhaps with a log10 scale). In addition, please explain better in the methods section how this experiment was performed.

This is a key experiment because it shows how killing functionality is affected by EVs. Please perform this experiment using different tumor lines, at different E:T ratios and using either pre-treatment of T cells with EV-CLDN18.2 or adding them during the co-culture. Authors should consider re-challenging the T cells with tumor to see better differences between groups. Likely the exhausted phenotype will become more apparent upon tumor rechallenge. As an example, see Figure 5 from Ukrainskaya et al. 2023.

- Effect of EV-CLDN18.2 on CAFs.
a) Substitute Figure 4b with a barplot showing biological replicates and assessing significance to be able to demonstrate CXCL8 and IL6 upregulation.
b) Please measure IL-6 and CXCL8 secretion in CAFs +/- S100A4 Knock down, treated or not with control of CLDN18.2 EV. Ideally, also perform the experiment using CRISPR knock-outs or using multiple shRNA for knocking down S100A4.
c) Is the effect on CAFs also observed with proCLDN18.2?

- In vivo functional effect on CAR-T cells. In vivo data in Figure 5 is particularly important as it shows a functional impact of CLDN18.2 EV in the presence of CLDN18.2 positive PDXs. Although the tumor expansion differences are small, they seem consistent in two different PDXs. I would rather see panels d and e merged (with controls and treatments in the same Figure) and then if the authors consider it necessary, zoom into the selected groups.

Moreover, the authors show decreases CAR-T cell numbers that would be consistent with CAR-T cell exhaustion.

- The in vivo data showing combination of Zolbetuximab with CLDN18.2 CAR-T is exciting. How do the authors explain that the antibody is not masking the antigen from CARs? Have the authors whether EV-CLDN18.2 could be also doing some CAR masking that could further contribute to decreased killing.

Minor:

- Panel in Figure 1a is clarifying to understand the scope of the study. Good job.

- In Figure 5B there are no error bars or replicates, please provide them.

- It is not possible to asses images from Fig3a and Fig4a with their current quality. Please provide clearer pictures.

- Regarding the RNAseq of CAR-T cells treated with CLDN18.2 exosomes. Instead of providing a heatmap in Figure 3b, please provide a barplots showing replicates and test for significance.

- I would move all trajectory analyses to Extended Data Figures.

- Please provide a clearer legend integrated in the Figure 5c-g, it is not immediately clear what the colors correspond to.

Version 1:

Reviewer comments:

Reviewer #2

(Remarks to the Author)
As mentioned in the first round of revisions, the manuscript contains a large amount of data with significant findings and several innovative approaches are used. The authors demonstrated an association between levels of CLDN18.2 EVs and

resistance to CAR-T therapy in patients. In addition, they show using plasma exchange that response to CAR-T and antibody can be restored in vivo and in patients. In contrast, the role of EVs in CAR-T cell dysfunction via EV antigen interaction with CAR is not novel. The role of EVs in blocking therapeutic antibodies has also been previously reported. The authors acknowledged the comments and made an effort to respond to all of them. However, it remains several shortcomings that need to be addressed before the study is suitable for publication.

Part 1: Identification of CLDN18.2/PD-1/PD-L2 EVs as circulating biomarkers of response to CLDN18.2 CAR-T therapy:
I thank the authors to provide additional experiments for the EV array, a critical method in the manuscript. Despite prior reports using the EV array and additional explanations from the authors, there is a lack of analytical validation and compelling evidence of the reproducible quantitative analysis of proposed markers. In the rebuttal letter, the authors claim that the linear dynamic range indicates sufficient sensitivity and accuracy. Without limit of detection, sensitivity is not measured. Accuracy can only be measured with true reference value. The study does not mention if positive and negative controls were used. Furthermore, no standard deviation is provided which suggests that no technical replicates have been used. It is also critical to assess the precision of the technique. No negative and positive controls are mentioned which is an important limitation.
The authors conducted an ELISA on isolated EVs to validate the data found with EV array. However, the agreement between both techniques is not acceptable suggesting a high variability for both analytical methods. Detailed methodology for the ELISA is required. Importantly, standard curve, limit of detection and limit of quantification are not provided for both ELISA and EV array. It is unclear if technical replicates were used. If yes, raw data of replicates should be provided. Without this information, it is difficult to conclude that the quantitative analysis of EV proteins is reliable and reproducible. Latter in the rebuttal, the authors claim that this work was not designed to methodologically compare EV array with ELISA. Therefore, it is unclear why the authors present ELISA data? The data shown are not supporting the performance of the EV-array.
In their revisions, the authors add they use SEC to purify EVs from plasma but no purity evaluation is provided and important methodological details are missing.The SEC purification kit provided by EVbio does not seem to be commercialized worldwide. Methodological details on the SEC purification kit, fractions collected with EV and protein concentrations measurements should be provided. Assessment of EV purity is also required per MISEV 2023 guidelines.

I appreciate the authors' response on their hypothesis they can detect intracellular proteins on surface of EVs as part of the "corona". They cite two landmark studies on the existence of protein corona in EVs and present TEM results from a previous study. However, it remains uncertain that the authors can quantify with accuracy expression of intracellular proteins on surface of EVs. Additional experiments are needed to support their hypothesis.

The authors looked at the association of EV proteins with response to CLDN18.2 CAR-T therapy using a discovery cohort (N=31 patients with baseline samples) and validation cohort (N=46 patients with baseline samples). Based on the figure 1, the discovery cohort includes 77 patients. In Extended Figure 3, the discovery cohort has 31 patients. In Extended Figure 4, the validation cohort has 46 patients. It seems that the authors combine both cohorts in Fig.1. This discrepancy needs to be addressed by the authors.
In the table S7, EV-PD1 does not show strong association with outcome (p=0.453 for PFS and p=0.524 for OS). Opposite effects are observed between discovery and validation cohorts. Surprisingly, high EV-PD1 expression is associated with shorter PFS and OS in both the discovery and validation cohorts (Ext Fig.3 and 4) but COX analysis does not show association of EV-PD1 with outcome in the validation cohort. Based on this, it is unclear how the EV-PD1 contributes to the CPP-score and how CPP-score shows similar association with outcome for both discovery and validation cohorts.

Part 2: CLDN18.2 EVs interacts with CAR and induces CAR-T dysfunction:
PKH26 is recognized to form aggregates in solution and false positives in EV uptake experiments. To address my initial comments, the authors use SEC to purify PKH26-labeled EVs post-SEC. They provide revised figures but they miss quantification of PKH26-positive cells and the distance indicated by the scale bar are missing. In the revised Ext Fig 7f, no PKH26 signal was found in PBS control which is odd considering PKH26 form aggregates in PBS. Based on authors' comments, Fig.3a, 4a have been revised using purified samples with SEC but they are the same picture as the original submission.
In Fig.3a, there is much more PKH26-positive structures of similar size to CAR-T cells are present outside CAR-T cells. PKH26-positive EVs do not show discrete signals in CAR-T cells but the entire cytosol is labeled suggesting indirect PKH26 staining of CAR-T cells. In addition, it looks like 100% of cells are positive for PKH26 which is unusual as prior reports show that the uptake of EVs by T cells is very low.

Part3: CLDN18.2 EVs interacts with CAFs promoting the release of IL-6 blocking CAR-T efficacy:
The raw data provided for the proximity labeling do not match with the results presented. 17 proteins are exclusively found in CLDN18.2-TurboID-TM and not in control conditions. Then, 17 proteins have at least 2-fold difference in in CLDN18.2-TurboID-TM compared to control. Some of these 17 proteins are presented in the heatmap above. S100A4 is ranked 12/17. Similarly, 4 proteins are found exclusively in the control conditions and 30 proteins are upregulated by at least 2 fold in the control condition compared to CLDN18.2-TurboID-TM. Some proteins included in the heatmap are detected in only 1 replicate. The authors should carefully analyzed the data given the low number of replicates and further explain how they established their list of top 10 and bottom 10 proteins. Several proteins detected are associated to T/NK activity (e.g Gzm).

The authors proposed revisions for Figure 7d, 8c with additional replicates. However, it is unclear how they were able to generate novel replicates from the same PDX mouse model and the same donor-derived CAR-T.

Reviewer #3

(Remarks to the Author)
The authors have done an exceptional work adressing all my comments, I sincerely congratulate them for their interesting work.

Reviewer #4

(Remarks to the Author)

Version 2:

Reviewer comments:

Reviewer #2

(Remarks to the Author)
Thank you for the revised manuscript and rebuttal. I appreciate the additional methodological detail and the steps taken to strengthen the EV workflow (e.g., SEC kit description, NTA/TEM, and proteomics summaries). These additions are helpful. However, several concerns raised in my prior review remain insufficiently addressed in the rebuttal, particularly given the central focus on EV functional effects on CAR-T efficacy, where EV purity and integrity are paramount for data quality and reproducibility:
EV isolation/purity: improved description, but MISEV-aligned purity evidence remains incomplete. While SEC + NTA/TEM + MS summaries are a step forward, NTA/TEM alone cannot establish purity, and the current MS “marker enrichment” presentation does not allow readers to assess co-isolated plasma protein contamination. For plasma EV functional claims, a minimal MISEV-aligned positive/negative marker panel is still expected on the actual EV preparations used in key downstream assays (EV-array and CAR-T functional assays), not only a limited subset. Please add a concise, MISEV-aligned purity panel (multiple positive EV markers plus at least one negative marker) for the relevant EV preparations used across the key experiments.
Conventional purification methods and potential co-isolated soluble components. Because widely used conventional isolation approaches can co-isolate soluble plasma components, the rebuttal does not yet adequately address how such contaminants were excluded/controlled for. This is important because co-isolated factors could influence EV-array readouts and functional assay outcomes, complicating attribution to EVs per se.
Replicates and statistical reporting for CAR-T/EV functional assays. In some figures, replicates and/or statistical details appear incomplete or not clearly reported in a way that supports reproducibility. Please ensure that replicate numbers, what constitutes biological vs technical replicates, and the statistical approach are explicitly stated for each relevant experiment.
Addressing these points directly in the manuscript (and supplement where appropriate) would substantially improve transparency and confidence that the reported functional effects are truly EV-dependent and reproducible.

Reviewer #4

(Remarks to the Author)
The authors have responded to most of my comments. I respectfully disagree with some of their assumptions, for example just because NTA is used widely and as such one may list a vast amount of literature, however these publications do not show that the method is both accurate or has no limitations. The authors should consider this. It would behoove the authors that they inform the reader on limitations of their assay for diagnostic purposes. Indeed as the auhtors do not see a need for technical validation with spike-inns, their CPP score is research use only and has not been rigorously validated as potential diagnostic test that for use in the clinic. They should make the reader aware of this important difference and not inflate their results. The functional experiments do however support the diagnostic potential of the EVs, and it should be communicated as such. In addition figure 1d remains confusing where EV-array and EV-elisa are compared, have the auhtors performed an outlier test, i could not find this.

Version 3:

Reviewer comments:

Reviewer #2

(Remarks to the Author)

Reviewer #4

(Remarks to the Author)
I have read the comments and rebuttal provided by the authors. They have anwsered all my questions and I commend them on their efforts revising the manuscript.

Open Access This Peer Review File is licensed under a Creative Commons Attribution 4.0 International License, which permits use, sharing, adaptation, distribution and reproduction in any medium or format, as long as you give appropriate credit to the original author(s) and the source, provide a link to the Creative Commons license, and indicate if changes were made.
In cases where reviewers are anonymous, credit should be given to 'Anonymous Referee' and the source.
The images or other third party material in this Peer Review File are included in the article's Creative Commons license, unless indicated otherwise in a credit line to the material. If material is not included in the article's Creative Commons license and your intended use is not permitted by statutory regulation or exceeds the permitted use, you will need to obtain permission directly from the copyright holder.
To view a copy of this license, visit https://creativecommons.org/licenses/by/4.0/

REVIEWER COMMENTS

Reviewer #1 (Remarks to the Author):

This manuscript investigates the role of extracellular vesicle in mediating the efficacy of CLDN18.2-targeted CAR-T cell and antibody therapies against gastrointestinal cancers. The study is ambitious in scope and employs sophisticated multi-omics techniques.

Strengths:

• Novelty: The identification of EV-derived proteins as biomarkers predictive of treatment response is a significant contribution to the field. The CPP-score, combining the expression of several key proteins, offers a robust and potentially clinically useful predictive biomarker.

• Comprehensive Methodology: The study uses a robust and comprehensive methodology including multi-omics analyses, functional assays, and in vivo experiments. The use of both CAR-T and antibody therapy arms strengthens the findings.

• Mechanistic Insights: The study makes attempts to elucidate the mechanisms by which EV-proteins interfere with therapy efficacy, implicating CAF activation and T cell exhaustion. This is a valuable step towards therapeutic intervention.

• Clinical Relevance: The authors directly translate their preclinical findings into clinical implications by exploring plasma exchange and intraperitoneal infusion strategies for therapeutic sensitization.

(Abstract):

• "but biomarkers for long-term benefit and therapeutic sensitization strategies are needed" —> "but biomarkers predictive of long-term benefit and therapeutic sensitization strategies are needed."

A. Thank you for your comment. We have revised the manuscript accordingly to address your suggestions.

• "We depicted EV-protein expression profiles" —> "We determined EV-protein expression profiles" or "We characterized EV-protein expression profiles".

A. Thank you for your comment. We have revised the manuscript accordingly to address your suggestions.

• "then performed multi-omics, functional and mechanistic investigations" —> "and then performed multi-omics, functional, and mechanistic investigations". (Improved flow)

A. Thank you for your comment. We have revised the manuscript accordingly to address your suggestions.

(Introduction):

• "In healthy gastric tissues, CLDN18.2 expression is limited to differentiated epithelial cells in the mucosa." —> "In healthy gastric tissue, CLDN18.2 expression is restricted to differentiated epithelial cells of the mucosa." (More precise)

A. Thank you for your comment. We have revised the manuscript accordingly to address your suggestions.

• "Due to its prevalence and tumor-specific presence," —> "Due to its tumor-specific expression and prevalence," (Improved flow)

A. Thank you for your comment. We have revised the manuscript accordingly to address your suggestions.

• "efficacy of CAR-T reported in solid tumors" —> "efficacy reported for CAR-T therapy in solid tumors". (Clarified)

A. Thank you for your comment. We have revised the manuscript accordingly to address your suggestions.

• "However, despite these advances, there is an urgent need for proper biomarkers instructing & monitoring CLDN18.2-targeted therapies' efficacy," —> "However, despite these advances, there is an urgent need for biomarkers to predict and monitor the efficacy of CLDN18.2-targeted therapies," (More concise and grammatically correct)

A. Thank you for your comment. We have revised the manuscript accordingly to address your suggestions.

Page 6 (Introduction):

• "we uncovered the roles and mechanisms of key EV-proteins in modulating TIME through influencing CLDN18.2-CAR-T cells, anti-CLDN18.2-antibody, cancer associated fibroblasts (CAFs) and T helper cell balance, thus interfering with the efficacy of CLDN18.2-targeted-CAR-T/antibody therapies." —> "We elucidated the roles and mechanisms of key EV proteins in modulating the tumor immune microenvironment (TIME) by influencing CLDN18.2-CAR-T cells, anti-CLDN18.2 antibodies, cancer-associated fibroblasts (CAFs), and T helper cell balance, thus interfering with the efficacy of CLDN18.2-targeted CAR-T and antibody therapies." (Improved flow and clarity. Rephrased for conciseness and accuracy)

A. Thank you for your comment. We have revised the manuscript accordingly to address your suggestions.

A thorough grammatical and typographical review of the entire manuscript is recommended before re-submission. This is a potentially impactful study that offers a novel approach to improve the efficacy of CLDN18.2-targeted therapies.

A. We truly appreciate your kind assistance with language revisions and your encouraging comments on our work. We have improved the formatting and language throughout the revised manuscript.

Reviewer #2 (Remarks to the Author):

In their study, Qi et al's investigated the role of CLDN18.2-derived EVs as 1) a blood biomarker of resistance to CAR-T therapy and CLDN18.2-targeting antibodies and 2) a mediator of CAR-T dysfunction by inducing CAR-T exhaustion through binding of EV-bound CLDN18.2 to the T-cell derived CAR and by promoting the release of IL-6 by CAFs.

The manuscript contains an impressive amount of results and discovery+validation patient cohorts. With 8 main figures, 9 extended figures, 10 Supplementary figures and 8 supplementary tables, this paper is difficult to read and at each sentence, we have to navigate through the paper to find the right figure panel. Separating the story in two high-impact papers should be considered. Most figures have 10+ panels which make them difficult to interpret. Some panels are very small and the statistical significance not always readable. Repetitive key panels could be kept in main figures and the remaining in Supplementary Data.

A. Thank you for the comment. This manuscript presents a translational research for CLDN18.2-targeted therapies, offering a comprehensive perspective from therapeutic biomarkers, functions & mechanisms to management strategies. Therefore, it contains an extensive amount of data and findings. We deeply appreciate your suggestion for the formatting of this manuscript. We have also considered to separating the story into two papers, however, given that the various components of this study are closely interconnected and mutually reinforcing, removing any section may disrupt the coherence and completeness of the overall body of evidence. Therefore, in order to maintain the integrity of the study, we have decided not to divide the manuscript into two separate papers.

We have improved the formatting and language throughout the revised manuscript, and have included additional statistical analyses and replicated key panels wherever possible.

The significance of the results presented is high because it "solves" several concepts in the EV biology that are still uncleared.

The authors could acknowledge and discuss their data with a recent study from the Guo's group showing the biological and clinical role of MSLN+-PD-L1+EVs in promoting resistance to MSLN CAR-T (Zhong W et al, Cancer Research, 2023). The study includes several innovative methods and all the results seem to align well the hypothesis. It is well recognized that the study of EVs not only as blood biomarker and as promoter of therapeutic resistance is challenging due to the difficulty of quantifying EV subsets with high reproducibility and isolating them with high purity. Given the quality of the results obtained in their study, the authors are demanded to provide more details on the procedures and methods used in their study. In addition, some key experiments require technical validation and appropriate controls.

A. Thank you for the suggestion. This recent study (Zhong W et al., *Cancer Res*. 2023 Aug 15;83(16):2790-2806) pointed out that solid tumors release EVs that carry both mesothelin and PD-L1. These EVs preferentially interacted with cognate CAR-T cells and inhibited their proliferation, migration, and function, while inhibiting EV secretion increased CAR-T's infiltration and anti-tumor activity. These reports are consistent with our observations in the context of CLDN18.2-targeted therapies. However, the study used chemical inhibitors to block exosome secretion, which presents limited clinical relevance and translational potential. Moreover, the possibility of off-target effects associated with the inhibitors cannot be excluded.

In contrast, our study provided more in-depth clinical/mechanistic/translational investigations, and further demonstrating that EV-CLDN18.2 interferes with both CLDN18.2-targeted CAR-T and antibody therapies. Additionally, our study employed plasma exchange (PE) and intraperitoneal infusion (IPI) approaches to depleted harmful EV-proteins in plasma, both PE and IPI offer direct clinical applicability. Therefore, we believe our work offers a more thorough understanding and holds stronger translational relevance.

We deeply appreciate your suggestions to improve the quality of our manuscript. We have discussed the *Cancer Research* paper in the revised manuscript, and have provided more details on the procedures and methods used in our study.

My comments will be divided in three major studies conducted in this manuscript:

Part 1: Identification of CLDN18.2/PD-1/PD-L2 EVs as circulating biomarkers of response to CLDN18.2 CAR-T therapy

Elevated plasma concentrations of CLDN18.2-EVs are associated with shorter clinical outcome in response to CAR-T which validate a prior report with MSLN-EVs and MSLN-CAR-T therapy (Zhong et al, in Cancer Research 2023). To that end, the authors employ a EV microarray similar to what has been originally described by Jorgensen M et al, JEV, 2013. The authors should cite the original paper to support feasibility of the EV array. Most clinical findings in this paper origin from the EV array data. Given the relevance and performance of this technique presented herein, the authors should address the concerns below and provide raw EV array data for all measurements in Supplementary files.

1) The protocol to isolate EVs from conditioned media and plasma is odd. The authors state they centrifuge at 15,000 x g to pellet EVs. At such speed, only low abundant large EVs > 200 nm are likely to pellet and a higher centrifugation speed is needed to isolate smaller EVs. Centrifugation alone has been shown to have low EV purity due to the co-isolation of aggregates, soluble factors and lipoproteins. Use of size-based methods combined with centrifugation has been shown to improve EV purity. The authors should address this as it is unclear whether EV array measured EVs only or EVs and soluble proteins.

There are several methodological details missing in the plasma EV isolation protocol. The authors should use the MIBlood-EV tool to report their technical procedures (Lucien F et al, JEV, 2023).

A. Thank you for the comment. We apologize for the ambiguous descriptions of protocol in the original submission. We have cited the original paper (Jørgensen et al, *J Extracell Vesicles*. 2013 Jun 18:2), and provided raw EV-array data (**Supplementary Table S5**) in the revised manuscript.

In this manuscript, both ultracentrifugation and size-exclusion chromatography (SEC) methods were applied to isolate EVs. To perform functional & mechanistic experiments, it is essential to obtain EVs with maximal preservation of biological activity. Therefore, we used ultracentrifugation to isolate EVs from human-CLDN18.2-overexpressing HEK293F cell subline, a human-PD1-overexpressing HEK293F cell subline and a mouse-PD-L2-overexpressing CT26 cell subline. The ultracentrifugation should be at 100,000g, we apologize for the typo of 15,000g. The purity & quality of isolated EV-CLDN18.2/EV-PD1/EV-PD-L2 have been verified with TEM, NTA, and western blot assay, as shown in Fig. 7a-7c/7f-7h and Extended Data Fig. 7a-7d.

On the other hand, to quality-control the accuracy of biomarker analysis, we used a SEC-based purification kit (EV40T, EVbio, China) to isolate EVs from patient plasma, then performed ELISA for isolated EVs to compare the quantification of EV-proteins with EV-array (**revised Fig. 1d, revised Extended Data Fig. 2k**). We also re-purified PKH26 pre-labeled EVs using a SEC-based purification kit (ES9016, Echobiotech) to eliminate unincorporated dye, then proceeded EVs to fluorescent staining (**revised Extended Data Fig. 7f**, **revised Fig. 3a, revised Fig. 4a**). We have improved the descriptions for methodology in the revised manuscript.

As we have described in **Methods**, EV-array reflects EV protein expression normalized by EV markers, without the need for EV isolation. In this assay, selected panel proteins are illuminated with biotinylated antibodies of EV markers (CD81, CD9, CD63, ALIX), and then normalized with EV-markers' expressions during quantification (each EV-protein's absolute expression was normalized into relative expression by dividing the geometric mean of CD81 and CD9). The rationale ensures that only proteins encapsuled by vesicles expressing EV-markers, rather than soluble proteins, can be quantified by this assay. The specificity of EV-array has been verified by several studies (Jørgensen et al, *J Extracell Vesicles*. 2013 Jun 18:2; Zhang et al., *J Extracell Vesicles*. 2022 Apr;11(4):e12209; Dai et al., *J Extracell Vesicles*. 2025 Apr;14(4):e70054). We also applied quality-control procedures including TEM, NTA, western blot and immunogold labeling assay to ensure that proteins screened by EV-array are expressed on plasma EVs (**revised Extended Data Fig. 1**). In the revised manuscript, we have also improved related descriptions to these results.

2) For the EV array, the authors claim they use 10 µL unpurified plasma sample diluted (1:10), in other words 1 microliter of plasma. It seems a very low volume to have enough sensitivity for detecting double-positive EVs (target + CD81/CD9/CD63/ALIX). In their original study, Jorgensen et al, showed that 1 microliters of plasma can be enough to detect signal over noise but there is a lot variability between patients and some may be below the LoD and LoQ. The assays conducted do not seem to include positive and negative controls.

A. Thank you for the comment, we apologize for the ambiguous descriptions. Our EV-array used 10 µL unpurified plasma sample diluted with 90 µL wash buffer (0.05% Tween20 in PBS) into 100µL volume, which provide a sufficient accuracy. We have improved the description in the revised manuscript.

Importantly, LoD for markers tested is not provided. While it could be difficult to demonstrate the linear range of detection and quantification of all markers, it should be presented for the key markers identified such as CLDN18.2, PD-L1 and PD-1.

A. Thank you for the question. To determine the limit of detection (LOD) of the EV-array, we performed serial dilution experiments using pooled plasma from ten individuals. Plasma volumes of 5 µL, 8 µL, 11 µL, 14 µL, 17 µL, and 20 µL were each diluted to a final volume of 100 µL with wash buffer (0.05% Tween-20 in PBS), followed by EV-array analysis targeting EV markers CD81/CD9/CD63/ALIX and EV-CLDN18.2/PD-1/PD-L2. A linear relationship between fluorescence intensity and plasma volume was observed up to 20 µL, beyond which signal saturation occurred (**Fig for review only. 1**). Therefore, 5 to 20 µL fall within the linear dynamic range for EV-protein quantification, indicating sufficient sensitivity and accuracy of the EV-array to evaluated 10 µL plasma samples. As EV-array is a well-

established method, we did not include these data in the revised manuscript considering the limitations of text and figure.

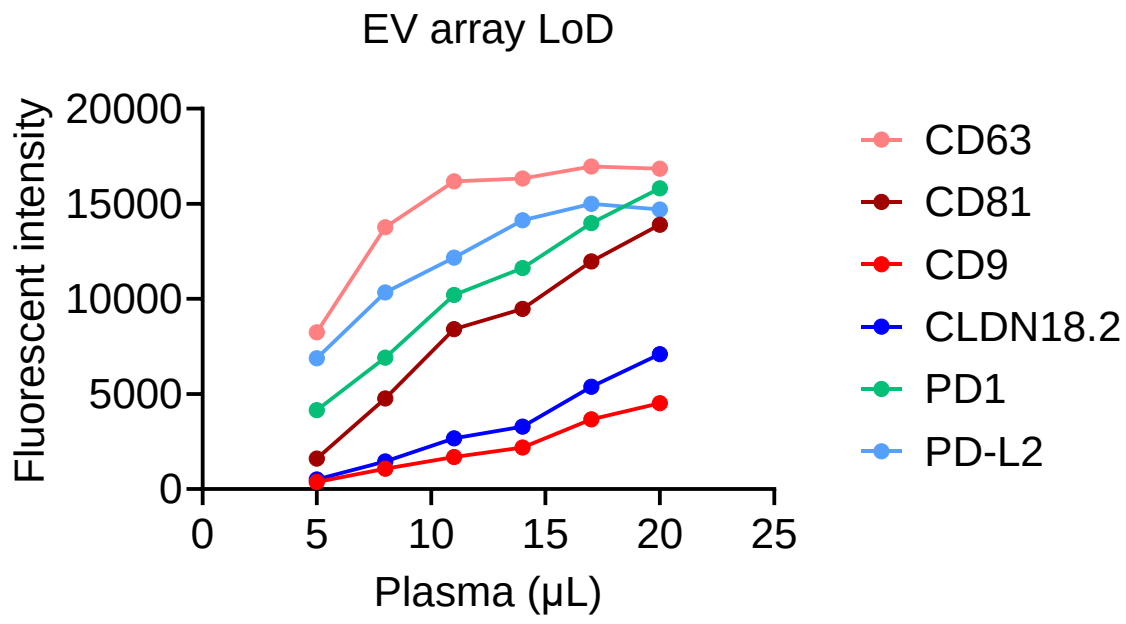

Fig for review only. 1

Interestingly, the panel of markers used include non-surface bound proteins and intravesicular proteins such as FOXP3, STING or ARG1. It is unclear how the authors have been able to capture EVs using intravesicular targets.

A. Thank you for the comment. As reported by two typical studies, intravesicular proteins may be presented on the surface of extracellular vesicles (EVs) as part of the protein corona, a crucial mediator of EV functions (Martin Wolf et al., A functional corona around extracellular vesicles enhances angiogenesis, skin regeneration and immunomodulation. *J Extracell Vesicles*. 2022 Apr;11(4):e12207; Buzas EI., Opportunities and challenges in studying the extracellular vesicle corona. *Nat Cell Biol*. 2022;24(9):1322-1325). As we have mentioned in Methods, EV-array works through: 1. Capturing EVs through a 3D modified slide pre-printed with antibodies for specific target proteins; 2. Combining captured EVs with biotinylated antibodies for EV markers; 3. Quantifying the fluorescent signal intensity for EV markers. Therefore, EV-array reflects the expression of proteins normalized by EV markers, provided that the protein is expressed on the captured EVs. As a result, in additional to membrane-bound proteins, we believe EV-array can also detecting intracellular proteins when they are presented as part of the EV's protein corona.

On the other hand, one of our previous studies also adopted EV-array to quantify the expression of a panel of key TIME molecules in patient plasma EVs, including both membrane-bound and intracellular proteins (Zhang et al., Plasma extracellular vesicle derived protein profile predicting and monitoring immunotherapeutic outcomes of gastric cancer. *J Extracell Vesicles*. 2022 Apr;11(4):e12209). In that study, we assessed mixed plasma EV specimens through immunogold labelling assay to confirm that ARG1 could be specifically detected on EVs, the same as other membrane-bound EV-proteins (**Cited Supplementary Fig. S4E**). We also identified EV-derived ARG1 as a therapeutic biomarker with EV-array, and combined EV-ARG1 with another three plasma EV-derived proteins to generate an EV-score that robustly predicting immunotherapeutic outcomes. These data proved that both surface-bound and intravesicular markers can be detected by EV-array.

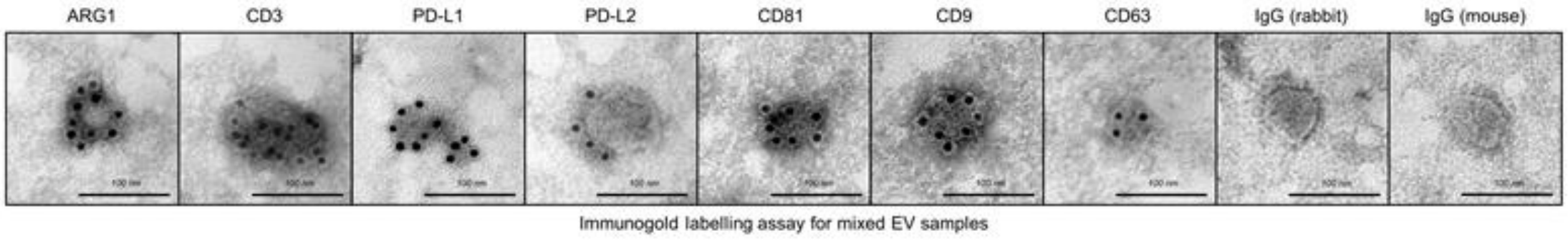

Cited Supplementary Fig. S4E from our previously published paper (*J Extracell Vesicles*. 2022 Apr;11(4):e12209).

The authors should also provide how they determine low and high cutoffs for KM curves.

A. Thank you for the comment. As we have mentioned in **Results** and the "Data processing of EV expression array" section of **Methods**, we categorized the expression of all panel EV proteins in discovery, validating and antibody cohorts as "high" or "low" by comparing with the median of a 71-case reference cohort. In each cohort, EV-proteins expressed higher than the reference cohort's median were defined as "high", while EV-proteins expressed lower than the reference cohort's median were defined as "low". The same standard was adopted to determine low and high cutoffs for KM curves. We have improved the descriptions in the revised manuscript.

In Fig1d, Extended Figure 2k, the authors claim that the accuracy of the EV array is proven due to the correlation between EV array and ELISA for CLDN18.2-EV quantification. A correlation coefficient of 0.66 and 0.70 is not impressive and does not convince on the accuracy of the EV array vs ELISA. Visually, the coefficient of determination would be very low, thereby not supporting the authors' claim. No detailed methodology for the ELISA is provided.

A. Thank you for the comment, here we provide additional data to address your concern. Among all the plasma samples analyzed by EV-array in this study, we were able to retrieve 31 patients' baseline samples for further assessment. We re-quantified EV-CLDN18.2/EV-PD1 expression with ELISA for EVs isolated from these 31 plasma samples, then compared the expressional correlations for ELISA and EV-array. Correlation coefficients of 0.5735 for EV-CLDN18.2 (**revised Fig. 1d**) and 0.5881 for EV-PD1 (**revised Extended Data Fig. 2k**) were observed in 31 patients, which is comparable to our previous data in 10 patients. We have updated the relevant figures in the revised manuscript.

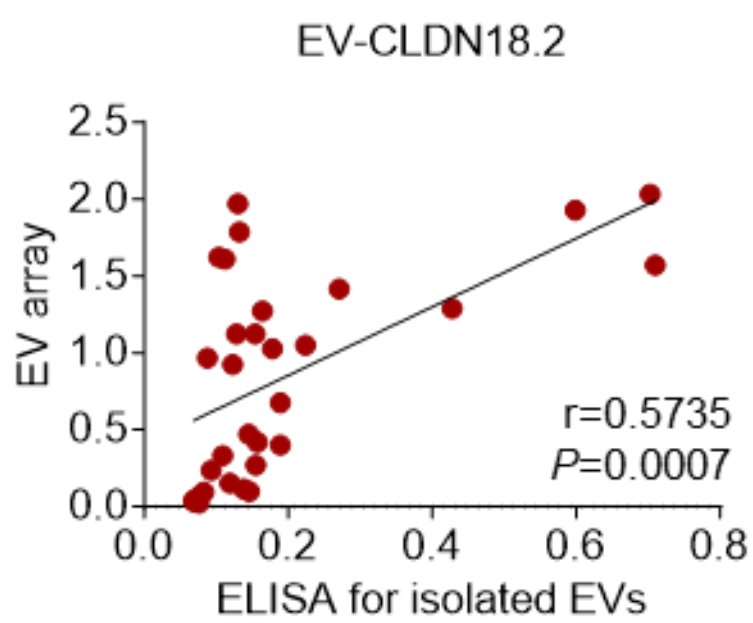

Revised Fig. 1d

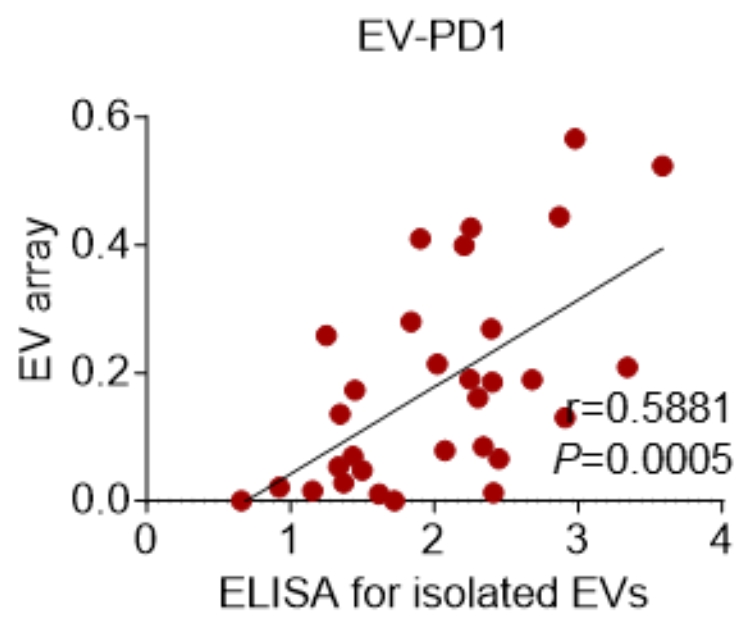

Revised Extended Data Fig. 2k

However, EV-array is not a mimic of ELISA. Although the correlation coefficient is not so impressive, it does not overshadow the accuracy of our quantification with EV-array. For quantifying the expression of EV proteins, ELISA works through: 1. Coating the slide with isolated EVs; 2. Directly assessing EV-proteins with primary/secondary antibodies. In this study, we used ELISA as a benchmark for quality control, rather than conclusive results. As a mature method, EV-array was first reported by Jørgensen et al (*J Extracell Vesicles*. 2013 Jun 18:2), whose accuracy in quantifying EV proteins has hereafter been proved by several studies (Zhang et al., *J Extracell Vesicles*. 2022 Apr;11(4):e12209; Dai et al., *J Extracell Vesicles*. 2025 Apr;14(4):e70054). EV-array works through: 1. Capturing EVs through a 3D modified slide pre-printed with antibodies for specific target proteins; 2. Combining captured EVs with biotinylated antibodies for EV markers (CD81/CD9/CD63/ALIX); 3. Quantifying the fluorescent signal intensity for EV markers. Therefore, EV-array reflects EV protein expression normalized by EV markers, without the need for EV isolation. Since ELISA and EV-array possess different rationales for quantifying EV proteins, it is reasonable that their results shown similarities while maintaining differences.

This work is not designed to methodologically compare EV-array with ELISA. Since all the plasma samples from CAR-T/antibody cohorts were quantified using EV-array, we believe the generated biomarker data for EV proteins are accurate and stable within the context of this single method. Additionally, as described in **Methods**, in order to generate conclusive results, we defined EV-protein expression generated by EV-array as “high” or “low” based on the reference cohort’s medians. This step converted the continuous variables from EV-array into categorical variables, which also helps minimizes potential disparities between EV-array and ELISA.

We appreciate your suggestions and apologize for not having provided detailed methodology. Please find more detailed descriptions in the revised manuscript.

3) Some figures lack statistical analysis. In Figure 1f and 1g, p value and statistical approach is not provided which precludes the identification of potential biomarker of response to CAR-T.

A. Thank you for the suggestion, we apologize for the lack of descriptions. As we have mentioned in the original **Results**, high-expression rates in nonresponders/responders were calculated into fold changes (FC) to screen all EV-proteins, with FC≥1.3/FC≤0.7 considered as cutoffs for responsive/nonresponsive EV-proteins. Since Fig. 1f and Supplementary Fig. 5c calculates fold changes of high-expression rates between nonresponders and responders, no *P*-value is involved.

For the screening with Kaplan-Meier analysis (Log-rank test), HR≥1.3/HR≤0.7 were considered as

favorable/adverse-prognostic EV-proteins. We have previously demonstrated HR from Log-rank test for all EV-proteins in Fig. 1g and Supplementary Fig. 5d, here we measure *P*-value from Log-rank test for all EV-proteins as you suggested. As shown below, EV-CLDN18.2/EV-PD1/EV-PD-L2 displayed significant *P*-values, which confirms they are important prognostic markers for CLDN18.2-CAR-T therapy. These modifications have been incorporated into **revised Fig. 1g** and **revised Supplementary Fig. 5d**.

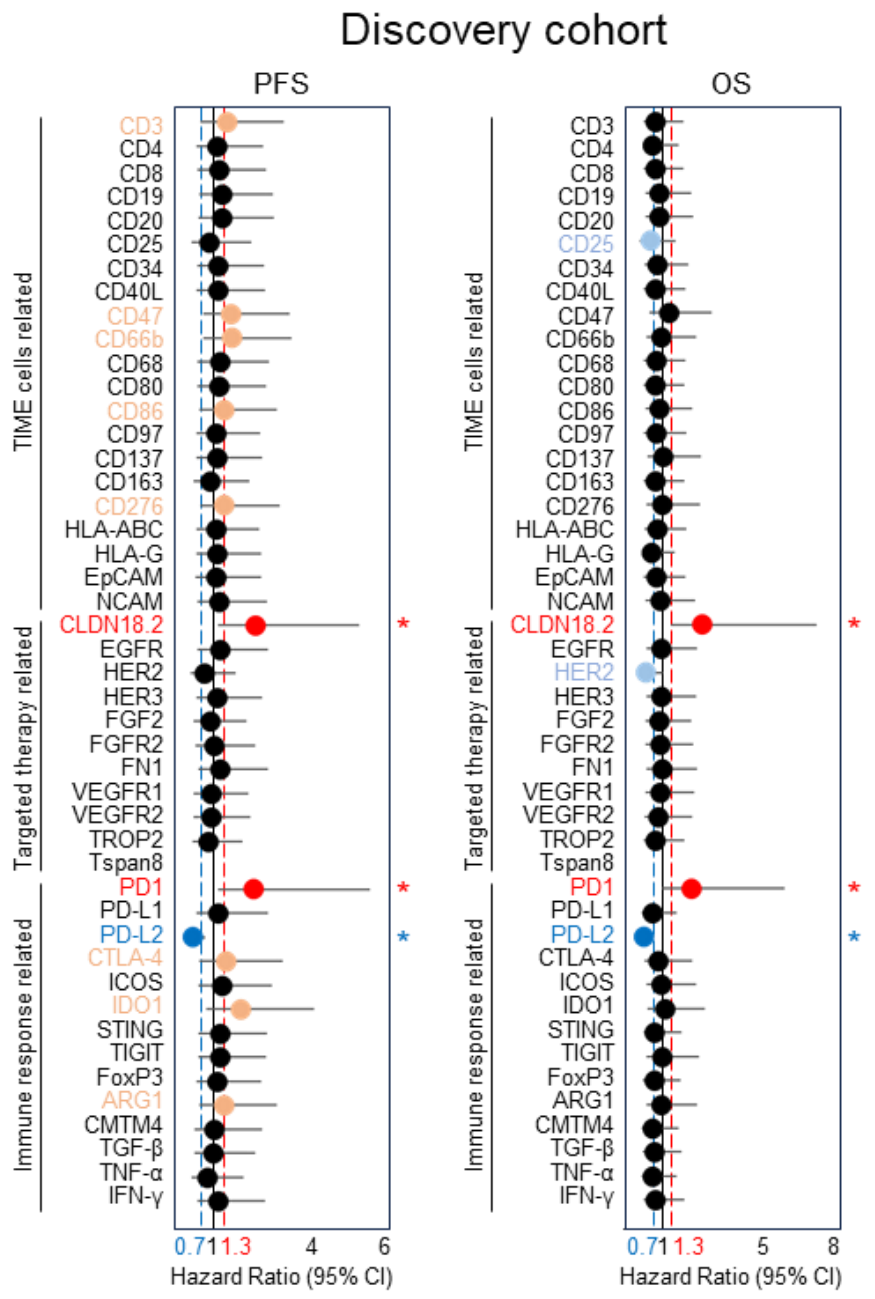

Revised Fig. 1g

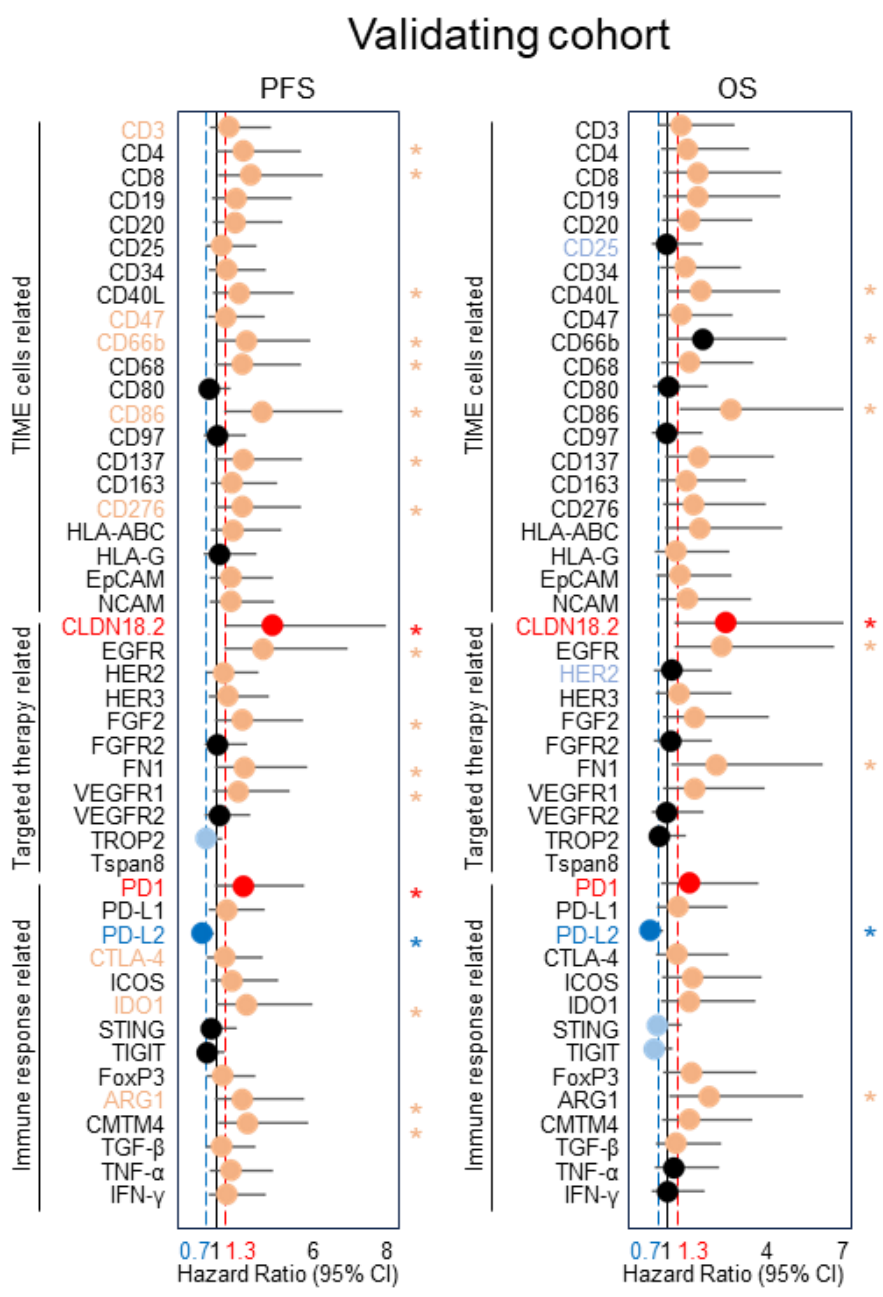

Revised Supplementary Fig. 5d

4) Gating strategy for flow cytometry analysis should be provided. No methodological details on PBMC isolation, staining and flow cytometry acquisition and data analysis are provided. Antibody concentrations and fluorophores used should be provided.

A. Thank you for the suggestion. Peripheral blood mononuclear cells (PBMCs) were isolated from fresh peripheral blood samples using Ficoll density gradient centrifugation. For flow cytometry, fresh cells were collected and washed with DPBS (Dulbecco's Phosphate-Buffered Saline), then resuspend with DPBS into 1×10^6 cells/mL. Ensuring a viability greater than 95%, cells were identified by CD3-FITC staining (317305, Biolegend), followed by surface staining of PD-1-PE (367404, Biolegend), TIM-3-APC (345012, Biolegend), LAG-3-PE/Cy7 (369310, Biolegend), and TIGIT-BV605 (372710, Biolegend) for the $CD3^+$ population. Antibodies were used at a concentration of 5 μL per 100 μL, with 1×10^5 cells per sample. Cells were stained at room temperature in dark, administrated with DPBS wash and filtering, and proceeded for flow cytometry detection (CytoFlex, Beckman).

The gating strategy was as follows: First gate (FSC/SSC): Forward and side scatter were adjusted to ensure that all cell populations of interest were within the view. A gate was drawn to include all viable cells. Second gate ($CD3^+$ cells): Based on the negative control (unstained T cells), the $CD3^+$ population was gated. Third gate (positive populations for checkpoint markers): Based on the $CD3^+$ gate, voltages were adjusted to resolve positive and negative populations for each marker. Populations with fluorescence intensity greater than 10^4 were considered positive. Flow cytometry results were analyzed using the CytoExpert software.

We apologize for not having provided detailed methodology. Please find more detailed descriptions in the revised manuscript.

Part 2: CLDN18.2 EVs interacts with CAR and induces CAR-T dysfunction:

1) At the conclusion of their first part (Figure 8), the authors state: "CPP-score remained stable throughout CLDN18.2-CAR-T treatment (Extended Data Fig. 3j), while dynamic CAR-T cell copies (Supplementary Table S5) were lower for patients with high baseline-EV-CLDN18.2/EV-PD1/CPP-score and higher for patients with high baseline-EV-PD-L2 (Extended Data Fig. 3k), indicating EV-CLDN18.2/EV-PD1 reduce CAR-T cell content, while EV-PD-L2 maintains it." With the correlative data presented, the association of EV dynamics with response to CAR-T cells does not mean that EVs are directly impacting on CAR-T efficacy. The authors should rephrase their conclusion.

A. Thank you for the suggestion. We have rephrased the text to "On the other hand, CPP-score remained stable throughout CLDN18.2-CAR-T treatment (Extended Data Fig. 3j), while dynamic CAR-T cell copies (Supplementary Table S6) were lower for patients with high baseline-EV-CLDN18.2/EV-PD1/CPP-score and higher for patients with high baseline-EV-PD-L2 (Extended Data Fig. 3k). These data indicated that EV-CLDN18.2/EV-PD1 were related with reduced, while EV-PD-L2 was related with increased CAR-T cell content" in the revised manuscript.

Did the authors look at the association of radiographic tumor burden with plasma CLDN18.2 EV concentration and response to CAR-T? It is possible that poor PFS/OS is found in patients high baseline tumor burden. Does the multivariate analysis of CLDN18.2 and outcome included radiographic tumor

burden?

A. Thank you for the question. In clinical practice of cancer treatment, instead of radiographic tumor burden, objective response rate (ORR)/disease control rate (DCR)/responses (responders vs nonresponders) and PFS/OS, are adopted to describe the efficacy of anti-cancer therapies. Among these parameters, ORR, DCR and response are calculated by referring to changes of radiographic tumor burden during treatment. In the original submission, we have provided definitions to these parameters (Materials & Methods section), and have already pointed out that EV-CLDN18.2 possesses higher proportions of high-expression rate in nonresponders than in responders for both discovery (Fig. 1f) and validating (Supplementary Fig. 5c) cohorts of CLDN18.2-CAR-T therapy.

Here we provide additional analysis to address your concern. As demonstrated in discovery+validating cohorts, although patients defined as high baseline EV-CLDN18.2 showed slightly higher radiographic tumor burden, there is no linear correlation between radiographic tumor burden and plasma EV-CLDN18.2 (**Fig for review only 2. a-b**). Also, baseline radiographic tumor burden is unrelated with response/ORR/DCR (**Fig for review only 2. c**) and median-stratified PFS/OS (**Fig for review only 2. d**) of CLDN18.2-CAR-T therapy. Considering radiographic tumor burden is not an appropriate parameter to describe therapeutic efficacy, we did not display these data in the revised manuscript.

On the other hand, for multivariate analysis, we have already proved in original submission that the CPP-score (in which EV-CLDN18.2 is a major component) outperforms a logistic regression model (CPP-logistics) for positive control in predicting CLDN18.2-CAR-T's response, PFS and OS (Supplementary Fig. 6a-6d). Here we have performed additional multivariate analysis, which proved that CPP-score also outperforms CPP-logistics in characterizing CLDN18.2-CAR-T's ORR and DCR (**Fig for review only 2. e**). We have incorporated the multivariate analysis data for ORR and DCR into a new **Supplementary Fig. 6d** in the revised manuscript.

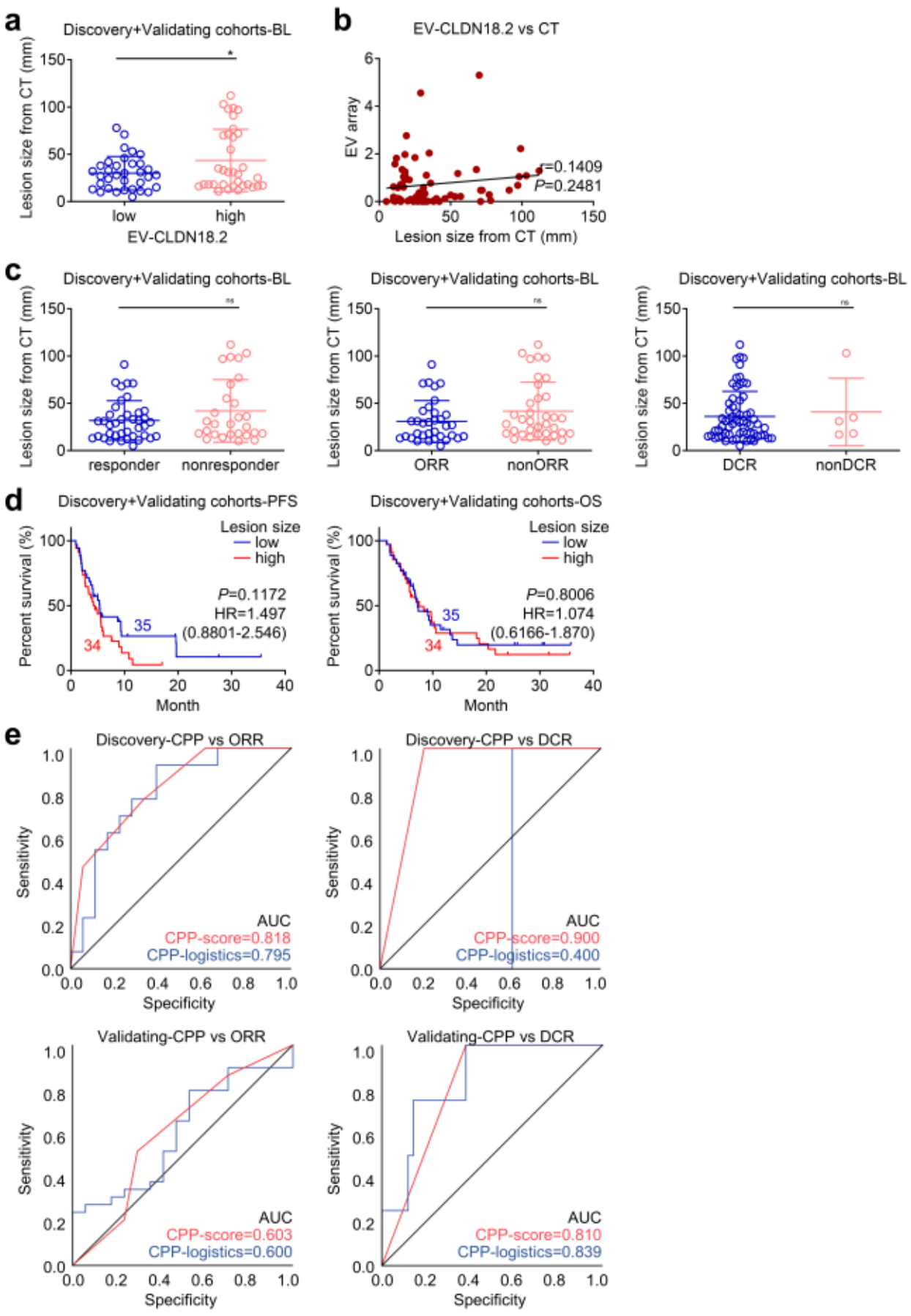

Fig for review only 2. For the two CLDN18.2-CAR-T cohorts, we compared the **(a)** radiographic tumor burden between patients defined as baseline EV-CLDN18.2-low and -high, calculated the **(b)** Pearson correlation between radiographic tumor burden and baseline EV-CLDN18.2 expression, compared the **(c)** radiographic tumor burden between patients defined as responser/nonresponder, ORR/nonORR, DCR/nonDCR, assessed the **(d)** PFS/OS stratified by the median of radiographic tumor burden, and performed the **(e)** ROC analysis of ORR/DCR for CPP-logistics and CPP-score.

In Supplementary Table S6, the authors claim that EV markers are independent prognostic markers at baseline of CAR-T response. However, p values are not showing statistical significance for all three markers, especially with OS as endpoint. CLDN18.2 EV seems more consistent. The authors should revise accordingly.

A. Thank you for the suggestion, we have revised the description to “For both discovery and validating cohorts, COX multivariate analysis confirmed EV-CLDN18.2/EV-PD1/EV-PD-L2 as more important prognostic markers than CLDN18.2-H-score”.

In the Page 10, the authors claim that: "These data indicate that CPP-score reflects higher tumor burden and more immunosuppressive

TIME.". Is it possible the authors refer to "tumor mutational burden" rather than "tumor burden" since they analyze CNV, Myc amplification...

A. Thank you for the suggestion, we have revised the description to "These data indicate that CPP-score reflects more tumor-associated genetic changes and more immunosuppressive TIME".

2) In Figure 3a, the authors show that CAR-T cells are positive for PKH26-labeled EVs. Negative control is CAR-T alone but given that PKH26 form aggregates and false positive signals, PKH26 dye in medium should be used as negative control. Methodology mentions purification of EVs post-PKH26 labeling using an EV purification kit from Echobiotech). More details on the purification kit is needed to evaluate the purity post-PKH26 labeling.

A. Thank you for the question. To address this concern, we performed fluorescent staining for PBS alone (blank control), PBS+PKH26 (negative control) and EV-CLDN18.2+PKH26 (positive control). As shown below, the PKH26-stained PBS group exhibited negligible fluorescence signals compared to the PKH26-labeled EV group, confirming that the red fluorescence observed in Fig. 3a primarily originates from pre-stained EVs rather than free dye.

For fluorescence uptake experiment, PKH26-labeled EVs were re-purified using an EV purification kit (ES9016, Echobiotech) to eliminate unincorporated dye. This kit operates based on molecular sieving and size-exclusion chromatography (SEC). A volume of 50 μL of stained EVs was processed according to the manufacturer's instructions. We performed fluorescent imaging for PKH26-stained EVs before and after re-purification, with PBS and PKH26-stained PBS as blank and negative control (**revised Extended Data Fig. 7f**). Comparable signal intensity was observed for PKH26-stained EVs before and after re-purification, indicating high quality of the re-purified EVs. Further methodological details regarding this purification step have been added to the revised manuscript. We have also provided an improved **revised Fig. 3a**.

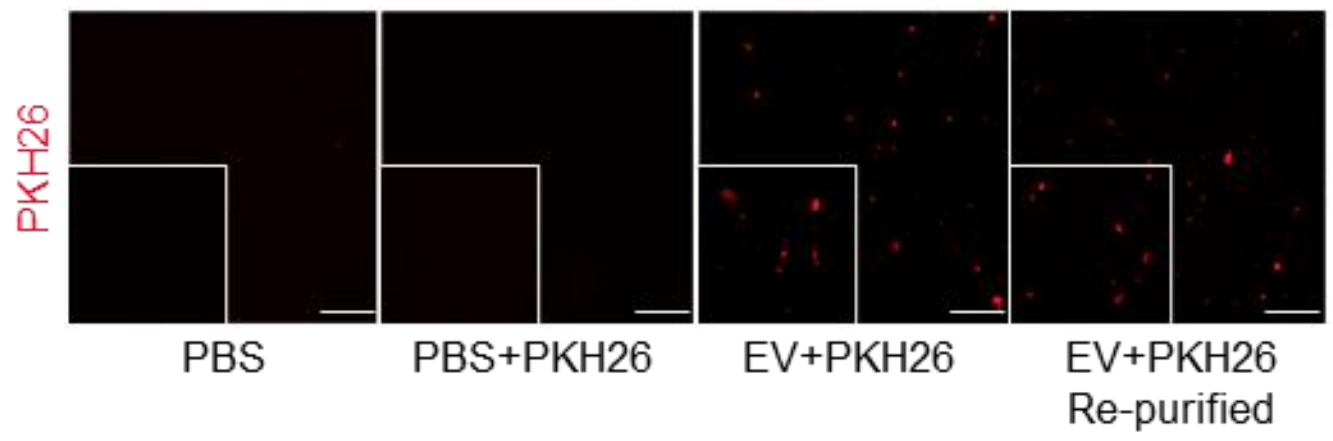

Revised Extended Data Fig. 7f

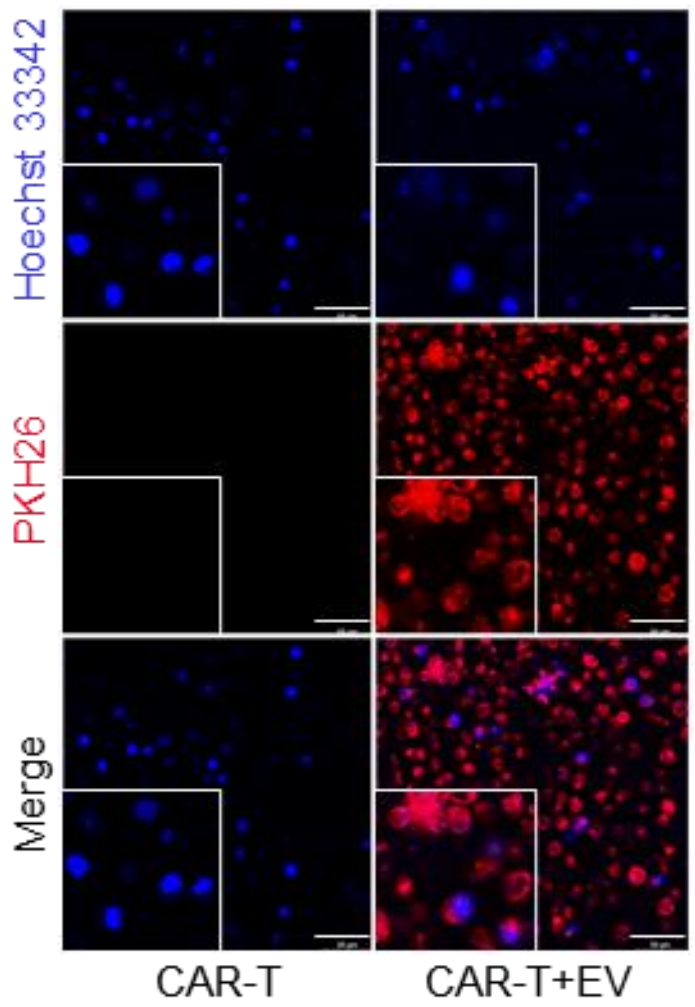

Revised Fig. 3a

Importantly, there is no mention of the EV-CART ratio used and co-culture duration in uptake and functional assays. Are they representative of what is found in patients?

A. Thank you for the comment, we apologize for not having provided detailed methodology. For the EVs uptake assay, PKH26 pre-stained EVs (10 µg/mL) were co-cultured with Hoechest 33342 pre-stained CAR-T cells or CAFs (1×10^6 cells/mL) for 12 hours. For functional assay, EV-CLDN18.2 (10 µg/mL) and EV-PD1 (10 µg/mL) were co-cultured with cells for 24 hours in 1 mL system. For HGC27/AGS-related functional assay, 3×10^5/9×10^5 CAR-T cells were used at respective E:T ratios. We have mentioned these parameters in **Methods** section.

These experiments were performed to illuminate the *in vitro* crosstalk between EV-CLDN18.2 and cells. However, due to the complexity of EV contents in plasma, we did not acquire data for the concentration of EV-CLDN18.2 in patient plasma. We hope to solve this question in future translational researches.

3) In Figure 3i, 3j, Extended Fig 7, data show 1 replicate from 1 experiment which is underpowered to draw any conclusions.

A. Thank you for the comment. In **revised Fig. 3i** and **revised Fig. 3j**, we have provided replicate data for the flow cytometry quantification of CAR-T #1, while retaining representative plots for each exhaustion marker.

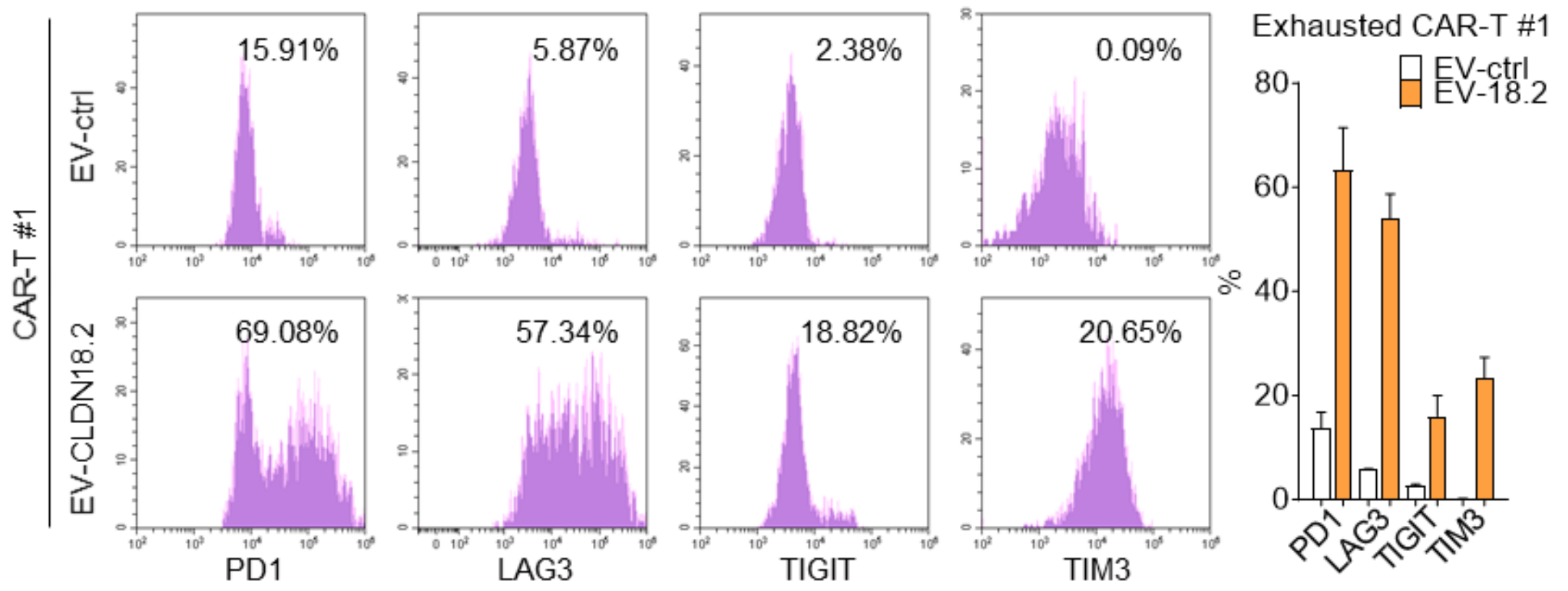

Revised Fig. 3i

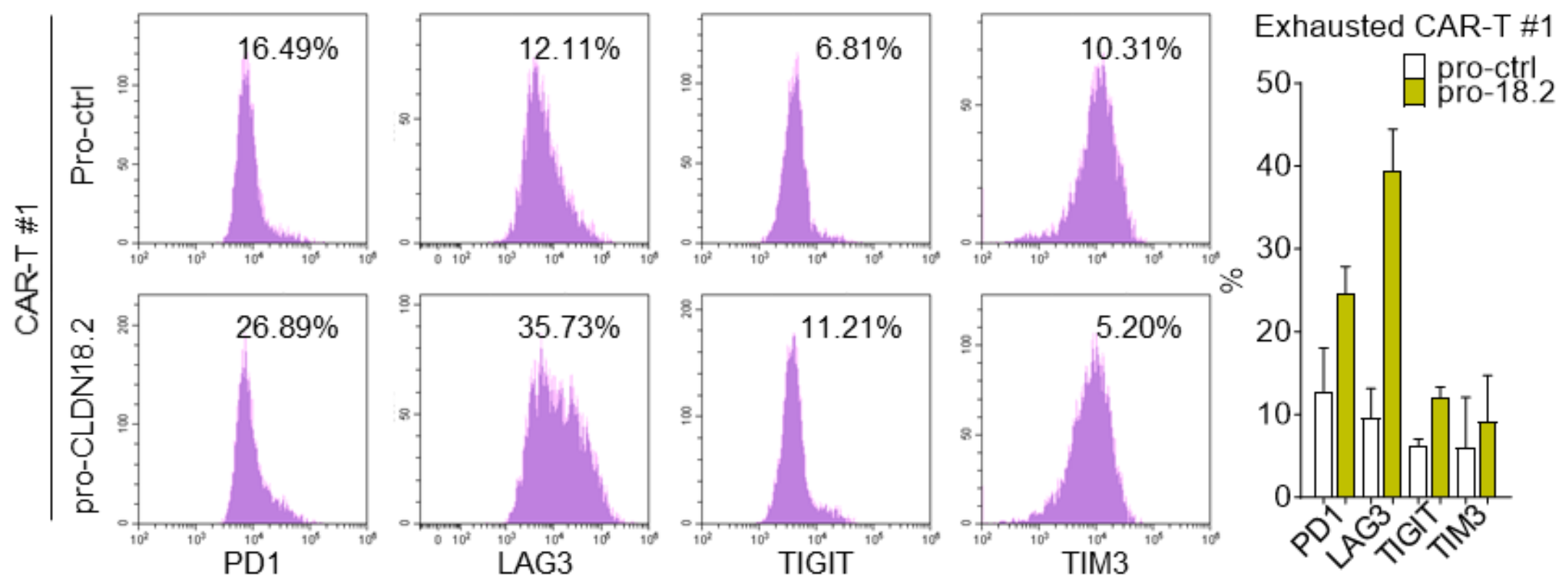

Revised Fig. 3j

Due to the limited time and resources during the revision period, we were unable to include replicate quantification data for CAR-T #2-#4 (Extended Data Fig. 7j-7k). However, we believe that the inclusion of replicates for CAR-T #1, along with the consistent results observed across different CAR-T strings, provides strong and reliable evidence that EV-proteins or soluble proteins induce CAR-T cell exhaustion.

Part3: CLDN18.2 EVs interacts with CAFs promoting the release of IL-6 blocking CAR-T efficacy:

1) The concerns for the PKH26 experiment described previously apply here. Co-culture details (time and EV-cell ratio) must be provided.

A. Thank you for the comment. For the EVs uptake assay, PKH26 pre-stained EVs (10 μg/mL) were co-cultured with Hoechest 33342 pre-stained CAR-T cells or CAFs (1×10^6 cells/mL) for 12 hours. We have updated these parameters in **Methods** section.

2）The authors had a good idea to use a proximity labeling system (TurboID) to identify interacting partners of CLDN18.2-expressing tumor cells in vivo. However, there is a lack of methodological details on the data acquisition and analysis by mass spectrometry. No validation is provided for the surface expression of engineered proteins in tumor cells. The number of samples included in the experiment is

unknown. The authors do not provide any negative control for the proximity labeling in vivo. Mass spectrometry data obtained from proximity labeling with statistical analysis should be provided in Supplementary files.

A. Thank you for the comment. We apologize for not describing this part in detail in our previous submission, due to the space limitations of text and figure.

As you pointed out, this part is indeed a re-analysis of previous data from our published paper (Liu et al., *Cell Commun Signal*. 2024 Jan 10;22(1):27), whose corresponding authors (Cheng Zhang and Lin Shen) are also corresponding authors in this study. In that paper, we fused and expressed biotin ligated TurboID in the extracellular segment of CLDN18.2 as the experiment group (CLDN18.2-TurboID-TM), while used a plasmid with only biotin ligated TurboID as the negative control group (SP-TurboID-TM). We constructed AGS cells stably expressing SP-TurboID-TM or CLDN18.2-TurboID-TM with lentivirus. Through immunoprecipitation with biotin followed by western blot, we have proved that CLDN18.2-TurboID was solely expressed in AGS cells transfected with CLDN18.2-TurboID-TM (**Cited Fig. 6d**). Given the fact that CLDN18.2 is a transmembrane protein (*Ann Oncol*. 2021 May;32(5):609-619; *Clin Cancer Res*. 2008 Dec 1;14(23):7624-34; *Chin J Cancer Res*. 2024 Feb 29;36(1):78–89), we did not pay extra attention to its expression on cell surface.

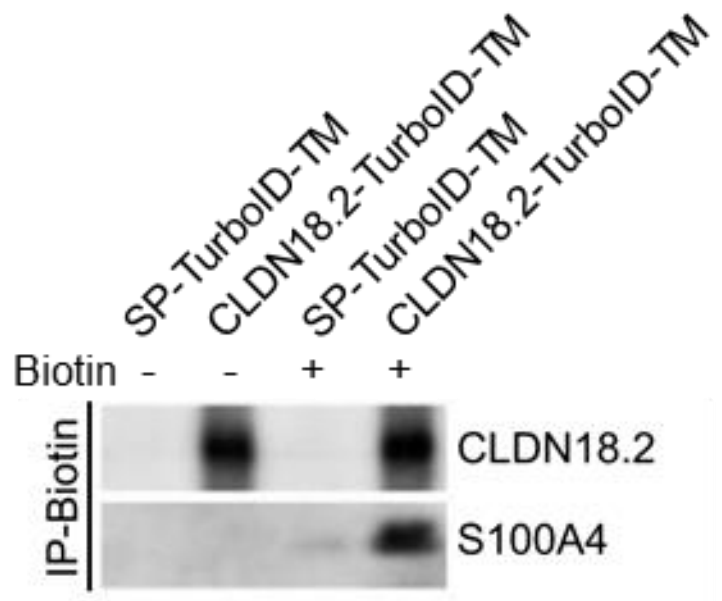

Cited Fig. 6d from our previously published paper (Liu et al., *Cell Commun Signal*. 2024 Jan 10;22(1):27).

Next, AGS cells stably transfected with SP-TurboID-TM or CLDN18.2-TurboID were employed to develop subcutaneous CDXs in nude mice model. After intraperitoneal injection of biotin, the tumor tissue from CDX models was subjected to proximity-dependent biotin identification. Proteins enriched by the *in vivo* proximity labeling assay were assessed through liquid chromatography-tandem mass spectrometry (LC-MS/MS) analysis, with two replicates of CDXs assessed for each group. Each protein's expressional fold changes between CLDN18.2-TurboID-TM and SP-TurboID-TM groups were calculated to represent their interaction affinity with CLDN18.2. Analysis like PCA, data distribution, and Venn diagram showing the overlapped & unique proteins detected in different groups have been reported by our published paper (Liu et al., *Cell Commun Signal*. 2024 Jan 10;22(1):27), as shown in **cited Fig. 6e-6g**.

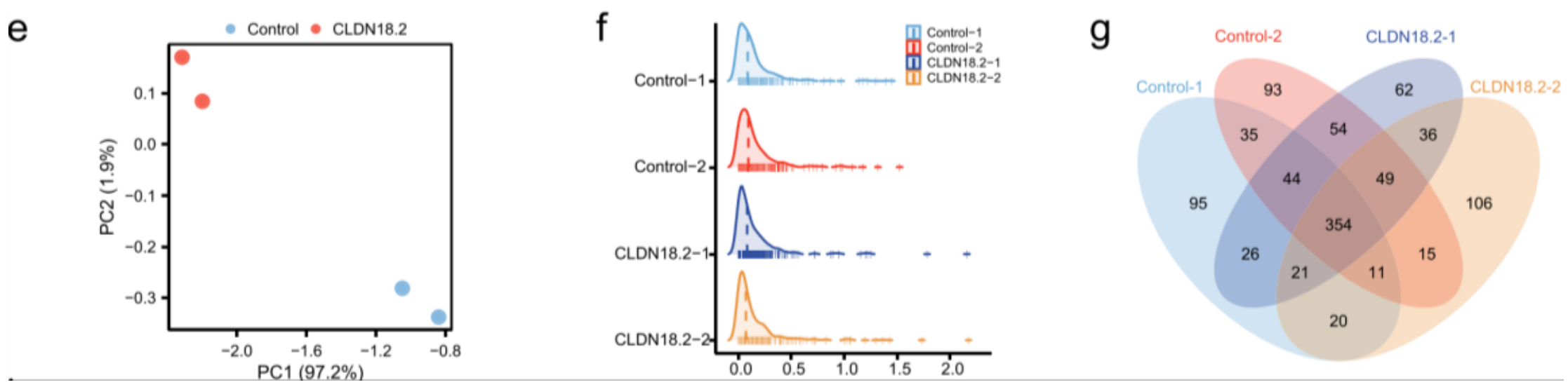

Cited Fig. 6e-6g from our previously published paper (Liu et al., *Cell Commun Signal*. 2024 Jan 10;22(1):27). **(e)** PCA of MS-data. **(f)** Distribution of MS-data. **(g)** Overlapped proteins and unique proteins associated with tumor tissue expressing TurboID-CLDN18.2.

In this current study, by reanalyzing the LC-MS data, we have updated the heatmap plot with replicate data and expressional fold changes between CLDN18.2-TurboID-TM and SP-TurboID-TM groups, which listed the proteins with highest 10 and lowest 10 interaction affinity with CLDN18.2 (**revised Fig. 4o**). We have also provided detailed methodology in the revised manuscript, and attached the mass spectrometry data from proximity labeling in the revision as the **Supplementary Table S9**.

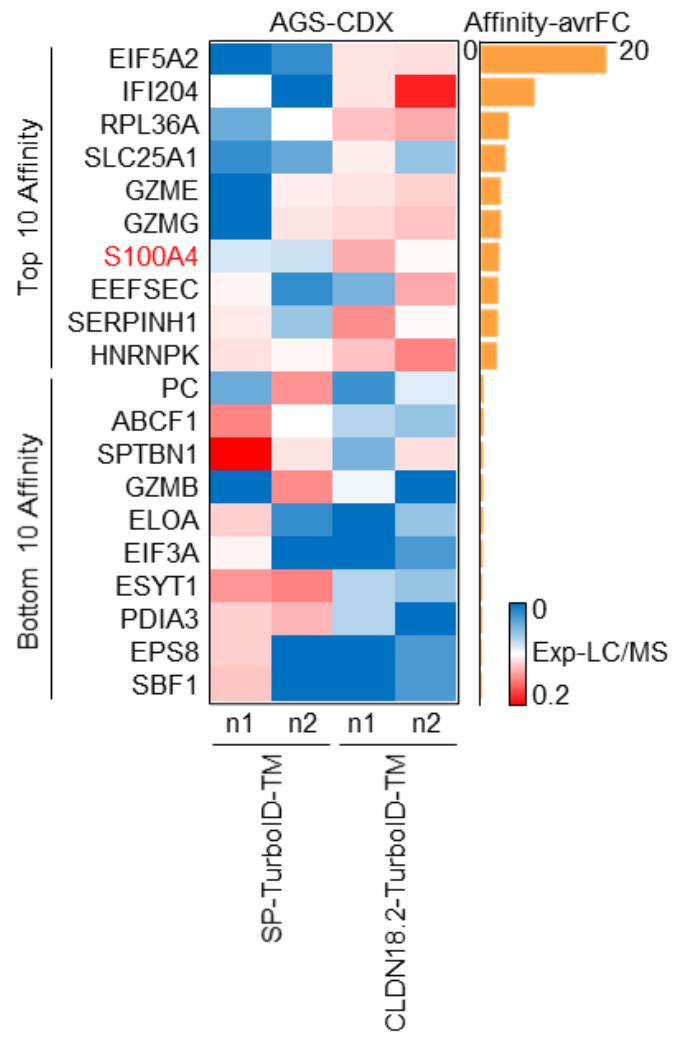

Revised Fig. 4o

3) The authors identify S100A4 as a CAF-associated marker highly present in tumors with CLDN18.2 oe. The authors should provide a more detailed analysis of their proximity labeling data such as number of proteins detected in ctrl and oe conditions, differential expression analysis and pathway enrichment analysis. Was S100A4 the only CAF-derived interaction of CLDN18.2 tumor cells? What was the rationale to select S100A4? The authors recently published the same experiment in 2024 https://biosignaling.biomedcentral.com/articles/10.1186/s12964-023-01406-8 and it is unclear what is different between the two proximity labeling experiments. In this submitted manuscript, it seems to be a reproduction of the data previously generated.

A. Thank you for the comment. As you pointed out, this part is indeed a re-production of previous data from our published paper (Liu et al., *Cell Commun Signal*. 2024 Jan 10;22(1):27), whose corresponding authors (Cheng Zhang and Lin Shen) are also the corresponding authors in this study. Since the purpose of this study is to investigate the interaction between EV-CLDN18.2 and CAFs, we directly referred to the data in our previous paper to introduce the interaction of CLDN18.2 with CAF-related molecules. After developing AGS-CDXs for CLDN18.2-TurboID-TM and SP-TurboID-TM groups, we performed proximity labeling assay and assessed proteins in CDXs through liquid chromatography-tandem mass spectrometry. Analysis like PCA, data distribution, and Venn diagram showing the overlapped & unique proteins detected in different groups have been reported by the paper (**cited Fig. 6e-6g** from our previously published paper (Liu et al., *Cell Commun Signal*. 2024 Jan 10;22(1):27), as shown above).

However, due to the substantial amount of content already included in this manuscript, a detailed investigation of the LC-MS proximity labeling data falls beyond the scope of the current study. Additionally, since the LC-MS data primarily reflect the diversity of CLDN18.2-associated protein interactions rather than direct molecular functional changes, we did not pursue further analyses such as differential expression or pathway enrichment. Instead, we calculated expression fold changes between the CLDN18.2-TurboID-TM and SP-TurboID-TM groups, which reflect the relative interaction affinity with CLDN18.2. We then listed the top 10 and bottom 10 proteins based on interaction strength. Among these proteins, S100A4 is most closely associated with CAF function. To support this, we now provide a revised heatmap (**Fig. 4o**) showing replicate expression levels and fold changes for these 20 proteins.

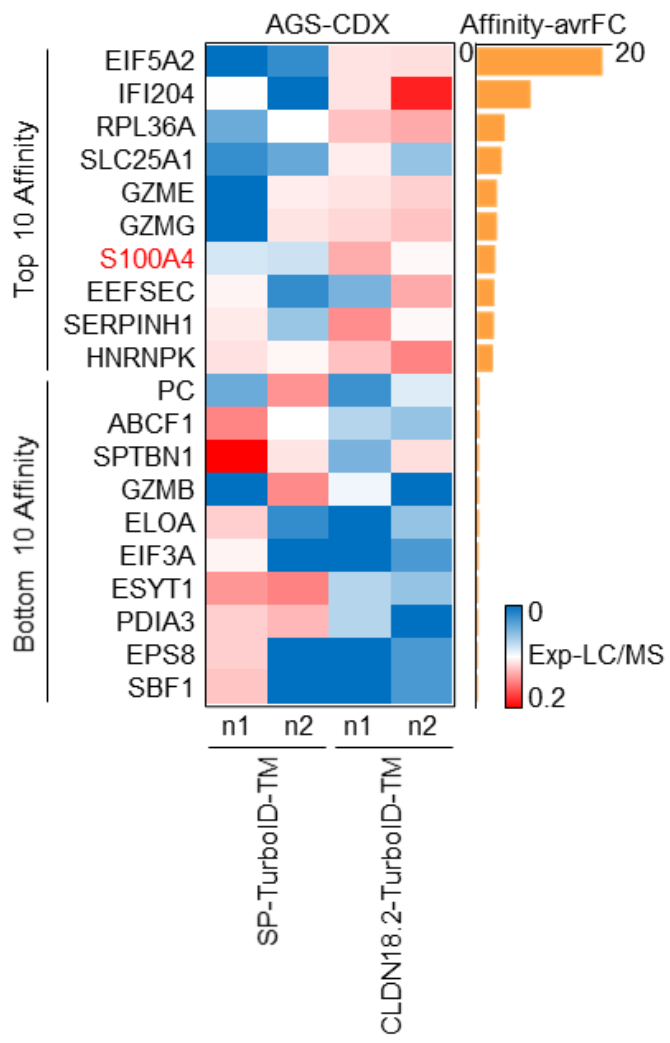

Revised Fig. 4o

We are also providing extra evidences referring to the CAF marker genes for your reference, among which S100A4 shows the strongest interaction affinity with CLDN18.2 than other CAF marker genes (**revised Fig. 4p**). Therefore, we selected S100A4 for subsequent analysis. While other proteins may also mediate the interaction between CLDN18.2 and CAFs, they were not the focus of this study.

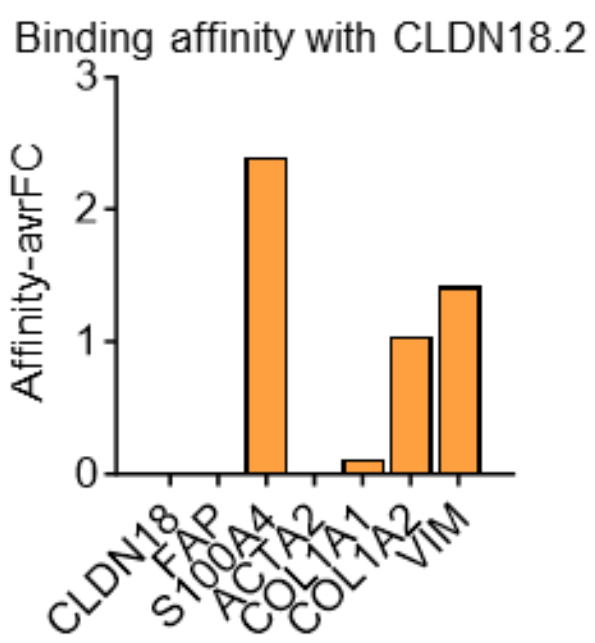

Revised Fig. 4p

4) Did they authors attempt to validate the interaction of CLDN18.2 EVs with S100A4 in vitro?

A. Thank you for the suggestion. As mentioned above, we have proved the interaction between CLDN18.2 and S100A4 in a previously published paper (Liu et al., *Cell Commun Signal*. 2024 Jan 10;22(1):27). We apologize for not providing strong evidences to address the interaction between EV-

CLDN18.2 and S100A4.

We have verified the interaction between EV-CLDN18.2 with S100A4 through an *in vitro* pulldown assay. We used beads for protein G pre-bounded with S100A4 antibody to combine S100A4 protein in cell lysate of CAFs isolated from a GC patient, then used the system to pulldown EV-ctrl or EV-CLDN18.2 isolated from HEK293F cells. Western Blot measurement for CLDN18.2 showed that S100A4 pulled-down more EV-CLDN18.2 than EV-ctrl (**revised Fig. 4s**).

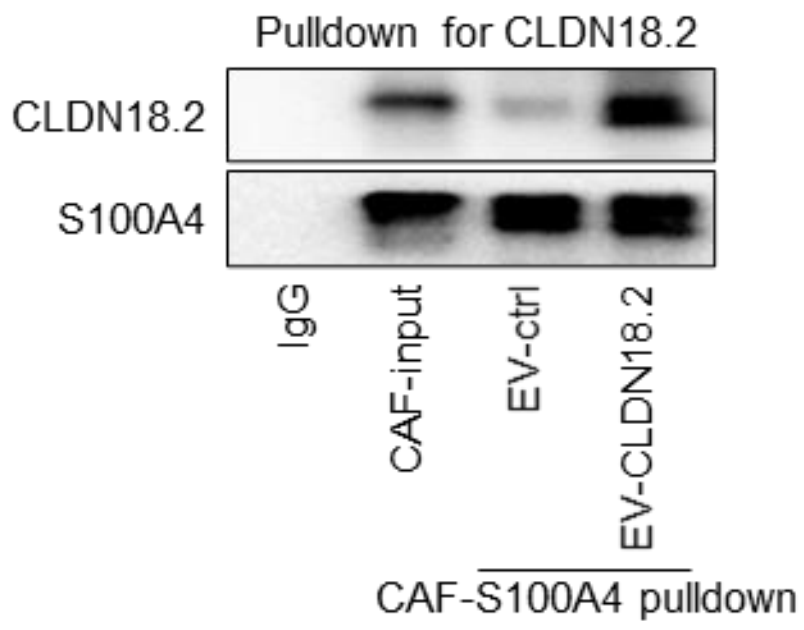

Revised Fig. 4s

For more precise investigation, we applied an *in vitro* binding affinity assay with purified S100A4 protein and isolated EVs. EV-ctrl/EV-CLDN8.2 were pre-immobilized in a 96-well microplate, incubated with recombinant human S100A4 protein, then probed with anti-S100A4 primary antibody and HRP-conjugated secondary antibody to measuring the absorbance at 450 nm. Purified S100A4 protein possesses stronger binding affinity with EV-CLDN18.2 than EV-ctrl (**revised Fig. 4t**).

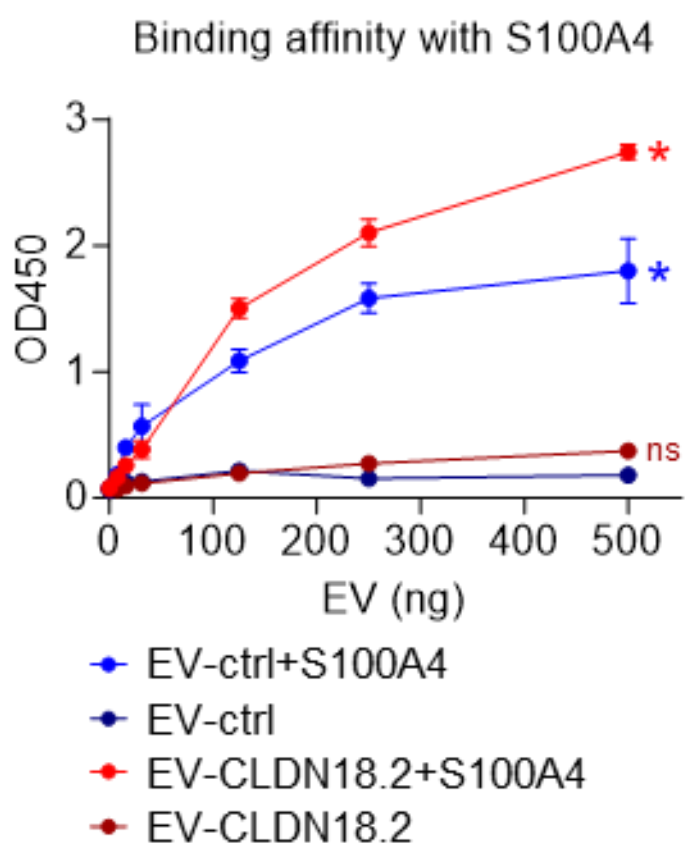

Revised Fig. 4t

Taken together, these *in vitro* data strongly support our conclusion that EV-CLDN18.2 interacts with S100A4.

5) In Figure 5, the authors show that CLDN18.2 EVs prevent CAR-T therapy in vivo. How did the authors measure EV-CLDN18.2 in plasma of mice treated with CLDN18.2 protein? How did they rule out the detection of soluble proteins?

A. Thank you for the question. EV-CLDN18.2 level in PDX plasma was measured by EV-array. As we

have described in **Methods**, EV-array reflects EV protein expression normalized by EV markers, without the need for EV isolation. In this assay, selected panel proteins are illuminated with biotinylated antibodies of EV markers, and then normalized with EV-markers during quantification (each EV-protein's absolute expression was normalized into relative expression by dividing the geometric mean of CD81 and CD9). The rationale ensures that only proteins encapsuled by vesicles expressing EV-markers, rather than soluble proteins, can be quantified by this assay. The specificity of EV-array has been verified by several studies (Jørgensen et al, *J Extracell Vesicles*. 2013 Jun 18:2; Zhang et al., *J Extracell Vesicles*. 2022 Apr;11(4):e12209; Dai et al., *J Extracell Vesicles*. 2025 Apr;14(4):e70054). We also applied quality-control procedures including TEM, NTA, western blot and immunogold labeling assay to ensure that proteins screened by EV-array are expressed on plasma EVs (**revised Extended Data Fig. 1**). In the revised manuscript, we have also improved related descriptions to these results.

6) In Figure 5b, the authors showed that CLDN18.2-EVs are higher in plasma of mice harboring CLDN18.2 positive xenografts compared to those with negative expression. It is different from patient data showing no correlation between CLDN18.2 EVs and tissue CLDN18.2 expression (Fig.1). The authors should discuss this discrepancy. Is there an association of plasma levels of CLDN18.2-EVs and tumor volume in PDX models?

A. Thank you for the question. In patient plasma we did observe no significant correlation between EV-CLDN18.2 and tissue-CLDN18.2 expression. Importantly, it has been reported that CLDN18.2 can be expressed by both normal stomach tissue and gastric adenocarcinoma (*Clin Cancer Res*. 2008 Dec 1;14(23):7624-34; *Chin J Cancer Res*. 2024 Feb 29;36(1):78–89). In normal stomach tissue, CLDN18.2 is strictly confined to differentiated epithelial cells of the gastric mucosa, which is embedded in the tight junctions. Upon malignant transformation of stomach, CLDN18.2 become physically exposed along with tissue disruption, providing epitopes for anti-cancer therapies (*Ann Oncol*. 2021 May;32(5):609-619). Since extracellular vesicles can be released by all tissue cells regardless of their physical positions and can finally converge into blood circulation, we believe plasma EV-CLDN18.2 is originated from both CLDN18.2-expressing normal or tumor epithelial cells in human stomach, which explains the irrelevance between plasma EV-CLDN18.2 and tumor tissue-CLDN18.2 expressions in human samples.

In terms of PDX, CLDN18.2-/+ gastric cancer tissues from patients are engrafted into mice models. Since cancer cells are the most significantly enriched cell types with the growth of xenograft tissue, EV-CLDN18.2 in PDX plasma is majorly contributed by these engrafted CLDN18.2+ cancer cells. Additionally, the human EV-CLDN18.2 in PDX plasma was solely derived from engrafted patient tumor tissues, rather than from mice. In this work, all EV-CLDN18.2/tissue-CLDN18.2 are assessed by antibodies for human-CLDN18.2, therefore we can rule out the interreference by mouse CLDN18.2 expressed in normal mouse stomach tissue. Because these evidences strongly prove EV-CLDN18.2 is derived from engrafted CLDN18.2+ PDX tissue, we did not investigate the association of plasma levels of EV-CLDN18.2 and tumor volume in PDX models.

This discrepancy has been discussed in the revised manuscript as "Notably, patients' plasma EV-protein expressions did not correlate with tissue/cell counterparts, confirming differences between plasma EV-based and tissue-based TIME, but EV-CLDN18.2 was higher in CLDN18.2-positive PDX mice. This discrepancy between patients and mice could be due to that both tumor and normal epithelial cells contributes to the production of human plasma EV-CLDN18.2, whereas in PDXs only engrafted

CLDN18.2+ tumor cells contribute to mice plasma EV-CLDN18.2".

6）The authors use 200 micrograms of EVs or 10 ug of soluble proteins twice a week in their in vivo experiments. It is a huge amount probably in supra physiological levels compared to circulating CLDN18.2 EV levels in patients. What is the lowest concentration to significantly block CAR-T efficacy? The authors should discuss this.

A. Thank you for the question, it should be 200 μg of EVs or 100 μg of soluble proteins for *in vivo* experiments, we apologize for mistakenly described it as 10 μg in the original submission.

In our *in vitro* assays, T cells were added with an effector-to-target (E:T) ratio of 1:1 for 3×10^5 HGC27 cells (3×10^5 CAR-T cells), or 3:1 for 3×10^5 AGS cells (9×10^5 CAR-T cells), in 1 mL culturing system. We explored the concentration of EV-CLDN18.2/pro-CLDN18.2 and found that culturing with 10 μg/mL EV-CLDN18.2 or 5 μg/mL pro-CLDN18.2 in this 1 mL culturing system (equivalent to 10 μg EVs or 5 μg pro-CLDN18.2) is sufficient to significantly induce CAR-T exhaustion (Fig. 3i-3j, Extended Data Fig. 7i-7j). We subsequently proved that 10 μg/mL EV-CLDN18.2 is sufficient to significantly block CAR-T's *in vitro* efficacy, as we provided in **revised Fig. 3l**.

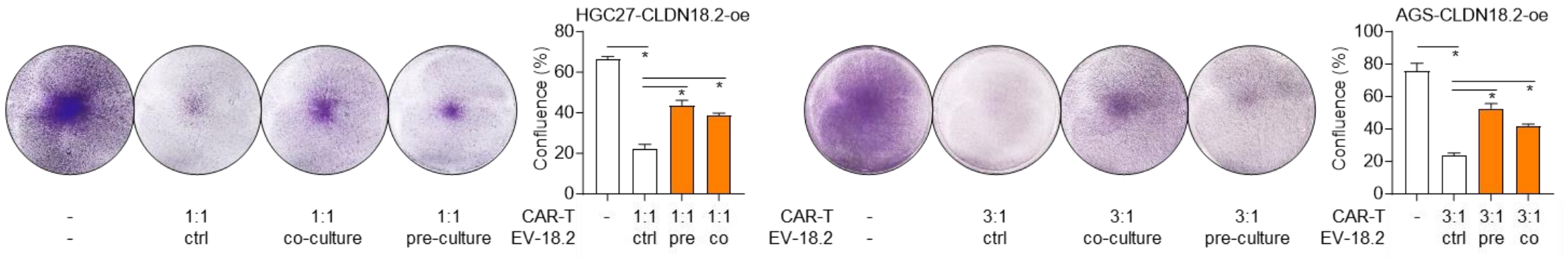

Revised Fig. 3l

For our *in vivo* studies, we intravenously infused 5×10^6 CAR-T cells into each PDX mice, which is around 5.6 to 16.7 times of each *in vitro* system. Therefore, the corresponding *in vivo* dose is theoretically 56-167 μg for EVs and 28-83.5 μg for soluble proteins. Accounting the potential loss during infusion and the fact that total protein concentration of CLDN18.2-expressing EVs does not fully reflect the effective EV-CLDN18.2 level, we administered 200 μg EVs or 100 μg soluble proteins per mouse.

We admit that 200/100 μg is a huge amount in supra physiological levels. Due to the complexity of EV contents in plasma, we did not acquire data for the concentration of EV-CLDN18.2 in patient plasma. However, our PDX experiments and patient re-investigations for plasma exchange (PE) and intraperitoneal infusion (IPI) supported that reducing the physiological levels of circulating EV-CLDN18.2 can enhance CAR-T efficacy. These data proved our conclusion that EV-proteins interferes with corresponding CAR-T or antibody therapies.

We apologize for all the inadequate descriptions and typos. Please find the improved text and figures in the revised manuscript.

7) To prevent the activity of EV-CLDN18.2, the authors ingeniously employed a plasma exchange model. Briefly, 300 microliters of blood was collected retro-orbitally from a CLDN18.2-positive xenograft was centrifuged to remove plasma and purified plasma was mixed with plasma from a CLDN18.2 negative xenograft or tumor-free mice and injected tail vein. It was performed prior CAR-T treatment and

biweekly after treatment. Each PE cycle corresponds to a change of ±15% of total blood. While the approach is interesting, there is no prior literature or technical validation of this PE model. Changes in circulating levels of CLDN18.2 pre and post-infusion would be important to support the efficacy of PE.

A. Thank you for the suggestion. In the original submission, we have measured the circulating levels of EV-CLDN18.2 in PDX plasma on Day 18 of plasma exchange (PE). We now present a **revised Fig. 8c**, in which the plasma EV-CLDN18.2 level of PDX #1 prior to CAR-T infusion and PE (on Day -1) was normalized and presented along with the levels observed on Day 18. This new plot exhibited an accumulation of EV-CLDN18.2 in plasma from Day 0 to Day 18, while PE reduces the level of accumulated EV-CLDN18.2 in plasma.

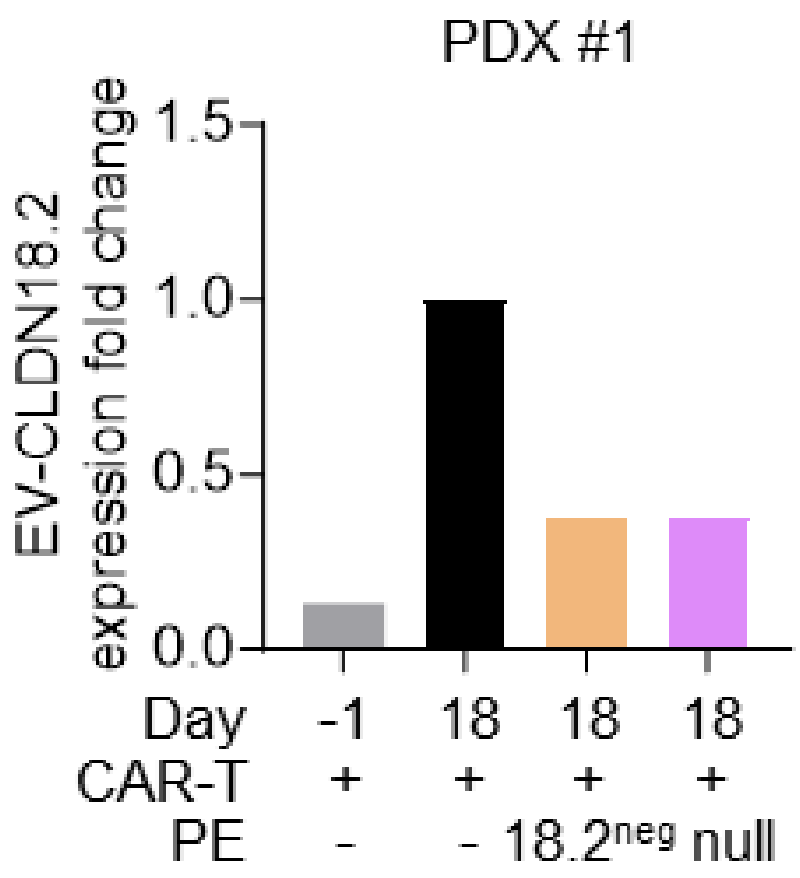

Revised Fig. 8c

8) Figures 7d, 7k, Suppl Fig8 show 1 replicate from 1 experiment which is underpowered to draw any conclusions.

A. Thank you for the question. Fig. 7d demonstrated the impact of EV-PD1 on CLDN18.2-CAR-T cells established from a healthy donor (CAR-T #1). As you suggested, here we are now providing replicate results for CLDN18.2-CAR-T cells established from a different healthy donor (CAR-T #2), which verified the original conclusion that EV-PD1 promoted PD1-featured exhaustion, rather than LAG3/TIGIT/TIM3-featured exhaustion, therefore reduces CAR-T cytotoxicity. These new results have been displayed as a **revised Fig. 7d**.

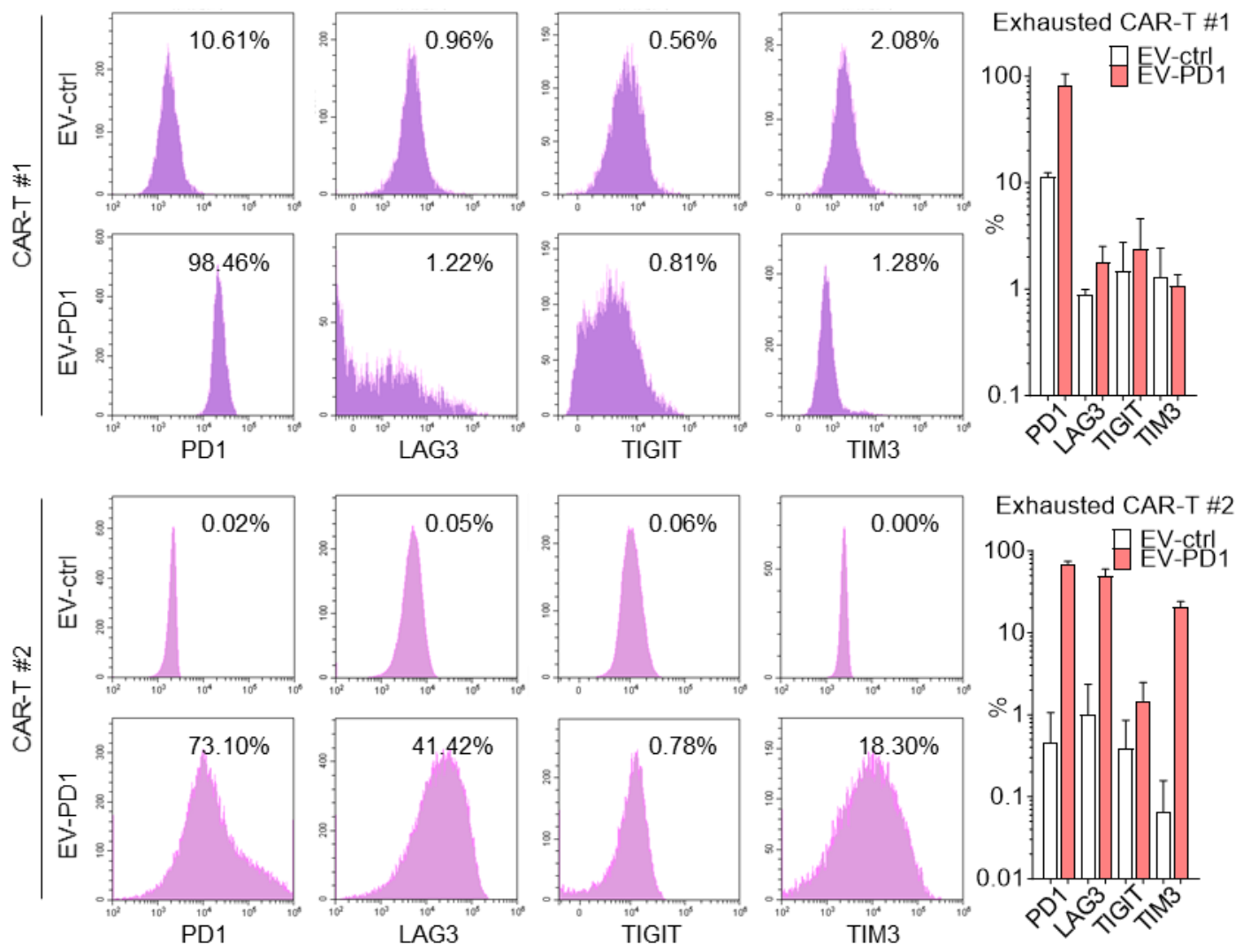

Revised Fig. 7d

For Fig. 7k, we assessed CT26 xenograft tissue with flow cytometry to measure EV-PD-L2 induced changes of TH1 ($CD4^{+}IFN\text{-}\gamma^{+}$) and TH2 ($CD4^{+}IL\text{-}4^{+}$) cells. Due to the small number of infiltrated TH1 and TH2 cells in xenograft tissue, we have to combine all replicate xenografts together for flow cytometry, and achieved only one set of data. However, since one of our previous publications has reported that EV-PD-L2 induces a shift of TH1/TH2 balance towards TH1 (Zhang et al., Plasma extracellular vesicle derived protein profile predicting and monitoring immunotherapeutic outcomes of gastric cancer. *J Extracell Vesicles*. 2022 Apr;11(4):e12209), we believe the data in this work represented reliable results.

For Supplementary Fig. 8, we described the changes of total EV content in plasma from PDX mice and patients. Because of the small volume of mice blood we collected (less than 500 µL/per mice), we have to combine the blood from all mice individuals for each PDXs together to perform EV-array, and achieved only one set of data for each PDXs. However, the fact that PDX #1 and PDX #2 displayed similar data supported our description that EV-CLDN18.2/pro-CLDN18.2 administration, PE (plasma exchange) and IPI (intraperitoneal infusion) all unaffecting the total EV content in plasma. For patients, data from 5 individuals proved accordant phenomenon that IPI induced minimal changes of total EV content in plasma.

Considering the limit of biological sample and time, we sincerely apologize for not being able to provide replicate data for all these parts. However, we believe it does not overshadow the respective conclusions related with these parts, as well as the importance of this study.

Reviewer #3 (Remarks to the Author):

In this manuscript, Qi et al. investigate the effect of extracellular vesicle (EV) proteins on the function of CLDN18.2 CAR-T cells. By profiling EV proteins in patients with gastrointestinal cancers receiving CLDN19.2 therapy or Zolbetuximab, they find that protein expression levels of CLDN18.2/PD1/PD-L2 correlate with patient responses to therapy. The authors demonstrate that extracellular vesicles containing CLDN18.2 protein exhaust CAR-T cells in vitro and lead to diminished efficacy in tumor models. Moreover, several different strategies to counteract EV proteins enhance therapeutic efficiency in mouse models.

The article is well presented and contains an impressive amount of data, ranging from patient data to sophisticated biochemical assays. I think the article should be considered for publication at Nature Communications, yet it would first need to address some concerns. Probably owing to the wide range of techniques employed, I think some key findings lack the sufficient depth and replicates. I detail my concerns below:

- The article is novel since this is the first time, as far as I know, where EV proteins are monitored and measured in patients received CAR T cell therapy. Nonetheless, the detrimental effect of tumor-derived exosomes in CAR T cell functionality has already been described at least in two previous articles: Ukrainskaya et al. Scientific Reports 2023 and Zhong et al. Cancer Research 2023. The authors should cite these two articles, which already showed that tumor antigens in EVs are capable of driving CAR-T cell exhaustion and reduction in cytotoxic capacity.

A. Thank you for the comment. Ukrainskaya et al (*Sci Rep*. 2023 Jan 10;13(1):463) reported that CD19+EVs lead to the activation, systemic exhaustion, and impaired cytotoxic activity of CD19-CAR-T cells. Zhong W et al (*Cancer Res*. 2023 Aug 15;83(16):2790-2806) pointed out that solid tumors release EVs that carry both mesothelin and PD-L1, which preferentially interacted with cognate CAR-T cells and inhibited their proliferation, migration, and function, while inhibiting EV secretion increased CAR-T's infiltration and anti-tumor activity. These reports are consistent with our observations in the context of CLDN18.2-targeted therapies, albeit with less in-depth analysis of the underlying mechanisms. Moreover, Zhong W et al (*Cancer Res*. 2023 Aug 15;83(16):2790-2806) used chemical inhibitors to block exosome secretion, which presents limited clinical relevance and translational potential. also, the possibility of off-target effects associated with the inhibitors cannot be excluded.

In contrast, our study provided more in-depth clinical/mechanistic/translational investigations, and further demonstrating that EV-CLDN18.2 interferes with both CLDN18.2-targeted CAR-T and antibody therapies. Additionally, our study employed plasma exchange (PE) and intraperitoneal infusion (IPI) approaches to depleted harmful EV-proteins in plasma, both PE and IPI offer direct clinical applicability. Therefore, we believe our work offers a more thorough understanding and holds stronger translational relevance.

We deeply appreciate your suggestions to improve the quality of our manuscript. We have discussed the *Sci Rep* and *Cancer Research* papers in the revised manuscript, and have provided more details on the procedures and methods used in our study.

- Correlation between levels of protein tissue expression and protein in extracellular vesicles. The authors show that EV CD19 and CLDN18.2 protein levels do not correlate with tumor tissue levels. On the other hand, in mice the authors observe higher EV-CLDN18.2 in mice with CLD18.2 + tumors. Altogether, this data is difficult to interpret.

A. Thank you for the question. In patient plasma we did observe no significant correlation between EV-CLDN18.2 and tissue-CLDN18.2 expression. Importantly, it has been reported that CLDN18.2 can be expressed by both normal stomach tissue and gastric adenocarcinoma (*Clin Cancer Res*. 2008 Dec 1;14(23):7624-34; *Chin J Cancer Res*. 2024 Feb 29;36(1):78–89). In normal stomach tissue, CLDN18.2 is strictly confined to differentiated epithelial cells of the gastric mucosa, which is embedded in the tight junctions. Upon malignant transformation of stomach, CLDN18.2 become physically exposed along with tissue disruption, providing epitopes for anti-cancer therapies (*Ann Oncol*. 2021 May;32(5):609-619). Since extracellular vesicles can be released by all tissue cells regardless of their physical positions and can finally converge into blood circulation, we believe plasma EV-CLDN18.2 is originated from both CLDN18.2-expressing normal or tumor epithelial cells in human stomach, which explains the irrelevance between plasma EV-CLDN18.2 and tumor tissue-CLDN18.2 expressions in human samples.

In terms of PDX, CLDN18.2-/+ gastric cancer tissues from patients are engrafted into mice models. Since cancer cells are the most significantly enriched cell types with the growth of xenograft tissue, EV-CLDN18.2 in PDX plasma is majorly contributed by these engrafted CLDN18.2+ cancer cells. Additionally, the human EV-CLDN18.2 in PDX plasma was solely derived from engrafted patient tumor tissues, rather than from mice. In this work, all EV-CLDN18.2/tissue-CLDN18.2 are assessed by antibodies for human-CLDN18.2, therefore we can rule out the interreference by mouse CLDN18.2 expressed in normal mouse stomach tissue. These evidences strongly prove EV-CLDN18.2 is derived from engrafted CLDN18.2+ PDX tissue.

This discrepancy has been discussed in the revised manuscript as “Notably, patients’ plasma EV-protein expressions did not correlate with tissue/cell counterparts, confirming differences between plasma EV-based and tissue-based TIME, but EV-CLDN18.2 was higher in CLDN18.2-positive PDX mice. This discrepancy between patients and mice could be due to that both tumor and normal epithelial cells contributes to the production of human plasma EV-CLDN18.2, whereas in PDXs only engrafted CLDN18.2+ tumor cells contribute to mice plasma EV-CLDN18.2”.

One plausible explanation is that CLDN18.2 from healthy tissue also contribute to EV-CLDN18.2 in patients. The authors should measure levels of CLDN18.2 in healthy patients and compare them to the current data) patients with low or high CLDN18.2 tumors).

A. Thank you for the suggestion. As discussed above, CLDN18.2’s expression in normal stomach tissue is strictly confined to differentiated epithelial cells of the gastric mucosa, which is embedded in the tight junctions. Upon malignant transformation of stomach, CLDN18.2 become physically exposed along with tissue disruption, providing epitopes for anti-cancer therapies (*Ann Oncol*. 2021 May;32(5):609-619) Importantly, it has been reported that normal stomach tissue displays comparable transcript or protein levels of CLDN18.2 with gastric adenocarcinoma (*Clin Cancer Res*. 2008 Dec 1;14(23):7624-34; *Chin J Cancer Res*. 2024 Feb 29;36(1):78–89). Since extracellular vesicles can be released by all tissue cells

regardless of their physical positions and can finally converge into blood circulation, we believe plasma EV-CLDN18.2 is originated from both CLDN18.2-expressing normal or tumor epithelial cells.

To address your concern, we collected plasma samples from 30 healthy (noncancerous) donors (all with informed consent), then we isolated EVs and assessed EV-CLDN18.2 with ELISA for these 30 healthy plasma samples, together with 31 available baseline cancer plasma samples (including both low tissue-CLDN18.2 and high tissue-CLDN18.2 patients) in current CLDN18.2-CAR-T cohorts. As shown below (**Fig for review only. 3**), plasma EV-CLDN18.2 level was even higher for healthy donors, while showing minimal differences between low tissue-CLDN18.2 and high tissue-CLDN18.2 cancer patients. This was in accordance with our previous conclusion that plasma EV-proteins possess a different expressional landscape from tissue proteins.

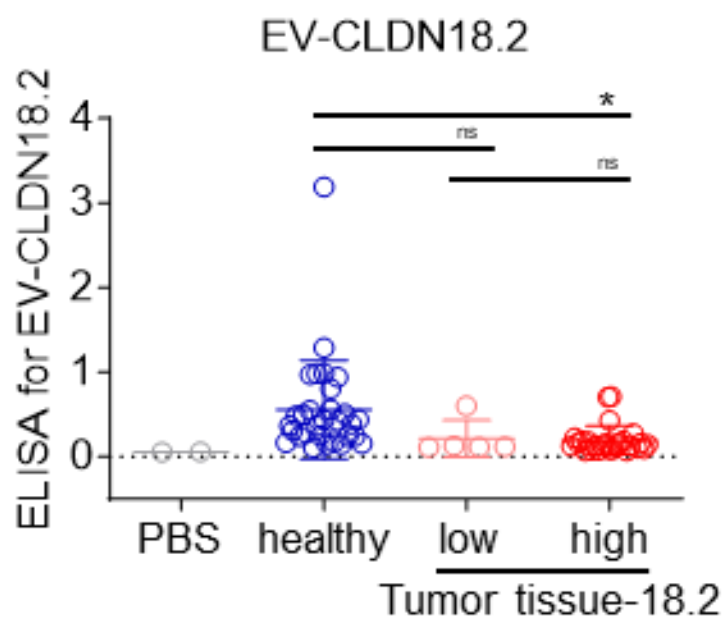

Fig for review only. 3

- Analysis of cytotoxic killing of HGC27-CDL18.2-oe (Figure 3m). The authors cannot conclude that cytotoxicity was impaired from this experiment, the wording must be changed. There is a lot of killing even when treated with EV-CLDN18.2. The axis used generates confusion, please change to a more standard axis (perhaps with a log10 scale). In addition, please explain better in the methods section how this experiment was performed.

A. Thank you for the comment, we apologize for the inadequate descriptions provided in the original submission.

For the real-time cytotoxic killing assay, Y axis represents the normalized index of target cancer cells, indicating the cytotoxic killing by CAR-T cells. Admittedly, there is a lot of killing even when treated with EV-CLDN18.2, which is due to a relatively low level of EV-CLDN18.2 (2/10/20 ng) used in this real-time assay. In contrast, our conventional cytotoxic killing assay demonstrated that EV-CLDN18.2 on μg level significantly impairs CAR-T's efficacy (**revised Fig. 3l**). Given the high sensitivity of this real-time cytotoxic killing assay, we intentionally used EV-CLDN18.2 on ng level to assess its dose-dependent impact on CAR-T cells over time, which leads to relatively modest changes of cytotoxic killing compared with the conventional assay. Therefore, we have modified the conclusion to "EV-CLDN18.2 interferes with CAR-T's cytotoxicity in a dose-dependent manner" and provided more detailed descriptions for this assay in the revised **Method** section,

For the formatting of this assay, the presence of negative values on the Y-axis makes the use of a log10 scale unsuitable, as it would have resulted in the loss of most time points along the X-axis. Therefore, we maintained the linear scale in the revised figure. For your reference, we also present a demonstration

using standard linear Y axis (**Fig for review only. 4**); however, it does not clearly illustrate the dose-dependent impact of EV-CLDN18.2 on CAR-T cells. As a result, we adjusted the linear scale of Y axis to allow a more precise interpretation, which is now presented as the **revised Fig. 3n**.

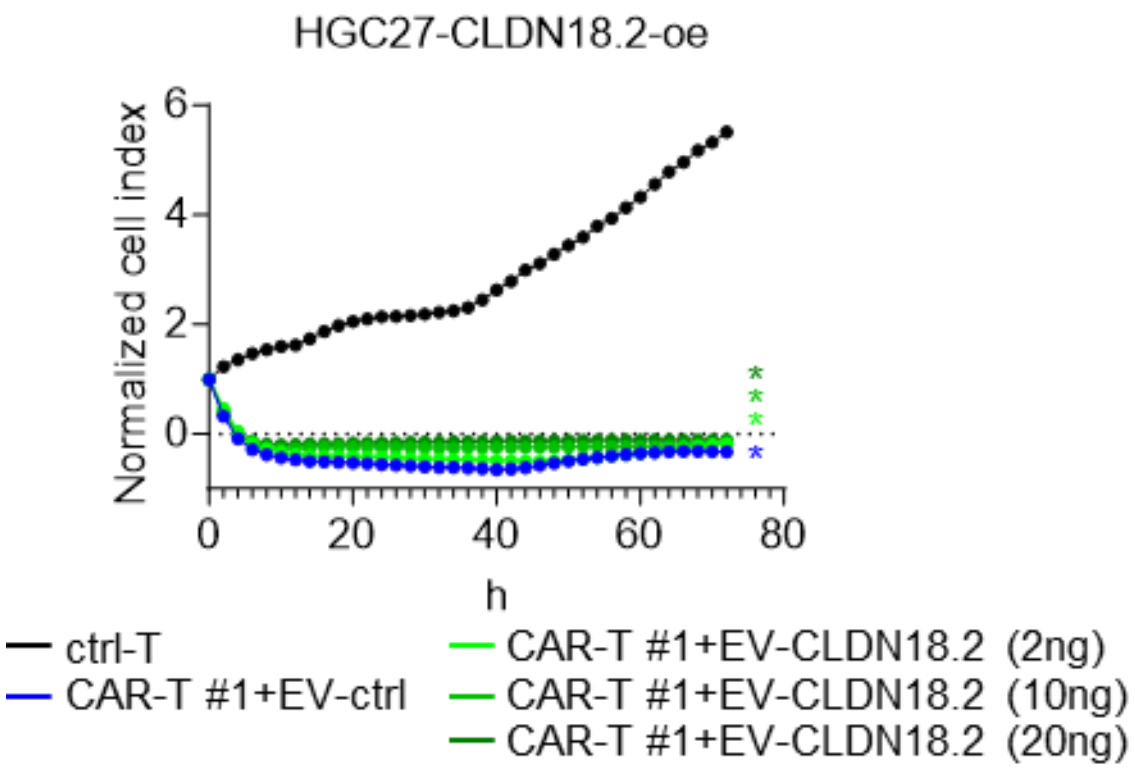

Fig for review only. 4

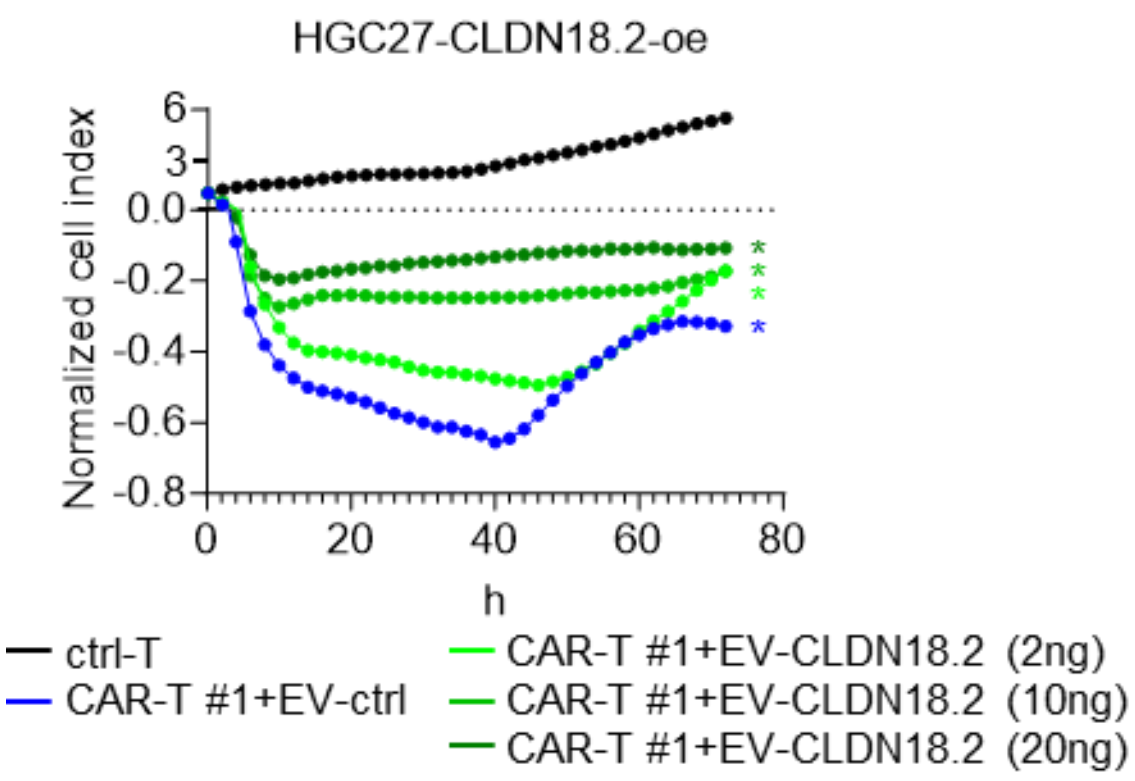

Revised Fig. 3n

This is a key experiment because it shows how killing functionality is affected by EVs. Please perform this experiment using different tumor lines, at different E:T ratios and using either pre-treatment of T cells with EV-CLDN18.2 or adding them during the co-culture. Authors should consider re-challenging the T cells with tumor to see better differences between groups. Likely the exhausted phenotype will become more apparent upon tumor rechallenge. As an example, see Figure 5 from Ukrainskaya et al. 2023.

A. Thank you for the suggestion. We apologize for not providing sufficient details in our previous submission, due to the space limitations of text and figure. Here we present additional data to address your concern. We stably overexpressed CLDN18.2 in gastric cancer cell lines HGC27 and AGS, then performed *in vitro* cytotoxic killing assay with CLDN18.2-CAR-T cells. Among different E:T ratios, 1:1 and 3:1 were sufficient for CLDN18.2-CAR-T-mediated *in vitro* cytotoxicity against CLDN18.2-overexpressing HGC27/AGS cells (**revised Extended Data Fig. 7m**).

By applying these E:T ratios, we verified that pre-culturing CAR-T cells for 12 hours with 10 μg/mL EV-CLDN18.2, or co-culturing CAR-T cells with 10 μg/mL EV-CLDN18.2, can both significantly impair

the 24-hour *in vitro* cytotoxicity of CLDN18.2-CAR-T cells against CLDN18.2-overpressing HGC27 (**revised Fig. 3l**).

Additionally, we also rechallenged CAR-T cells by co-culturing it with CLDN18.2-overpressing HGC27 for three consecutive 24-hour rounds. CAR-T cells subjected to 4 rounds of rechallenge exhibited significantly reduced 24-hour *in vitro* cytotoxicity, which was comparable to fresh CAR-T cells co-cultured with EV-CLDN18.2 (**revised Fig. 3m**).

Below we present the combined new results for your reference. Due to the limited time and resources available of revision, we are unable to include cytotoxicity data from multiple time points. However, we believe the current data are sufficient to demonstrate that CAR-T cells effectively kill target cancer cells, and treatment with EV-proteins impairs this cytotoxicity.

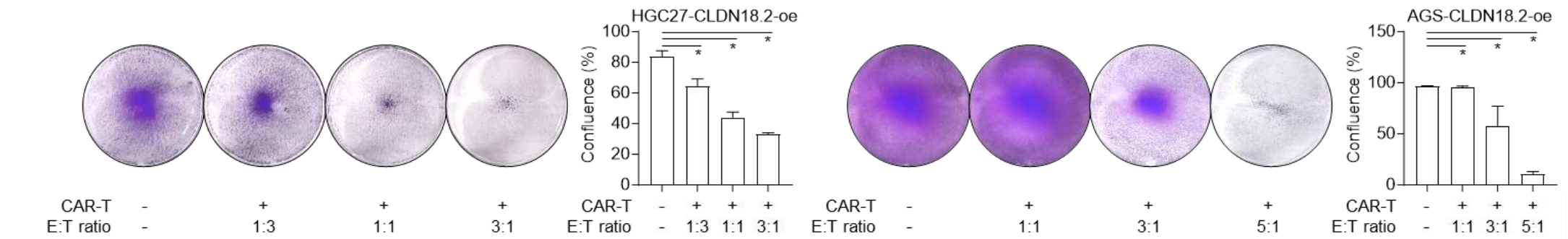

Revised Extended Data Fig. 7m

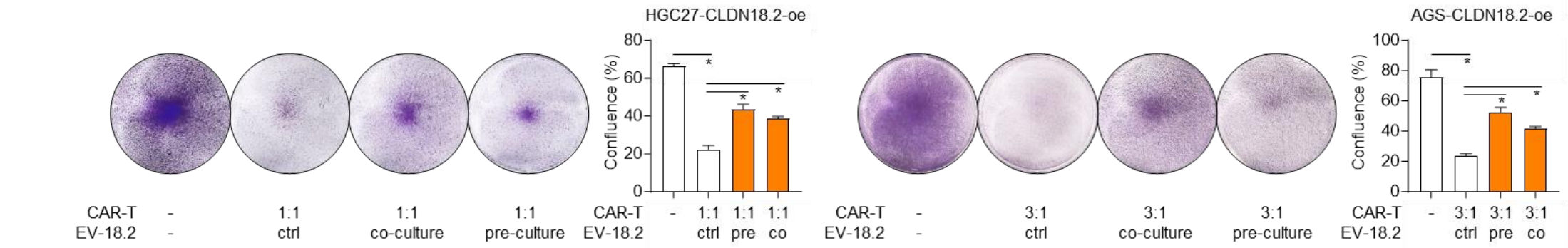

Revised Fig. 3l

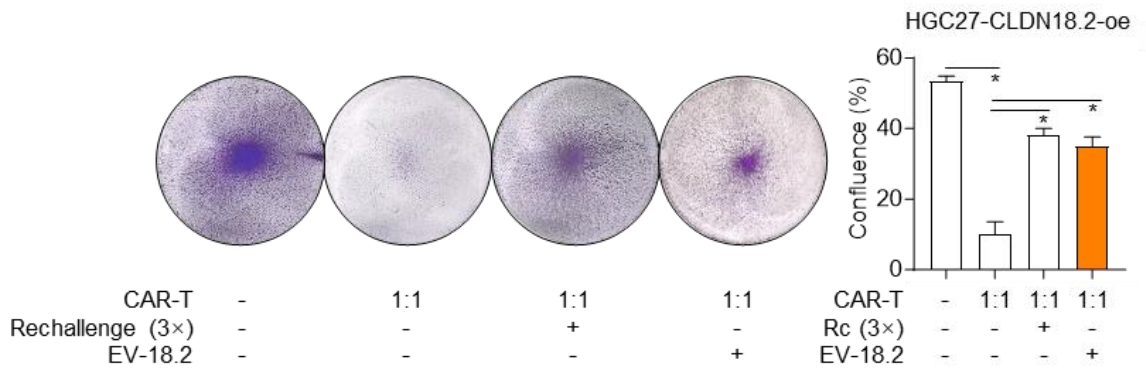

Revised Fig. 3m

- Effect of EV-CLDN18.2 on CAFs.

a) Substitute Figure 4b with a barplot showing biological replicates and assessing significance to be able to demonstrate CXCL8 and IL6 upregulation.

A. Thank you for your valuable suggestion. We apologize for the inappropriate data presentation in the previous submission. In response, we have provided improved heatmaps and bar plots incorporating biological replicates and relevant statistical analysis (t-test) for your reference, which highlight the upregulation of CXCL8 and IL6 following EV-CLDN18.2 administration, as shown below. However, due to the substantial variability in gene expression levels, bar plots are insufficient to outline the inter-group diversities. Also, the limited space available for figures makes it difficult to integrate the bar plots effectively. Therefore, we have opted to retain only the improved heatmaps (left panel) in the **revised Fig. 4b**.

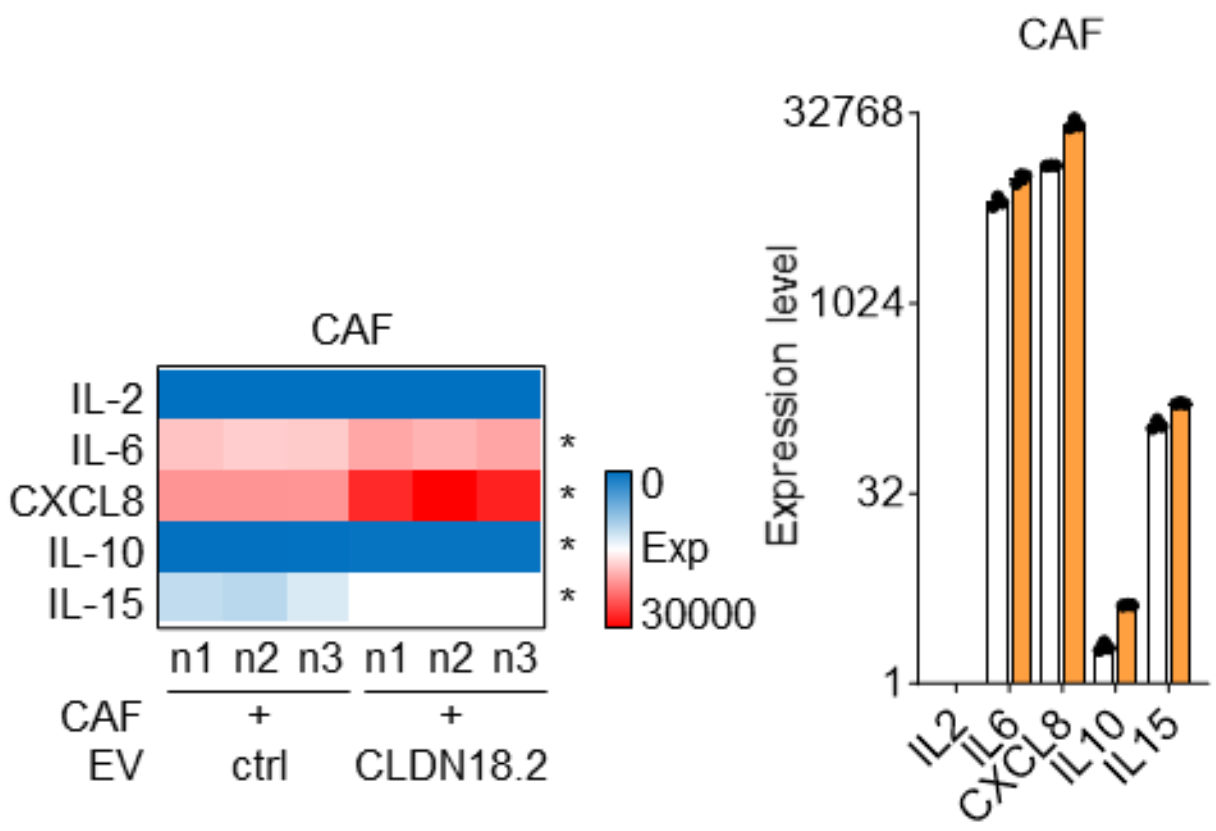

Revised Fig. 4b

b) Please measure IL-6 and CXCL8 secretion in CAFs +/- S100A4 Knock down, treated or not with control of CLDN18.2 EV. Ideally, also perform the experiment using CRISPR knock-outs or using multiple shRNA for knocking down S100A4.

A. Thank you for the suggestion, we have provided additional data to address your concern. We used 3 shRNA sequences for S100A4 knockdown in patient-derived CAFs (**revised Fig. 4u**), then measured their IL-6/CXCL8 secretion with/without EV-CLDN18.2 treatment (**revised Fig. 4v-4w**). EV-CLDN18.2 treatment enhanced the secretion of both IL-6 and CXCL8, while S100A4 knockdown reduced IL-6 level but showed less pronounced impact on CXCL8 level. Notably, EV-CLDN18.2 failed to rescue the reduction of IL-6 caused by S100A4 knockdown, but retained its function to restore CXCL8 secretion. These new findings underscore that EV-CLDN18.2 compromises the efficacy of CLDN18.2-targeted CAR-T cells by stimulating an IL-6–expressing CAF lineage in a S100A4-dependent manner, whereas its effect on CXCL8 expression appears to be less dependent on S100A4.

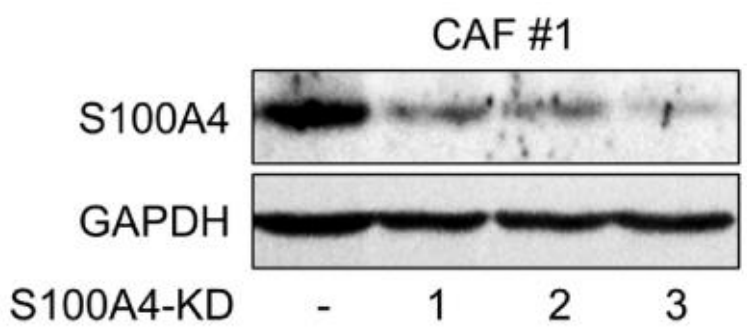

Revised Fig. 4u

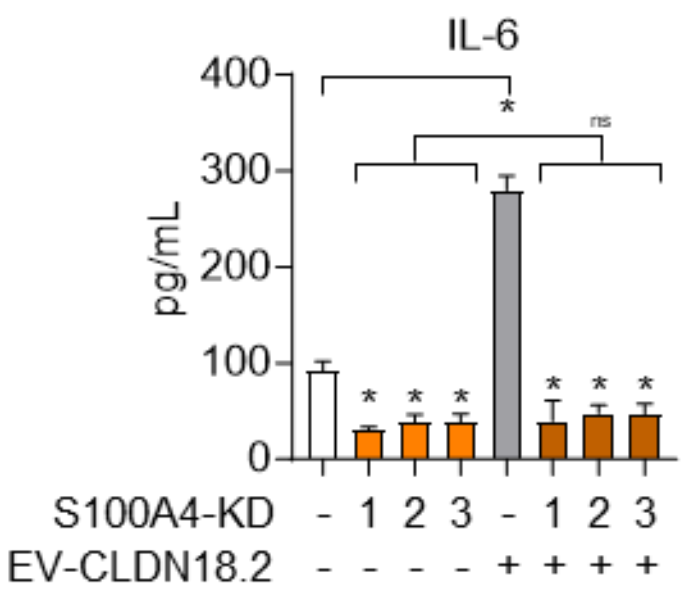

Revised Fig. 4v

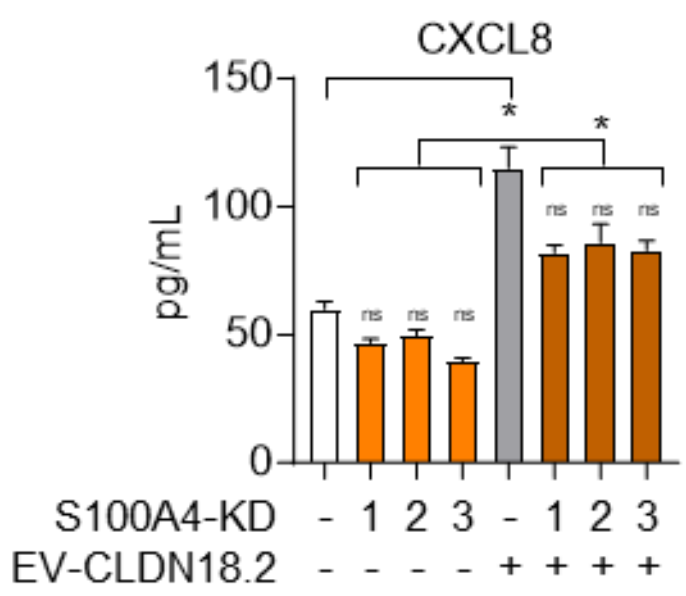

Revised Fig. 4w

c) Is the effect on CAFs also observed with proCLDN18.2?

A. Thank you for the question. As shown in our original Fig. 3j and Fig. 5e-5h, pro-CLDN18.2 exerts an influence on the *in vitro* and *in vivo* efficacy of CLDN18.2-CAR-T cells that is similar to, but weaker than that of EV-CLDN18.2. Notably, administering pro-CLDN18.2 increased plasma EV-CLDN18.2 levels in GC-PDX models, suggesting that pro-CLDN18.2 can be re-encapsulated into plasma circulating EVs. Although this intriguing finding merits further investigation, in the present study we treat pro-CLDN18.2 primarily as a functional mimic for EV-CLDN18.2; therefore, we did not assess pro-CLDN18.2's effects on CAFs.

- In vivo functional effect on CAR-T cells. In vivo data in Figure 5 is particularly important as it shows a functional impact of CLDN18.2 EV in the presence of CLDN18.2 positive PDXs. Although the tumor expansion differences are small, they seem consistent in two different PDXs. I would rather see panels d and e merged (with controls and treatments in the same Figure) and then if the authors consider it necessary, zoom into the selected groups.

A. Thank you for your suggestion. We did attempt to combine the data from both panels into a single plot. However, the tumor volumes in the "control-T" group (gray curves and bars) were substantially larger than those in the CLDN18.2-CAR-T-treated groups, regardless of EV administration. Combining all groups in one panel resulted in an overcrowded Y-axis and overlapping curves, which impaired clarity. Therefore, to ensure clear data presentation, we chose to display the groups separately into panels d and e. This approach more effectively highlights the impact of EV-CLDN18.2 on the efficacy of CLDN18.2-CAR-T therapy.

Moreover, the authors show decreases CAR-T cell numbers that would be consistent with CAR-T cell exhaustion.

- The in vivo data showing combination of Zolbetuximab with CLDN18.2 CAR-T is exciting. How do the authors explain that the antibody is not masking the antigen from CARs? Have the authors whether EV-CLDN18.2 could be also doing some CAR masking that could further contribute to decreased killing.

A. Thank you for the comment. CLDN18.2-CAR-T (Carsgen's CT041) and CLDN18.2-antibody (Astellas' Zolbetuximab) are both designed to recognize CLDN18.2 molecule expressed on target cancer

cells. We have also proved their interaction with EV-CLDN18.2.

Here, we investigated public patent and literature information for both products. CT041's scFv sequence (hu2B1-S54A) was identified in the Carsgen patents CN113227146A and CA3030257A1. Zolbetuximab's sequence is disclosed in MorphoSys/Astellas filings and related patents. From these sources, we extracted the amino acid sequences of the heavy (VH) and light (VL) chain variable regions of the antibodies, as shown below:

Product	Source / Patent No.	Chain Type	Example Amino Acid Sequence Fragment	Humanized
**CT041 (scFv)**	Carsgen (CN113227146A / CA3030257A1)	VH	QVQLVQSGAEVKKPGASVKVSCASGYTFTSYT...CFAYWQGTLVTVSSASTKGPSVFP (~120 aa)	Yes
		VL	DIVMTQSPSASLSASLGERVTLVRSSASTKGPRSPSFQSGQVTLSANSS...KYGTFGGGTKLEIK (~110 aa)	Yes
**Zolbetuximab (IMAB 362)**	Astellas / MorphoSys patents & publications	VH	QVQLQESGPGLVKPSQTLSLTCTVSGGSISTTYWYQQSLRHVPGVPSRFSGSGSGTDFTLTISSLQPEDFATYYC (~115 aa)	No (chimeric)
		VL	DIVMTQSPATLSLSPGERATLSCRASQSVSSYWYQQKPGQAPRLMIYGSTSRSLGHFWIRQRRPGKPPRLQKLG (~110 aa)	No (chimeric)

CT041 utilizes a humanized version of the murine 2B1 antibody (hu2B1-S54A mutant), with full human framework regions. Zolbetuximab is a chimeric IgG1 antibody comprising a murine VH and human VL. Sequence alignment revealed low overall homology between the VH regions of CT041 and Zolbetuximab, with minor similarity limited to the N-terminal framework region ("QVQL"). Crucially, the CDR (Complementarity-Determining Region) sequences for CDR1–CDR3 show significant divergence. CT041's VH CDR3 ends with "…FAYWQG", while zolbetuximab's VH CDR3 terminates with "…GYTGT". Similar divergence is seen in the VL regions. These findings suggest the two regimens bind to distinct epitopes or spatially distant regions on the CLDN18.2 extracellular domain. As a result, we believe that although Zolbetuximab neutralizes EV-CLDN18.2, its binding to CLDN18.2 antigen on cancer cells does not preclude CLDN18.2-CAR-T's function.

On the other hand, we have proved EV-CLDN18.2 treatment both enhances exhaustion (Fig. 3i-3j) and induces the release of IFN-γ/TNF-α by CLDN18.2-CAR-T cells (Fig. 3d). Thus, we believe the decreased killing of CAR-T cells by EV-CLDN18.2 is more likely to be done through hyperactivating CAR-T cells, rather than through CAR masking.

Minor:

- Panel in Figure 1a is clarifying to understand the scope of the study. Good job.

A. We sincerely appreciate your positive evaluation of our work.

- In Figure 5B there are no error bars or replicates, please provide them.

A. Thank you for the comment, we apologize for the lack of data in this part. For Fig. 5b, we described the expression of EV-CLDN18.2 in plasma from PDX mice. Because of the small volume of mice blood we collected (less than 500 μL/per mice), we have to combine the blood from all mice individuals for each PDXs together to perform EV-array, and achieved only one set of data for each PDXs. However, the fact that PDX #1 and PDX #2 displayed similar data supported our description that CLDN18.2-positive xenograft mice harbored higher human-EV-CLDN18.2 expression in plasma than CLDN18.2-negative xenograft mice and tumor-free mice.

Considering the limit of biological sample and time, we sincerely apologize for not being able to provide replicate data for all these parts. However, we believe it does not overshadow the related conclusions, as well as the importance of this study.

- It is not possible to asses images from Fig3a and Fig4a with their current quality. Please provide clearer pictures.

A. Thank you for the suggestion. We apologize for the low quality of the original plots. In response, we have revised **Fig. 3a** and **Fig. 4a** with higher-resolution images and increased magnification for improved clarity.

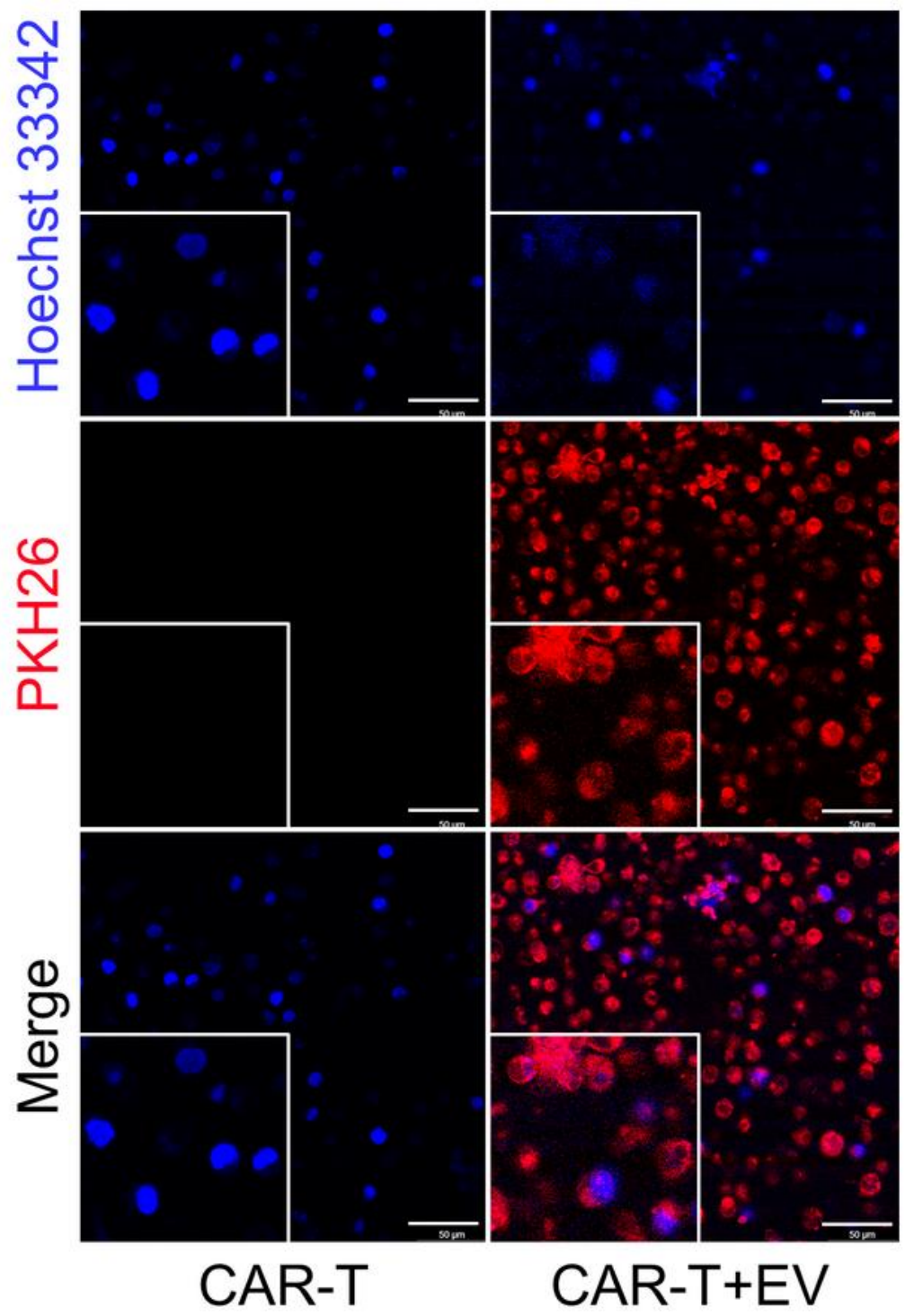

Revised Fig. 3a

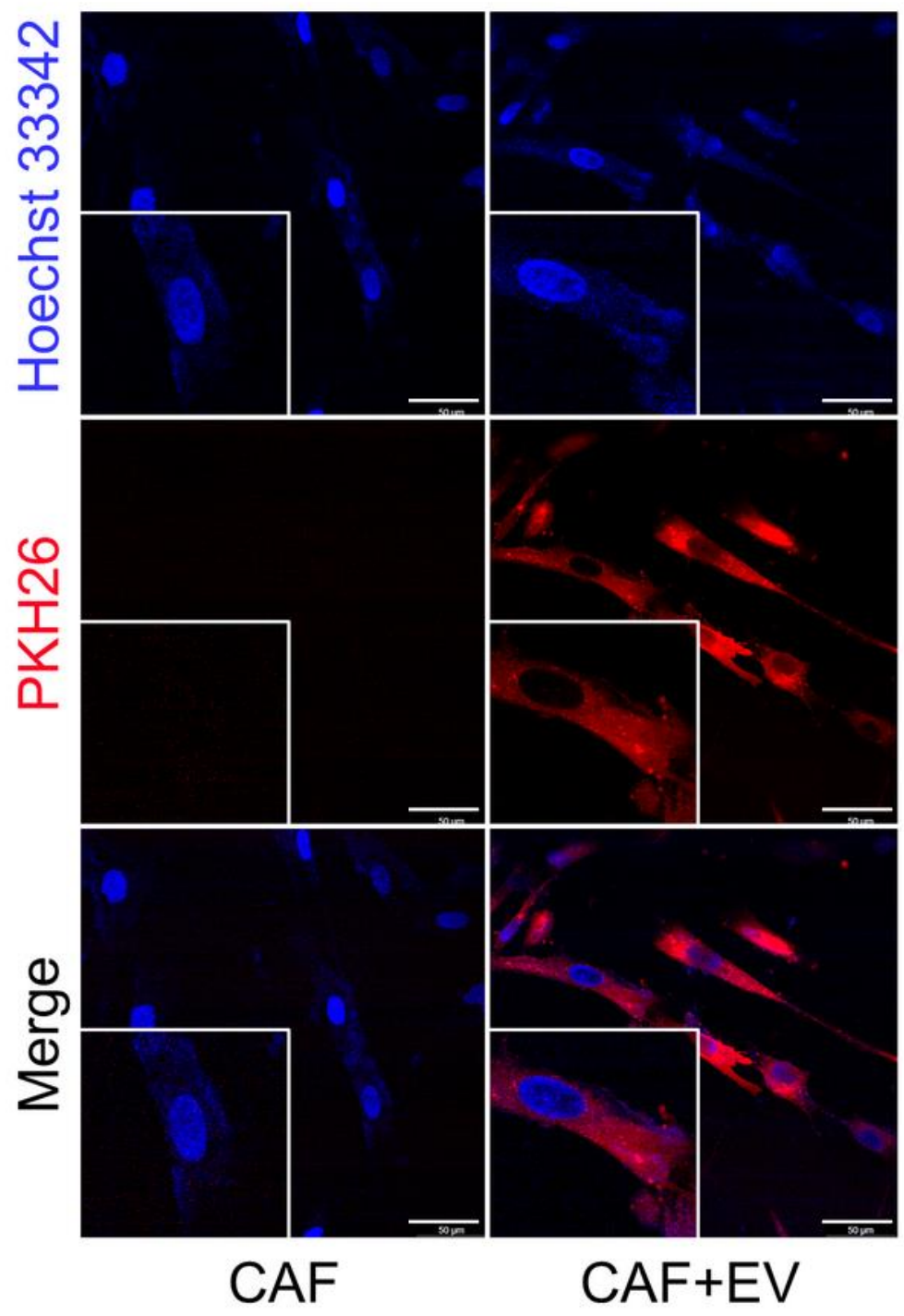

Revised Fig. 4a

- Regarding the RNAseq of CAR-T cells treated with CLDN18.2 exosomes. Instead of providing a heatmap in Figure 3b, please provide a barplots showing replicates and test for significance.

A. Thank you for your suggestion. As mentioned above, we have provided improved heatmaps and bar plots incorporating biological replicates and relevant statistical analysis (t-test) for your reference, which highlight the activation of CAR-T cells by EV-CLDN18.2 administration. However, due to the substantial variability in gene expression levels, bar plots are insufficient to outline inter-group diversities. Also, the limited space available for figures makes it difficult to integrate the bar plots effectively. Therefore, we have opted to retain only the improved heatmaps (left panel) in the **revised Fig. 3b**.

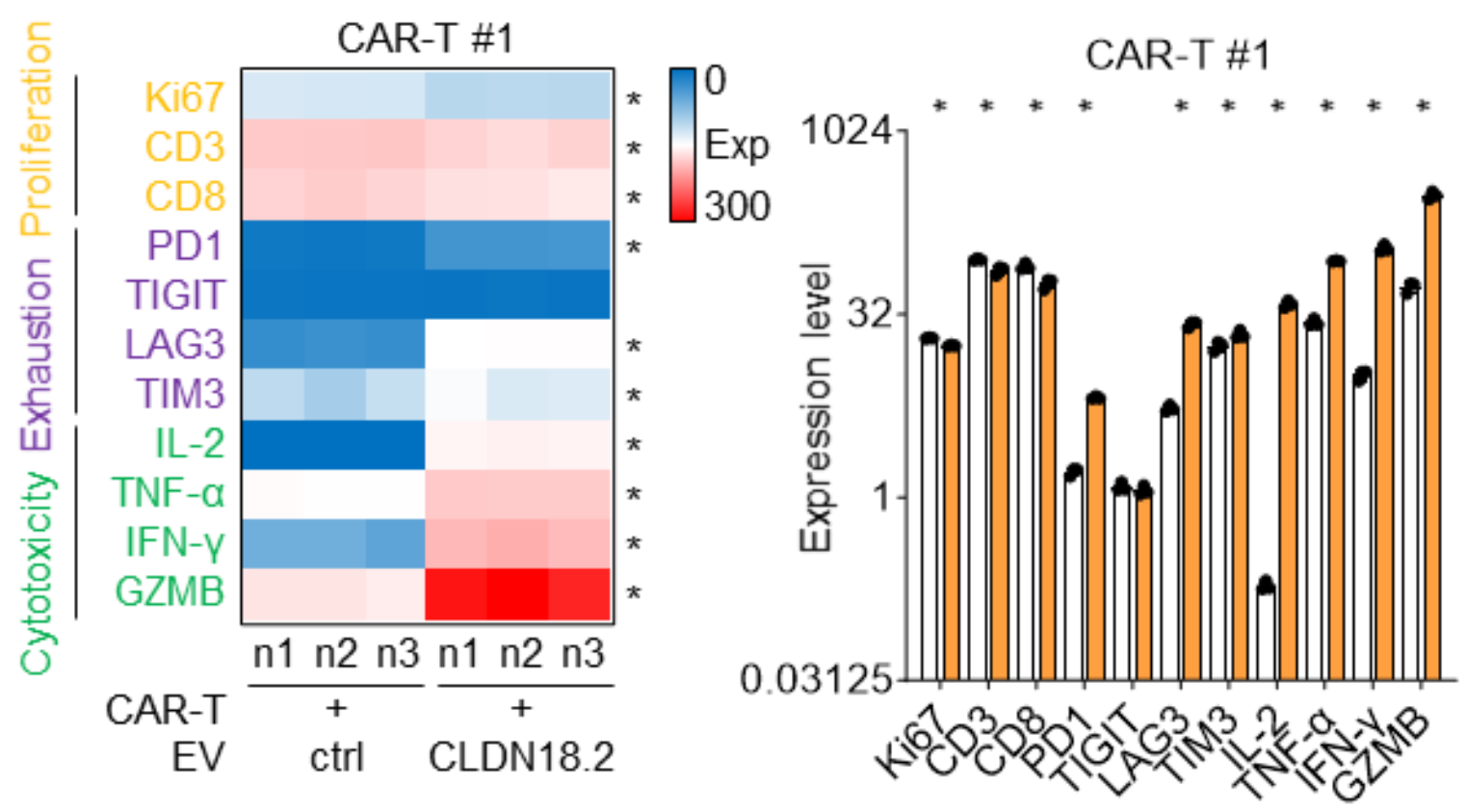

Revised Fig. 3b

- I would move all trajectory analyses to Extended Data Figures.

A. Thank you for the suggestion. These trajectory analyses illustrate EV-CLDN18.2-induced changes of single cell features. Considering the limited space in Extended Data Figure, we have chosen to retain the trajectory analyses in their current form.

- Please provide a clearer legend integrated in the Figure 5c-g, it is not immediately clear what the colors correspond to.

A. Thank you for the suggestion, we apologize for the ambiguous descriptions in this part. We have improved related figure legends in the revised manuscript.

Because of the large number of plots incorporated in this figure, we used different colors to mark different administrations for PDXs. Accordingly, these colors were used to link tumor growth curves, body weight curves, tumor weight bar plots, as well as bar plots for EV-CLDN18.2/CLDN18.2-CAR-T cell/cytokines.

REVIEWER COMMENTS

Reviewer #2 (Remarks to the Author):

As mentioned in the first round of revisions, the manuscript contains a large amount of data with significant findings and several innovative approaches are used. The authors demonstrated an association between levels of CLDN18.2 EVs and resistance to CAR-T therapy in patients. In addition, they show using plasma exchange that response to CAR-T and antibody can be restored in vivo and in patients. In contrast, the role of EVs in CAR-T cell dysfunction via EV antigen interaction with CAR is not novel. The role of EVs in blocking therapeutic antibodies has also been previously reported. The authors acknowledged the comments and made an effort to respond to all of them. However, it remains several shortcomings that need to be addressed before the study is suitable for publication.

Identification of CLDN18.2/PD-1/PD-L2 EVs as circulating biomarkers of response to CLDN18.2 CAR-T therapy:

I thank the authors to provide additional experiments for the EV array, a critical method in the manuscript. Despite prior reports using the EV array and additional explanations from the authors, there is a lack of analytical validation and compelling evidence of the reproducible quantitative analysis of proposed markers. In the rebuttal letter, the authors claim that the linear dynamic range indicates sufficient sensitivity and accuracy. Without limit of detection, sensitivity is not measured. Accuracy can only be measured with true reference value. The study does not mention if positive and negative controls were used. Furthermore, no standard deviation is provided which suggests that no technical replicates have been used. It is also critical to assess the precision of the technique. No negative and positive controls are mentioned which is an important limitation.

A. We are sincerely grateful for your acknowledgment of our efforts and for highlighting these critical points regarding the analytical validation of the EV-Array. We agree that rigorous characterization is essential, and we have performed a series of experiments to address these specific concerns, as detailed below. We have also improved the descriptions for the methodology of EV-array in the 2nd revised manuscript.

1. Assessment of sensitivity, dynamic range, and controls

- For sensitivity & dynamic range

The sensitivity of the EV-array assay is intrinsically linked to its working range. In order to address your concern, we mixed 5 plasma samples from GC patients, then determined the operational range of the EV-Array by testing the dilution series of mixed GC plasma samples (2/5/8/11/14/17/20 μL plasma, diluted into 100 μL volume with wash buffer (0.05% Tween20 in PBS)). After EV-array assessment, we selected three representative EV-proteins across a wide dynamic range of expression level (high for CD81, medium for CLDN18.2, and low for EpCAM). According to the standard curve, all three EV-proteins displayed strong linear correlations ($R^2>0.9$, **Fig for review only. 1**) between the input plasma

volume and the signal intensities, confirming that EV-array assay is capable of precisely quantifying variations in EV-protein levels within a calibrated sample range (2-20 µL). This established range ensures all subsequent measurements were performed under quantitatively reliable conditions. Therefore, we used 10 µL plasma per sample for subsequent EV-array assessment.

	Exp value by EV-array-Replicates					
Mixed plasma volume (µL)	CD81	CD81	CD81	mean	SD	CV
20	35871.33	32913.33	34860.33	34548.33	1503.479	0.043518
17	32107.67	31572.67	31050.67	31577	528.5133	0.016737
14	26222	28188	26763	27057.67	1015.584	0.037534
11	22481	20792	21635	21636	844.5004	0.039032
8	19241	18192	19419	18950.67	663.0251	0.034987
5	10356.67	11027.67	10632.67	10672.33	337.2541	0.031601
2	3225	4292	3416	3644.333	568.9678	0.156124
	Exp value by EV-array-Replicates					
Mixed plasma volume (µL)	CLDN18.2	CLDN18.2	CLDN18.2	mean	SD	CV
20	14901.33	15485.33	13792.33	14726.33	859.9599	0.058396
17	15804.67	15924.67	16421.67	16050.33	327.1335	0.020382
14	12898	12552	11248	12232.67	870.118	0.071131
11	7999	7972	6442	7471	891.2424	0.119294
8	5228	6951	5784	5987.667	879.3704	0.146864
5	3564.667	3062.667	3233.667	3287	255.2143	0.077644
2	1124	1159	1123	1135.333	20.50203	0.018058
	Exp value by EV-array-Replicates					
Mixed plasma volume (µL)	EpCAM	EpCAM	EpCAM	mean	SD	CV
20	3428.333	2603.333	4023.333	3351.667	713.0977	0.212759
17	3519.667	3064.667	2640.667	3075	439.5911	0.142956
14	3327	2831	2106	2754.667	614.0687	0.222919
11	2002	2369	2344	2238.333	205.052	0.091609
8	1313	1236	724	1091	320.1547	0.293451
5	1093.667	1048.667	1146.667	1096.333	49.05439	0.044744
2	616	500	678	598	90.35486	0.151095

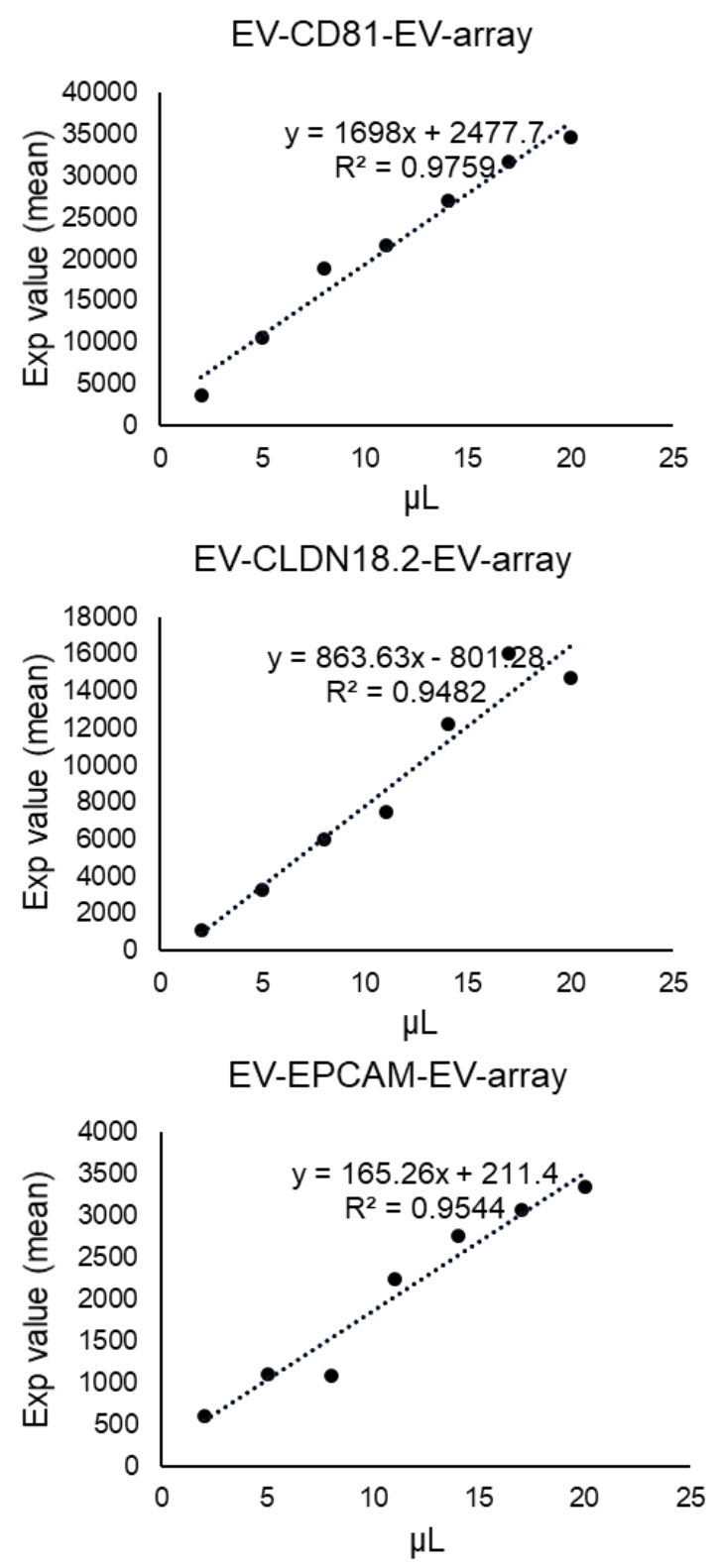

Fig for review only. 1

- For positive & negative controls

We apologize for the omission to describe EV-array's controls in both the original manuscript and the previous revision. Specific positive and negative controls were included to ensure the quality of EV-array assay. Biotinylated BSA (100 mg/mL) served as the positive control, while PBS containing 5% glycerol served as the negative control, both were systematically included on EV-array's chip for quality control purposes, as described below.

2. Evaluation of precision and reproducibility

- For the design & structure of EV-array

Structurally, the chip for EV-array contains 14 wells, which supports the assessment for 14 plasma samples at one time. Each well consists of 60 captures (3 replicates per capture), including 2 captures for positive control, 4 captures for negative control and 54 captures for EV-proteins' antibodies, to ensure the intra-assay accuracy. The fluorescent signal intensity of each replicate is recorded as its expression value. The average expression value of the 3 replicates in each capture is recorded as the expressional matrix of EV-array (**Fig for review only. 2**).

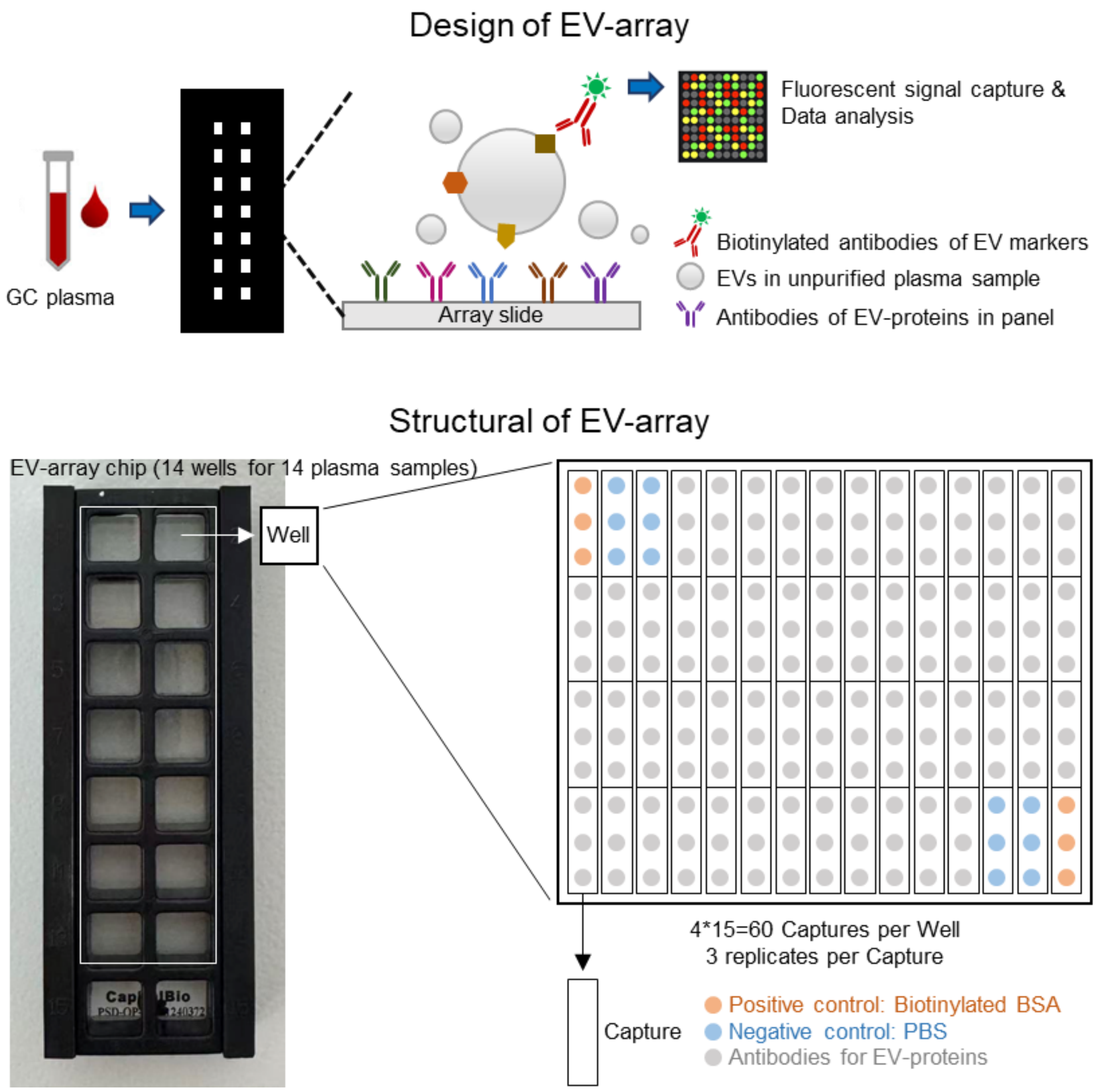

Fig for review only. 2

- For technical replicates & standard deviation

The consistency of these technical replicates was rigorously assessed by calculating the Coefficient of Variation (CV). For instance, the CV values for CD81/CLDN18.2/EpCAM replicates were 3.9%/11.9%/9.2% with ~10 μL plasma sample (shown in **Fig for review only. 1**), which is well below the commonly accepted threshold of ≤25% for immunoassay reproducibility, as referenced from Findlay et al (*Validation of immunoassays for bioanalysis: a pharmaceutical industry perspective. J Pharm Biomed Anal. 2000 Jan;21(6):1249-73*).

In order to ensure the precision of the technique, we evaluated the overall consistency of the platform by performing 5 independent reactions of EV-array (well#1-#5) in randomly selected wells on a single chip, each loaded with the identical GC plasma sample. Pearson correlation analysis demonstrated a very high degree of signal consistency across these wells (**Fig for review only. 3**), providing strong evidence for the robust reproducibility of the entire EV-array assay.

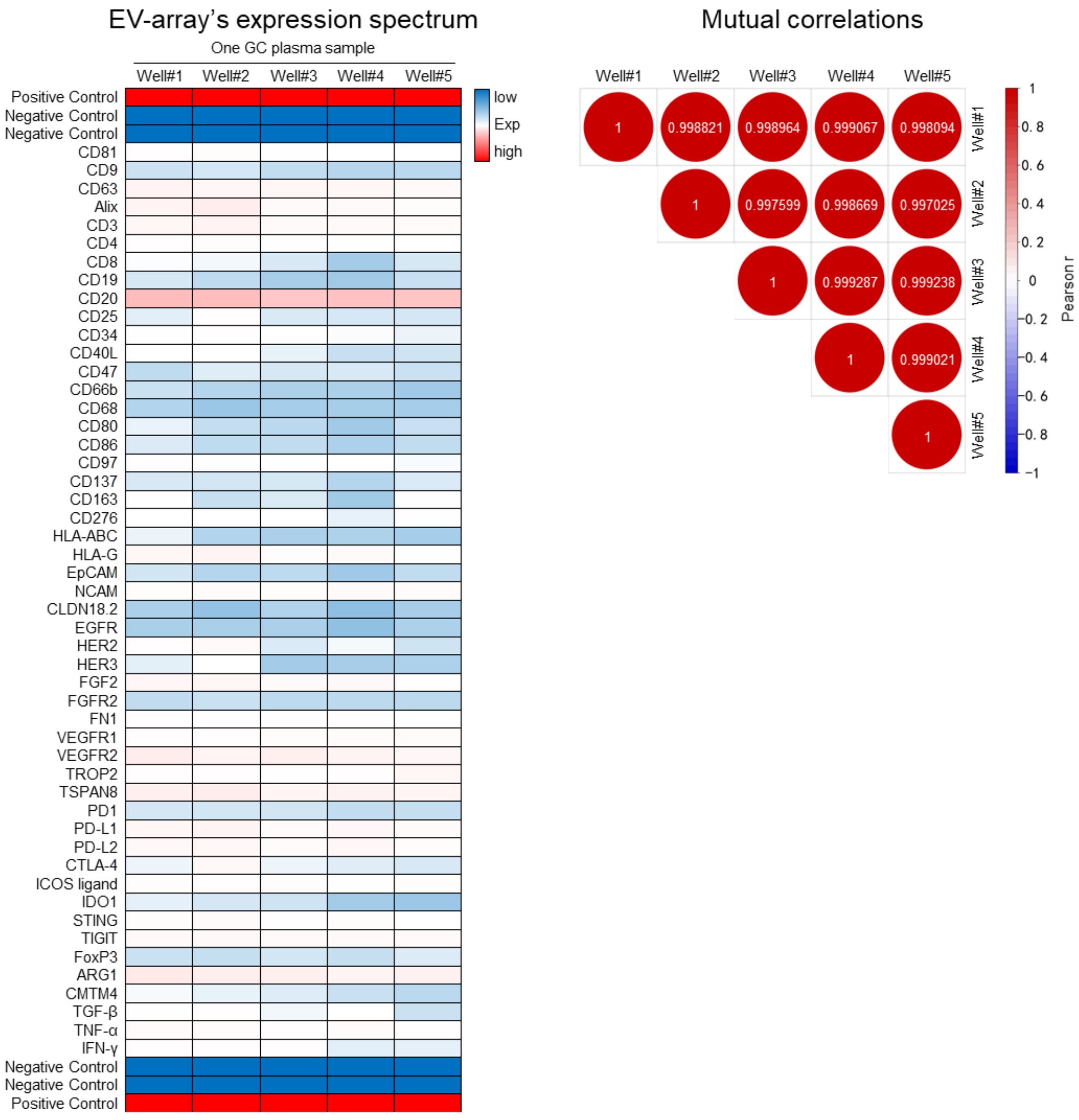

Fig for review only. 3

In summary, the data presented from the linearity, precision, and consistency analyses, combined with the inclusion of appropriate positive/negative controls, provide a comprehensive analytical validation of the EV-Array for the reproducible quantification of EV-proteins in our study.

The authors conducted an ELISA on isolated EVs to validate the data found with EV array. However, the agreement between both techniques is not acceptable suggesting a high variability for both analytical methods. Detailed methodology for the ELISA is required. Importantly, standard curve, limit of detection and limit of quantification are not provided for both ELISA and EV array. It is unclear if technical replicates were used. If yes, raw data of replicates should be provided. Without this information, it is difficult to conclude that the quantitative analysis of EV proteins is reliable and reproducible. Latter in

the rebuttal, the authors claim that this work was not designed to methodologically compare EV array with ELISA. Therefore, it is unclear why the authors present ELISA data? The data shown are not supporting the performance of the EV-array.

A. Thank you for the comment. We have revised the manuscript to incorporate the requested methodological details of EV-ELISA and have demonstrated additional characterization to address your concerns regarding EV-ELISA.

1. Detailed methodology for EV-ELISA

Plasma EVs for ELISA were isolated with a SEC-based purification kit (EV40T, EVbio, China). Information for the kit was provided below. Isolated plasma EVs were diluted to a final volume of 100 μL with coating buffer (0.1 M carbonate, pH=9.6) and incubated on ELISA plates overnight at 4°C. Following incubation, the plates were washed with PBST and blocked with 100 μL of 5% BSA for 60 minutes at room temperature. The samples were then incubated with primary antibodies for CLDN18.2 (ab241330, Abcam)/PD1 (ab52587, Abcam) for 2 hours at room temperature. After another round of PBST wash, the plates were incubated with a goat-anti-mouse IgG secondary antibody for 1 hour at room temperature. Subsequently, 100 μL of TMB substrate solution was added and allowed to react for 30 minutes. The reaction was stopped by adding 100 μL of stop solution, and the optical density (OD) was immediately measured at 450 nm.

2. Assessment of EV-ELISA's reliability

We mixed 5 plasma EV samples isolated from 5 GC patients, then determined the operational range of ELISA by testing the dilution series of mixed EVs (0.15625-10 μL isolated EVs, diluted into 100 μL volume with coating buffer). According to the standard curve, both EV-CLDN18.2 and EV-PD1 displayed strong linear correlations (R^2>0.9, **Fig for review only. 4**) between the input EV volume and the signal intensities, confirming that ELISA is capable of precisely quantifying variations in EV-protein levels within a calibrated sample range (0.15625-10 μL). This established range ensures all subsequent measurements were performed under quantitatively reliable conditions. Therefore, appropriate doses of EVs (2 μL for CLDN18.2 and 4 μL for PD1) were determined for subsequent ELISA assessment in multiple isolated plasma EV samples.

	Exp value by ELISA-Replicates					
Mixed plasma volume (μL)	CLDN18.2	CLDN18.2	CLDN18.2	mean	SD	CV
10	1.418	1.415	1.222	1.351667	0.112305	0.083086
5	0.513	0.453	0.521	0.495667	0.037166	0.074982
2.5	0.338	0.314	0.326	0.326	0.012	0.03681
1.25	0.192	0.18	0.176	0.182667	0.008327	0.045584
0.625	0.122	0.11	0.131	0.121	0.010536	0.087072
0.3125	0.086	0.082	0.086	0.084667	0.002309	0.027276
0.15625	0.075	0.069	0.072	0.072	0.003	0.041667

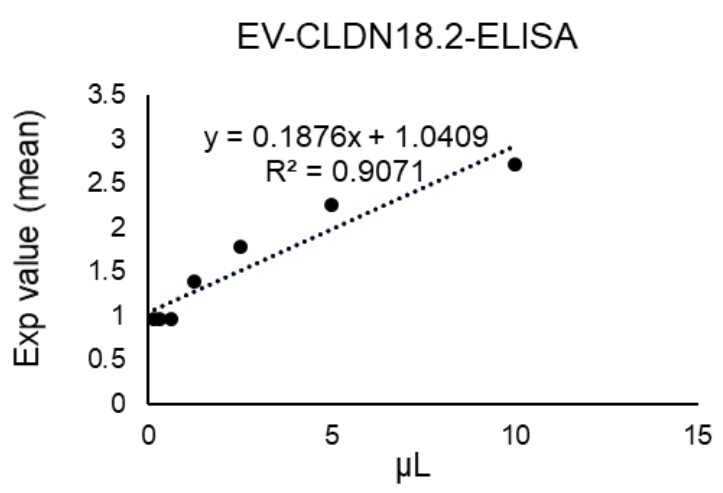

	Exp value by ELISA-Replicates					
Mixed plasma volume (μL)	PD1	PD1	PD1	mean	SD	CV
10	3.027	3.165	3.048	3.08	0.074357	0.024142
7.5	2.682	2.787	2.672	2.713667	0.063705	0.023476
5	2.271	2.46	2.056	2.262333	0.202139	0.08935
3	1.722	1.857	1.755	1.778	0.070378	0.039582
2	1.268	1.428	1.45	1.382	0.099338	0.07188
1	0.582	0.951	1.34	0.957667	0.379044	0.395799

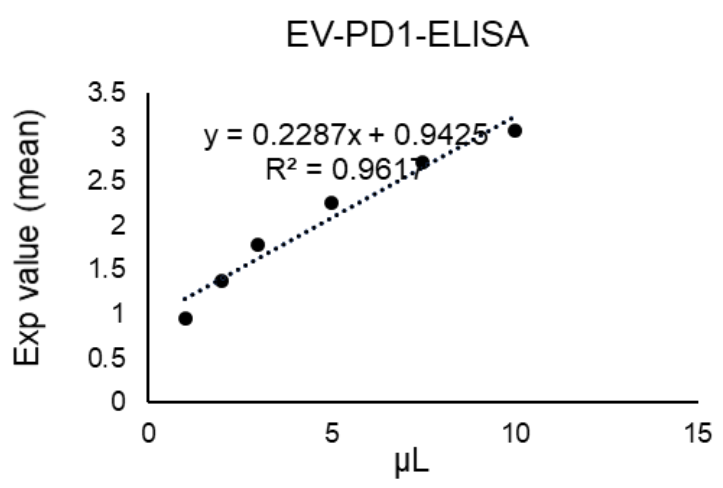

Fig for review only. 4

3. Raw data of replicates

In the 1st revision, we have compared the expressional correlations of ELISA and EV-array for EV-CLDN18.2 and EV-PD1. The raw data for replicates of ELISA for EV-CLDN18.2 and EV-PD1 are provided here in **Fig for review only. 5.**

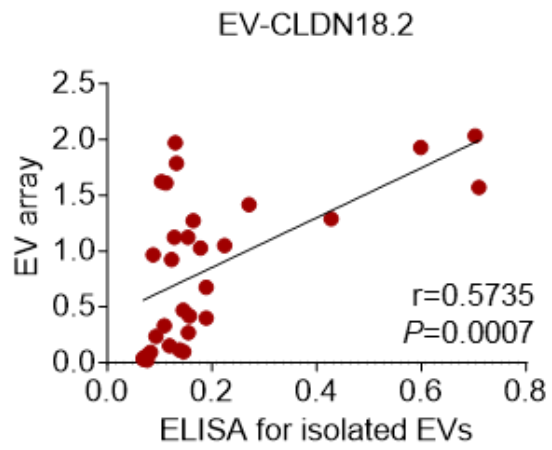

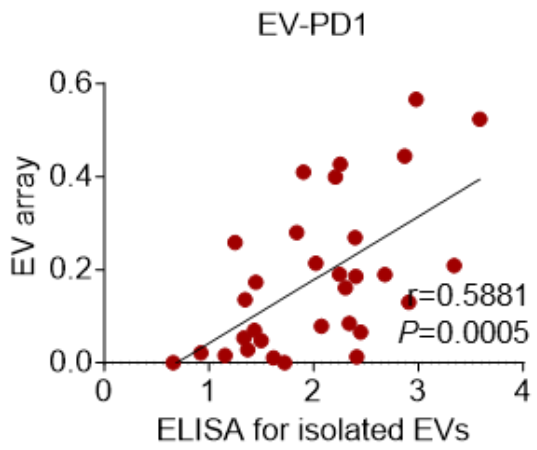

ELISA-CLDN18.2				
Plasma ID-CLDN18.2	Replicate 1	Replicate 2	Replicate 3	**Mean**
Plasma 1	0.099	0.087	0.094	**0.093**
Plasma 2	0.176	0.138	0.149	**0.154**
Plasma 3	0.084	0.072	0.074	**0.077**
Plasma 4	0.075	0.066	0.065	**0.069**
Plasma 5	0.166	0.135	0.135	**0.145**
Plasma 6	0.136	0.170	0.131	**0.146**
Plasma 7	0.080	0.081	0.088	**0.083**
Plasma 8	0.119	0.116	0.121	**0.119**
Plasma 9	0.137	0.106	0.092	**0.112**
Plasma 10	0.266	0.310	0.237	**0.271**
Plasma 11	0.160	0.165	0.168	**0.164**
Plasma 12	0.150	0.160	0.154	**0.155**
Plasma 13	0.136	0.139	0.140	**0.138**
Plasma 14	0.106	0.094	0.111	**0.104**
Plasma 15	0.686	0.751	0.692	**0.71**
Plasma 16	0.257	0.227	0.187	**0.224**
Plasma 17	0.124	0.134	0.131	**0.130**
Plasma 18	0.069	0.070	0.067	**0.069**
Plasma 19	0.448	0.435	0.402	**0.428**
Plasma 20	0.724	0.732	0.653	**0.703**
Plasma 21	0.115	0.128	0.125	**0.123**
Plasma 22	0.111	0.111	0.106	**0.109**
Plasma 23	0.185	0.188	0.195	**0.189**
Plasma 24	0.126	0.119	0.140	**0.128**
Plasma 25	0.192	0.186	0.190	**0.189**
Plasma 26	0.054	0.173	0.245	**0.157**
Plasma 27	0.172	0.172	0.189	**0.178**
Plasma 28	0.641	0.579	0.576	**0.599**
Plasma 29	0.131	0.129	0.135	**0.132**
Plasma 30	0.091	0.089	0.085	**0.088**
Plasma 31	0.075	0.076	0.077	**0.076**

ELISA-PD1				
Plasma ID-PD1	Replicate 1	Replicate 2	Replicate 3	**Mean**
Plasma 1	1.306	1.42	1.28	**1.335333**
Plasma 2	1.504	1.391	1.141	**1.345333**
Plasma 3	3.595	3.597	3.57	**3.587333**
Plasma 4	1.11	0.855	1.496	**1.153667**
Plasma 5	2.52	2.428	2.075	**2.341**
Plasma 6	1.714	1.386	1.748	**1.616**
Plasma 7	1.812	1.355	1.176	**1.447667**
Plasma 8	2.264	2.289	2.19	**2.247667**
Plasma 9	0.857	0.945	0.968	**0.923333**
Plasma 10	3.379	3.226	3.422	**3.342333**
Plasma 11	1.605	1.525	1.367	**1.499**
Plasma 12	1.983	1.965	2.114	**2.020667**
Plasma 13	3.077	3.119	2.739	**2.978333**
Plasma 14	2.313	1.346	2.198	**1.952333**
Plasma 15	2.402	2.595	2.191	**2.396**
Plasma 16	1.571	1.756	1.843	**1.723333**
Plasma 17	2.242	2.256	2.122	**2.206667**
Plasma 18	2.99	2.669	2.382	**2.680333**
Plasma 19	0.68	0.653	0.642	**0.658333**
Plasma 20	2.91	2.994	2.829	**2.911**
Plasma 21	2.322	1.941	1.962	**2.075**
Plasma 22	2.885	2.934	2.788	**2.869**
Plasma 23	2.278	1.498	1.936	**1.904**
Plasma 24	2.351	1.287	1.583	**1.740333**
Plasma 25	1.416	1.09	1.246	**1.250667**
Plasma 26	2.404	2.236	2.271	**2.303667**
Plasma 27	2.641	2.392	2.32	**2.451**
Plasma 28	2.463	2.265	2.506	**2.411333**
Plasma 29	2.602	2.465	2.146	**2.404333**
Plasma 30	2.047	1.804	1.667	**1.839333**
Plasma 31	1.585	1.163	1.365	**1.371**

Fig for review only. 5

4. The reason to present ELISA data

In response to your question regarding EV-array versus EV-ELISA, our primary objective was to identify a direct and widely accepted approach to validate that the EV-array reliably captures a broad dynamic range of expression levels for specific EV-associated proteins. Accordingly, we used ELISA as a benchmark method for the validity of EV-array, and applied Pearson correlation analysis to demonstrate that the distributions of low-to-high EV-CLDN18.2/EV-PD-1 expression were highly consistent between EV-ELISA and EV-array measurements. Given that EV-array and ELISA rely on distinct detection principles, it is reasonable that the two methods exhibit similar overall trends while retaining certain quantitative differences.

We acknowledge that the concordance between EV-array and EV-ELISA results is not optimal; however, it is sufficient to prove that EV-array detects a broad range of expression for EV-proteins. Given that all the result-oriented assessments and analyses were performed using the EV-array and are supported by multiple lines of clinical & experimental evidences, the inclusion of EV-ELISA validation does not change the conclusions or overall interpretation of the study.

We deeply appreciate your comment that our description about EV-array and ELISA may be misleading. Thus, we have improved the descriptions in the manuscript (for Fig. 1d, Extended Data Fig. 2k) to "ELISA validation for CLDN18.2/PD1 expression in EVs isolated from 31 random plasma samples in discovery cohort confirmed that EV expression array reliably captures a broad dynamic range of expression levels for specific EV-proteins".

In their revisions, the authors add they use SEC to purify EVs from plasma but no purity evaluation is provided and important methodological details are missing.The SEC purification kit provided by EVbio does not seem to be commercialized worldwide. Methodological details on the SEC purification kit, fractions collected with EV and protein concentrations measurements should be provided. Assessment of EV purity is also required per MISEV 2023 guidelines.

A. Thank you for these important comments. We have revised the manuscript to incorporate the requested methodological details of EV isolation and have demonstrated additional characterization to address your concerns regarding EV purity.

1. Detailed methodology for EV isolation

Plasma EVs were isolated using a SEC purification kit (EVTMC, EVbio, China), which is commercially available in China and employs a two-step purification process:

- Step 1 (Size Exclusion): 100 μL of plasma was loaded onto a column pre-packed with Sepharose CL-6B resin (Cytiva). After the flow-through was discarded by gravity, the EV-enriched fraction was eluted with 200 μL of PBS (Fraction 1).
- Step 2 (Mixed-Mode Chromatography): The eluate (Fraction 1) was then applied to a second column containing a proprietary mixture of Fractogel EMD SO3-(M) (MilliporeSigma) and Capto Core 700 (Cytiva) resins. Following a brief incubation, the purified EVs were collected in the flow-through by centrifugation at 500 × g for 30 seconds (Final EV Fraction).

While the specific kit is not commercialized worldwide, its core components consist of globally available, well-characterized chromatography resins, ensuring the transparency and reproducibility of the protocol.

2. Assessment of purity for EVs isolated by the SEC purification kit

In accordance with the MISEV 2023 guidelines, we re-performed the following characterizations for plasma EVs isolated using the SEC purification kit (**Fig for review only. 6**).

- Concentration & Size: Nanoparticle Tracking Analysis (NTA) confirmed that isolated EVs have a high particle concentration and appropriate particle size distribution of EV.

- Morphology: Visualization by Transmission Electron Microscopy (TEM) confirmed that isolated EVs have a typical EV morphology (cup-shaped structures).
- Protein expression: Mass-spectrum (MS) quantification for EV-proteins confirmed that isolated EVs have high expression of typical EV markers (such as CD81, CD9, and TSG101).

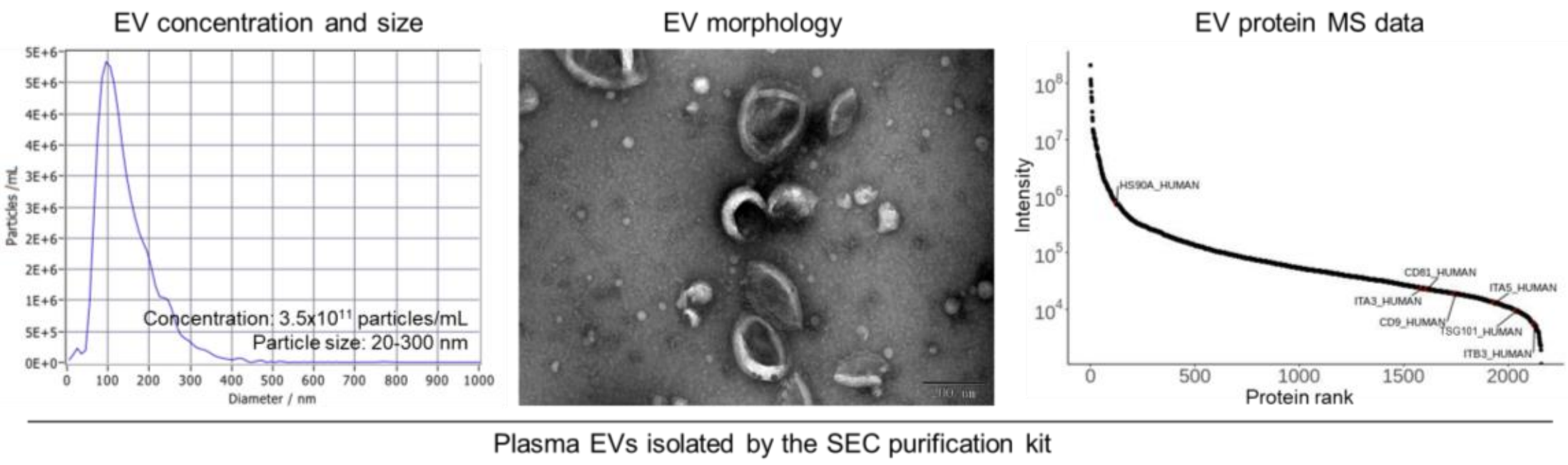

Fig for review only. 6

These NTA, TEM, and protein expression data—consistent with established biomarker studies—confirm the successful isolation of EVs suitable for downstream analyses.

I appreciate the authors' response on their hypothesis they can detect intracellular proteins on surface of EVs as part of the "corona". They cite two landmark studies on the existence of protein corona in EVs and present TEM results from a previous study. However, it remains uncertain that the authors can quantify with accuracy expression of intracellular proteins on surface of EVs. Additional experiments are needed to support their hypothesis.

A. Thank you for the important question. We agree with your insightful comments that detecting the accurate expression of intracellular proteins on surface of EVs is an important issue. As demonstrated in **Fig for review only. 2**, the EV-array assay is inherently designed to detect surface-accessible proteins carried by EVs. The platform utilizes a sandwich immunoassay principle: EVs are first captured by antibodies immobilized on the array surface, then administrated with biotinylated antibodies of CD81/CD9/CD63, the canonical transmembrane markers of EVs. Ideally, this two-step process ensures that only intact EVs are captured & measured, and the fluorescent signal is contingent on the presence of these surface-exposed proteins. We have improved related descriptions in Methods section.

Indeed, most TIME-related proteins we adopted in EV-array's panel are predominantly expressed on cell surface, which endows them a better chance to be released as EV-bounded cargos to mediate crosstalk between cells and therapeutic agents in TIME. However, based on our limited knowledge, there is currently no evidence to support the notion that the subcellular localization of a protein necessarily precludes its presentation on the surface of extracellular vesicles upon EV release. In the complex microenvironment of cancer patients, these intracellular proteins could be presented on surface of EVs by protein corona or other unknown mechanisms, and become detectable by EV-array.

Importantly, we directly addressed your concerns of surface localization through a controlled lysis experiment. We applied 2 plasma samples of GC patients (#A-#B) for EV isolation and subsequent

analysis. When isolated plasma-EVs were subjected to lysis using RIPA buffer and sonication—a treatment that disrupts the lipid bilayer of EVs, the concentrations of EV particles detected by NTA were reduced from 3.3×10^{10} particles/mL to 1.2×10^{10} particles/mL, while the majority of protein expressions detected by EV-array were also evidently diminished (**Fig for review only. 7**). These results provide direct experimental evidence that EV-array's detection of EV-proteins depends on the structural integrity of extracellular vesicles. This assay detects protein epitopes exposed on the EVs surface, irrespective of whether they are integral membrane proteins or presented by protein corona/other unknown mechanisms. Therefore, although the precise origin of some signals may be complex, our data demonstrate that the EV-array specifically and reliably detects surface-accessible proteins on EVs.

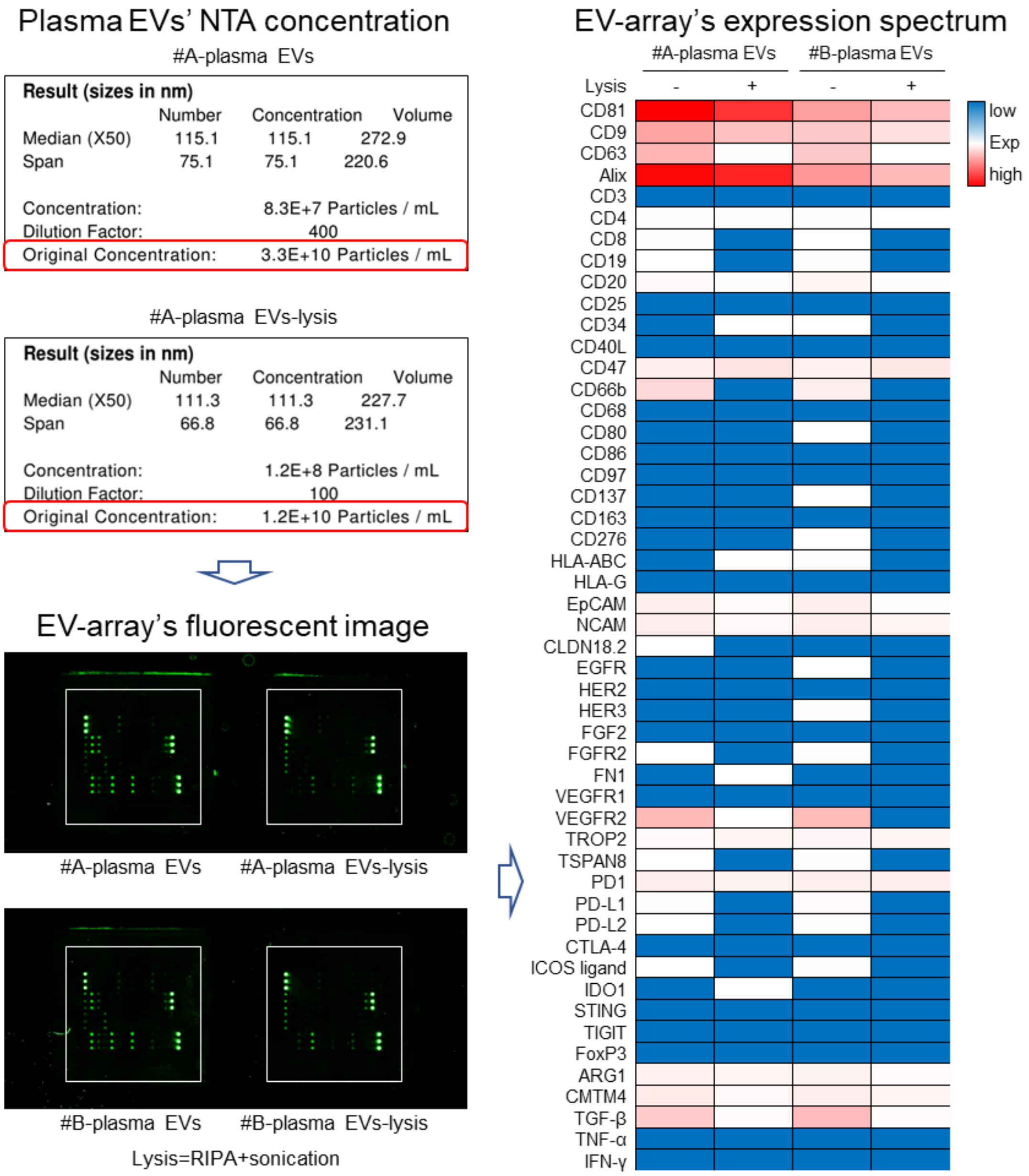

Fig for review only. 7

Moreover, we wish to clarify that our manuscript is not designed as a methodological instruction for EV-array or other assessment techniques. The goal of our work is to explore what and why specific proteins,

in form of EVs, influence CLDN18.2-targeted CAR-T and antibody therapies, then propose therapeutic managements to improve therapeutic efficacy. As we observed, all three proteins (CLDN18.2, PD1 and PD-L2) we screened to be involved with therapeutic efficacy are predominantly expressed or exposed on cell surface. Through performing comprehensive studies, we have elucidated their functions & mechanisms in modulating TIME and the efficacy of CLDN18.2-CAR-T/antibody therapies. Since all non-surface bound or intracellular proteins displayed relatively low correlation with therapeutic efficacy, whether they can be expressed on EVs' surface or whether they can be detected by EV-array have no influence on the conclusions or overall interpretation of this study.

The authors looked at the association of EV proteins with response to CLDN18.2 CAR-T therapy using a discovery cohort (N=31 patients with baseline samples) and validation cohort (N=46 patients with baseline samples). Based on the figure 1, the discovery cohort includes 77 patients. In Extended Figure 3, the discovery cohort has 31 patients. In Extended Figure 4, the validation cohort has 46 patients. It seems that the authors combine both cohorts in Fig.1. This discrepancy needs to be addressed by the authors.

A. Thank you for the comment. As you mentioned, we used a discovery cohort (31 patients) and a validating cohort (46 patients) to explore the association of EV proteins with response to CLDN18.2-CAR-T therapy.

- For discovery cohort, Supplementary Fig. S1 described its absolute expression spectrum. Extended Fig. 2a-2f described its relative expression spectrum. Fig. 1e-1g and Supplementary Fig. S2 described its screening. Extended Fig. 3 described the correlations of key EV-proteins and CPP-score with therapeutic efficacy in discovery cohort.
- For validating cohort, Supplementary Fig. S3 described its absolute expression spectrum, Supplementary Fig. S4 described its relative expression spectrum, Supplementary Fig. S5 described its screening. Extended Fig. 4 described the correlations of key EV-proteins and CPP-score with therapeutic efficacy in validating cohort.

As described in the Results section, key EV-proteins and related CPP-score consistently reflected the therapeutic efficacy of CLDN18.2-targeted CAR-T therapy in both the discovery and validating cohorts. Given the limited space of main figures, we combined the therapeutic efficacy data from both cohorts into Fig. 1h-1o to more effectively illustrate the clinical relevance of the key EV-proteins and CPP-score. Corresponding descriptions have been revised and clarified in the 2nd revision.

In the table S7, EV-PD1 does not show strong association with outcome (p=0.453 for PFS and p=0.524 for OS). Opposite effects are observed between discovery and validation cohorts. Surprisingly, high EV-PD1 expression is associated with shorter PFS and OS in both the discovery and validation cohorts (Ext Fig.3 and 4) but COX analysis does not show association of EV-PD1 with outcome in the validation

cohort. Based on this, it is unclear how the EV-PD1 contributes to the CPP-score and how CPP-score shows similar association with outcome for both discovery and validation cohorts.

A. Thank you for the question. As stated in the manuscript, Supplementary Table S7 demonstrated the COX multivariate analysis for the involvement of CLDN18.2 H-score, EV-CLDN18.2, EV-PD1 and EV-PD-L2 with CLDN18.2-CAR-T's PFS and OS, in both discovery and validating cohorts. Instead of being individually compared by univariate analysis, all 4 parameters (CLDN18.2 H-score, EV-CLDN18.2, EV-PD1 and EV-PD-L2) were simultaneously used by the multivariate analysis. Therefore, the relatively large *P* values for EV-PD1 in the validating cohorts should be interpreted as EV-CLDN18.2 and EV-PD-L2 being more prognostic than EV-PD1, rather than EV-PD1 being non-prognostic. More importantly, all three EV-proteins are stronger prognostic markers than CLDN18.2-H-score. We have provided clearer descriptions in the 2nd revised manuscript.

Here we are demonstrating the COX univariate analysis for the involvement of EV-PD1 with CLDN18.2-CAR-T's PFS and OS in both discovery and validating cohorts, to address your concern (**Table for review only. 1**). In both discovery and validating cohorts, EV-PD1's result of univariate COX displayed the same unfavorable prognostic trend of correlation with PFS/OS as indicated by Kaplan-Meier survival analysis. Therefore, EV-PD1 appropriately contributes to the therapeutic indications of CPP-score.

	**Discovery cohort**				**Validating cohort**			
	**PFS**		**OS**		**PFS**		**OS**	
**Variates**	***P* value**	**Hazard Ratio (95% CI)**	***P* value**	**Hazard Ratio (95% CI)**	***P* value**	**Hazard Ratio (95% CI)**	***P* value**	**Hazard Ratio (95% CI)**
EV-PD1	0.063	7.814 (0.895-68.205)	0.0473	10.152 (1.028-100.280)	0.049	6.789 (1.007-45.777)	0.047	10.152 (1.028-100.28)

Table for review only. 1

Part 2: CLDN18.2 EVs interacts with CAR and induces CAR-T dysfunction:

PKH26 is recognized to form aggregates in solution and false positives in EV uptake experiments. To address my initial comments, the authors use SEC to purify PKH26-labeled EVs post-SEC. They provide revised figures but they miss quantification of PKH26-positive cells and the distance indicated by the scale bar are missing. In the revised Ext Fig 7f, no PKH26 signal was found in PBS control which is odd considering PKH26 form aggregates in PBS. Based on authors' comments, Fig.3a, 4a have been revised using purified samples with SEC but they are the same picture as the original submission.

In Fig.3a, there is much more PKH26-positive structures of similar size to CAR-T cells are present outside CAR-T cells. PKH26-positive EVs do not show discrete signals in CAR-T cells but the entire cytosol is labeled suggesting indirect PKH26 staining of CAR-T cells. In addition, it looks like 100% of

cells are positive for PKH26 which is unusual as prior reports show that the uptake of EVs by T cells is very low.

A. Thank you for the question. PKH26 labeling is a widely used method to study EVs' interaction with cells. As shown below, multiple reports have pointed out that after administrating cells with PKH26-labeled EVs, the fluorescent imaging of PKH26 resembles cells' uptake of EVs.

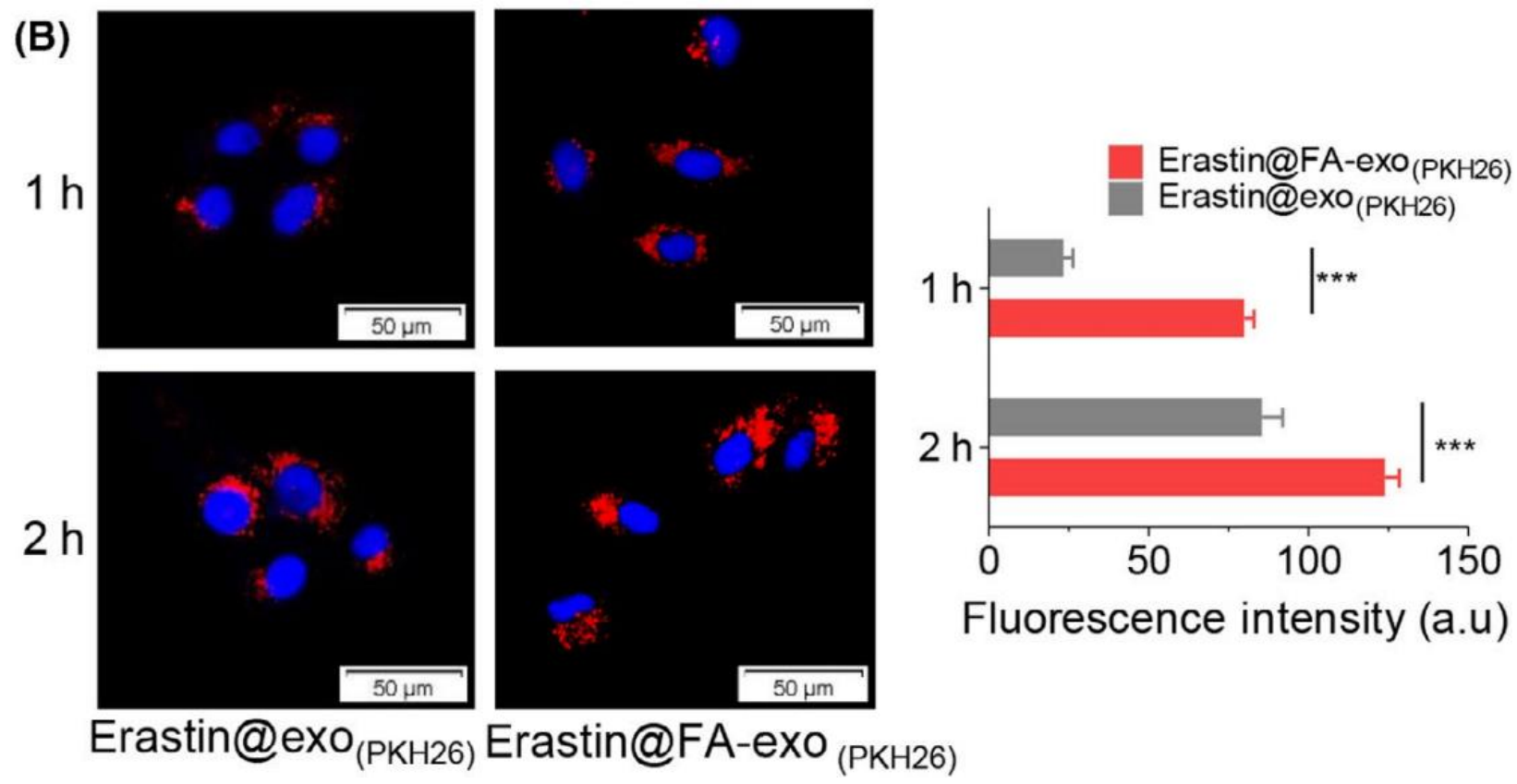

Cited Fig. 3B displaying that PKH26-labeled EVs internalized by MDA-MB-231 cells (Targeted exosome-encapsulated erastin induced ferroptosis in triple negative breast cancer cells, Cancer Sci. 2019 Oct;110(10):3173-3182).

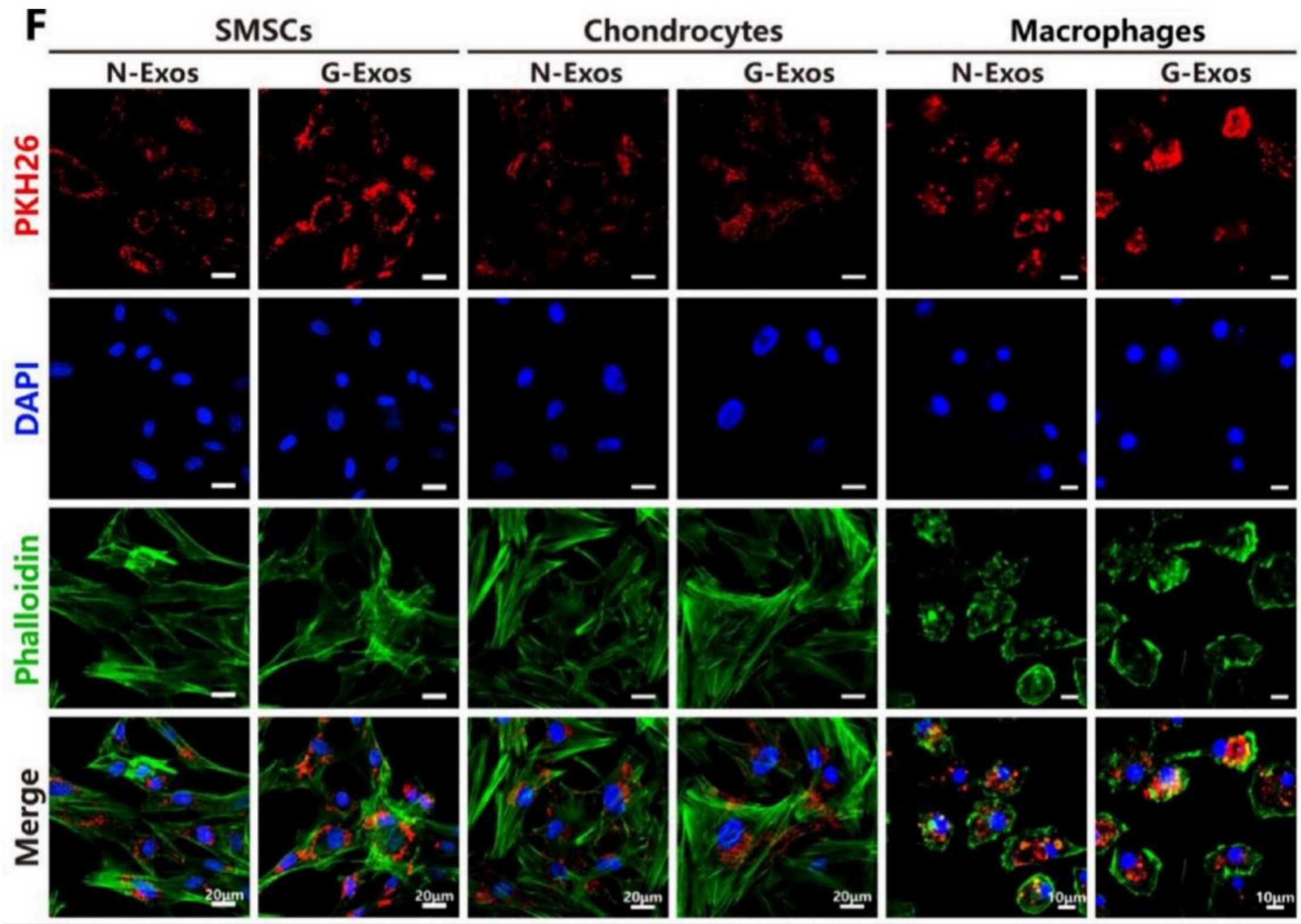

Cited Fig. 2F displaying that PKH26-labeled EVs internalized by SMSCs, chondrocytes, and macrophages (Three-Dimensional Bioprinting of Growth Differentiation Factor 5-Preconditioned Mesenchymal Stem Cell-Derived Exosomes Facilitates Articular Cartilage Endogenous Regeneration, ACS Nano. 2025 Apr 29;19(16):15281-15301).

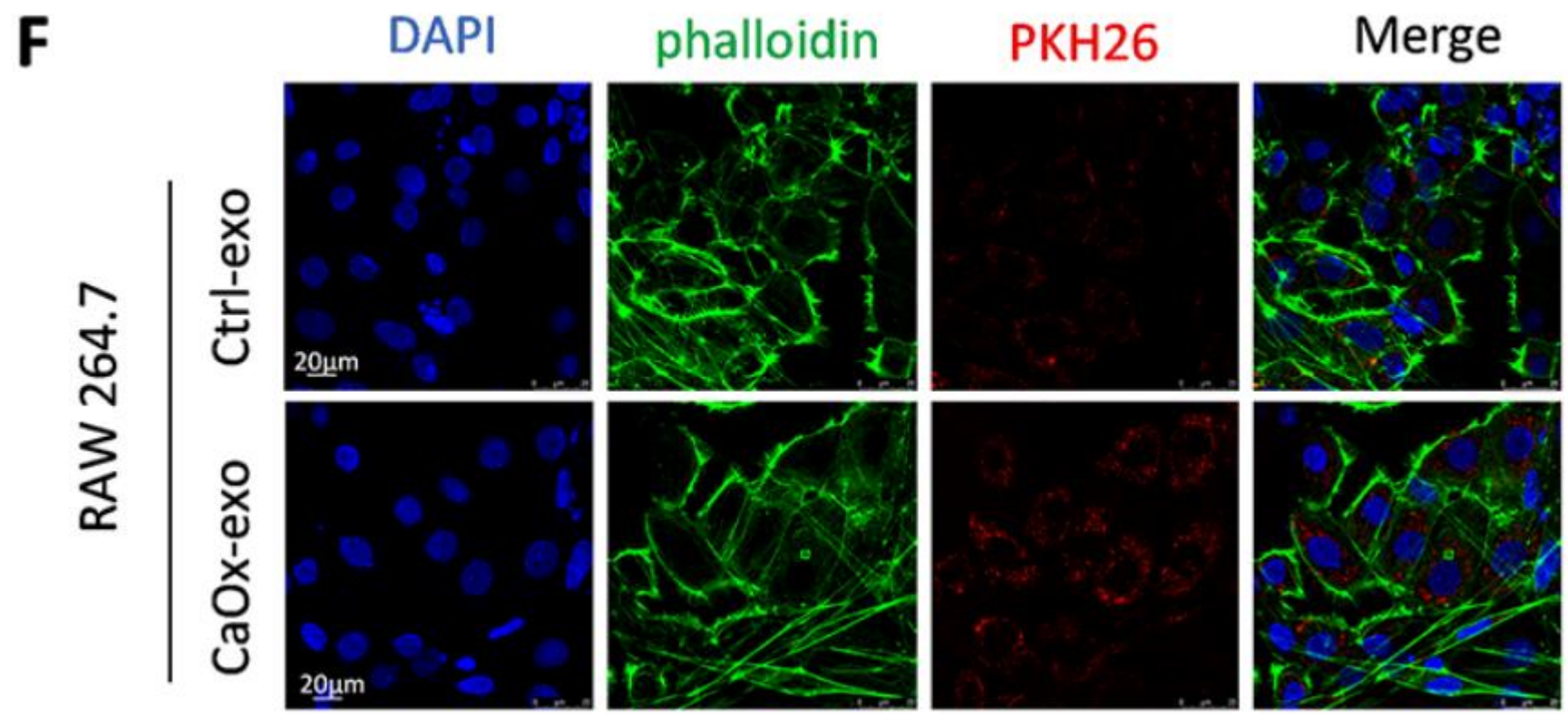

Cited Fig. 2F displaying that PKH26-labeled EVs taken up by RAW264.7 cells (CREB1/CRTC2 regulated tubular epithelial-derived exosomal miR-93-3p promotes kidney injury induced by calcium oxalate via activating M1 polarization and macrophage extracellular trap formation. J Nanobiotechnology. 2025 Mar 12;23(1):204)

We apologize for any misunderstandings caused by our previous submission & revision. We have re-performed all fluorescent experiments in this revision to address your concern.

1. For the question about PKH26's aggregates and scale bar

It has been implicated by the manufacturer (Solarbio) that PKH26 may form aggregates in low-protein medium. For the staining of PKH26 in our last revision, before added into PBS or isolated EVs for staining, PKH26 dye was first diluted into 100 μg/mL working solution with Diluent C (provided by the manufacturer) following the manufacturer's instructions, which may reduce the formation of aggregates. We did observe a minimal amount of PKH26 aggregates when adding diluted PKH26 (with Diluent C) into PBS (group name: PBS+diluted PKH26), as shown in the **2nd revised Extended Fig. 7F**, which was negligible compared with EV+PKH26 (group name: EV (PKH26-stained)). We have also provided the distance indicated by the scale bar, which was omitted in last revision.

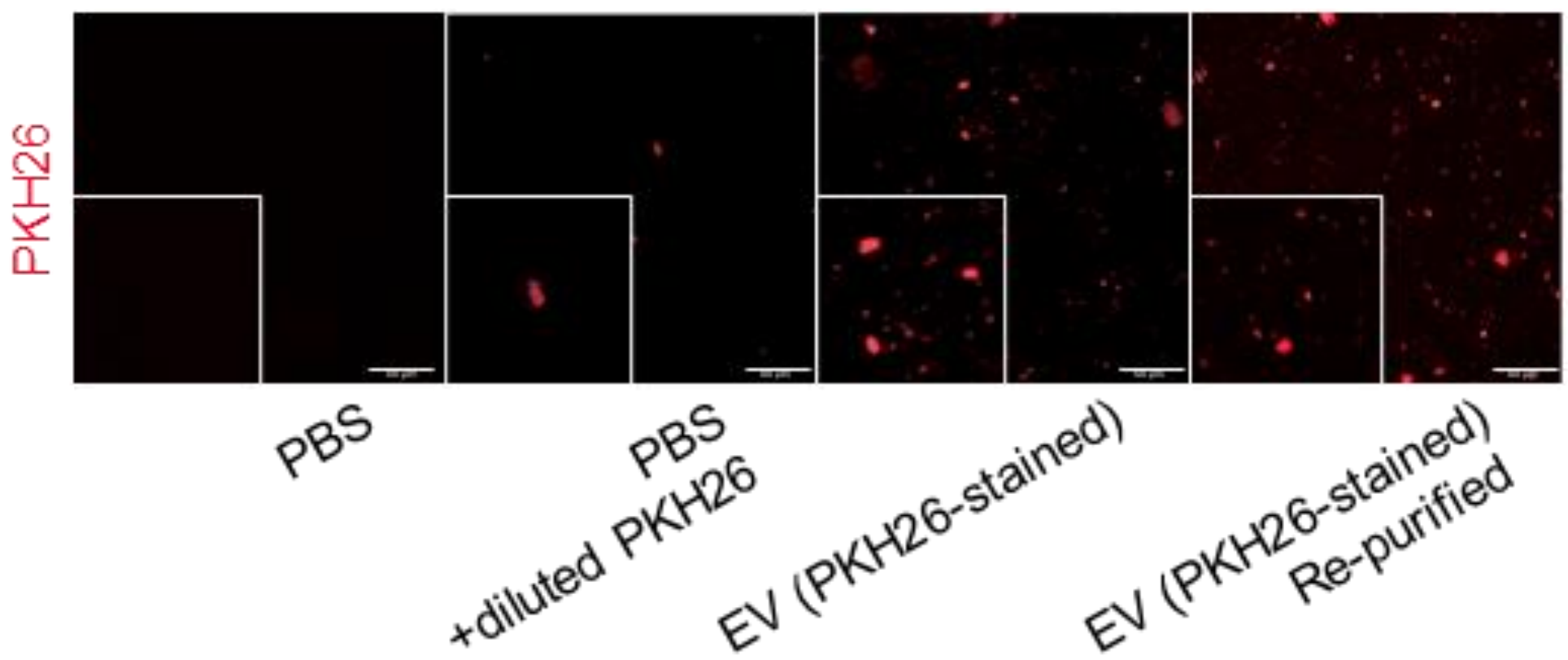

2nd revised Extended Fig. 7F

2. For Fig.3a, 4a and quantification

For previous Fig. 3a/4a, in our initial submission, these images were generated by administrating SEC-purified EV samples (pre-stained with PKH26) into CAR-T cells/CAFs, then staining cells with Hoechst 33342. We have provided more detailed declaration of methods in this revision.

Indeed, previous Fig. 3a displayed more PKH26-positive structures than Hoechst-positive structures in the CAR-T group, we infer it was due to that Hoechst-33342 only stains alive cells while PKH26 stains both dead/alive cells due to its lipophilicity. Because CAR-T cells are fragile and suspension cells, they possess a higher death rate during the administration with PKH26-labeled EVs and are hard to be removed when they are dead. Therefore, it is reasonable that previous Fig. 3a displaying more PKH26-positive structures than Hoechst-positive structures in the CAR-T group. We have provided **2nd revised Fig. 3a/4a** with higher quality, which suggest the same conclusion that CAR-T/CAFs can uptake EVs. Given the same principle, we would expect to observe a higher count of PKH26-stained CAR-T cells compared to their Hoechst-stained counterparts, but a comparable or lower count of PKH26-stained CAFs relative to Hoechst-stained CAFs. Additionally, there are certain phenotypic disparities between CAR-T cells and CAFs (e.g., suspension vs adherent, small vs large). These factors collectively compromise the validity of quantifying PKH26-positive cells.

Instead, we directly stained CAR-T and CAF with PKH26 in **2nd revised Fig. 3a/4a** for your reference. Cells directly stained with PKH26 displayed a clear red shade, which is comparable to unstained cells administrated with EVs pre-stained with PKH26. This new evidence demonstrates the direct uptake of EVs by CAR-T cells or CAFs.

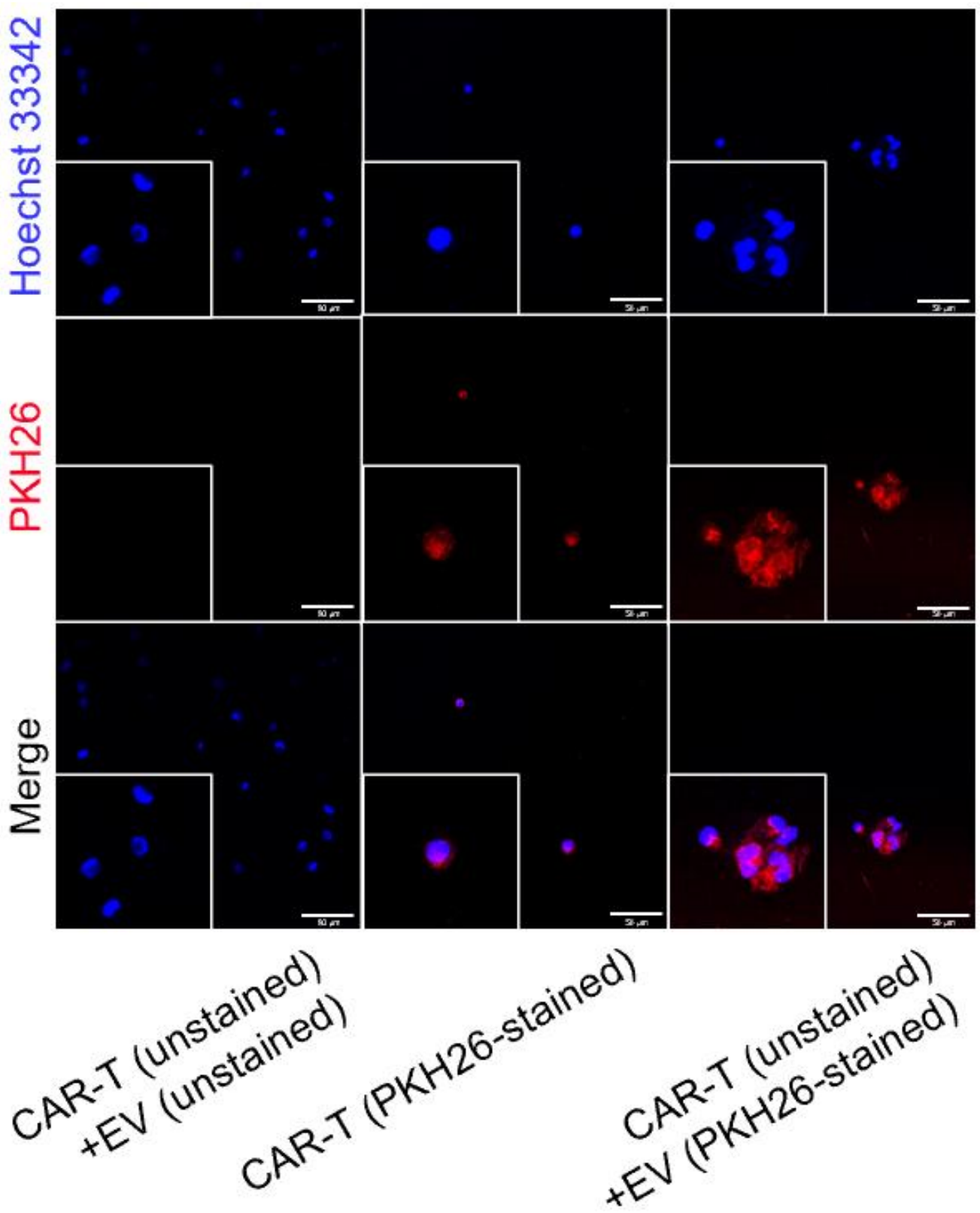

2nd revised Fig. 3a

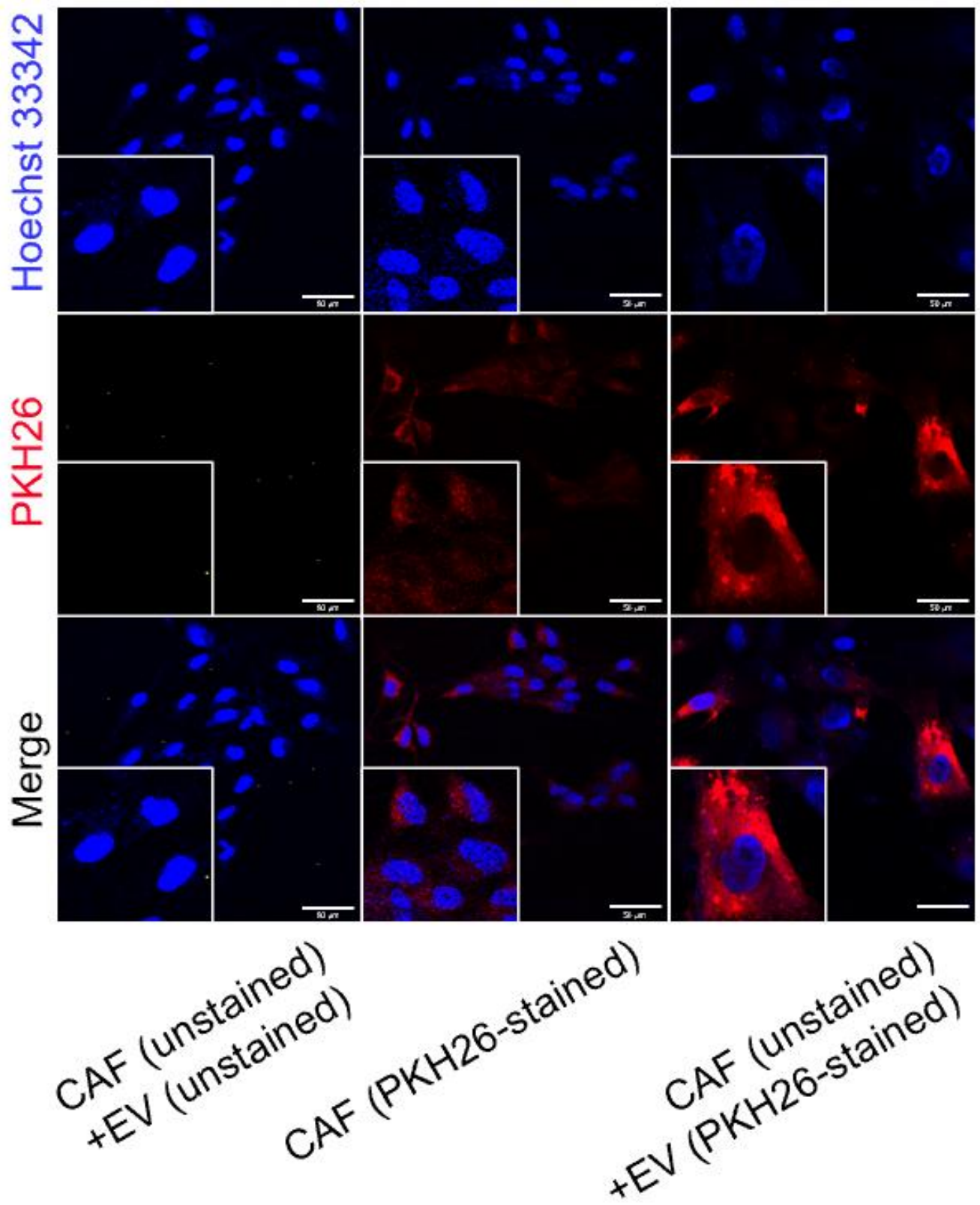

2nd revised Fig. 4a

3. For the scope of EVs' fluorescent staining

More importantly, we should emphasize that this manuscript focuses on the importance of EV-proteins in affecting CLDN18.2-CAR-T or antibody therapies. Our fluorescent staining for EVs is a qualitative, rather than a quantitative analysis. This staining experiment is designed as a quality control, only to demonstrate a cellular basis that EVs may influence the function of cells, while measuring the degree of EVs uptake between CAR-T cells and CAFs is beyond the scope of our study. The detailed influences and relevant mechanisms of EV-proteins on CAR-T cells, on CAFs and on antibodies have been verified by other comprehensive experiments in our study.

Part3: CLDN18.2 EVs interacts with CAFs promoting the release of IL-6 blocking CAR-T efficacy:

The raw data provided for the proximity labeling do not match with the results presented. 17 proteins are exclusively found in CLDN18.2-TurboID-TM and not in control conditions. Then, 17 proteins have at least 2-fold difference in in CLDN18.2-TurboID-TM compared to control. Some of these 17 proteins are presented in the heatmap above. S100A4 is ranked 12/17. Similarly, 4 proteins are found exclusively in the control conditions and 30 proteins are upregulated by at least 2 fold in the control condition compared to CLDN18.2-TurboID-TM. Some proteins included in the heatmap are detected in only 1 replicate. The authors should carefully analyzed the data given the low number of replicates

and further explain how they established their list of top 10 and bottom 10 proteins. Several proteins detected are associated to T/NK activity (e.g Gzm).

A. We sincerely apologize for all the ambiguous descriptions about proximity labeling. This part is generated by referring to the proteomics data we have provided in a previously published study, as cited in the manuscript. We inadequately demonstrated a previous version of analysis, which leads to inappropriate description and should be updated in this revision.

As you have pointed out, protein expression from the mass-spectrum data should be ranked according to the foldchanges of CLDN18.2-TurboID-TM group's average compared to control group's average. We set up the following standards to re-analyze the mass-spectrum data for better presentation: For proteins harboring >2 foldchanges of CLDN18.2-TurboID-TM vs SP-TurboID-TM, the expression value should be larger than zero and rank within the upper three quartiles (highest 75%) for at least one of CLDN18.2-TurboID-TM's replicate; For proteins harboring <0.5 foldchanges of CLDN18.2-TurboID-TM vs SP-TurboID-TM, the expression value should be larger than zero and rank within the upper three quartiles (highest 75%) for at least one of SP-TurboID-TM's replicate. According to these standards, 16 proteins displayed high interaction affinity with CLDN18.2 and 29 proteins displayed low interaction affinity with CLDN18.2 were identified, in which S100A4 was ranked 11/16 (**Fig for review only. 8**). We have described the analytical standards in Methods section and incorporated the new data into the **2nd revised Fig. 4o**.

Accession	Protein ID	ctrl1 (SP-TuroboID-TM)	ctrl2 (SP-TuroboID-TM)	CLDN18.2-1 (CLDN18.2-TuroboID-TM)	CLDN18.2-2 (CLDN18.2-TuroboID-TM)	Rank-ctrl1	Rank-ctrl2	Rank-CLDN18.2-1	Rank-CLDN18.2-2	Protein ID	AVR-ctrl	AVR-CLDN18.2	FC-CLDN18.2 vs ctrl	Definition
Q8BGY2	EIF5A2	0	0.007213851	0.058467424	0.061417973	286	273	189	177	EIF5A2	0.0036069	0.0599427	16.618779	high affinity
P0DOV2	IFI204	0.037158102	0	0.058467424	0.204413744	251	290	189	76	IFI204	0.0185791	0.1314406	7.0746661	high affinity
Q9D8C4	IFI35	0	0.007213851	0.016948154	0.031035795	286	273	253	229	IFI35	0.0036069	0.023992	6.6516414	high affinity
O70325	GPX4	0	0.007213851	0.008498964	0.031035795	286	273	284	229	GPX4	0.0036069	0.0197674	5.480396	high affinity
P83882	RPL36A	0.014978281	0.035713865	0.082817285	0.100957172	273	219	156	131	RPL36A	0.0253461	0.0918872	3.6253044	high affinity
Q8JZU2	SLC25A1	0.007508579	0.01439181	0.05025861	0.020764536	280	263	201	247	SLC25A1	0.0109502	0.0355116	3.2430085	high affinity
P08884	GZME	0	0.049755487	0.058467424	0.071404827	286	196	189	161	GZME	0.0248777	0.0649361	2.6102096	high affinity
P13366	GZMG	0	0.056725378	0.066629794	0.081323022	286	185	175	150	GZMG	0.0283627	0.0739764	2.6082297	high affinity
P67984	RPL22	0.421347328	0.595793722	1.201541206	1.427013598	27	12	8	5	RPL22	0.5085705	1.3142774	2.5842579	high affinity
Q3THE2	MYL12B	0.620554584	0.783951669	1.782482901	1.734118389	18	10	5	4	MYL12B	0.7022531	1.7583006	2.503799	high affinity
P07091	S100A4	0.029802651	0.02864147	0.098825161	0.041234446	258	231	137	212	S100A4	0.0292221	0.0700298	2.3964704	high affinity
P62806	H4C1	0.229216122	0.12463264	0.36682582	0.476555684	67	109	33	26	H4C1	0.1769244	0.4216908	2.3834519	high affinity
Q9JHW4	EEFSEC	0.044476242	0.007213851	0.016948154	0.100957172	239	273	253	131	EEFSEC	0.025845	0.0589527	2.2810043	high affinity
P19324	SERPINH1	0.051757448	0.021534233	0.122508786	0.041234446	223	244	119	212	SERPINH1	0.0366458	0.0818716	2.2341312	high affinity
P61979	HNRNPK	0.05900209	0.042751759	0.082817285	0.129916294	212	207	156	112	HNRNPK	0.0508769	0.1063668	2.0906686	high affinity
P07356	ANXA2	0.143213604	0.02864147	0.106762981	0.249093861	112	231	128	54	ANXA2	0.0859275	0.1779284	2.0706799	high affinity
P62843	RPS15	0.163520241	0.176747025	0.138084592	0.031035795	97	79	110	229	RPS15	0.1701336	0.0845602	0.4970222	low affinity
O89053	CORO1A	0.066210535	0.170334752	0.033699519	0.081323022	203	84	220	150	CORO1A	0.1182726	0.0575113	0.4862601	low affinity
Q8BVY0	RSL1D1	0.101722199	0.056725378	0.025348149	0.051361506	148	185	236	192	RSL1D1	0.0792238	0.0383548	0.4841327	low affinity
Q91YR1	TWF1	0.176900925	0.163893851	0.082817285	0.081323022	92	86	156	150	TWF1	0.1703974	0.0820702	0.4816397	low affinity
Q8VEK3	HNRNPU	0.122617056	0.144396797	0.066629794	0.061417973	126	95	175	177	HNRNPU	0.1335069	0.0640239	0.4795548	low affinity
Q99K48	NONO	0.05900209	0.035713865	0.033699519	0.010419625	212	219	220	268	NONO	0.047358	0.0220596	0.4658048	low affinity
Q99KP6	PRPF19	0.044476242	0.021534233	0.008498964	0.020764536	239	244	284	247	PRPF19	0.0330052	0.0146318	0.443316	low affinity
P46735	MYO1B	0.150014291	0.08427302	0.05025861	0.051361506	105	155	201	192	MYO1B	0.1171437	0.0508101	0.4337414	low affinity
P11103	PARP1	0.051757448	0.111304492	0.016948154	0.051361506	223	120	253	192	PARP1	0.081531	0.0341548	0.4189185	low affinity
Q00547	HMMR	0.129515296	0.070564949	0.042002822	0.041234446	123	172	211	212	HMMR	0.1000401	0.0416186	0.4160194	low affinity
Q8C0C7	FARSA	0.044476242	0.021534233	0.016948154	0.010419625	239	244	253	268	FARSA	0.0330052	0.0136839	0.4145975	low affinity
Q922B2	DARS1	0.007508579	0.01439181	0.008498964	0	280	263	284	298	DARS1	0.0109502	0.0042495	0.3880737	low affinity
P01872	IGHM	0.014978281	0.007213851	0.008498964	0	273	273	284	298	IGHM	0.0110961	0.0042495	0.382972	low affinity
Q921N6	DDX27	0.080520264	0.077435266	0.008498964	0.051361506	178	163	284	192	DDX27	0.0789778	0.0299302	0.3789704	low affinity
O55142	RPL35A	0.051757448	0.021534233	0.016948154	0.010419625	223	244	253	268	RPL35A	0.0366458	0.0136839	0.3734091	low affinity
Q68FD5	CLTC	0.229216122	0.035713865	0.066629794	0.031035795	67	219	175	229	CLTC	0.132465	0.0488328	0.3686468	low affinity
Q8R1A4	DOCK7	0.156783071	0.01439181	0.042002822	0.020764536	102	263	211	247	DOCK7	0.0855874	0.0313837	0.3666856	low affinity
P05977	MYL1	0.150014291	0.251550338	0.145809885	0	105	52	105	298	MYL1	0.2007823	0.0729049	0.3631044	low affinity
O54774	AP3D1	0.051757448	0.042751759	0.008498964	0.020764536	223	207	284	247	AP3D1	0.0472546	0.0146318	0.3096365	low affinity
Q6P542	ABCF1	0.129515296	0.035713865	0.025348149	0.020764536	123	219	236	247	ABCF1	0.0826146	0.0230563	0.2790832	low affinity
Q62261	SPTBN1	0.229216122	0.056725378	0.016948154	0.061417973	67	185	253	177	SPTBN1	0.1429708	0.0391831	0.2740635	low affinity
Q8CB77	ELOA	0.073383141	0.007213851	0	0.020764536	190	273	302	247	ELOA	0.0402985	0.0103823	0.2576341	low affinity
O35643	AP1B1	0.190158646	0.097852066	0.066629794	0	80	140	175	298	AP1B1	0.1440054	0.0333149	0.2313448	low affinity
Q3U7R1	ESYT1	0.115685674	0.131250822	0.025348149	0.020764536	131	105	236	247	ESYT1	0.1234682	0.0230563	0.186739	low affinity
P27773	PDIA3	0.073383141	0.091078519	0.025348149	0	190	146	236	298	PDIA3	0.0822308	0.0126741	0.154128	low affinity
Q5SX39	MYH4	0.190158646	0.942872529	0.058467424	0.031035795	80	7	189	229	MYH4	0.5665156	0.0447516	0.0789945	low affinity
Q8BQZ4	RALGAPB	0.101722199	0.007213851	0	0	148	273	302	298	RALGAPB	0.054468	0	0	low affinity
Q5SX40	MYH1	0.176900925	0.808934747	0	0	92	9	302	298	MYH1	0.4929178	0	0	low affinity
P58774	TPM2	0.254679103	0.36258615	0	0	53	31	302	298	TPM2	0.3086326	0	0	low affinity

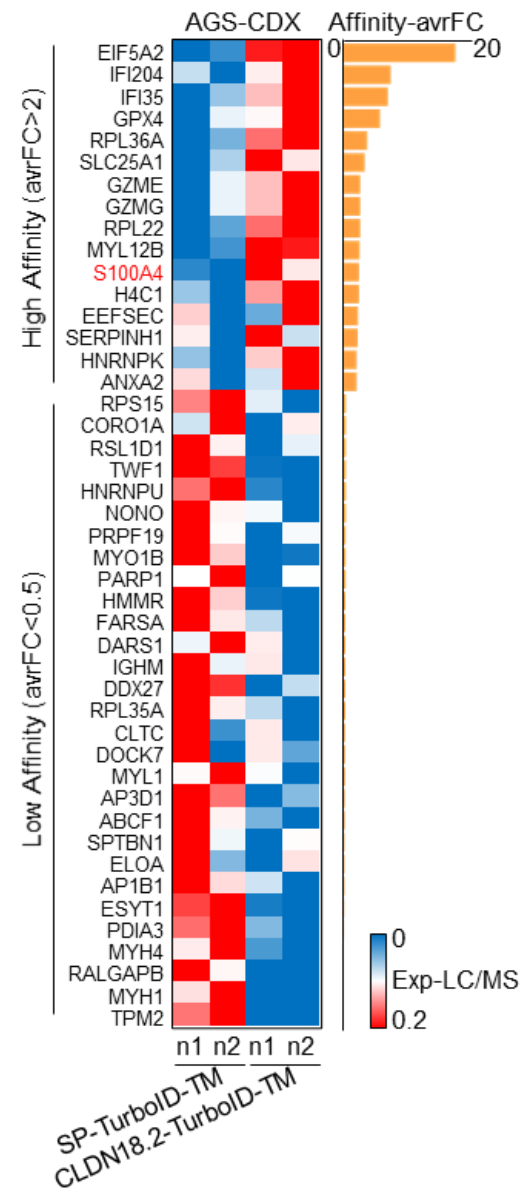

Fig for review only. 8

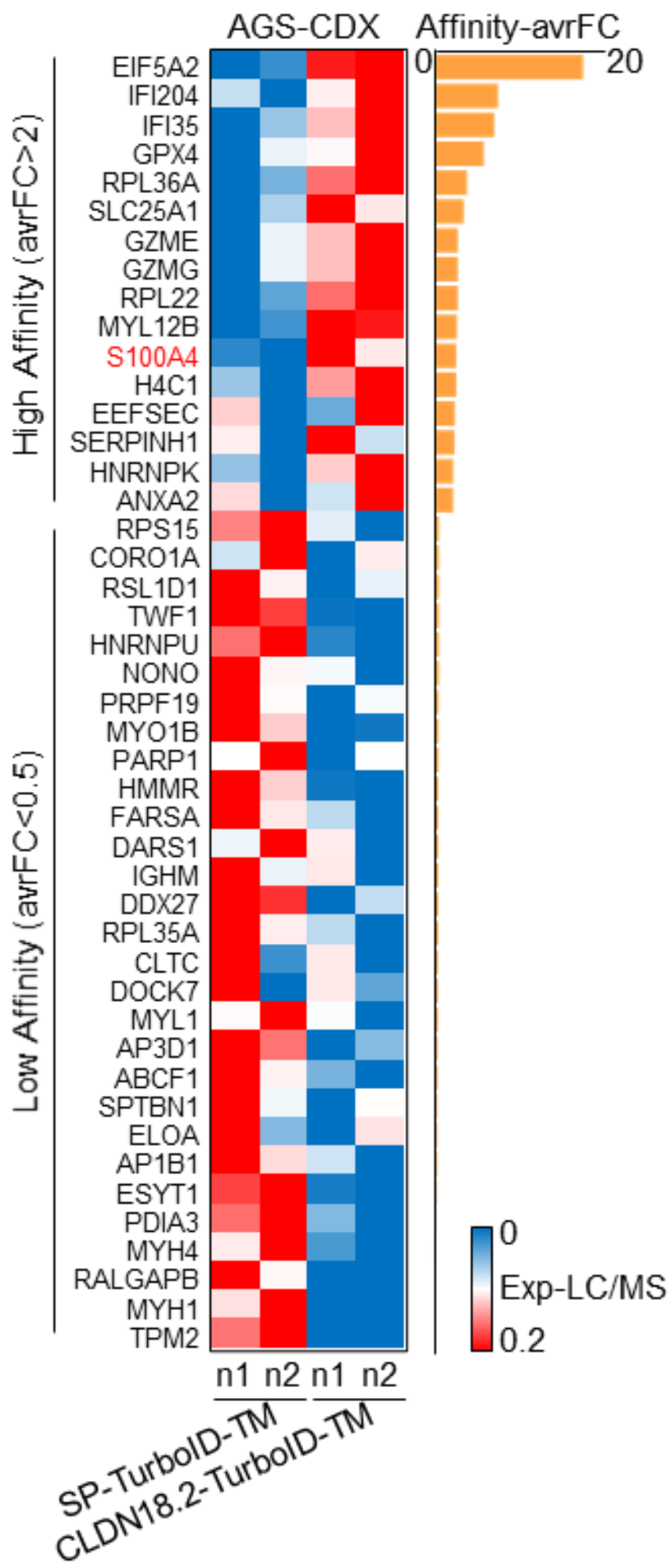

2nd Revised Fig. 4o

As we have described in the manuscript, before analyzing CLDN18.2's proximity labeling data, we have noticed the elevated levels of IL-6/CXCL8 (fibroblast-related cytokines) in plasma of high CPP-score patients (Fig. 2i), and proved that EV-CLDN18.2 amplified CAF's interference on CAR-T's efficacy through increasing the proportion of an IL-6/CXCL8 expressing CAF linage (Fig. 4a-4m). Inspired by these evidences, we next sought to prove the association between EV-CLDN18.2 and CAFs, rather than other TIME cells such as cytotoxic T or NK cells. Therefore, we re-analyzed CLDN18.2's proximity labeling data to specifically affirm the strong association between CLDN18.2 and CAFs (Fig. 4o). Although S100A4's interaction affinity with CLDN18.2 ranked 11/16, it was the highest rank observed among canonical CAF marker genes, that's why we selected S100A4 for subsequent analysis. The interaction between EV-CLDN18.2 and S100A4 has been verified by the subsequent pulldown assay (Fig. 4s) and binding affinity assay (Fig. 4t).

The authors proposed revisions for Figure 7d, 8c with additional replicates. However, it is unclear how they were able to generate novel replicates from the same PDX mouse model and the same donor-derived CAR-T.

A. Thank you for the question, we apologize for the ambiguous demonstrations. We have improved the descriptions and corrected some calculation errors in the 2nd revision.

1. For Fig. 7d

In this part, our previous flow cytometry experiments have generated the data for 2 replicates. We only presented the flow charts and data of one representative replicate in the originally submitted Fig. 7d. In the 1st revision, we provided the flow charts of only one representative replicate due to limited figure space, but formatted the bar plots with the data of both replicates.

Here we are providing the data & charts of all replicates for your reference (**Fig for review only. 9**). We apologize for the ambiguous demonstrations in the 1st revision, and have improved the descriptions for Fig. 7d.

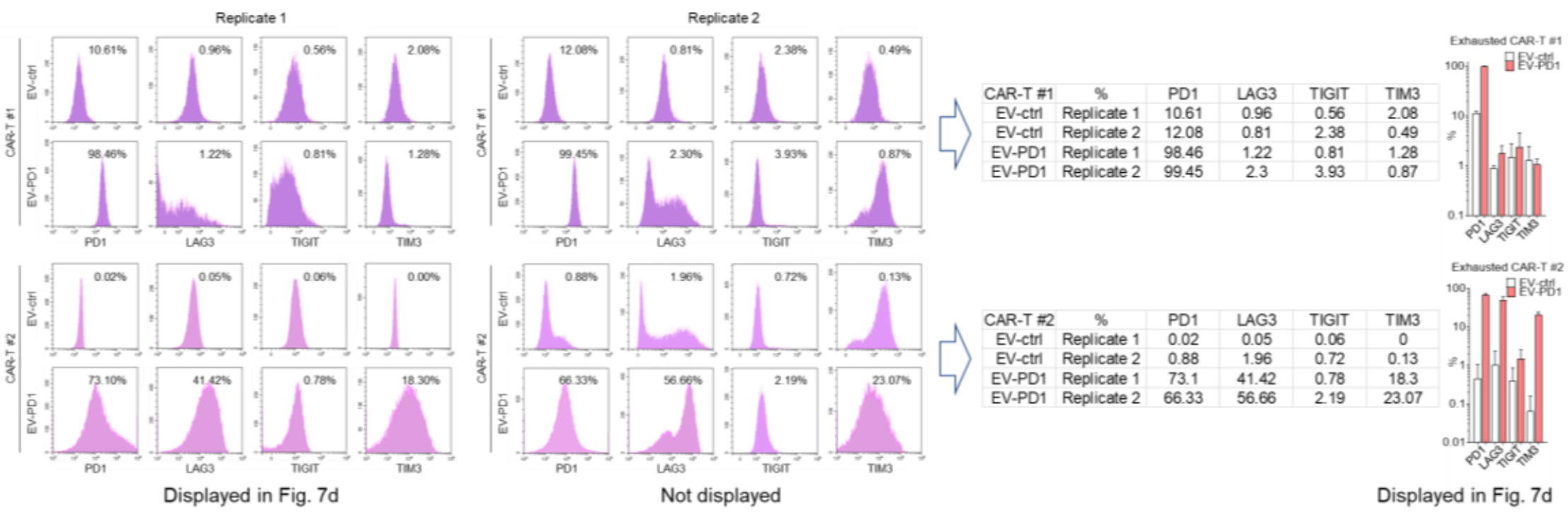

Fig for review only. 9

2. For Fig. 8c

Through applying EV-array assessment, we have previously generated the data for 3 replicates of EV-CLDN18.2's relative expression fold change in different timepoint of PDX plasma for Fig. 8c. We presented the average expression value in the original submission and in the 1st revision. Here we are providing the data & charts of all replicates for your reference (**Fig for review only. 10**). We apologize for the ambiguous demonstrations in the 1st revision, and have improved the descriptions for Fig. 8c.

	Relative expression fold change			
Sample	Replicate 1	Replicate 2	Replicate 3	Mean
CAR-T-before PE	0.083373111	0.508772216	0.022091572	0.20475
CAR-T-PE1	0.952532282	1.875558148	0.17190957	1
	Relative expression fold change			
Sample	Replicate 1	Replicate 2	Replicate 3	Mean
CAR-T-PE2	1.257967978	1.127186141	0.614845881	1
CAR-T-PE-18.2negative	0.298205936	0.485399663	0.356549018	0.38005
CAR-T-PE-WT	0.199520908	0.458022993	0.466470891	0.37467

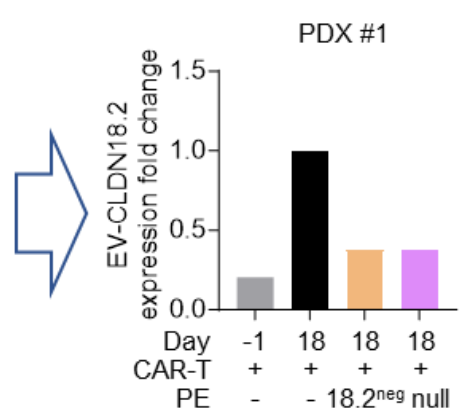

Fig for review only. 10

Reviewer #3 (Remarks to the Author):

The authors have done an exceptional work adressing all my comments, I sincerely congratulate them for their interesting work.

A. We sincerely appreciate your valuable suggestions and recognition of our work.

Reviewer #4 (Remarks to the Author):

Indeed referee 2 questioned whether the EV analysis was comparable to the current state of the art. In the original article the authors wrote their EV protocol used one 15k centrifigutaion. In their response the authors claim that this was an error and that they used a 100k step.

I am not convinced by the response of the auhtors. Typically EVs are isolated by sequenctial multi-step differential ultracentrifigation. Purity is ontained with sucrose-density gradients. I agree that these EVs are not usefull for functional experiments but they are usefull to better analyze the EV-associated proteins. The authors should adhere as much as possible to the MISEV 2023 guidelines and show that their EV preps lack ER proteins but enclose ESCRT and/or syntenin markers for example. Typically a pane of 5 proteins is used to show puritiy of EVs. Also the quantitation based on BSA is often misleading as the authors statte themselves, contaminating proteins in the EV corona may obscure the actual amount of EVs. On that note NTA is used by many, but not a very accurate tool for EV concentration unless, the EVs preps are made ultrapure, which is not the case with the methods used (or at least as explained).

Also im not convinced by their EM data, these are single EVs with immuno-EM, FACS analysis would be much more appropiate to show single EV analysis of their markers. Indeed their elisa-like assays using minimal input of serum may or may not report on EV-proteins. Spike-in experiments with purified EV that contain their markers in EV-depleted on control plasma/serum should be performed imo.

A. Thank you for the professional and rigorous comment, which allows us to further clarify the fundamental rationale behind our methodological choices. We apologize for all the misleading parts in previous versions, and have improved relevant descriptions in this revised manuscript.

1. For the question about ultracentrifugation, EV purity, and EV concentration

We fully acknowledge the value of sequential ultracentrifugation and sucrose-density gradients for obtaining ultra-pure EVs for fundamental biological studies. In our study, to produce EVs expressing crucial proteins, we used a CLDN18.2-overexpressing cell subline, a PD1-overexpressing cell subline and a PD-L2-overexpressing cell subline, then isolated EVs from the culture medium supernatant of these cells. We used the tangential flow filtration (TFF) plus ultracentrifugation method for the isolation of EVs highly expressing CLDN18.2/PD1/PD-L2 proteins, in order to reserve their biological activities. We apologize for the ambiguous descriptions in previous manuscript, and have more carefully described the related procedures in Methods section of 2nd revised manuscript, as stated below:

- To produce EVs expressing specific proteins, a human-CLDN18.2-overexpressing HEK293F cell subline, a human-PD1-overexpressing HEK293F cell subline and a mouse-PD-L2-overexpressing CT26 cell subline were cultured in medium containing exosome-free serum and proceeded for the collection of culture medium supernatant when cell density reached 80%. Culture medium was centrifugated at 300 ×g for 10 min to collect the supernatant. Supernatant was centrifugated at 2,000 ×g for 15 min to remove cell debris, then diluted by seven-fold volume of PBS and processed through a 0.22 μm vacuum filter to remove large particles. Supernatant was diafiltrated and concentrated using Hydrosart® TFF (tangential flow filtration) device with 300 kDa cut-off filters (item number: 3M51467901E-SW, Sartorius, NY, USA), then ultracentrifuged using a P50A72-986 rotor (CP100NX; Hitachi, Brea, CA, USA) at 100,000 ×g, 4 °C for 2 h to pellet EVs. Pelleted

EVs was resuspended in PBS and centrifuged again at 100,000 ×g, 4 °C for 2 h. After PBS wash, isolated EVs was re-suspended in 100 µL PBS.

TFF plus ultracentrifugation has been employed by multiple studies (e.g., *Nat Commun. 2023 Oct 23;14(1):6692; J Extracell Vesicles. 2024 Dec;13(12):e70018; J Control Release. 2024 Nov:375:105-115*), demonstrating its effectiveness in purifying EVs. However, sucrose-density is suggested, but not mandated by MISEV 2023 guidelines. Due to the limited time and resource, we did not perform sucrose-density gradients during the isolation of these cell-derived EVs. Instead, we performed NTA to verify EVs' particle size, western blot to verify the purity of EVs, and immunogold labeling to verify the expression of specific proteins on EVs (Extended Data Fig. 7b-7d, Fig. 7a-7c, Fig. 7f-7h).

Second, we must respectfully emphasize that the objectives of our work are to screen clinically related EV-proteins from plasma of patients, to elucidate the therapeutic effects & relevant functional mechanisms of these EV-proteins, and to explore potential strategies countering EV-proteins' interference. We have thoroughly validated these objectives through comprehensive evidences. Therefore, as long as specific proteins are expressed on extracellular vesicles, they can be defined as EV-proteins. Whether proteins are exclusive expressed on EVs, or whether EVs are precisely purified & quantified, have no influence on the conclusions or overall interpretation of this study.

2. For the question about ER proteins and ESCRT/syntenin markers

For quality control, ER (Endoplasmic reticulum) proteins such as Calnexin and GRP94 are generally negative, while ESCRT/syntenin proteins such as CD81, CD9, CD63, Alix, TSG101 and Syntenin-1 are generally positive on isolated EVs. We have already provided Western Blot for isolated EVs derived from HEK293F cells (Extended Data Fig. 7c), in which ER protein (Calnexin, as the negative EV marker) and ESCRT/syntenin proteins (CD81/CD9, as positive EV markers) both exhibit appropriate indications for the high quality of isolated EVs. Since EVs derived from CT26 cells underwent the same isolation steps, it is reasonable to expect that they possess comparable quality.

As for the number of EV markers, a series of other EV-related works published in high-impact journals also employed less than 5 EV markers, such as the *Nature Communications* paper shown below. Therefore, while a comprehensive panel (e.g., assessing all ER/ESCRT markers) is excellent for the characterization of EVs' purity, it is not a prerequisite for validating the cell-derived EV-proteins isolated for functional assessments, or for the clinically oriented EV-array assay (whose specificity we have confirmed through more direct and relevant functional tests, as presented above in response to the concerns raised by you and Reviewer #2).

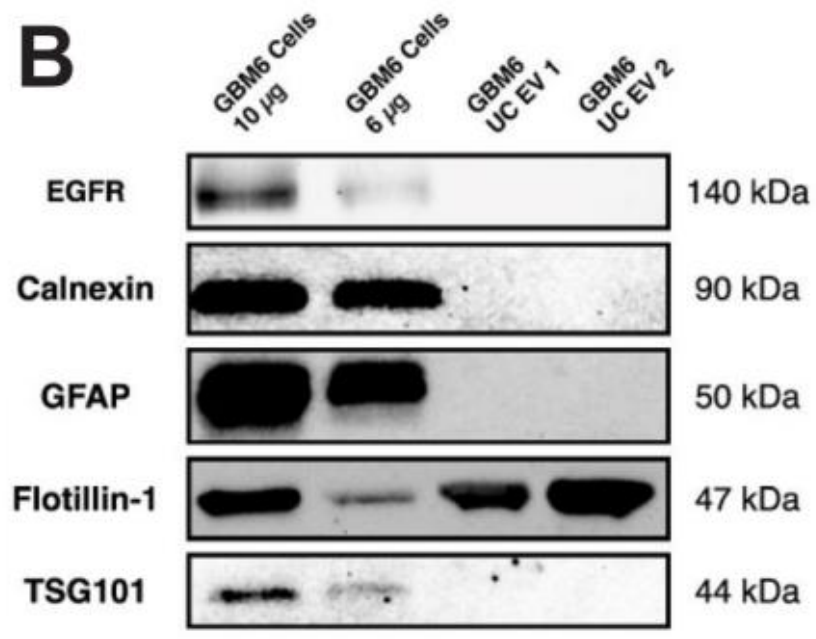

Cited Fig. 1B using Calnexin, TSG101 and Flotillin-1 to mark vesicles from the supernatant of cultured GBM cells (Dynamic release of extracellular particles after opening of the blood-brain barrier predicts glioblastoma susceptibility to paclitaxel. Nat Commun 2025 Dec 16;16(1):11045)

Furthermore, multiple established methodologies (including NTA and immunogold labelling assay, result shown in Extended Data Fig. 7b-7d, Fig. 7a-7c, Fig. 7f-7h) were utilized to verify that the quality of isolated EVs adhere to the MISEV 2023 guidelines.

3. For the question about EVs quantitation

We assume here you are mentioning BCA (Bicinchoninic Acid) method, which we used to quantify the amount of EVs isolated from cells' culture medium supernatant. We agree that techniques like NTA and BCA have limitations in impure preparations, but these limitations do not affect the core principle of our study. As we have previously clarified, the scope of this study is confined to investigating the clinical, functional, and translational involvement of certain EV proteins in CLDN18.2-targeted anti-cancer therapies. Therefore, it is the relative quantification of isolated EVs, rather than the absolute quantification, is needed in this study. All the data related with experimental EV-protein groups (EV-CLDN18.2/EV-PD1/EV-PD-L2) are paired with control (EV-ctrl). BCA assay was solely employed to obtain a rapid, approximate protein quantification to administrating identical amount of EVs in functional studies, enabling the comparison of differences in EV-CLDN18.2/EV-PD1/EV-PD-L2 vs EV-ctrl groups. Because EV-CLDN18.2/EV-PD1/EV-PD-L2 and EV-ctrl were isolated from the same cell line (HEK293F or CT26), BCA quantification and NTA assessment are sufficient for us to generate experimental evidences.

4. For the question about immuno-EM and FACs

Immuno-EM (immunogold labeling assay) and FACs (flow cytometry) have distinct detection principles and applications. We acknowledge that FACs method provides a powerful tool for the quantification of single EVs. However, as previously clarified, the scope of this study is focused on investigating the clinical significance, functional involvement, and translational applications of specific EV proteins in CLDN18.2-targeted anti-cancer therapies. We implemented two main measurements for EVs to meet the scope: 1. We screened clinically relevant EV-proteins via EV-array (EV-CLDN18.2/EV-PD1/EV-PD-L2), then stratified cohort patients into "high" and "low" expression groups for each EV-protein using median-based cutoffs. 2. We extracted EV-CLDN18.2/EV-PD1/EV-PD-L2 proteins from the culture medium supernatant of CLDN18.2/PD1/PD-L2 overexpressed cell sublines, then studied the functions of these EV-proteins. These measurements demand qualitative verification for the expression of proteins on EVs, rather than precise concentration or quantification of single EVs. Therefore, we adopted immunogold labeling assay (paired with NTA, TEM and Western Blot) to provide the direct evidence that certain proteins (such as CLDN18.2/PD1/PD-L2) are expressed on plasma EVs, EV-array effectively detects these EV-proteins from patient plasma, and that EVs extracted from cells' culture medium supernatant harbor the artificial expression of these proteins.

On the other hand, since we did not require single-EV quantification, we did not employ FACs assessment for the isolation of EVs from cells. Moreover, the limited volume of patient plasma samples from completed clinical trials also precluded the use of FACs assessment for plasma EVs.

5. For the question about EV-array

We wish to clarify that EV-array is a established method, specifically designed for the detection of EV-protein expression. This method has been adopted & verified by a series of representative studies from different research groups, as listed below. Therefore, it is a reliable method for the phenotyping of extracellular vesicles.

- *Jørgensen MM, Bæk R, Sloth JK, et al. A novel multiple marker microarray analyzer and methodology to predict major obstetric syndromes using surface markers of circulating extracellular vesicles from maternal plasma. Acta Obstet Gynecol Scand. 2025;104(1):151-163. doi:10.1111/aogs.15020*
- *Jung AL, Møller Jørgensen M, Bæk R, et al. Surface Proteome of Plasma Extracellular Vesicles as Biomarkers for Pneumonia and Acute Exacerbation of Chronic Obstructive Pulmonary Disease. J Infect Dis. 2020;221(2):325-335.*
- *Bæk R, Jørgensen MM. Multiplexed Phenotyping of Small Extracellular Vesicles Using Protein Microarray (EV Array). Methods Mol Biol. 2017;1545:117-127.*
- *Sandfeld-Paulsen B, Aggerholm-Pedersen N, Bæk R, et al. Exosomal proteins as prognostic biomarkers in non-small cell lung cancer. Mol Oncol. 2016;10(10):1595-1602.*
- *Jørgensen MM, Bæk R, Varming K. Potentials and capabilities of the Extracellular Vesicle (EV) Array. J Extracell Vesicles. 2015;4:26048.*
- *Jørgensen M, Bæk R, Pedersen S, Søndergaard EK, Kristensen SR, Varming K. Extracellular Vesicle (EV) Array: microarray capturing of exosomes and other extracellular vesicles for multiplexed phenotyping. J Extracell Vesicles. 2013 Jun 18:2.*
- *Chen T, Wang W, Qian Y, et al. Immune checkpoint inhibitors combined with anti-angiogenic therapy as second- or further-line treatment for small cell lung cancer: Efficacy, safety, and prognostic biomarkers. Int J Cancer. 2026;158(3):666-681.*
- *Sun L, Wei X, Zhao Q, et al. Dynamic Change of PD-L2 on Circulating Plasma Extracellular Vesicles as a Predictor of Treatment Response in Melanoma Patients Receiving Anti-PD-1 Therapy. J Extracell Vesicles. 2025;14(4):e70054.*
- *Huang Z, Deng C, Ma C, et al. Identification and validation of the surface proteins FIBG, PDGF-β, and TGF-β on serum extracellular vesicles for non-invasive detection of colorectal cancer: experimental study. Int J Surg. 2024;110(8):4672-4687.*
- *Chen D, Li L, Wang M, et al. Efficacy and safety of immunotherapy combined with single-agent chemotherapy as second- or later-line therapy for metastatic non-small cell lung cancer. Front Immunol. 2023;14:1086479.*

- *Chen D, Li L, Wang M, et al. Efficacy and safety of immunotherapy combined with single-agent chemotherapy as second- or later-line therapy for metastatic non-small cell lung cancer. Front Immunol. 2023;14:1086479.*
- *Jiang F, Zhang Z, Chong X, et al. Extracellular Vesicle-Derived Protein File from Peripheral Blood Predicts Immune-Related Adverse Events in Gastric Cancer Patients Receiving Immunotherapy. Cancers (Basel). 2022;14(17):4167.*
- *Zhang C, Chong X, Jiang F, Gao J, Chen Y, Jia K, Fan M, Liu X, An J, Li J, Zhang X, Shen L. Plasma extracellular vesicle derived protein profile predicting and monitoring immunotherapeutic outcomes of gastric cancer. J Extracell Vesicles. 2022 Apr;11(4):e12209.*

As we have stated throughout this manuscript, we used plasma samples to perform EV-array assessment, rather than serum samples. We have conducted a series of experiments to validate the sensitivity, dynamic range, controls, precision, and reproducibility of the EV-array assay, as presented above in response to the concerns raised by you and Reviewer #2. We have also improved the descriptions for the methodology of EV-array.

6. For the comment about spike-in experiment

We appreciate your suggestion about spike-in experiment. We fully agree that such an experiment is a valuable approach to rigorously validate an assay's specificity and linearity. However, in the context of our study directly using patient plasma samples, performing a classic spike-in experiment into "EV-depleted" plasma presents a significant technical challenge. As the reviewer is well aware of, achieving complete and reproducible removal of all endogenous EVs from human plasma to create a true "blank" matrix is extremely difficult, if not impossible, with current methods. Any residual EVs could confound the signal from the spiked-in purified EVs, compromising the interpretation of the recovery and linearity data.

7. Additional evidences from a controlled lysis experiment

Instead, we directly addressed the concerns raised by you and Reviewer #2 through a controlled lysis experiment. We applied 2 plasma samples of GC patients (#A-#B) for EV isolation and subsequent analysis. When isolated plasma-EVs were subjected to lysis using RIPA buffer and sonication—a treatment that disrupts the lipid bilayer of EVs, the concentrations of EV particles detected by NTA were reduced from 3.3×10^{10} particles/mL to 1.2×10^{10} particles/mL, while the majority of proteins' fluorescence signal and spectrum expressions detected by EV-array were also evidently diminished (**Fig for review only. 7**). The reduction in signal intensity and spectrum expressions upon vesicle disruption provides direct and compelling proof that our EV-array assay is dependent on the structural integrity of EVs and is detecting surface-exposed EV-proteins, not co-isolated soluble contaminants. Therefore, this lysis experiment strongly validated that EV-array specifically and reliably detects surface-accessible proteins on EVs, which directly aligns with the scope of our study.

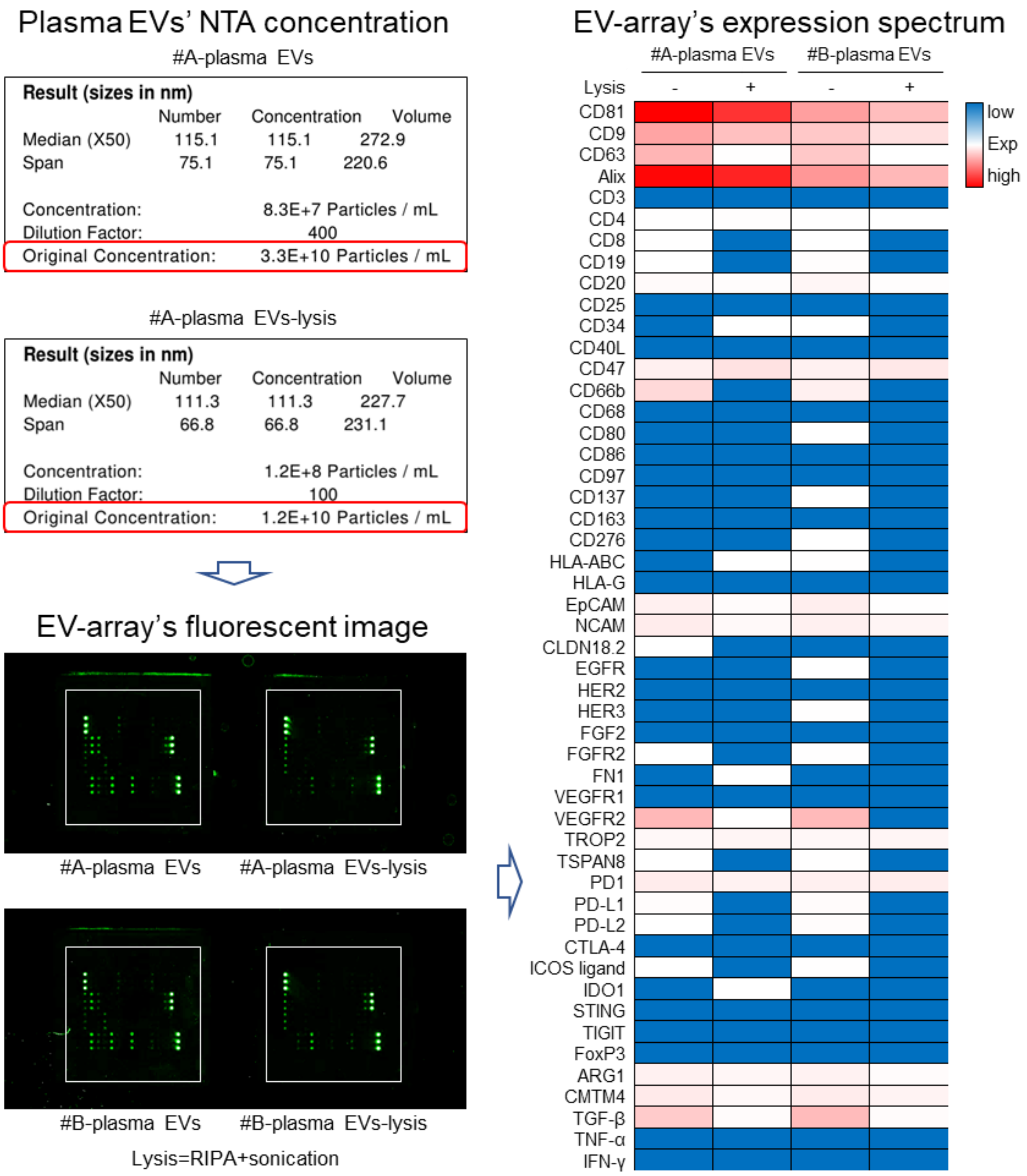

Fig for review only. 7

We sincerely thank the Editors and all Reviewers for their careful evaluation and constructive comments on our manuscript. We greatly appreciate the time and effort invested throughout this rigorous review process.

In this 3rd revision, we have systematically addressed the remaining comments. Specifically, we have performed additional experimental validations, expanded data analyses, and revised the manuscript extensively to improve its clarity, rigor, and overall presentation. We believe these revisions have significantly strengthened the manuscript and provided further evidence to support our central findings regarding the role of EV proteins in CLDN18.2-targeted therapies, thus ensuring a robust and conclusive study.

We have made every effort to address the reviewers' concerns while maintaining the focus on the translational implications of our study. We hope the revised manuscript is now suitable for publication in ***Nature Communications***.

Below, we provide a detailed, point-by-point response to each comment.

REVIEWER COMMENTS

Reviewer #2 (Remarks to the Author):

Thank you for the revised manuscript and rebuttal. I appreciate the additional methodological detail and the steps taken to strengthen the EV workflow (e.g., SEC kit description, NTA/TEM, and proteomics summaries). These additions are helpful. However, several concerns raised in my prior review remain insufficiently addressed in the rebuttal, particularly given the central focus on EV functional effects on CAR-T efficacy, where EV purity and integrity are paramount for data quality and reproducibility:

EV isolation/purity: improved description, but MISEV-aligned purity evidence remains incomplete. While SEC + NTA/TEM + MS summaries are a step forward, NTA/TEM alone cannot establish purity, and the current MS "marker enrichment" presentation does not allow readers to assess co-isolated plasma protein contamination. For plasma EV functional claims, a minimal MISEV-aligned positive/negative marker panel is still expected on the actual EV preparations used in key downstream assays (EV-array and CAR-T functional assays), not only a limited subset. Please add a concise, MISEV-aligned purity panel (multiple positive EV markers plus at least one negative marker) for the relevant EV preparations used across the key experiments.

Conventional purification methods and potential co-isolated soluble components. Because widely used conventional isolation approaches can co-isolate soluble plasma components, the rebuttal does not yet adequately address how such contaminants were excluded/controlled for. This is important because co-isolated factors could influence EV-array readouts and functional assay outcomes, complicating attribution to EVs per se.

A. Thank you for your constructive comments and for the opportunity to further clarify our methodology. We have carefully considered your points and will revise the manuscript accordingly. Below is a point-by-point response to the specific concerns raised regarding EVs.

1. EV isolation/purity

We agree on the importance of providing comprehensive EV characterization. However, we wish to claim again that <u>our study falls into two parts: one for the identification of EV-proteins that indicating therapeutic efficacy, another for the validation of EV-proteins' functions and related mechanisms.</u> Here, we provide a workflow to better illustrate all analyses related to EVs in this study (**Fig for review only. 1**). All methodological details for these analyses have been described in the previous version of the response letter and the revised manuscript.

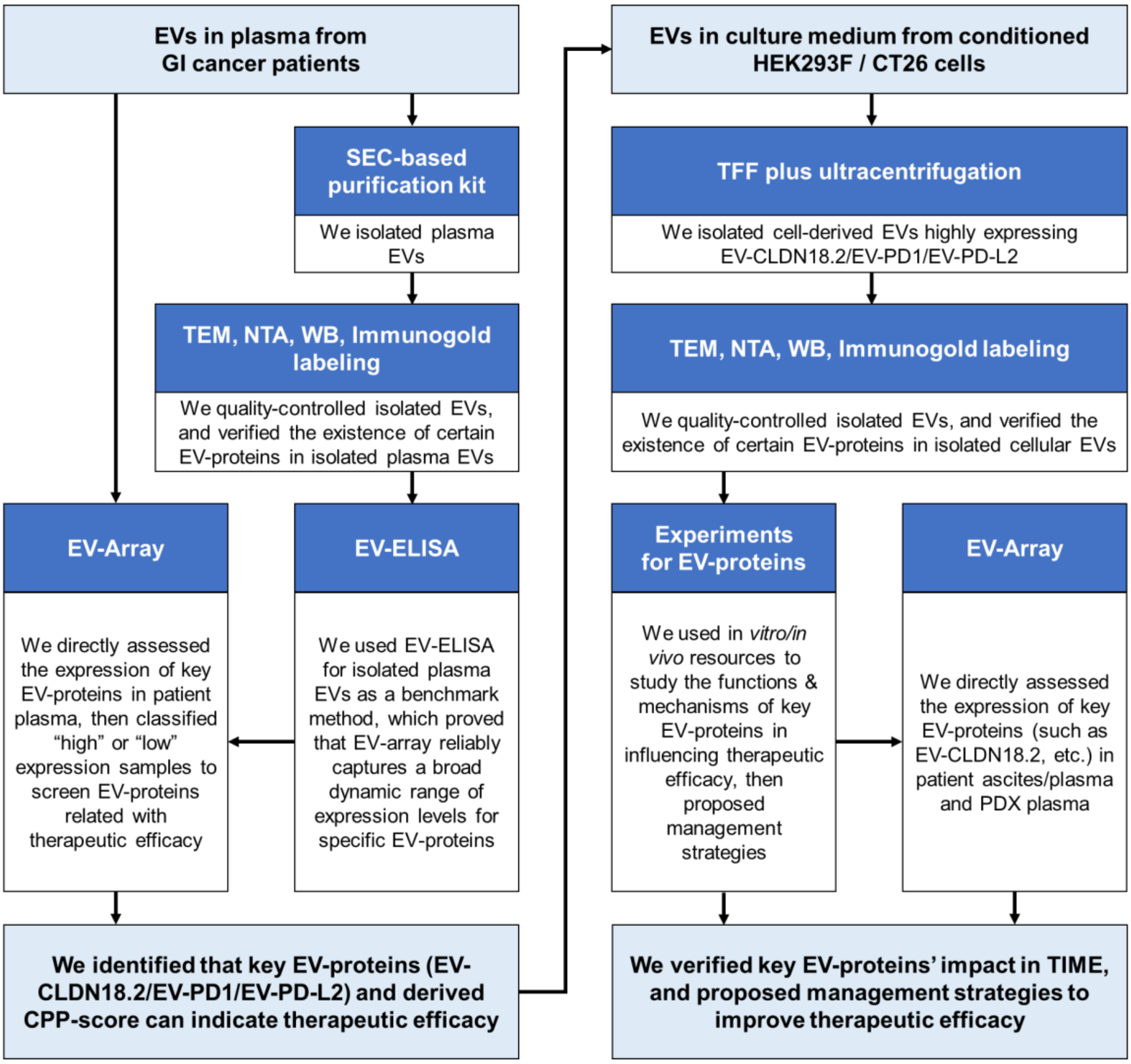

Fig for review only. 1

- Part 1: the identification of EV-proteins that indicating therapeutic efficacy

In this part, our objective is to assess whether the expression levels of specific proteins expressed on EVs (EV-proteins, in short) in plasma are associated with the therapeutic efficacy of patients. Accordingly, our analysis relies on relative comparisons within patient cohorts (i.e., classifying patients into high-versus low-expression of EV-proteins by comparing training/validating cohorts to a 71-patient reference cohort), rather than precisely quantifying the absolute concentrations of these EV-proteins in plasma. While complete exclusion of potential interference from non-EV proteins in plasma remains technically challenging, our study is specifically designed to rely on relative differences within cohorts, which mitigates the impact of such potential confounders and is sufficient for the identification of clinically relevant biomarkers.

The identification of EV-proteins in patient plasma is performed with EV-array, an assay designed to directly measure the expression levels of surface proteins on EVs, without the need for isolating EVs. As detailed in our last revision, we have provided assessments for the sensitivity, dynamic range, negative/positive controls, precision, and reproducibility of the EV-Array assay in detecting specific EV-proteins. We have also demonstrated that using EV-ELISA as a benchmark ensured that EV-array reliably captures a broad dynamic range of expression levels for specific EV-proteins. Based on EV-array

measurements, we categorized each patient plasma samples in training/validating cohorts as “high-” or “low-” expression of EV-proteins according to the medians of the reference cohort. This analysis identified that the relative expressions of 3 key EV-proteins (EV-CLDN18.2/EV-PD1/EV-PD-L2) as well as a composite signature (CPP-score) can indicate therapeutic efficacy.

- Part 2: the validation of EV-proteins’ functions and related mechanisms

In this part, EVs highly expressing key EV-proteins (EV-CLDN18.2/EV-PD1/EV-PD-L2) were isolated exclusively from the culture medium of conditioned cells (CLDN18.2- or PD1-overexpressing HEK293F, and PD-L2-overexpressing CT26 cells), not from plasma. The isolation involved tangential flow filtration (TFF) with a 300 kDa cut-off, followed by ultracentrifugation—a well-established protocol for obtaining high-purity EVs while effectively removing soluble protein contaminants. These isolated cell-derived EVs (EVs highly expressing key EV-proteins, versus EVs from control groups) were quality-controlled with TEM, NTA, WB, and immunogold labeling, then used for all functional & mechanistic assays (including omics/molecular experiments and *in vitro*/*in vivo* efficacy experiments).

Taken together, part 1 and part 2 address distinct but complementary objectives: ① EV-array was applied directly to patient plasma samples to assess relative expression patterns of EV-associated proteins, without requiring EV isolation. As such, potential contributions from non-EV plasma components do not materially affect the identification of relative expression-based biomarkers or the predictive performance of the CPP-score. ② Functional and mechanistic studies were conducted exclusively using well-characterized, cell-derived EVs with controlled expression of the key proteins. This experimental design ensures that the observed biological effects can be attributed to EV-associated proteins rather than co-isolated soluble factors.

2. MISEV-aligned evidences

We apologize for the incomplete authentication. As requested, we have reperformed the WB experiments for the expression of a concise MISEV-aligned purity panel in isolated EVs, including CD81/CD9 as positive EV markers and Calnexin as the negative marker. The expression of these biomarkers confirmed that the purity and integrity of EVs are suitable for downstream analyses. The identification for these EV markers has been incorporated in the revised manuscript (**Revised Fig. 7b, Revised Fig. 7g, Revised Extended Data Fig. 2i, Revised Extended Data Fig. 7c**).

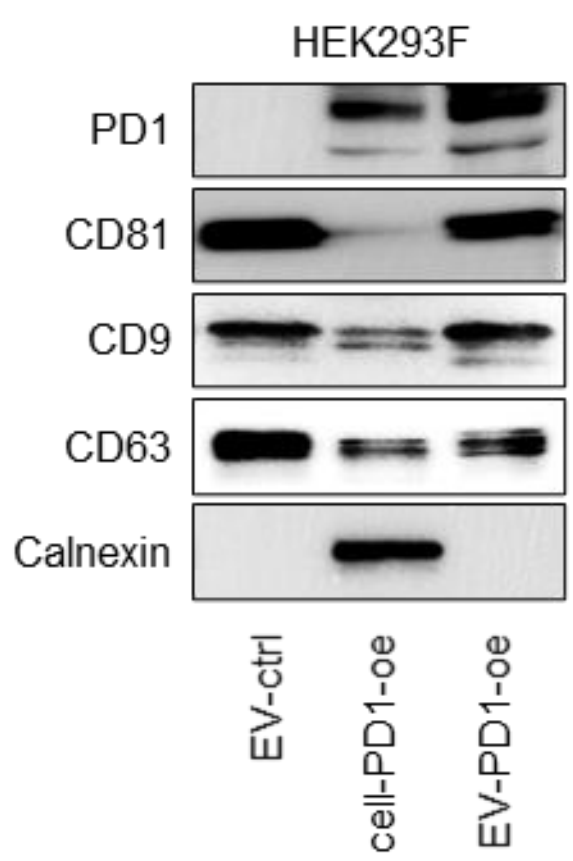

3rd Revised Fig. 7b

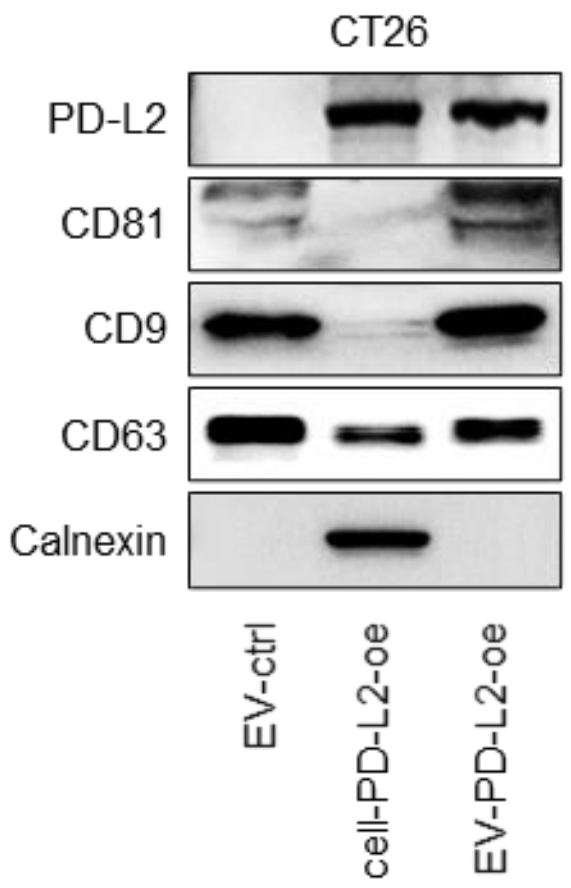

3rd Revised Fig. 7g

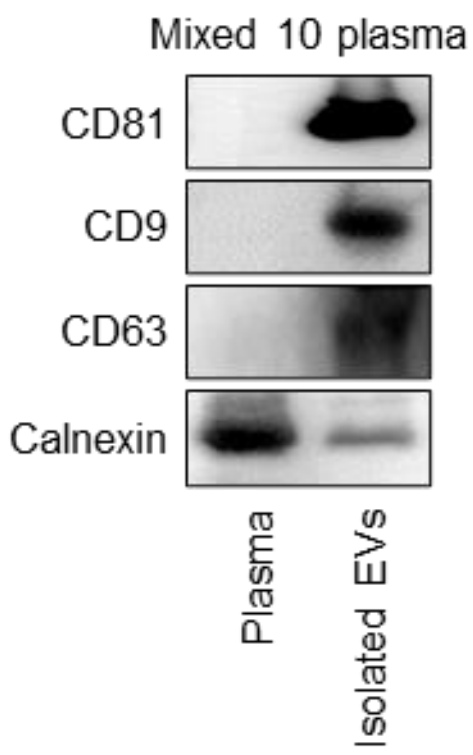

3rd Revised Extended Data Fig. 2i

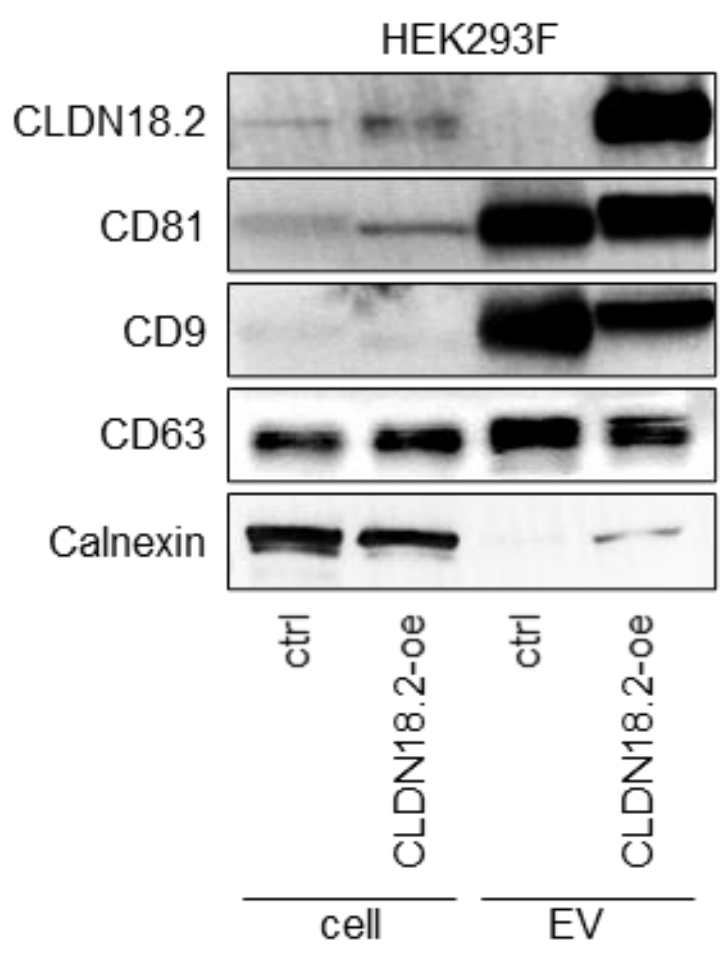

3rd Revised Extended Data Fig. 7c

3. Conventional purification methods and potential co-isolated soluble components

We acknowledge your concern regarding co-isolation of soluble plasma components. This question has been explained in our first point of answers (EV isolation/purity), as shown above.

As we clarified above, patient plasma-derived EVs were used solely for biomarker screening via the EV-array assay, which is designed to directly detect EV-associated proteins in samples. These plasma EVs were not used in any functional & mechanistic assays. Instead, the functional and mechanistic assays were performed exclusively with cell-derived EVs from conditioned cell culture medium, isolated via the stringent TFF/ultracentrifugation protocol mentioned above. TEM, NTA, WB, and immunogold labeling were performed as quality control steps to ensure that isolated cell-derived EVs possess high purity, and highly express specific EV-proteins. This procedures can minimize co-isolation of non-EV components, ensuring that the observed functional effects can be attributed to specific EV-proteins.

The whole workflow related to EVs in this study is demonstrated above in **Fig for review only. 1**. Collectively, our work demonstrated that key EV-proteins act as both biomarkers and functional regulators of CLDN18.2-targeted therapies, irrespective of the potential confounding effects from soluble proteins in non-EV forms.

Replicates and statistical reporting for CAR-T/EV functional assays. In some figures, replicates and/or statistical details appear incomplete or not clearly reported in a way that supports reproducibility. Please ensure that replicate numbers, what constitutes biological vs technical replicates, and the statistical approach are explicitly stated for each relevant experiment. Addressing these points directly in the manuscript (and supplement where appropriate) would substantially improve transparency and confidence that the reported functional effects are truly EV-dependent and reproducible.

A. We apologize for the lack of these information for CAR-T/EV functional assays. In the revised Methods section, we have stated the number of biological & technical replicates and statistical methods for relevant experiments, as listed below: For flow cytometry, CLDN18.2-CAR-T cells from 4 donors' PBMC (CAR-T #1-#4) were used to assess cell exhaustion, in which 2 replicates were provided for the assessment of statistical significance in CAR-T #1 (t-test). For ELISA, in each group, 3-4 replicates were provided for the assessment of statistical significance (t-test). For antibody backfill assay and binding affinity assay, in each group, 2 replicates were provided for the assessment of statistical significance (t-test). For both cytotoxic killing and invasion assay, 3 replicates were provided in each group for the assessment of statistical significance (t-test). For animal experiments, 5 mice were ensured in each group for the assessment of statistical significance (Two-way repeated-measures ANOVA for growth curve, t-test for tumor weight, cytokine concentration and CAR-T concentration). We have also improved some statistics and adjusted current plots to better demonstrate the replicates in the revised Figures.

Reviewer #4 (Remarks to the Author):

The authors have responded to most of my comments. I respectfully disagree with some of their assumptions, for example just because NTA is used widely and as such one may list a vast amount of literature, however these publications do not show that the method is both accurate or has no limitations. The authors should consider this. It would behoove the authors that they inform the reader on limitations of their assay for diagnostic purposes. Indeed as the auhtors do not see a need for technical validation with spike-inns, their CPP score is research use only and has not been rigorously validated as potential diagnostic test that for use in the clinic. They should make the reader aware of this important difference and not inflate their results. The functional experiments do however support the diagnostic potential of the EVs, and it should be communicated as such. In addition figure 1d remains confusing where EV-array and EV-elisa are compared, have the auhtors performed an outlier test, i could not find this.

A. Thank you for your thoughtful review and for highlighting these important points. We appreciate the opportunity to clarify and refine our manuscript in response to your feedback.

1. Regarding the use and limitations of Nanoparticle Tracking Analysis (NTA)

We fully acknowledge your point that the widespread use of a method does not equate to its infallibility or absence of limitations. By assessing the particle size of isolated EVs, NTA was employed in our study as one component of a multi-step EV characterization strategy, alongside transmission electron microscopy (TEM) for the shape of isolated EVs, Western blot analysis for MISEV-aligned EV markers, and immunogold labeling for the expression of specific EV-proteins that we decided to proceed for functional & mechanistic analysis. All methodological details for these analyses have been described in the previous version of the response letter and incorporated in the revised manuscript. Here, we provide a workflow to better illustrate all analyses related to EVs in this study (**Fig for review only. 1**).

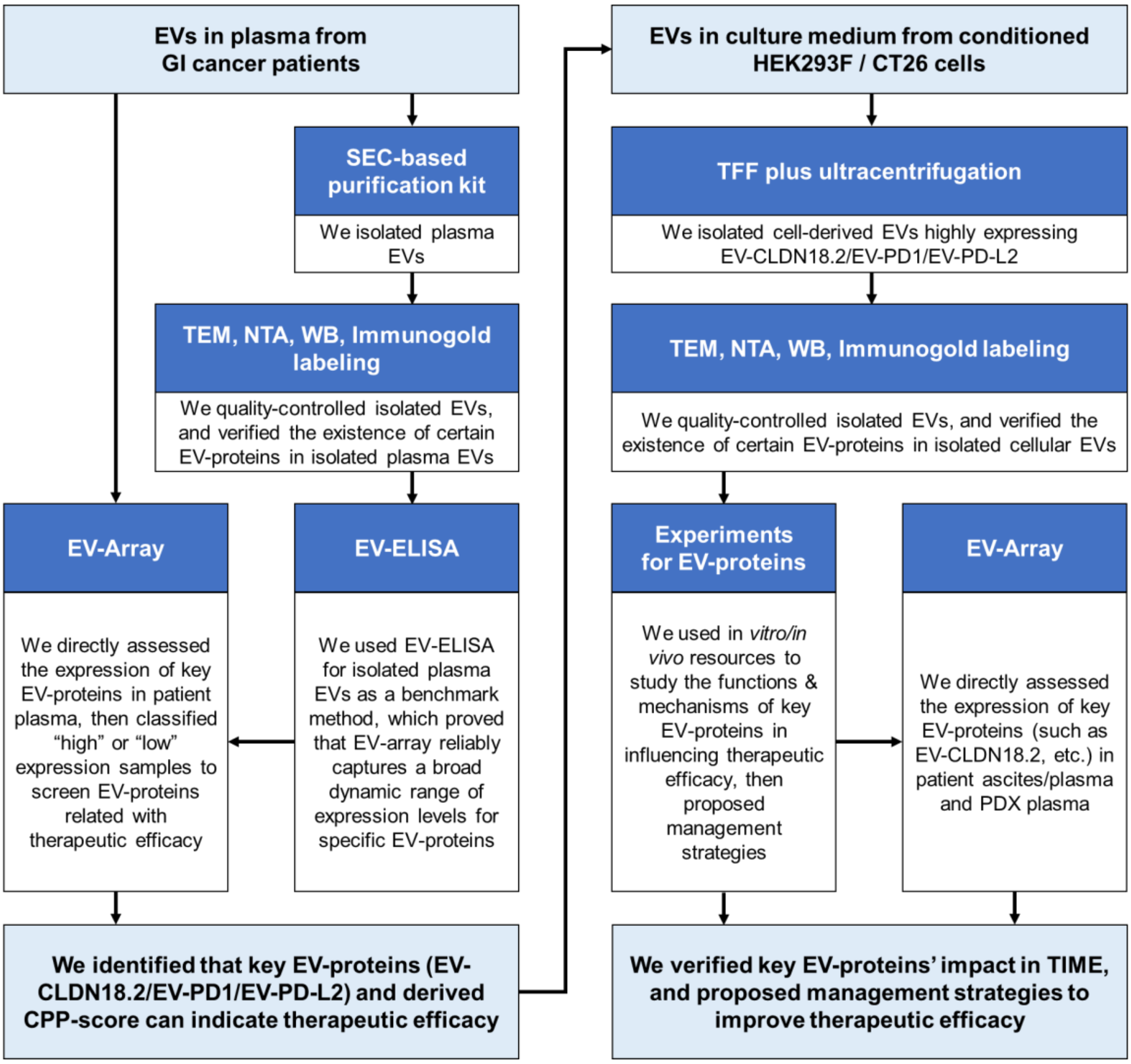

Fig for review only. 1

In the revised manuscript, we have incorporated a statement in the Methods section to explicitly address the known technical considerations of NTA, including its sensitivity to sample preparation and its primary use for estimating size distribution and particle concentration rather than assessing absolute purity. The statement of methods for NTA has been improved as follows: "For NTA assay, isolated EVs were observed using the ZetaView PMX 110 (Particle Metrix, Meerbusch, Germany) powered by a 405 nm laser to measure particle size and quality. Of note, while NTA is widely used for estimating EV size distribution and concentration, its results are known to be sensitive to sample preparation and should not be interpreted as an indicator of absolute EV purity." We believe that transparent acknowledgment of these methodological limitations can now enhance the rigor and interpretability of the study.

We would also like to respectfully clarify that this study is intended as a translational investigation addressing clinically relevant questions rather than a methodological study focused on optimization of EV isolation or characterization techniques. The experimental approaches used in this work follow currently accepted standards in the field. The experimental approaches used in this work follow currently accepted standards in the field. While we acknowledge the inherent limitations of existing EV methodologies, we believe these limitations do not materially affect the validity of our main findings or their translational significance.

2. Clarification of the research-use status of the CPP-score and its diagnostic potential

We agree completely that our CPP-score, a signature derived from counting the "high" expressions of EV-CLDN18.2 plus EV-PD1 minus EV-PD-L2, is presented as a research tool and has not been validated as a clinical diagnostic test. To prevent any overstatement, we have revised the text throughout the manuscript, particularly in the Results and Discussion sections, to clearly state that the current findings are for research use only. We now emphasize that "However, at this stage, CPP-score is presented as a reference and has not yet been validated for clinical diagnostic use. Further rigorous validation—including the incorporation of spike-in controls, assessment of inter-laboratory reproducibility, and evaluation in larger independent cohorts—will be required before considering any potential clinical applications". We thank you for this critical suggestion, which helps position our work accurately.

3. Clarification of Figure 1d (EV-array vs. EV-ELISA comparison) and outlier analysis

We apologize for the lack of clarity regarding the statistical approach in Figure 1d. As we presented in **Fig for review only. 1**, the primary intent of EV-ELISA was to demonstrate that the EV-array captures a broad dynamic range of expression levels, not to claim its quantitative concordance with EV-array. The raw replicate data for the ELISA measurements (for both EV-CLDN18.2 and EV-PD1) are shown below in **Fig for review only. 3**. We performed Pearson correlation analysis to evaluate the overall trend of EV-CLDN18.2/EV-PD1 between EV-ELISA and EV-array, which revealed a consistent pattern of low-to-high expression across the two platforms. Therefore, we used EV-ELISA as a benchmark method to prove that EV-array reliably captures a broad dynamic range of expression levels for specific EV-proteins. We have now improved the descriptions of **Figure 1d** in 3rd revision to better clarify these points.

Regarding your question about an outlier test: We did not perform a formal outlier test (e.g., Grubbs' test) because the observed dispersion is consistent with the expected variability between two fundamentally different detection platforms (the array-based multiplexing (EV-array) versus plate-based immunoassay (EV-ELISA)). The correlation, while not perfect, was statistically significant and sufficient to support our conclusion that the EV-array provides a reliable semi-quantitative profiling tool. Please refer to the next response point for the methodological details and characterization of EV-ELISA.

4. Methodological details and characterization of EV-ELISA

In last (2nd) revision, we have already thoroughly described the purpose of design and methodological details of EV-ELISA. Here we are more adequately presenting our responses for your information.

- Detailed methodology for EV-ELISA

Plasma EVs for ELISA were isolated with a SEC-based purification kit (EV40T, EVbio, China). Information for the kit was provided below. Isolated plasma EVs were diluted to a final volume of 100 µL with coating buffer (0.1 M carbonate, pH=9.6) and incubated on ELISA plates overnight at 4℃. Following incubation, the plates were washed with PBST and blocked with 100 µL of 5% BSA for 60 minutes at room temperature. The samples were then incubated with primary antibodies for CLDN18.2 (ab241330, Abcam)/PD1 (ab52587, Abcam) for 2 hours at room temperature. After another round of

PBST wash, the plates were incubated with a goat-anti-mouse IgG secondary antibody for 1 hour at room temperature. Subsequently, 100 µL of TMB substrate solution was added and allowed to react for 30 minutes. The reaction was stopped by adding 100 µL of stop solution, and the optical density (OD) was immediately measured at 450 nm.

- Assessment of EV-ELISA's reliability

We mixed 5 plasma EV samples isolated from 5 GC patients, then determined the operational range of ELISA by testing the dilution series of mixed EVs (0.15625-10 µL isolated EVs, diluted into 100 µL volume with coating buffer). According to the standard curve, both EV-CLDN18.2 and EV-PD1 displayed strong linear correlations (R^2>0.9, **Fig for review only. 2**) between the input EV volume and the signal intensities, confirming that ELISA is capable of precisely quantifying variations in EV-protein levels within a calibrated sample range (0.15625-10 µL). This established range ensures all subsequent measurements were performed under quantitatively reliable conditions. Therefore, appropriate doses of EVs (2 µL for CLDN18.2 and 4 µL for PD1) were determined for subsequent ELISA assessment in multiple isolated plasma EV samples.

	Exp value by ELISA-Replicates					
Mixed plasma volume (µL)	CLDN18.2	CLDN18.2	CLDN18.2	mean	SD	CV
10	1.418	1.415	1.222	1.351667	0.112305	0.083086
5	0.513	0.453	0.521	0.495667	0.037166	0.074982
2.5	0.338	0.314	0.326	0.326	0.012	0.03681
1.25	0.192	0.18	0.176	0.182667	0.008327	0.045584
0.625	0.122	0.11	0.131	0.121	0.010536	0.087072
0.3125	0.086	0.082	0.086	0.084667	0.002309	0.027276
0.15625	0.075	0.069	0.072	0.072	0.003	0.041667

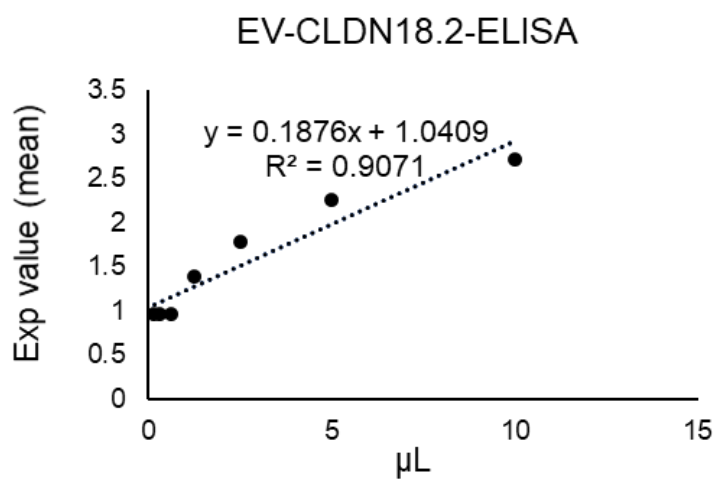

	Exp value by ELISA-Replicates					
Mixed plasma volume (µL)	PD1	PD1	PD1	mean	SD	CV
10	3.027	3.165	3.048	3.08	0.074357	0.024142
7.5	2.682	2.787	2.672	2.713667	0.063705	0.023476
5	2.271	2.46	2.056	2.262333	0.202139	0.08935
3	1.722	1.857	1.755	1.778	0.070378	0.039582
2	1.268	1.428	1.45	1.382	0.099338	0.07188
1	0.582	0.951	1.34	0.957667	0.379044	0.395799

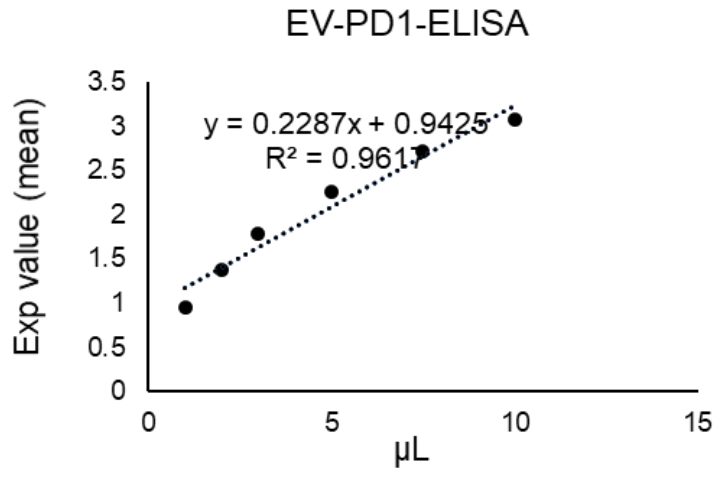

Fig for review only. 2

- Raw data of replicates

In the 1st revision, we have compared the expressional correlations of ELISA and EV-array for EV-CLDN18.2 and EV-PD1. The raw data for replicates of ELISA for EV-CLDN18.2 and EV-PD1 are provided here in **Fig for review only. 3.**

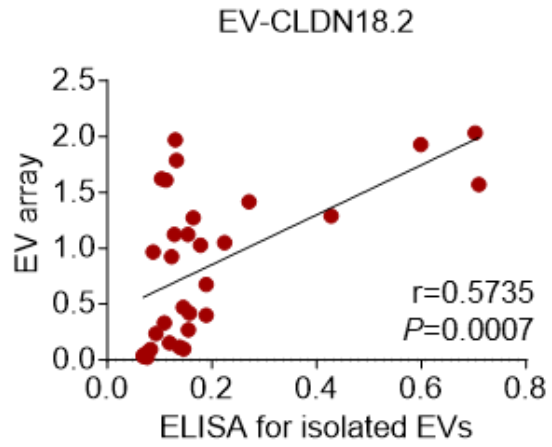

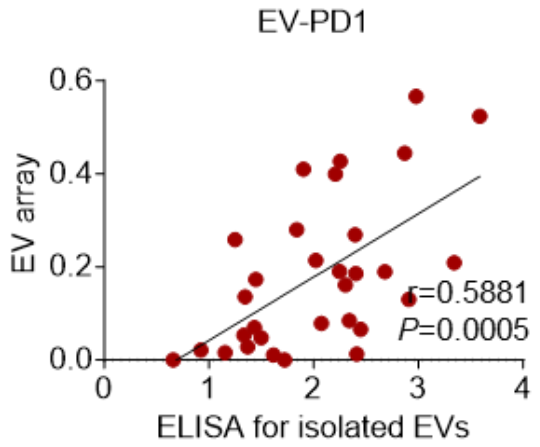

ELISA-CLDN18.2				
Plasma ID-CLDN18.2	Replicate 1	Replicate 2	Replicate 3	**Mean**
Plasma 1	0.099	0.087	0.094	**0.093**
Plasma 2	0.176	0.138	0.149	**0.154**
Plasma 3	0.084	0.072	0.074	**0.077**
Plasma 4	0.075	0.066	0.065	**0.069**
Plasma 5	0.166	0.135	0.135	**0.145**
Plasma 6	0.136	0.170	0.131	**0.146**
Plasma 7	0.080	0.081	0.088	**0.083**
Plasma 8	0.119	0.116	0.121	**0.119**
Plasma 9	0.137	0.106	0.092	**0.112**
Plasma 10	0.266	0.310	0.237	**0.271**
Plasma 11	0.160	0.165	0.168	**0.164**
Plasma 12	0.150	0.160	0.154	**0.155**
Plasma 13	0.136	0.139	0.140	**0.138**
Plasma 14	0.106	0.094	0.111	**0.104**
Plasma 15	0.686	0.751	0.692	**0.71**
Plasma 16	0.257	0.227	0.187	**0.224**
Plasma 17	0.124	0.134	0.131	**0.130**
Plasma 18	0.069	0.070	0.067	**0.069**
Plasma 19	0.448	0.435	0.402	**0.428**
Plasma 20	0.724	0.732	0.653	**0.703**
Plasma 21	0.115	0.128	0.125	**0.123**
Plasma 22	0.111	0.111	0.106	**0.109**
Plasma 23	0.185	0.188	0.195	**0.189**
Plasma 24	0.126	0.119	0.140	**0.128**
Plasma 25	0.192	0.186	0.190	**0.189**
Plasma 26	0.054	0.173	0.245	**0.157**
Plasma 27	0.172	0.172	0.189	**0.178**
Plasma 28	0.641	0.579	0.576	**0.599**
Plasma 29	0.131	0.129	0.135	**0.132**
Plasma 30	0.091	0.089	0.085	**0.088**
Plasma 31	0.075	0.076	0.077	**0.076**

ELISA-PD1				
Plasma ID-PD1	Replicate 1	Replicate 2	Replicate 3	**Mean**
Plasma 1	1.306	1.42	1.28	**1.335333**
Plasma 2	1.504	1.391	1.141	**1.345333**
Plasma 3	3.595	3.597	3.57	**3.587333**
Plasma 4	1.11	0.855	1.496	**1.153667**
Plasma 5	2.52	2.428	2.075	**2.341**
Plasma 6	1.714	1.386	1.748	**1.616**
Plasma 7	1.812	1.355	1.176	**1.447667**
Plasma 8	2.264	2.289	2.19	**2.247667**
Plasma 9	0.857	0.945	0.968	**0.923333**
Plasma 10	3.379	3.226	3.422	**3.342333**
Plasma 11	1.605	1.525	1.367	**1.499**
Plasma 12	1.983	1.965	2.114	**2.020667**
Plasma 13	3.077	3.119	2.739	**2.978333**
Plasma 14	2.313	1.346	2.198	**1.952333**
Plasma 15	2.402	2.595	2.191	**2.396**
Plasma 16	1.571	1.756	1.843	**1.723333**
Plasma 17	2.242	2.256	2.122	**2.206667**
Plasma 18	2.99	2.669	2.382	**2.680333**
Plasma 19	0.68	0.653	0.642	**0.658333**
Plasma 20	2.91	2.994	2.829	**2.911**
Plasma 21	2.322	1.941	1.962	**2.075**
Plasma 22	2.885	2.934	2.788	**2.869**
Plasma 23	2.278	1.498	1.936	**1.904**
Plasma 24	2.351	1.287	1.583	**1.740333**
Plasma 25	1.416	1.09	1.246	**1.250667**
Plasma 26	2.404	2.236	2.271	**2.303667**
Plasma 27	2.641	2.392	2.32	**2.451**
Plasma 28	2.463	2.265	2.506	**2.411333**
Plasma 29	2.602	2.465	2.146	**2.404333**
Plasma 30	2.047	1.804	1.667	**1.839333**
Plasma 31	1.585	1.163	1.365	**1.371**

Fig for review only. 3

- The reason to present ELISA data

In response to your question regarding EV-array versus EV-ELISA, our primary objective was to identify a direct and widely accepted approach to validate that the EV-array reliably captures a broad dynamic range of expression levels for specific EV-associated proteins. Accordingly, we used ELISA as a benchmark method for the validity of EV-array, and applied Pearson correlation analysis to demonstrate that the distributions of low-to-high EV-CLDN18.2/EV-PD-1 expression were highly consistent between EV-ELISA and EV-array measurements. Given that EV-array and ELISA rely on distinct detection principles, it is reasonable that the two methods exhibit similar overall trends while retaining certain quantitative differences.

We acknowledge that the concordance between EV-array and EV-ELISA results is not optimal; however, it is sufficient to prove that EV-array detects a broad range of expression for EV-proteins. Given that all the result-oriented assessments and analyses were performed using the EV-array and are supported by multiple lines of clinical & experimental evidences, the inclusion of EV-ELISA validation does not change the conclusions or overall interpretation of the study.

We deeply appreciate your comment that our description about EV-array and ELISA may be confusing. Thus, we have improved the descriptions in the manuscript (for Fig. 1d, Extended Data Fig. 2k) to "ELISA validation for CLDN18.2/PD1 expression in EVs isolated from 31 random plasma samples in

discovery cohort confirmed that EV expression array reliably captures a broad dynamic range of expression levels for specific EV-proteins".
